# Harnessing the Estradienone Scaffold to Develop Dual GPBAR1 and LIFR Modulators for Liver Fibrosis

**DOI:** 10.1021/acs.jmedchem.5c00705

**Published:** 2025-09-24

**Authors:** Rosa De Gregorio, Federica Moraca, Pasquale Rapacciuolo, Bianca Fiorillo, Elva Morretta, Cristina Di Giorgio, Silvia Marchianò, Ginevra Lachi, Carmen Massa, Benedetta Sensini, Michele Biagioli, Lucio Spinelli, Maria Chiara Monti, Bruno Catalanotti, Valentina Sepe, Stefano Fiorucci, Angela Zampella

**Affiliations:** † Department of Pharmacy, 9307 University of Naples “Federico II”, Via D. Montesano, 49, I-80131 Naples, Italy; ‡ Department of Medicine and Surgery, 60250 University of Perugia, Piazza L. Severi 1, 06132 Perugia, Italy

## Abstract

Fibrosis is a pathological process characterized by excessive deposition of the extracellular matrix (ECM) within tissues. Chronic fibrotic disorders involving the lungs, liver, intestine, and kidneys represent a major cause of morbidity and mortality and remain a major unmet therapeutic need. In the liver, the development of pathological ECM depends on the activation of key cell targets, i.e., the hepatic stellate cells (HSC). HSCs express the leukemia inhibitory factor receptor (LIFR), which promotes fibrosis, and a bile acid-activated receptor, GPBAR1, which attenuates HSC activation. Herein, we report the design and synthesis of a new class of 4,9-estradien-3,17-dione derivatives acting as dual LIFR inhibitors and GPBAR1 agonists. *In silico* and pharmacological characterization of these dual modulators led to the identification of compound **2o** as a first-in-class LIFR/GPBAR1 modulator that reverses liver fibrosis in vitro and in vivo. These findings demonstrate the therapeutic potential of LIFR/GPBAR1 hybrid molecules in human fibrotic disorders.

## Graphical abstract

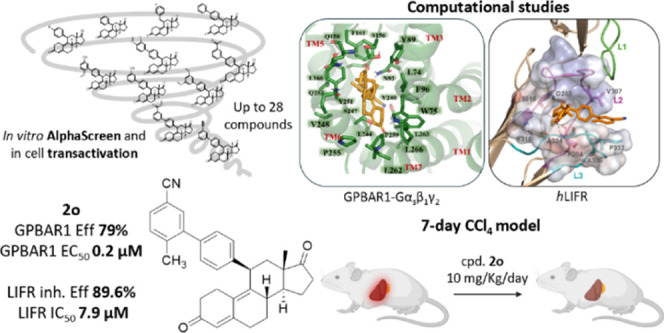

## Introduction

Fibrosis is a pathological condition associated with several inflammation-driven-diseases,^1^ resulting in structural damage and functional impairment.^2,3^ The pathogenesis of fibrosis is multifactorial^4,5^ and involves a complex interplay of several cell types within the extracellular matrix (ECM), including immune cells such as monocytes/macrophages and leukocytes. These cells secrete pro-fibrotic mediators, ultimately leading to recruitment and activation of fibroblasts, which further contribute to the inflammatory milieu by producing cytokines and chemokines such as IL-6, CCL-2, IL-17, TNF-α, TGF-β, and collagen.^6^

The leukaemia inhibitory factor (LIF) is a member of the IL-6 family of cytokines, which also includes IL-11, ciliary neurotrophic factor, cardiotrophin-1, and oncostatin M.^7–10^ LIF is expressed in various cell types, including hepatocytes, adipocytes, megakaryocytes, neurons, myocytes, embryonic stem cells, and osteoblasts, and plays a crucial role in inflammation, autoimmunity and cancer.^11^ LIF exerts its function by binding to a ubiquitously expressed receptor, LIFR,^12^ inducing a conformational change that facilitates the recruitment of glycoprotein (gp)­130, leading to the formation of an active heterodimeric (LIFRβ/gp130) signaling complex.^13^ The binding of LIF to LIFR activates several intracellular downstream signals, including the JAK/STAT, MAPK, and PI3K/AKT, which are essential for regulating key cellular processes, such as proliferation, survival, self-renewal, and differentiation.^14^ Therefore, LIFR inhibitors represent a promising approach to treat a number of human diseases, and both anti-LIFR antibodies and small-molecule inhibitors have been developed.^15–17^ A series of patented derivatives acting as LIFR inhibitors with anticancer effects based on a 4,9-estradien-3-one backbone with specific C11 and C17 substitutions has been recently identified. Among these compounds, EC359 emerged as the most potent first-in-class LIF-LIFR inhibitor, demonstrating significant in vitro and in vivo efficacy in triple-negative breast cancer (TNBC) models, reducing invasiveness, stemness, and promoting apoptosis.^18,19^

Using a drug repurposing approach, we screened FDA-approved drugs and identified mifepristone (RU-486, Figure 1) as a potential LIFR inhibitor^20^ and confirmed its efficacy by in vitro studies on pancreatic cancer cell lines. Structural modification of EC359 and mifepristone led to the development of novel derivatives, among which LRI201 (Figure 1) exhibited the most promising inhibitory profile. Its pharmacological potential was further validated in gastric cancer models.

**Figure 1:**
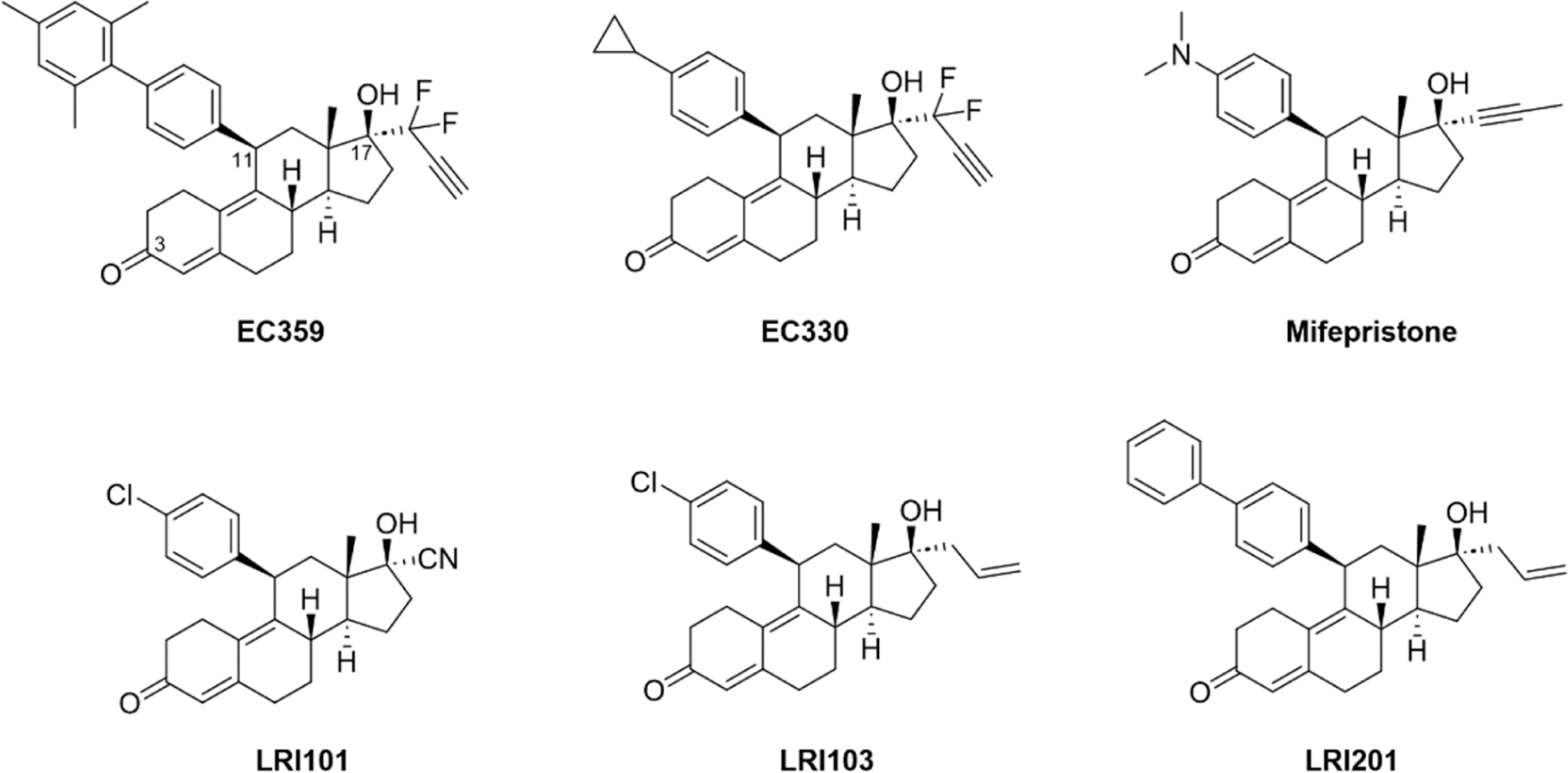
4,9-Estradien-3-one LIFR inhibitors previously identified.^10,18,20^

A significant breakthrough was the discovery of a series of in-house bile acid derivatives as LIFR inhibitors.^21,22^ Notably, several secondary bile acids were found to be not only G protein-coupled bile acid receptor 1 (GPBAR1) agonists but also natural LIFR inhibitors, identifying deoxycholic acid (DCA) and its glyco- and tauro-conjugated forms (GDCA and TDCA), and 3-oxodeoxycholic acid (3-oxoDCA) as the most potent endogenous inhibitors of LIFR of the entire series.

Therefore, we envisaged the possibility of developing a novel library of 4,9-estradien-3,17-dione derivatives with enhanced dual or even triple modulatory properties targeting LIFR, GPBAR1, and Farnesoid X Receptor (FXR).

Both GPBAR1 (also known as TGR5) and FXR are bile acid-activated receptors that play significant and complex roles in the progression and potential treatment of liver fibrosis.^23,24^ FXR is a nuclear receptor primarily expressed in the liver and intestine, which is crucial for regulating bile acid homeostasis. FXR exerts an antifibrotic effect by regulating various cellular pathways, including protecting hepatocytes and inhibiting the activation of hepatic stellate cells (HSCs), which are key drivers of fibrosis.^25^ FXR activation can also modulate inflammation and lipid metabolism, contributing to its protective role against liver injury and fibrosis (Figure 2). Dysfunction or downregulation of FXR is often associated with the progression of liver diseases, including fibrosis.^26^ GPBAR1 is a G protein-coupled receptor activated by bile acids, widely distributed across various organs and tissues, including the liver, although in contrast to FXR, it is not expressed by hepatocytes but is mostly detected in HSC, endothelial cells, and liver macrophages.^27–29^ GPBAR1 agonist exhibits anti-inflammatory effects and can confer protective effects against many liver diseases.^30,31^ GPBAR1 activation alleviates diabetic liver fibrosis, inhibits the contraction of HSCs, and improves intrahepatic microcirculation.^32^ It also promotes the secretion of glucagon-like peptide-1 (GLP-1) from intestinal L cells, which has antidiabetic and antifibrotic properties.^33,34^ Accordingly, both FXR and GPBAR1 are considered potential therapeutic targets for liver diseases, and dual FXR/GPBAR1 agonists are being explored, as their combined activation might offer synergistic benefits in treating conditions like metabolic dysfunction-associated steatohepatitis (MASH),^35^ which can progress to fibrosis and cirrhosis.

**Figure 2:**
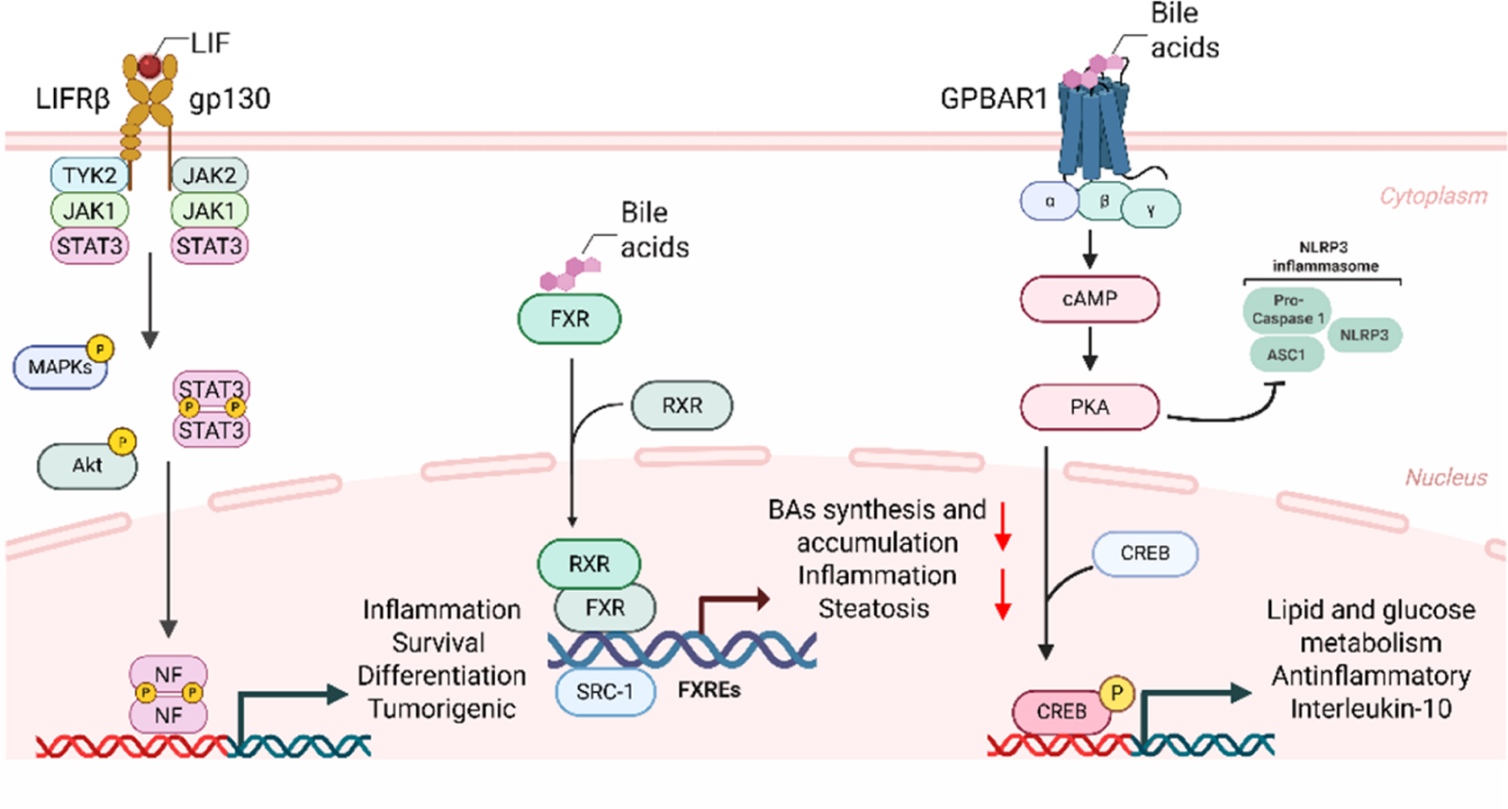
Some of the biological roles of LIFR, GPBAR1, and FXR. Created in BioRender. Rapacciuolo, P. (2025) https://BioRender.com/v1tq1rm.

The potential of such multitarget ligands to act as integrated modulators of interconnected signaling pathways is further illustrated in Figure 2, which highlights the therapeutic relevance and mechanistic rationale for the development of dual or triple modulators in complex disease models.

Here, we therefore report the synthesis of new compounds with optimized activity by simplifying the scaffold through the introduction of a ketone at the C17 position and refining modifications at C11.

Comprehensive synthetic, pharmacological, and computational studies led to the identification of compound **2o** as a potent GPBAR1 agonist and LIFR inhibitor. This represents the first synthetic dual modulator of GPBAR1 and LIFR, offering a promising therapeutic strategy for liver fibrosis, one of the major causes of morbidity and mortality.^23^

## Chemistry

As for the synthesis of the whole library, the key intermediate compound **2** was synthesized as previously reported.^10^ Compounds **3** and **4** were prepared by Grignard reaction in *dry* THF using copper­(I) chloride and either biphenylmagnesium bromide or 4-(*N*,*N*-dimethyl)­aniline magnesium bromide, respectively. Finally, the treatment with acetic anhydride and dimethylaminopyridine (DMAP) in pyridine, followed by repartition with HCl 6 N, allowed us to obtain C5 elimination and C3 deprotection in a single reaction step, with the formation of compounds **1a** and **1b** (Scheme 1, panel A). Following the same procedure, compounds **1c–1e** were also obtained (Scheme 1, panel B).

**Scheme 1:**
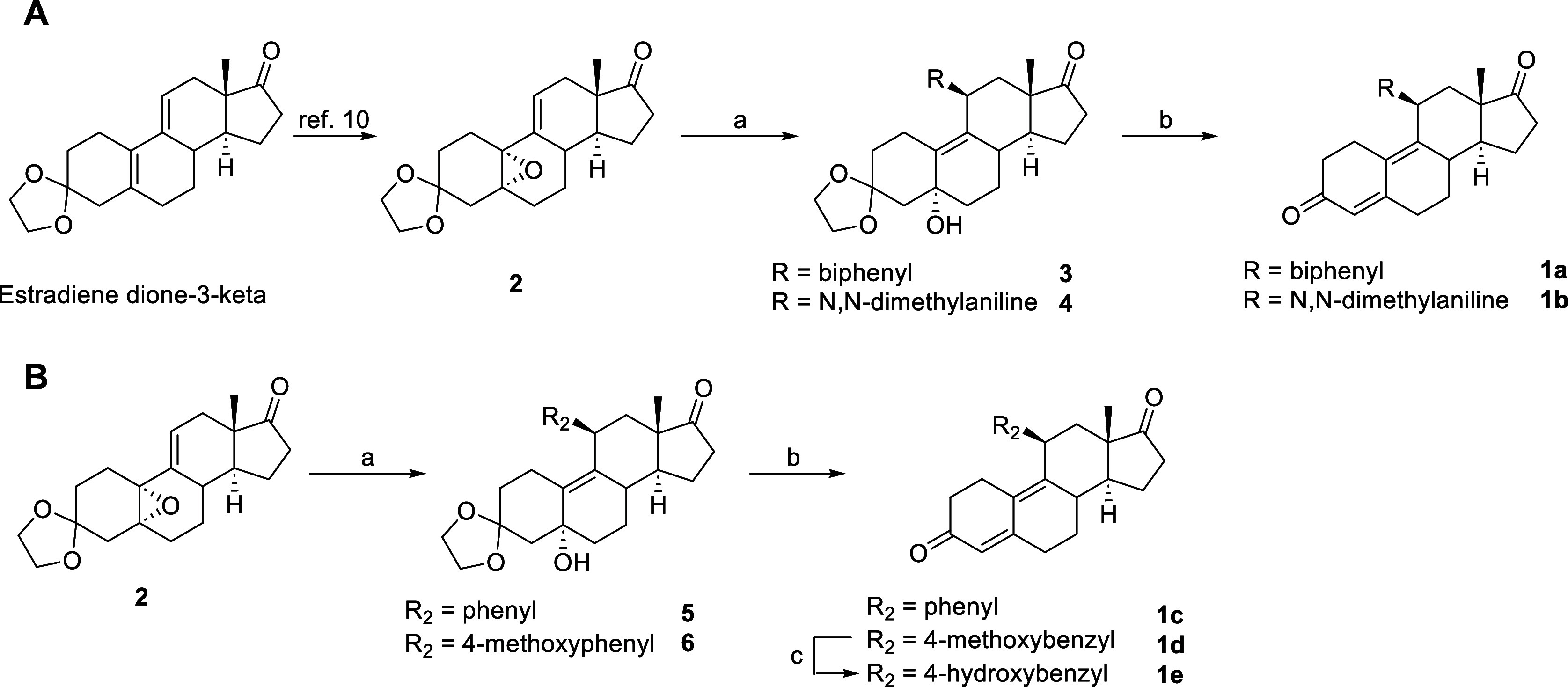
Synthesis of Compounds **1a–1e** ^a^

a Reagents and conditions. (a) RMgCl (2.2 equiv), CuCl (0.9 equiv), dry THF, 0 °C, 52–70%; (b) anhydride acetic (6 equiv), DMAP (0.2 equiv), pyridine, 60 °C, then HCl 6 N, 45–72%; (c) BBr_3_ (1.5 equiv), dry DCM, −20 °C, 44%.

Alternatively, the addition of 1,4-diiodobenzene was carried out in the presence of isopropylmagnesium chloride and CuCl. This reaction consists of the synthesis of 4-iodophenylmagnesium chloride in situ and is based on metal exchange occurring between iPrMgCl, a highly reactive Grignard reagent, and 1,4-diiodobenzene. Compared to the conventional Grignard procedure, the reaction requires milder conditions and overcomes the drawback of handling magnesium. Thus, this method was used to obtain (4-iodophenyl)magnesium chloride, which then reacted with copper chloride to give compound **7** by 1,4-addition (Scheme 2). The key intermediate **7** was then subjected to dehydration and the C3 deprotection reaction to yield compound **1f**. Finally, by applying Suzuki coupling and employing different boronic acids, we readily obtained a large library of C11-substituted compounds (Scheme 2).

**Scheme 2:**
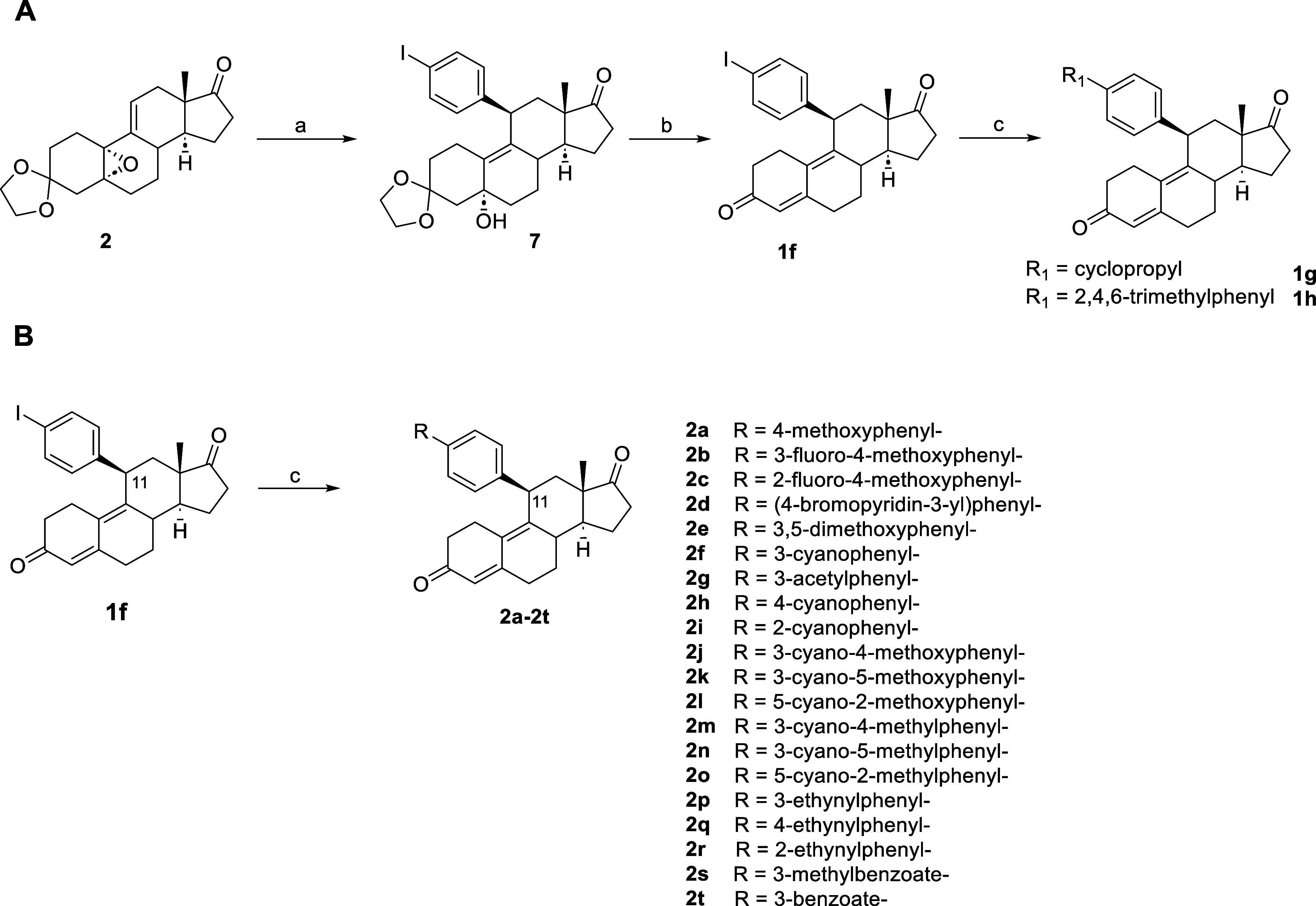
Synthesis of Compounds **1f–1h** and **2a–2t** ^a^

a Reagents and conditions. (a) 1,4-Diiodobenzene (2.2 equiv), CuCl (0.9 equiv), iPrMgCl (2.2 equiv), dry THF, 0 °C, 52%; (b) anhydride acetic (6 equiv), DMAP (0.2 equiv), pyridine, 60 °C, then HCl 6 N, 70%; (c) different boronic acids (2 equiv), Pd­(PPh_3_)_4_ (0.015 equiv), K_2_CO_3_ (2 equiv), toluene/MeOH (1:1), 20–90%.

## Results and Discussion

As of late, our research group has intensified its interest in LIF-LIFR, aiming to develop novel LIFR inhibitors (LRIs). We have previously reported the discovery and pharmacological characterization of a 4,9-estradien-3-one derivative, compound LRI201,^10^ as a proof of concept for the druggability of LIFR receptor. This compound, together with its analogues, LRI101 and LRI103, presented a similar in vitro inhibitory profile compared to other known LIFR ligands such as mifepristone, EC359, and EC330 (Figure 1).^19,20,36^

To further explore the LIFR chemical space and identify better LIFR inhibitors, we tested compound **1a**, a precursor in LRI201 synthesis, in the AlphaScreen in vitro assay. This decision was guided by our computational studies on LRI201 and Mifepristone, which suggested that a hydrogen bond acceptor at position C17 could play a significant role. Surprisingly, **1a** maintained the inhibitory activity on the LIF-LIFR axis, as reported in Table 1 (inhibition activity 62.4% and 76.4% at 10 and 50 μM, respectively, and with an IC_50_ of 1.42 μM).

**Table 1:**
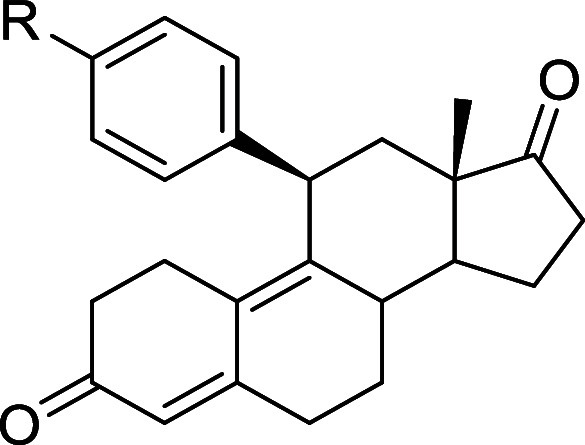

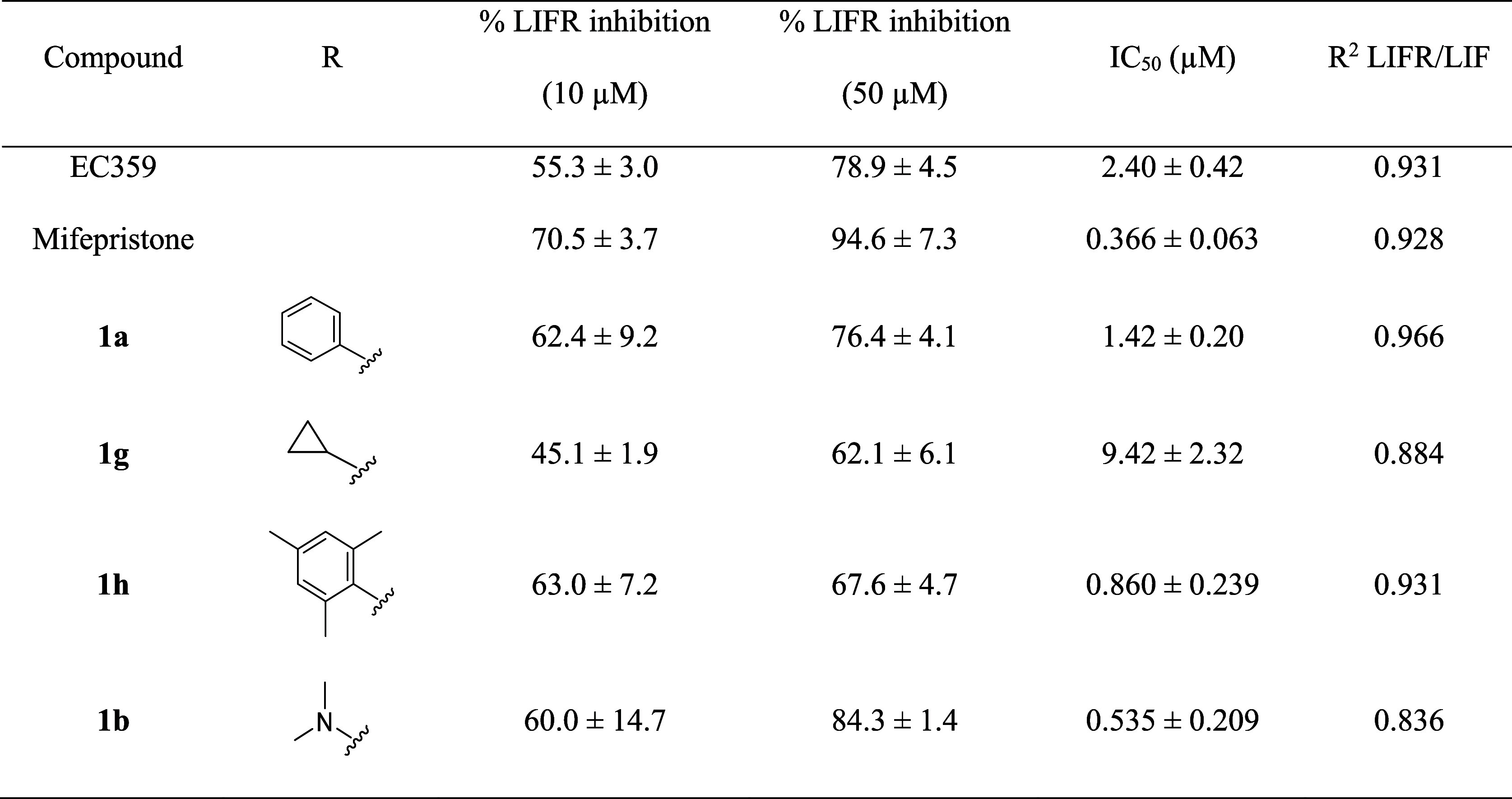
Inhibition Activity of LIF-LIFR Interaction by Cell-Free *AlphaScreen* Assay of Compounds **1a**, **1b**, **1g**, **1h** ^a^

a Data showcase molecule efficacies (i.e., % LIFR inhibition with standard deviations) and potencies (i.e., IC_50_ values with standard deviations and *R* ^2^ values).

Consequently, the development of a new library of LRIs through scaffold simplification became the logical next step. Our efforts focused on identifying additional substitutions, particularly at the C11 position, to further enhance the inhibitory activity. For this reason, we began synthesizing simplified compounds by introducing a ketone at the C17 position and removing previously added substituents while focusing exclusively on modifications at the C11 position. First, we decided to synthesize the analogues of EC330, EC359, and mifepristone (Scheme 1, compounds **1b**, **1g**, **1h**).

Initially, a cell-free assay based on an AlphaScreen platform was set.^21,22,37,38^ The assays were carried out using a concentration range from 1.37 nM to 200 μM and the efficacy and potency of LRIs in inhibiting LIF-LIFR interaction were compared to those of two well-characterized LIFR inhibitors (EC359 and mifepristone).^18–20^ The signal generated by the LIF-LIFR interaction in the absence of any compound was set as a 100% interaction. The in vitro efficacy and potency results are summarized in Table 1 and in Figure S1, panel A.

Since structural and synthetic simplification did not result in a loss of activity, our efforts focused on the synthesis of a large library of compounds with different substitutions at the C11 position.

We thus obtained a subset of compounds, substituted with differently decorated phenyl or biphenyl groups at C11, which were tested on our AlphaScreen platform as inhibitors of the LIF-LIFR interaction in terms of both efficacy and potency. As clearly emerges from Table 2, the best hits were compounds **2e** and **2f** (Figure S1, panels B–D).

**Table 2:**
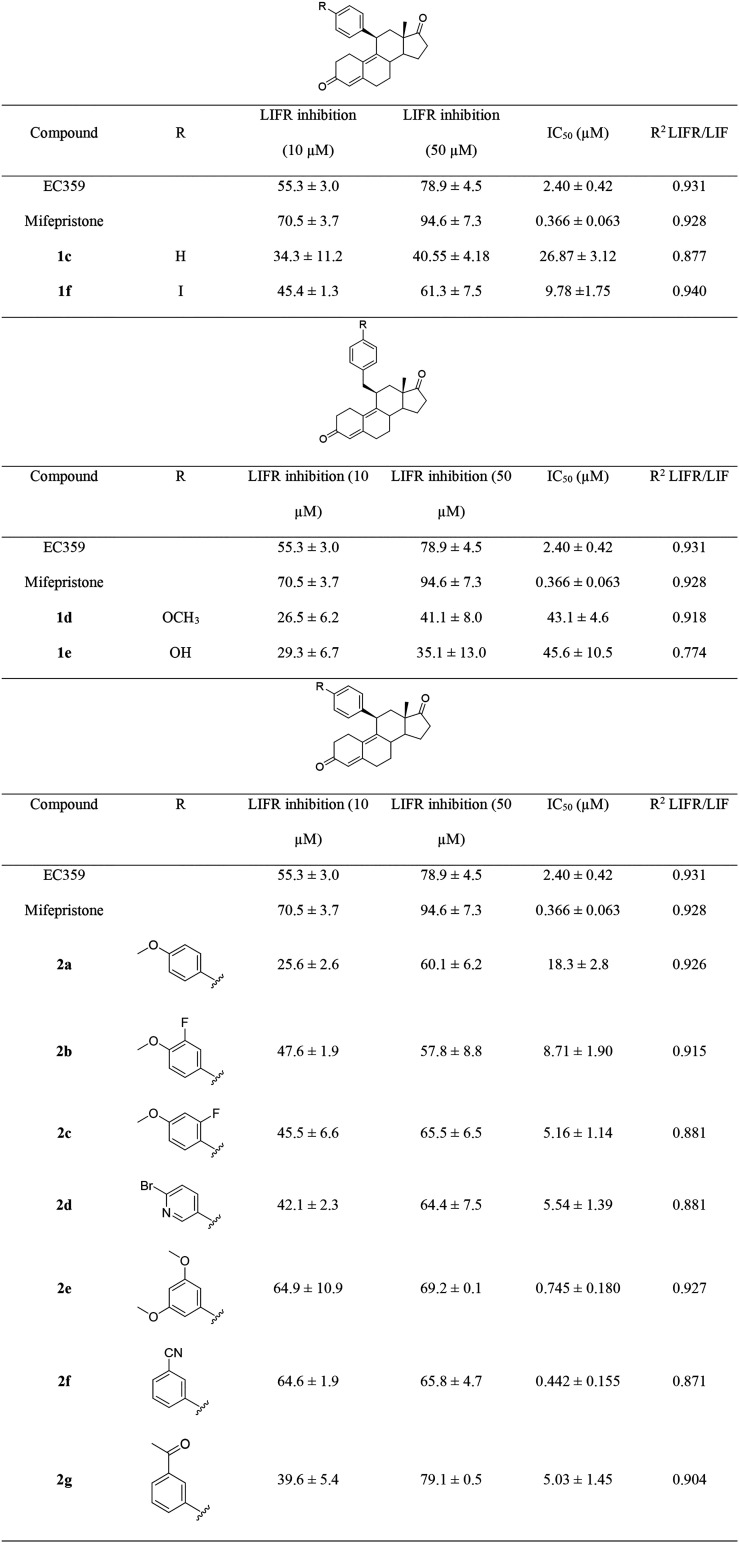
Inhibition Activity of the LIF-LIFR Interaction by Cell-Free *AlphaScreen* Assay of Compounds **1c–1f** and **2a–2g** ^a^

a Data showcase molecules’ efficacies (i.e., % LIFR inhibition with standard deviations) and potencies (i.e., IC_50_ values with standard deviations and *R* ^2^ values).

The introduction of the more flexible benzyl group at C11 led to a drastic reduction in both efficacy and potency toward LIFR (see compounds **1d** and **1e**). Moreover, the substitution of the dimethylamine moiety with iodine (compound **1f**) was detrimental to both efficacy and potency, while the removal of the dimethylamine substituent resulted in a loss of activity (see **1c**).

The presence of the *p*-methoxy group (compound **2a**) caused a 10-fold reduction in activity compared to that of compound **1g**. However, the simultaneous presence of fluorine with the *p*-methoxy group, as in **2b** and **2c,** was less detrimental, especially when the F atom was positioned in the *ortho* position (IC_50_ = 5.16 μM for **2c** compared to 8.71 μM for **2b**). Interestingly, the presence of the 3,5-dimethoxy groups slightly improved activity with respect to the parent compound (IC_50_ = 0.745 μM for **2e** compared to 0.860 μM for **1h**).

Additionally, other groups were introduced at the meta position, such as cyano and acetyl groups, leading to compounds **2f** and **2g**. While compound **2g** exhibited a weaker inhibitory activity towards LIFR, compound **2f** (IC_50_ = 0.442 μM) demonstrated the best activity profile within this whole subset, with an IC_50_ lower than that of the parent compound **1h** and established LIFR inhibitors (EC359 and mifepristone). Finally, the presence of a heterocyclic substituent (compound **2d**) resulted in weak inhibitory activity (IC_50_ = 5.54 μM).

Compounds **2e** and **2f** showed the best activity, suggesting that the presence of either the cyano group or the 3,5-dimethoxy groups is beneficial for the inhibition. Consequently, we designed more derivatives to further explore the effect of placing these substituents (methyl, methoxy, and/or cyano groups) at different positions, aiming to also improve both efficacy and potency.

We also decided to introduce the alkyne group to mimic the linear and rod-like geometry of the nitrile group, while removing its characteristic hydrogen bond acceptor property. Moreover, we explored the possible effect of introducing the carboxyl group (methyl esters and carboxylic acid). As a result, we obtained 13 new compounds (named **2h**-**2t**), which were tested using the AlphaScreen assay (Table 3 and Figure S1, panel E).

**Table 3:**
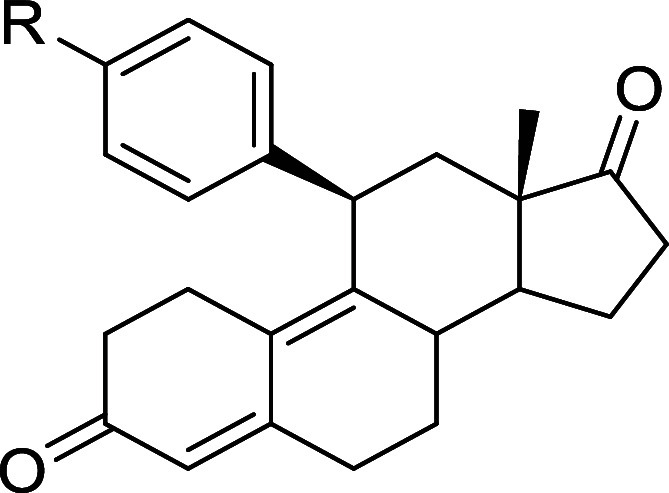

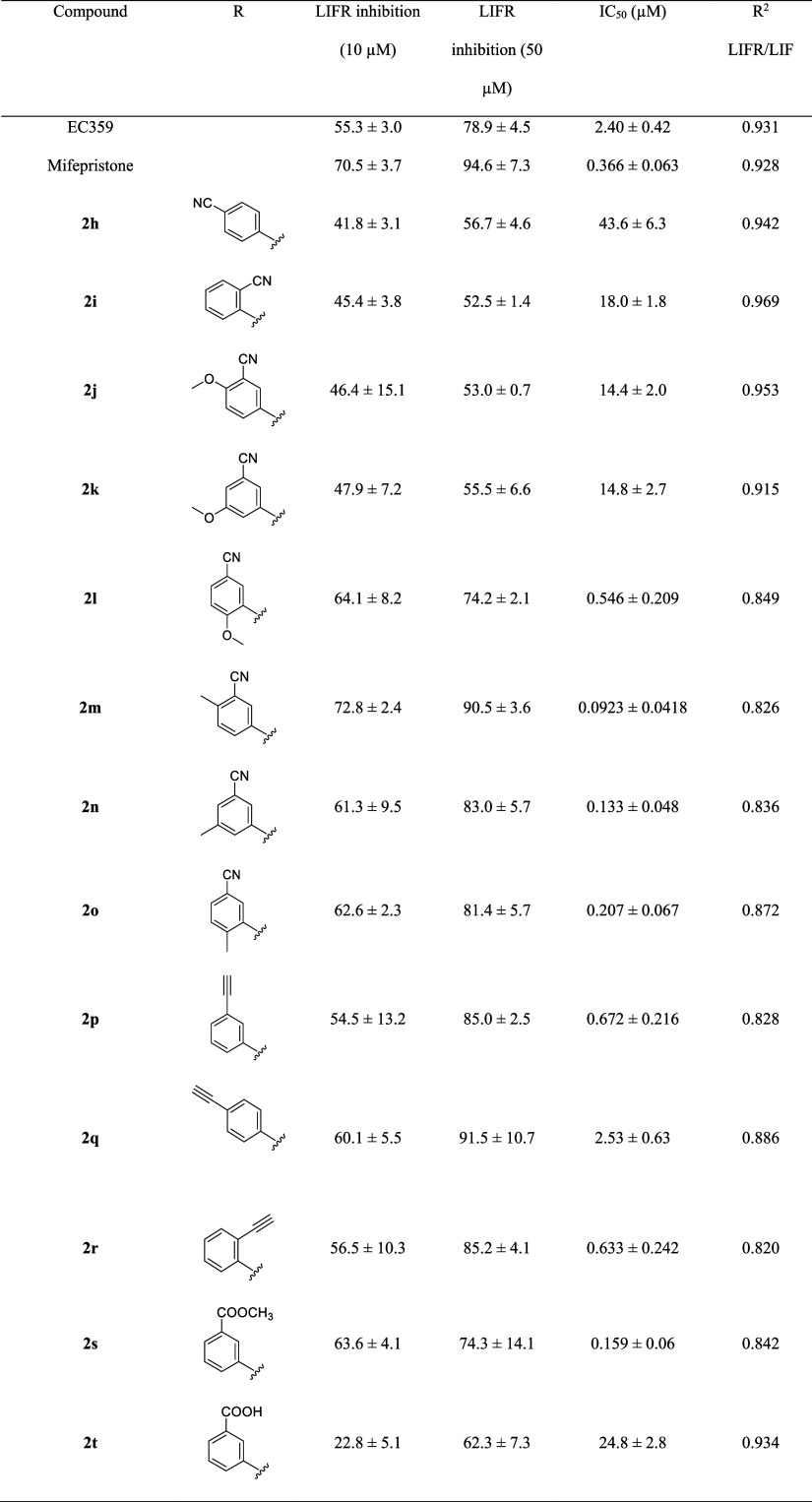
Inhibition Activity of LIF-LIFR Interaction by Cell-Free *AlphaScreen* Assay of Compounds **2h–2t** ^a^

a Data showcase molecules’ efficacies (i.e., % LIFR inhibition with standard deviations) and potencies (i.e., IC_50_ values with standard deviations and *R* ^2^ values).

The Alphascreen results allowed us to identify the best biphenyl substitutions associated with the best inhibitory activity toward LIFR.

Shifting the cyano group position proved to be detrimental, as the potency progressively decreased considering the *para* and the *ortho* positions (see **2h** and **2i**, respectively).

As for compounds **2j**, **2k**, and **2l**, the *ortho* methoxy group combined with the CN group in *meta* retained good inhibitory activity (**2l**, IC_50_ of 0.546 μM), while the presence of the methoxy group in either *meta* or *para* position was quite detrimental (IC_50_ of 14.4 and 14.8 μM for **2j** and **2k**, respectively). The simultaneous presence of the *m*-CN group and the methyl group in either *para* or *meta* position further increased the overall inhibitory activity on LIFR (**2m**, IC_50_ of 0.0923 μM and **2n**, IC_50_ of 0.133 μM, respectively) when compared to **1h** and established LIFR inhibitors, while *m*-CN and *o*-methyl substitution led to slightly lower potency (see compound **2o**, IC_50_ of 0.207 μM). Interestingly, the introduction of a *m*-methylester group (**2s**) improved the inhibitory activity with an IC_50_ of 0.159 μM compared to that of parent compound **2f** (IC_50_ of 0.442 μM). Conversely, compound **2t** performed poorly with an IC_50_ value of 24.8 μM, suggesting that the presence of a very polar acid group is detrimental to the activity. Finally, the alkyne group performed better when in position *meta* or *ortho* with IC_50_ values slightly lower than 0.7 μM (see **2q** and **2r**, respectively), while the *p*-alkyne substitution decreased the potency. Finally, considering compounds **2j** to **2o**, the presence of a methyl group proved to be more favorable than the methoxy one.

### In Vitro Transactivation

Given our previous finding that bile acids and their semisynthetic derivatives can inhibit *h*LIFR,^21,22^ and that a series of isoxazole derivatives acted as dual inhibitors of *h*LIFR and the bile acid receptor FXR,^37^ we investigated whether this new series of 4,9-estradien-3,17-dione derivatives might exhibit a dual or a triple modulatory activity, by also targeting the bile acid receptors FXR and GPBAR1.

Therefore, all compounds were also tested on FXR-SRC-1 complex by Alphascreen assay, and they resulted in being fully inactive (data not shown), and then, they were also tested in the luciferase reporter assays on HepG2 and HEK293T cells transfected with LIFR and GPBAR1, respectively, and the results are summarized in Table 4.

**Table 4:** Efficacy toward LIFR and GPBAR1 on HepG2 and HEK293T^a^

Compound	LIFR inhibition % (10 μM)	GPBAR1 eff % (10 μM)
**1a**	33.8 ± 3.7	44.8 ± 5.1
**1b**	104.0 ± 2.5	21.7 ± 0.3
**1c**	76.8 ± 6.7	35.0 ± 1.5
**1d**	92 ± 7	na
**1e**	29.1 ± 1.4	9 ± 4
**1f**	89.9 ± 1.2	na
**1g**	51.1 ± 1.4	16.5 ± 1.5
**1h**	104.7 ± 0.4	12.2 ± 2.1
**2a**	79.4 ± 1.6	na
**2b**	82.2 ± 3.4	16.8 ± 1.5
**2c**	109 ± 3	na
**2d**	88.8 ± 3.4	na
**2e**	82.3 ± 1.6	8 ± 3
**2f**	76.9 ± 2.4	7.2 ± 0.7
**2g**	84 ± 2	11.0 ± 1.4
**2h**	84.9 ± 2.1	31 ± 2
**2i**	82.3 ± 1.9	6.7 ± 1.3
**2j**	43.50 ± 0.04	42 ± 5
**2k**	77.9 ± 1.4	25.1 ± 4.6
**2l**	88.1 ± 1.2	33.0 ± 4.5
**2m**	71.2 ± 9.8	12.3 ± 3.1
**2n**	29.6 ± 2.5	29.4 ± 0.3
**2o**	89.6 ± 3.0	79.4 ± 7.0
**2p**	95.6 ± 3.4	20 ± 4
**2q**	58.7 ± 4.6	19 ± 2
**2r**	77.5 ± 7.5	12 ± 8
**2s**	80.8 ± 0.9	43 ± 4
**2t**	38.7 ± 0.6	20.4 ± 1.4

a Eff (%) is the maximum efficacy of the compound (10 μM) relative to LIF as 100% in LIFR transactivation and the maximum efficacy of the compound (10 μM) relative to TLCA (10 μM) as 100% in GPBAR1 transactivation; % values were calculated from at least three experiments. Results are expressed as mean ± SD; na means not active.

Most of the compounds exhibited a significant inhibitory efficacy toward LIFR, while only a few derivatives presented an interesting agonism on GPBAR1, as reported in Table 4.

More in detail, we identified new promising dual modulators of GPBAR1 and LIFR, namely, compounds **1c**, **2h**, **2o**, and **2s**. However, compounds **1c** and **2h** showed conflicting data between Alphascreen and cell-based transactivation assays, and they also have a moderate efficacy on GPBAR1; compound **2s** exhibited GPBAR1 efficacy higher than 50% but was not further considered as its corresponding possible hydrolysis metabolite (compound **2t**) is significantly less active. Because compound **2o** modulates both LIFR and GPBAR1 and is our best candidate as a dual derivative, we have then investigated the concentration–response curve for this compound by transactivation. As shown in Figure 3, compound **2o** inhibits LIFR with an IC_50_ of 7.9 μM (panel A) and induces GPBAR1 activity with an EC_50_ of ∼0.2 μM (panels B). Thus, its in vitro stability was evaluated in the S9 liver fraction, and it showed a half-life (*t*_1/2_) of 289.31 ± 17.05 min and a Cl_int_ of 4.0 ± 0.24 μL/min/mg (Figure S2).

**Figure 3:**
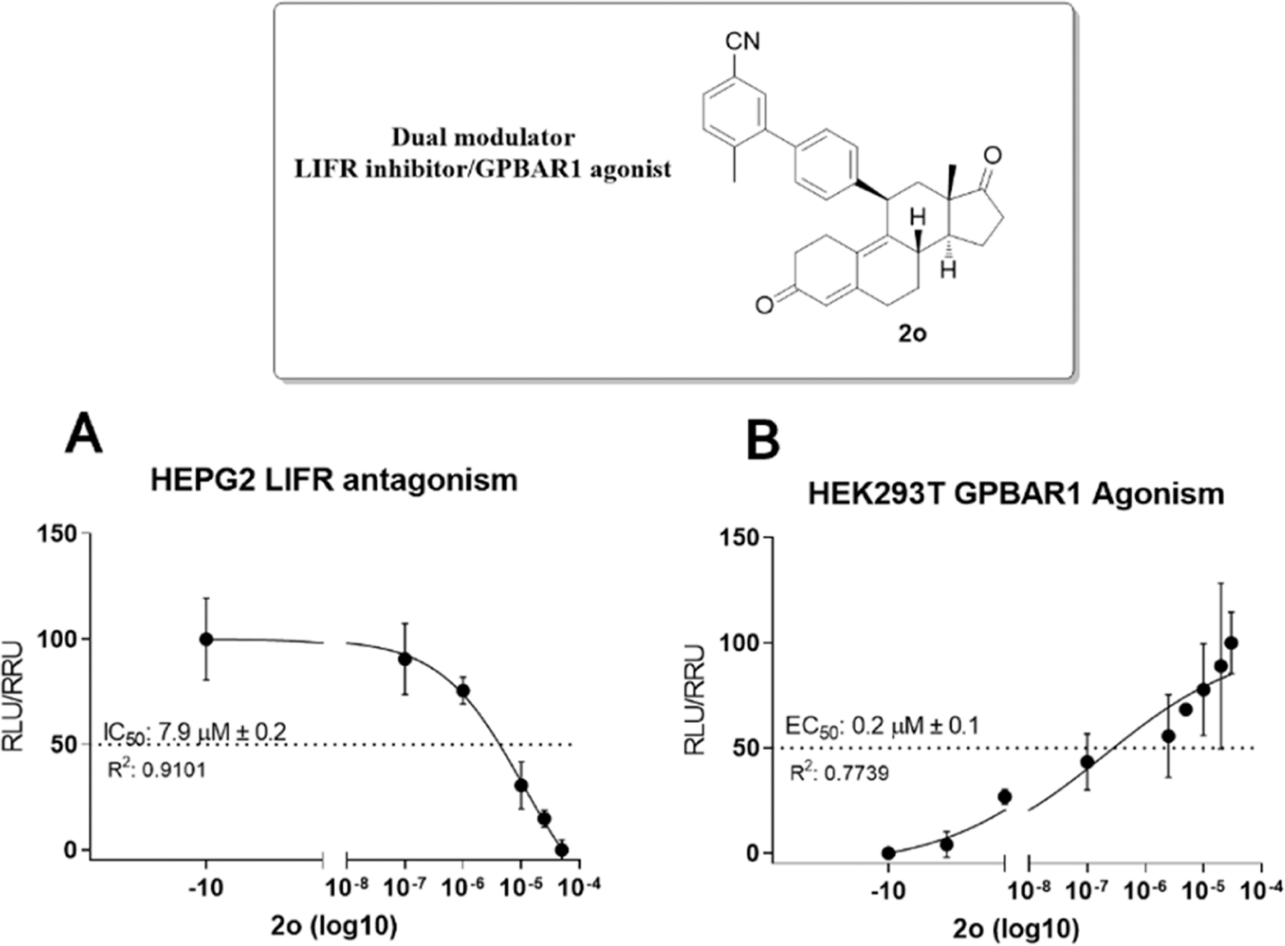
Activity profile of the best modulator of the library (**2o**).

Therefore, we have decided to deepen our computational and pharmacological studies on the dual modulator **2o** (Figure 3) with the aim of proposing a new antifibrotic drug.

### Computational Studies

To elucidate the molecular mechanism underlying the dual activity of GPBAR1 agonism and *h*LIFR antagonism, we investigated the molecular basis of these properties of **2o**, which was selected as the most promising compound of the series through docking and molecular dynamics simulations (MDs).

### Binding Mode of Compound **2o** to GPBAR1

The homology model of the active conformation of GPBAR1 in complex with Gα_s_β_1_γ_2_ protein system was built using the human cryo-electron microscopy (cryo-EM) structure with PDB ID 7CFN as a template^39^ (manuscript in preparation).

Molecular docking studies of **2o** were performed on the ligand-binding domain (LBD) of the homology model using the quantum-polarized ligand docking (QPLD) protocol and refining the best-scored pose with induced fit docking (IFD) to identify the energetically most favorable binding pose and also taking into account structural rearrangements of the LBD residues upon ligand binding. Interestingly, IFD revealed a binding mode of compound **2o** to GPBAR1, making interactions with residues known to be involved in GPBAR1 activation, like Leu71^2.60^, Tyr89^3.29^, Asn93^3.33^, Glu169^5.42^, Tyr240^6.51,^ and Ser267^7.40^ (superscripts refer to Ballesteros–Weinstein numbering)^40^ (Supporting Information, Figure S3).^39,41,42^

The stability of such interactions was monitored through 200 ns of MDs in an explicit water–lipid environment on both the compound **2o**-GPBAR1-Gα_s_β_1_γ_2_ bound system and the apo form of the receptor, allowing us to assess the receptor’s ligand-induced structural rearrangements. MDs showed a stable binding mode of **2o** with a root mean square deviation (rmsd) average of 2.20 Å, which closely resembles the initial docking orientation (Supporting Information, Figure S3). In addition, GPBAR1 remained highly stable (rmsd average of 1.41 Å, Supporting Information, Figure S4, panel A), preserving its overall conformation. On the contrary, the G protein subunits exhibited more pronounced conformational changes over the course of the MDs. This is not surprising, as the GPCR activation processes commonly induce structural rearrangements of the intracellular transducer that are essential for signaling (Supporting Information, Figure S4, panels C–E).^43,44^ Conformational cluster analysis of compound **2o** was performed on the merged trajectories of the three MD replicas (Supporting Information, SM1–3) to allow for more robust statistical analysis. The study yielded four clusters, among which the centroid structure of the most populated one (49% of the total population) (Figure 4, panel A) revealed that the estradiendione scaffold was positioned between TM2, TM5, and TM6, surrounded by hydrophobic residues, including Leu74^2.63^, Trp75^2.64^, Tyr89^3.29^, Gln253^6.64^, Pro255^ECL3^, Pro259,^7.32^ Leu262^7.35^, Leu263^7.36^, and Leu266^7.39^, all contributing to the ligand binding environment. The carbonyl oxygen at C17 formed two hydrogen bonds with Gln158^ECL2^ and Ser156^ECL2^, while the biphenyl group was oriented toward the binding cavity, in contact with Asn93^3.33^, Phe96^3.36^, Ser157^ECL2^, Phe161^ECL2^, Leu166^5.39^, Glu169^5.42^, Leu174^5.47^, Leu244^6.55^, Ser247^6.58^, Val248^6.59^, and Tyr251^6.62^, further reinforcing the interaction network and thus contributing to anchoring the ligand within the ligand binding domain (LBD). Interestingly, the biphenyl moiety underwent a rotational rearrangement, allowing the nitrile group to adopt an optimal orientation to engage a hydrogen bond with Tyr240^6.51^, further stabilizing the ligand’s binding. Notably, all the mentioned residues have been previously reported in the literature as key players in GPBAR1 activation.^38–40^ These residues contribute to ligand recognition, stabilization within the binding pocket, and subsequent conformational changes required for receptor activation. Their involvement suggests that the identified binding mode of **2o** is not only structurally relevant but also functionally significant in driving the activation process of GPBAR1.^39,41,42^ Finally, the *ortho*-methyl group on the biphenyl ring induced steric hindrance, promoting an optimal rotational adjustment of the biphenyl moiety. This conformational shift positioned the cyano group in a favorable orientation for key interactions within the binding site, thereby explaining the higher efficacy of compound **2o** compared to **2f**, which lacks this steric constraint and does not adopt the optimal binding conformation (Supporting Information, Figure S4, panel F).

**Figure 4:**
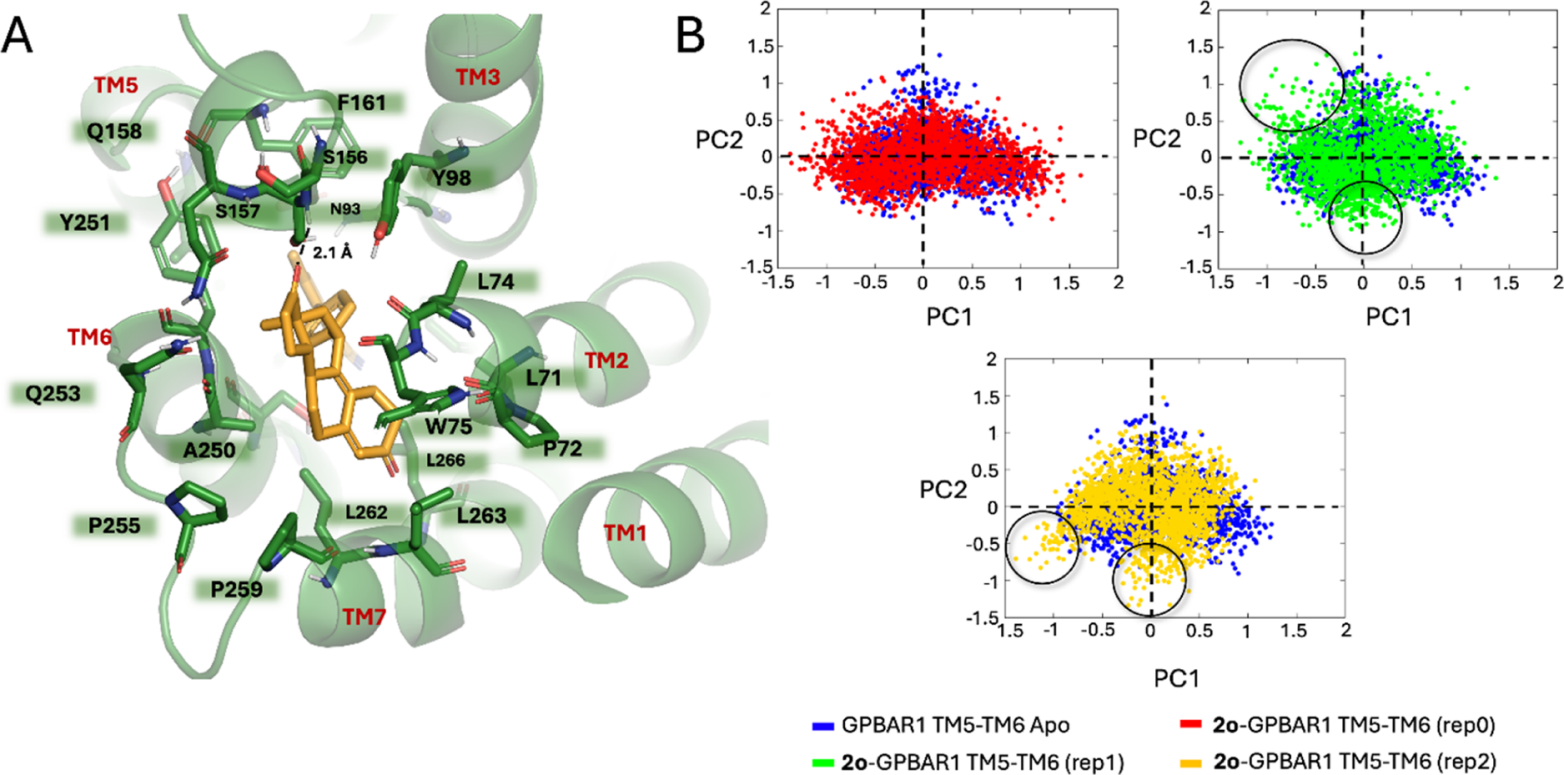
(A) Representation of the centroid retrieved from the three MD replicas of compound **2o** in the GPBAR1-Gα_s_β_1_γ_2_ homology model. The ligand is represented as orange sticks, and the interacting residues of the receptor are shown in green and labeled, with oxygen atoms in red and nitrogen in blue. The receptor is represented as ribbons with its helices labeled. Hydrogens are omitted for the sake of clarity, and H-bonds are displayed as black dashed lines. (B) Plots of the projection on eigenvectors 1 and 2 (PC1 and PC2) from the PCA analysis computed on the receptor’s TM5 and TM6 backbone of the ligand-GPBAR1-Gα_s_β_1_γ_2_ bound (rep0 highlighted in red point, rep1 in green point, and rep2 in gold point) and the apo system (highlighted in blue point). Black circles highlight the more pronounced vectorial motions of TM5 and TM6.

Ligand-induced conformational changes were further investigated by means of principal component analysis (PCA). PCA, a technique commonly used in MD simulations, allows the identification of dominant motion patterns by reducing the complexity of atomic fluctuations into a set of principal components (PCs), ranked according to their contribution to the overall system dynamics.^45^ To this end, three replica simulations of the compound **2o**-GPBAR1-Gα_s_β_1_γ_2_ complex, as well as a single simulation of the apo form of the receptor, were subjected to this analysis. In this study, PCA was performed by constructing and diagonalizing the covariance matrix of atomic fluctuations, using the backbone atoms of the receptor’s transmembrane helix (TM) 5 and TM6 as reference points (Figure 4, panel B). As shown in the PCA plot (Figure 4, panel B), the analysis revealed distinct differences in the dynamic behavior of the ligand-bound receptor versus the apo receptor. In the case of the compound **2o**-GPBAR1-Gα_s_β_1_γ_2_ complex, three independent replicas were conducted. Notably, in replicas 1 (rep1) and 2 (rep2), we observed more pronounced vectorial motions (highlighted by black circles) relative to the apo form (blue points), particularly involving an outward movement of TM5 and TM6. These motions were less marked in replica 0 (rep0), although they were still distinguishable from the apo trajectory.

These ligand-induced conformational shifts suggest a potential opening of the intracellular region of the receptor, a movement typically associated with receptor activation and G protein engagement. In contrast, the apo receptor exhibits a more restricted conformational space, indicating a structurally more rigid and less dynamic state.

Importantly, this dynamic behavior is visually represented in the Supporting Information Movie SM4, where the intracellular portions of TM5 and TM6 in the ligand-bound complex (orange) clearly move outward, whereas in the apo form (blue), the same regions display an inward movement, suggesting a closing motion. This further supports the hypothesis that compound **2o** stabilizes an active-like conformation of GPBAR1, potentially facilitating downstream signaling events.

### Binding Mode of Compound **2o** to *h*LIFR

The binding mode of compound **2o** was investigated on the unliganded conformation of the human leukemia inhibitory factor receptor (*h*LIFR) (PDB ID: 3E0G). X-ray data (PDB ID: 2Q7N) showed that the IL-6 LIF binding to the *h*LIFR occurs at the interface of loop L1-L2 of the D3 Ig-like domain, with a partial involvement of the L3 loop in the cytokine-binding module D4 domain (Supporting Information, Figure S5, panels A and B).^46^ This latter region has already been targeted as a potential binding pocket in previous works,^10,18,20,37^ allowing the design and development of *h*LIFR inhibitors. In order to deal with the high flexibility of the L3 loop, the same two-step docking protocol applied to GPBAR1 and already successfully adopted in our previous works^10,21,22,37^ was employed. In particular, QPLD docking was performed by positioning the box in the region between the L2 and L3 loop residues as previously observed for other LIFR inhibitors. The best ranked QPLD pose of **2o** obtained was used as a starting conformation for IFD, which led to the identification of two best top-ranked poses, pose A and pose B, differing primarily in the orientation of the steroid core within the binding pocket, driven by distinct conformations of the L2–L3 loops (Supporting Information, Figure S5, panels C and D, respectively). Interestingly, of the 80 IFD docking poses analyzed, a direct interaction between the cyano group and LIFR residues was observed in only 16 cases (20%), all of which were associated with higher IFD scores. Among these, the pose ranked sixth is shown in Supporting Information, Figure S5 panel E. This finding suggests that, due to the unique shape and flexibility of the L2–L3 loops, direct interaction involving the cyano group is only possible when compound **2o** adopts an energetically less favorable binding conformation. Based on this, we hypothesized that the role of the cyano group in compound **2o** may be more related to modulating the electronic properties of the molecule, particularly the biphenyl moiety, such as its electrophilicity or nucleophilicity. To explore this hypothesis, we analyzed the electronic features of compound **2o** by mapping its molecular electrostatic potential (ESP) onto the electron density surface and comparing it with that of the nonsubstituted biphenyl ring in compound **1a** (Supporting Information, Figures S6 panels A and B). The ESP map of **2o** (Supporting Information, Figure S6 panel A) reveals a red-yellow region around the cyano-substituted ring, indicating a more negative electrostatic potential due to the strong electron-withdrawing nature of the –CN group. This results in an increased electrophilicity of the aromatic ring compared to that of compound **1a**, which instead shows a slightly more nucleophilic character (Supporting Information, Figure S6 panel B). Given the higher energetic stability, both pose A and pose B were submitted to 200 MDs. Looking at the average trend of the rmsd plot along 200 ns MDs, we observed that both poses A and B of **2o** are quite stable, although a slight deviation of pose A occurred in the last 40 ns (Supporting Information, Figure S7, panel A). The solvent accessible surface area (SASA) was further analyzed as a descriptor of ligand binding, indicating that pose A is more buried than pose B, thus suggesting stronger ligand–protein interactions within the L2–L3 surface of *h*LIFR (Supporting Information, Figure S7, panel B) as reflected also by the higher number of hydrogen bonds observed during MDs (Supporting Information, Figure S8, panels A and B). Hierarchical clustering analysis was performed on MDs with the average-linkage algorithm (Supporting Information, Figure S9, panels A and B), is consistent with the rmsd trend, identifying a main binding population of pose A in the cluster0 (cl0) (Figure 5, panel B) representing approximately 74% of the simulation (Supporting Information, Figure S9, panel B). In this cluster, the ketone oxygen at C17 of the estradienone moiety was anchored via hydrogen bonds to the backbone of Asp283, not belonging to L2 or L3 loops, along with a second discontinuous hydrogen bond with the side chain Thr308 and Arg306 on L2. Additionally, hydrogen bonding between the oxygen at C3 and the backbone of Ala334 on L3 further stabilized the estradienone system within L2 and L3 (Figure 5, panel B). Notably, the time course analysis of the three main populated clusters of pose A (Supporting Information, Figure S9, panel B) revealed that the ligand continuously transitioned through three distinct binding states over time, showing substantial convergence of **2o** across the clusters regarding the hydrophobic interactions involving side chains of Pro304, Thr316, Tyr318, and Ala334 with the steroid system, as well as Val307, Ala336, and Pro337 with the biphenyl system contributed further to the ligand stabilization across the three clusters (Figure 5, panel B).

**Figure 5:**
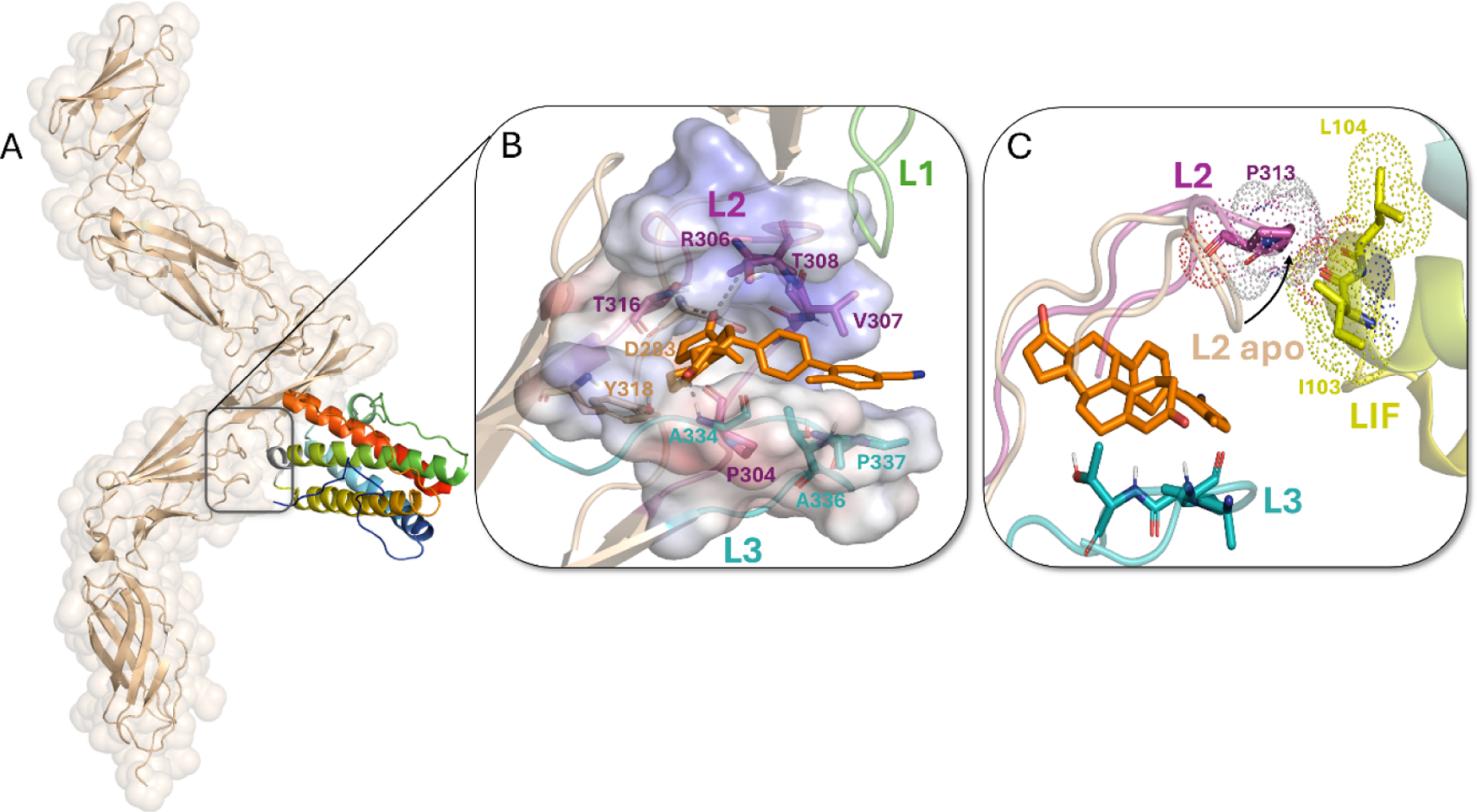
(A) X-ray structure of the D1–D5 extracellular domain of LIFR bound to the IL-6 LIF (PDB ID: 2Q7N). The rectangular selection focuses on the potential hLIFR binding site for small molecule inhibitors, already targeted in previous works. (B) Noncovalent interactions mostly occurring in cluster0 (cl0) of pose A: hydrogen bond interactions with the C17 and C3 atoms of **2o** are shown as dashed black lines, while hydrophobic/van der Waals interactions involving the estradienone and the substituted biphenyl groups are shown as surface colored by adaptive Poisson–Boltzmann solver electrostatic maps; (C) superposition between the X-ray structure of the extracellular domain of LIFR bound to the IL-6 LIF (PDB ID: 2Q7N) and the most representative cluster (cl0) of pose A found from MDs, highlighting the opening movement of L2 loop that induces, with P313, steric hindrance on LIF residues I103 and L104.

On the contrary, the average trend of the rmsd plot of pose B showed less deviations (Supporting Information, Figure S7, panel A) but the ligand is more solvent exposed as reflected by the SASA value that fluctuated around a constant average value of ∼280 Å^2^ in pose B with respect the average value of ∼200 Å^2^ of pose A (Supporting Information, Figure S7, panel B) over the MDs. This is correlated with a reduction in the hydrogen bond network interactions, as depicted by the time-dependent variations of the hydrogen donor–acceptor distances (Supporting Information, Figure S8, panel B). Hierarchical clustering analysis of pose B showed one main cluster (cl0), accounting approximately for 70% of the population, that is very similar to the conformation found in cl1 that differs from the pose of cl2, thus indicating a slightly higher variability than pose A, probably induced by the wider interactions with the solvent (Figure S9, panels C,D).

Indeed, the ketone group at position C17 formed a discontinuous hydrogen bond with the Gly312 backbone on L2, while the oxygen atom of C3 engages a hydrogen bond only in 1.62% of the entire simulation with Trp302. Furthermore, unlike the binding in pose A, pose B established less specific hydrophobic interactions than pose A, because of a more open conformation of L3 and the more solvent-exposed area, particularly with Ala336 and Pro337 of L3 (Supporting Information, Figure S10). Globally, **2o** binding in both pose A and pose B impacted the structural stability of the protein, leading to a slight increase in the rmsd average of the *h*LIFR D3–D4 domains with respect to the rmsd in the apo form (Supporting Information, Figure S11 panel A). However, this variation does not involve the L3 loop that, on the contrary, showed a higher rigidity, especially in pose A of **2o** (Supporting Information, Figure S11 panel B). Thus, to understand the dynamic behavior of specific regions of *h*LIFR upon ligand binding, root mean square fluctuations (RMSF) (Supporting Information, Figure S12) and PCA analysis (Supporting Information, Figure S13) were performed, showing that the binding of the ligand, performed on both pose A and pose B simulations, induced a different behavior of the complex with respect to the apo protein. Indeed, as demonstrated in our previous studies, ligand-induced conformational changes in the hLIFR loops near the LIF-binding region can hinder hLIF binding by introducing steric hindrance. Thus, the antagonistic mechanism of action of compound **2o** may be explained by its ability to alter the conformational dynamics of the L2 loop, particularly its opening and closing motions, thereby creating steric clashes with key residues of hLIF. This is particularly evident by superimposing the representative structure of cluster0 (cl0) of pose A obtained from the MD simulation onto the X-ray structure of the hLIFR extracellular domain in complex with LIF (PDB ID: 2Q7N) (Figure 5C). This comparison revealed that the opening motion of the L2 loop leads to spatial overlap between Pro313 of L2 and residues Ile303 and Leu304 of hLIF, likely impairing the proper binding of the cytokine (Figure 5C). This evidence from MD simulations, together with the binding stability of pose A, suggests pose A as the most likely mode of binding of **2o** to *h*LIFR.

### Evaluation of Biological Activity of Compound **2o** on Human HSC LX2

Exploiting compound **2o**, we investigated the impact of simultaneous LIFR inhibition and GPBAR1 activation on HSC, two pathways that have been implicated in the regulation of fibrogenesis and inflammation. LIF signaling has been shown to drive HSC activation and ECM deposition, contributing to liver fibrosis, while GPBAR1 activation exerts anti-inflammatory and antifibrotic effects by modulating HSC contractility, ECM remodeling, and cytokine release. For this purpose, LX2 cells were exposed to LIF alone or plus increasing concentration of **2o** (1–5–10 μM). Gene expression analysis revealed that exposure to LIF (10 ng/mL) had no effect on LIFR expression and downregulated anti-inflammatory GPBAR1. The cotreatment with **2o** (1–10 μM) upregulated LIFR and GPBAR1 expression in a concentration-dependent manner, which is consistent with its activity as a LIFR inhibitor and GPBAR1 agonist (Figure 6, panels A and B). Compound **2o** also significantly attenuated the expression of profibrotic genes, including COL1A1, ASMA, and TGFβ, all of which were upregulated by LIF exposure (Figure 6, panels C–E). The inhibitory effect was more pronounced at higher concentration (10 μM), suggesting a dose-dependent repression of fibrogenic signaling. Moreover, LIFR/GPBAR1 modulation exerted an effect on ECM remodeling markers, and **2o** reduced TIMP1 expression while increasing MMP9, indicating a shift toward enhanced ECM degradation. This regulatory pattern suggests that compound **2o** promotes matrix turnover, counteracting the pro-fibrotic effects of LIF (Figure 6, panels F and G).

**Figure 6:**
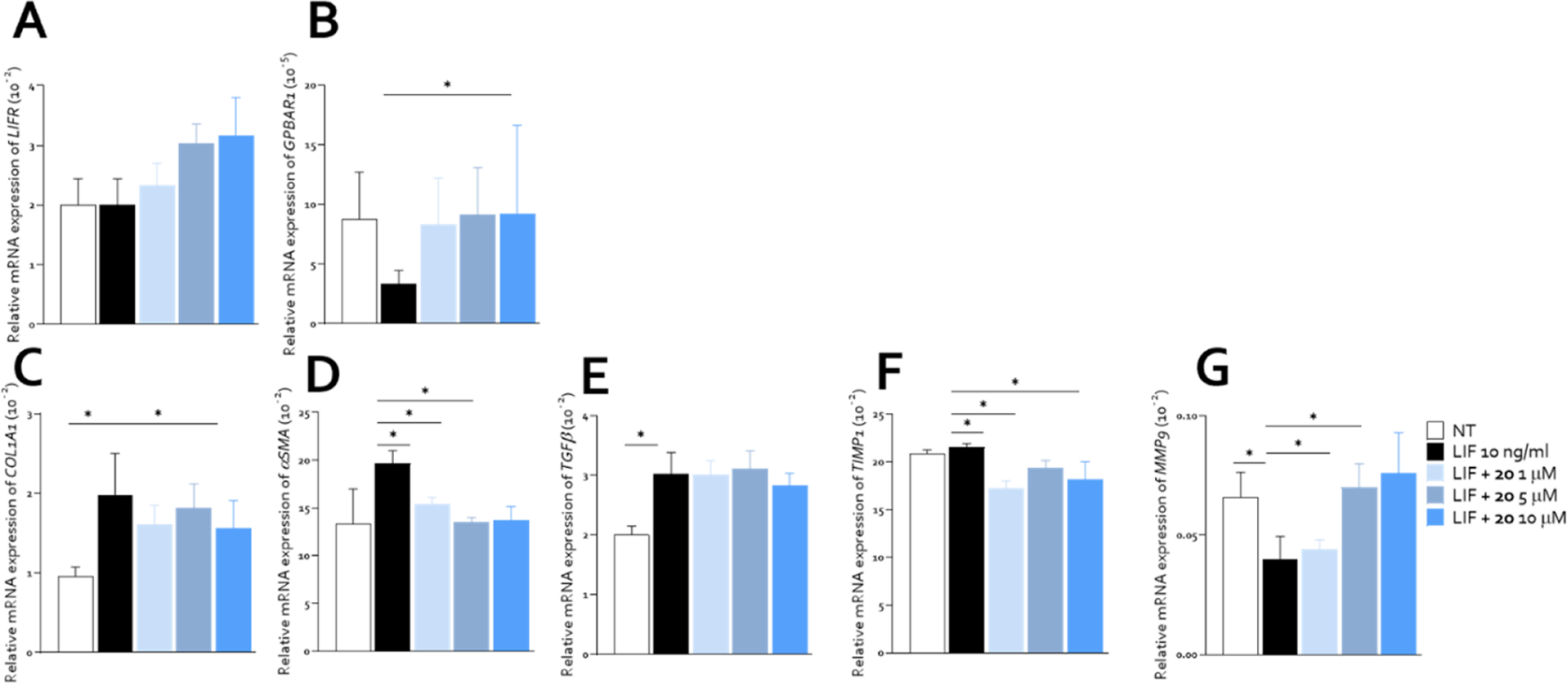
Compound **2o** exerted an antifibrotic activity on human hepatic stellate cell line LX2. LX2 were exposed to 10 ng/mL of LIF alone or in combination with **2o** (1–5–10 μM), for 24 h or left untreated. Relative mRNA expression of molecular targets: (A) LIFR, and (B) GPBAR1; (C–E) the pro-fibrotic markers: COL1A1, ASMA, and TGFβ. ECM remodeling markers: (F) TIMP1 and (G) MMP9. Each value was normalized to GAPDH. Results are the mean ± SEM of 5 samples for each group. (*Represents statistical significance: *p* < 0.05).

Finally, based on the overall results, we additionally evaluated the in vivo pharmacokinetics parameters of compound **2o**. A single oral dose of compound **2o** (10 mg/kg) produced a pharmacokinetic profile in mice with a 1.60 h half-life, 37.63 ng/mL of *C*_max,_ and 113.69 ng·h/mL area under the curve (AUC) peak (Figure 7).

**Figure 7:**
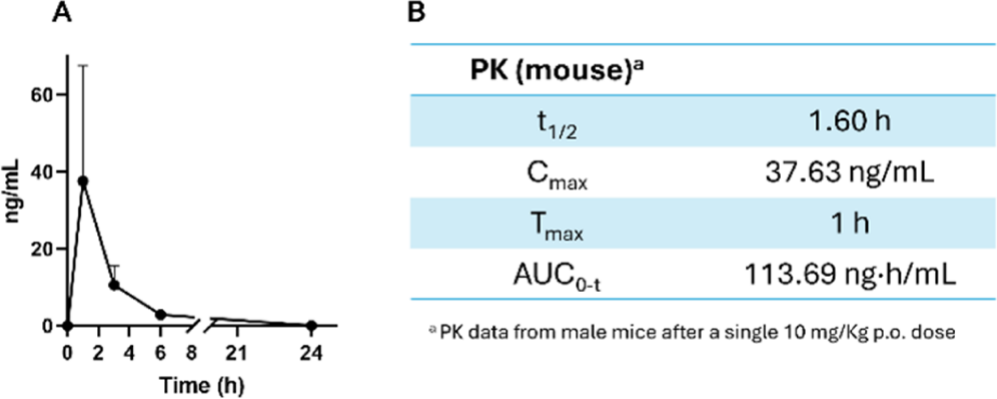
Pharmacokinetics parameters. (A) PK profile in rats after a single 10 mg/kg p.o. dose. (B) PK data. Data are the mean ± S.E.M.; *n* = 3.

### In Vivo Model

To assess whether compound **2o** counteracts hepatic stellate cell (HSC) activation in a murine model of liver fibrosis, mice were administered CCl_4_ alone or in combination with **2o** at doses of 10 mg/kg/day for 1 week (Figure 8). Carbon tetrachloride (CCl_4_) is a well-established agent for inducing liver fibrosis in mice through repeated hepatotoxic injury. In this model, liver damage leads to progressive fibrotic changes resembling human liver disease. CCl_4_ treatment alone resulted in weight loss (Figure 8, panel A), hepatocellular injury, as indicated by increased plasma levels of AST (U/L, a marker of hepatocyte damage), ALT (U/L, an enzyme released during liver cell injury), bilirubin (mg/dL, a marker of impaired hepatic clearance), LDH (U/L) (Figure 8, panel B, an indicator of tissue damage), and systemic inflammation as demonstrated by hematological analysis (Figure 8, panel C). Co-administration of compound **2o** mitigated these pathological alterations, reducing enzymatic markers of liver damage, LDH (Figure 8, panel B), WCB count (10^3^/μL), and having an effect on frequencies of neutrophils and monocytes (Figure 8, panel C).

**Figure 8:**
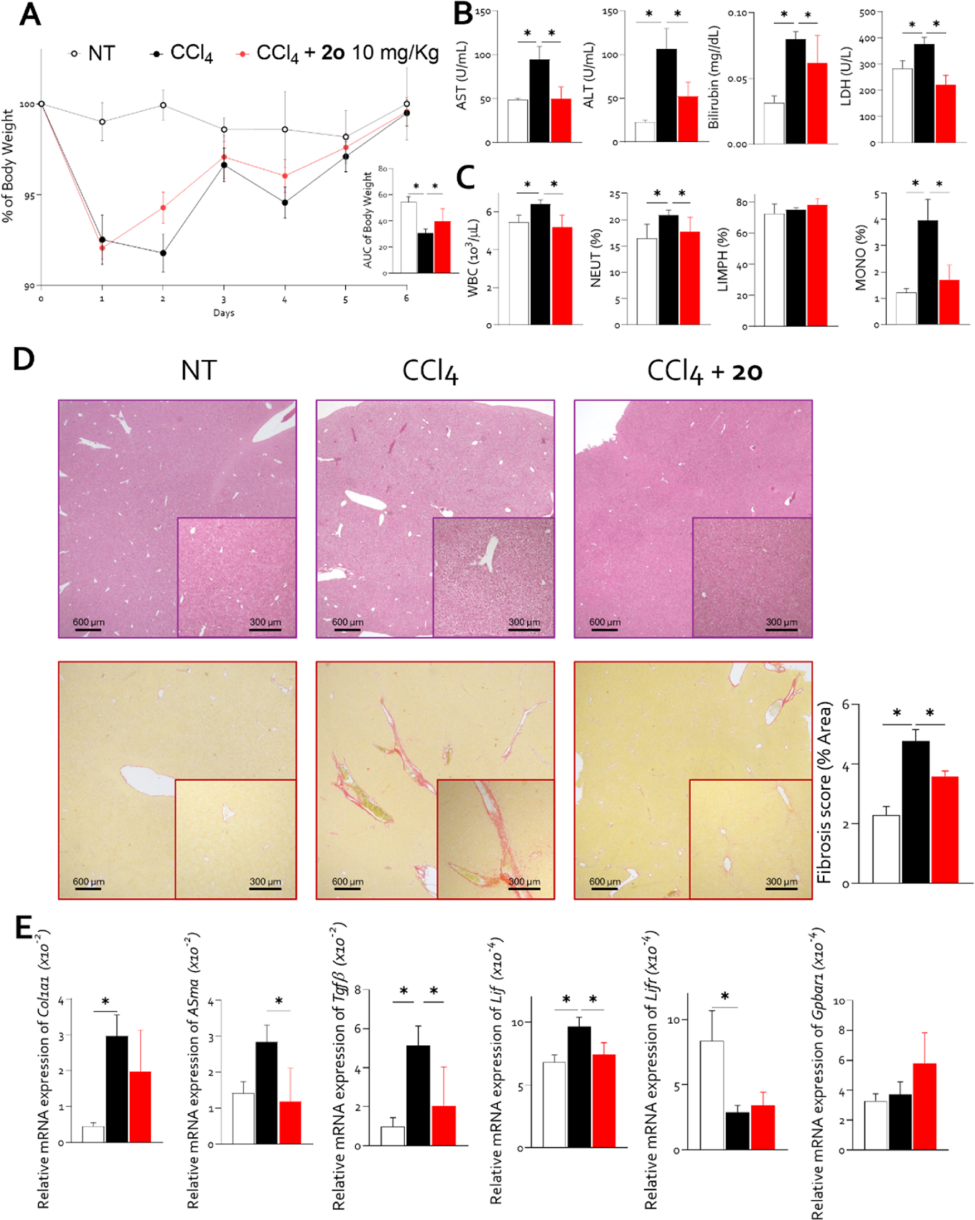
Compound **2o** exerted a protective effect against the development of liver damage and fibrosis induced by CCl_4_, improving liver function and histopathological features. Hepatic damage was induced in 12-week-old C57BL6/J male mice through administration of 0.5 mL/kg CCl_4_ twice a week, alone or in combination with 10 mg/kg of compound **2o** for 1 week. The progression of the disease was monitored by recording daily body weight. (A) % of body weight loss and AUC. (B) Severity of the disease was assessed by evaluation of AST (U/mL), ALT (U/mL), bilirubin (mg/dL), and LDH (U/mL). Hematological analysis: (C) white blood cells (WBC) (10^3^/μL), % of neutrophils, % of lymphocytes, % of monocytes in each experimental group. (D) Hematoxylin and eosin (H&E) staining (on top) and Sirius-red staining (on bottom) of liver section with fibrosis score (% of area). Relative mRNA expression of (E) fibrotic markers *Col1a1*; *aSma*; *Tg*β; molecular target: *Lif*; *Lifr*; and *Gpbar1*. Each value is normalized to GAPDH. Results are the mean ± SEM of 5–8 mice per group: *p < 0.05. Where not indicated, the data are not significant.

Histological analysis revealed that CCl_4_ exposure induced HSC activation and collagen deposition, as quantified by Sirius Red staining (Figure 8, panel D). While fibrosis induced by a 7 day CCl_4_ regimen was mild, cotreatment with compound **2o** significantly attenuated ECM remodeling (Figure 8, panel D). Furthermore, **2o** effectively reduced the expression of key fibrotic markers, including COL1a1, α-SMA, and TGFβ (Figure 8, panel E).

Moreover, to assess the residual activity on the key targets related to fibrosis, we evaluated the expression of Fxr, Shp, Lxr, Pxr, Vdr, Pparα, PPARγ, and Fgf21 by RT-PCR. As shown in Figure S14, the effect of compound **2o** in treated mice is negligible.

## Conclusion

This study presents the design of a new family of LIFR inhibitors, building on our previous computational research on mifepristone.^20^ Mifepristone was identified as a potential LIFR inhibitor through an in silico screening of FDA-approved drugs. Additionally, we explored other estradiene derivatives modified at the C11 and C17 positions within the estradiene scaffold.^10^

Considering our previously reported computational studies on mifepristone and LRI201, we further explored the LIFR chemical space to identify improved LIFR inhibitors by testing compound **1a** in an Alphascreen in vitro assay. Notably, compound **1a** retained inhibitory activity against LIF-LIFR, with an IC_50_ of 1.42 μM, despite the chemical difference at position 17, where a hydrogen bond acceptor replaced the alcohol and vinyl group.

As a result, simplifying the scaffold to develop a new library of LIFR inhibitors became the logical next step. Our goal was to identify additional substitutions, particularly at the C11 position, to further enhance inhibitory activity. The structure-guided design led to the synthesis of new 4,9-estradien-3,17-dione compounds. The synthetic protocol used was much simpler than those published previously, with fewer synthetic steps and higher yields. Notably, they diverge only in the final step, providing opportunities for further substitutions and modifications.

Although we were able to design several LIFR inhibitors, we focused our attention on the identification of dual modulators. This study led to the identification of three different compounds, see compounds **1c**, **2l**, and **2o**, endowed with good efficacy and potency toward LIFR and GPBAR1. Specifically, this study also showed for the first time that the 4,9-estradien-3,17-dione scaffold can interact with GPBAR1. MD simulations revealed the binding mode of compound **2o** in both receptors and identified key events underlying the mechanism of action. Indeed, the MD analysis of the complex of **2o** with the active conformation of GPBAR1 bound to Gα protein showed that the ligand binding event introduces new dynamic features that may be crucial for understanding the activation mechanism of GPBAR1 and its interaction with intracellular signaling partners. On the other hand, MD simulations of **2o** in LIFR showed that the ligand altered the dynamics of the loop region, specifically of L3, thereby changing the LIF binding site.

Furthermore, results from the in vitro transactivation assays and the in vivo model of liver fibrosis confirmed the therapeutic potential of dual receptor modulators of LIFR and GPBAR1. Compound **2o** significantly attenuated the expression of profibrotic genes, including COL1A1, ASMA, and TGFβ, in the LX2 cells. Moreover, compound **2o** promoted a substantial reduction of collagen deposition in a widely used model of liver fibrosis and exerts a protective effect against the development of liver damage. The CCl_4_ model has been extensively used to validate potential clinical candidates for treating liver fibrosis, and, along with in vitro testing on HSCs, is considered a robust preclinical indicator of potential clinical efficacy.^1,47^ Thus, the efficacy of compound **2o** in reversing liver fibrosis in these models strongly advocates its further testing.

Taken together, these results suggest that compound **2o** is a promising novel therapeutic candidate targeting LIFR and GPBAR1, justifying further development for treating liver fibrotic disorders.

## Experimental Section

### General Experimental Information

All chemicals were purchased from Sigma-Aldrich or Zentek srl, and solvents and reagents were used as supplied from commercial sources, with some exceptions. Tetrahydrofuran and dichloromethane were distilled from calcium hydride immediately prior to use. All reactions were carried out using flame-dried glassware. Reaction progress was monitored via thin-layer chromatography (TLC) on Alugram silica gel G/UV254 plates. The purification of synthesized compounds was carried out by flash chromatography on Biotage Selekt and by prep Agilent HPLC 1290 Infinity II system equipped with VWD or RID detector, specific column, and gradient are specified for each compound below. NMR spectra were obtained on a Bruker 400 spectrometer and recorded in CDCl_3_ (δ_H_ = 7.26, δ_C_ = 77.0 ppm). *J* are reported in hertz (Hz), and chemical shifts (δ) are in ppm and referred to CHCl_3_ as internal standards. Spin multiplicities are given as s (singlet), d (doublet), t (triplet), m (multiplet), or ovl (overlapped). High-resolution ESI-MS spectra were obtained with an Orbitrap analyzer equipped with a Vanquish Flex LC (Thermo Fisher Scientific). The purity of all final compounds was determined to be greater than 95% by analytical HPLC analysis via a Knauer system equipped with AZURA P 6.1L Analytical HPLC Pumps and AZURA RID 2.1L Refractive Index Detector, as indicated in the Supporting Information.

Compound **2** was synthesized as previously reported in the Supporting Information.^10^

### Grignard Reaction to Afford Compounds **3**, **4**, **5**, and **6**

In a solution of **2** in *dry* THF was added CuCl (0.9 equiv). The reaction mixture was flushed with N_2_, and then cooled to −10 °C. Grignard reagent (2.2 equiv) was added dropwise over a period of 15 min. The reaction mixture was stirred at RT for about 4 h. Then, the reaction was cooled and quenched by the addition of a saturated NH_4_Cl solution. The reaction was extracted with ethyl acetate (×3), and the organic layers were washed with brine, dried over Na_2_SO_4_, filtered, and concentrated in vacuo. The crude was then purified by flash chromatography in a mixture of hexane and ethyl acetate (6:4) to afford the final compounds.

#### 11β-Biphenyl-3,3-ethylenedioxy-5α-hydroxyestra-9-en-17-one (**3**)

Grignard reagent: 4-(biphenyl)­magnesium bromide. Yield: 70%. Appearance: yellow solid. ^1^H NMR (CDCl_3_, 400 MHz): δ 7.58 (d, *J* = 9.0 Hz, 2H), 7.51 (d, *J* = 8.0 Hz, 2H), 7.43 (t, *J* = 9.0 Hz, 2H), 7.32 (3H, m), 4.37 (d, *J* = 7.5 Hz, 1H), 4.03–3.88 (m, 4H), 2.53–2.34 (ovl, 5H), 2.09–2.02 (ovl, 4H), 1.93 (m, 1H), 1.88–1.80 (ovl, 2H), 1.74–1.63 (ovl, 3H), 1.60–1.55 (ovl, 2H), 1.30 (m, 1H), 0.60 (s, 3H). HR ESIMS *m*/*z*: 467.2508 [(M + H) – H_2_O]^+^, C_32_H_37_O_4_–H_2_O requires 467.2581.

#### 11β-(4-*N*,*N*-Dimethylaniline)-3,3-ethylenedioxy-5α-hydroxyestra-9-en-17-one (**4**)

Grignard reagent: 4-(*N*,*N*-dimethyl)­aniline magnesium bromide solution. Yield: 55% appearance: yellow solid. ^1^H NMR (CDCl_3_, 400 MHz): δ 7.06 (d, *J* = 8.6 Hz, 2H), 6.64 (d, *J* = 8.6 Hz, 2H), 4.24 (d, *J* = 7.0 Hz, 1H), 4.05–3.89 (m, 4H), 2.91 (s, 6H), 2.49–2.39 (ovl, 3H), 2.35–2.28 (m, 2H), 2.18–2.07 (ovl, 2H), 2.01–1.98 (ovl, 2H), 1.84–1.76 (m, 3H), 1.70–1.65 (ovl, 2H), 1.55–1.46 (ovl, 3H), 1.30 (m, 1H), 0.52 (s, 3H). HR ESIMS *m*/*z*: 434,2688 [(M + H) – H_2_O]^+^, C_28_H_38_NO_4_–H_2_O requires 434.2690.

#### 3,3-Ethylenedioxy-5α-hydroxy-11β-phenylestra-9-en-17-one (**5**)

Grignard reagent: phenyl magnesium bromide. Yield: 70%. Appearance: white solid. ^1^H NMR (CDCl_3_, 400 MHz): δ 7.24–7.20 (m, 4H), 7.12 (m, 1H), 4.32 (d, *J* = 7.6 Hz, 1H), 4.04–3.88 (m, 4H), 2.47–2.29 (ovl, 6H), 2.19–2.06 (ovl, 2H), 1.94–1.76 (ovl, 4H), 1.70–1.52 (ovl, 5H), 1.30 (m, 1H), 0.55 (s, 3H). HR ESIMS *m*/*z*: 391.2274 [(M + H) – H_2_O]^+^, C_26_H_33_O_4_–H_2_O requires 391.2268.

#### 3,3-Ethylenedioxy-5α-hydroxy-11β-(4-methoxybenzyl)-9-en-17-one (**6**)

Grignard reagent: 4-methoxybenzyl magnesium bromide. Yield: 61%. Appearance: white solid. ^1^H NMR (CDCl_3_, 400 MHz): δ 6.95 (d, *J* = 8.0 Hz, 2H), 6.77 (d, *J* = 8.0 Hz, 2H), 3.97–3.81 (m, 4H), 3.74 (s, 3H), 3.19 (q, *J* = 7.7 Hz, 1H), 2.65 (m, 2H), 2.48–2.31 (ovl, 3H), 2.15 (m, 1H), 1.97 (ovl, 2H), 1.85–1.73 (ovl, 4H), 1.60 (m, 1H), 1.52–1.34 (ovl, 6H), 1.08 (s, 3H), 0.57 (m, 1H). HR ESIMS *m*/*z*: 435.2541 [(M + H) – H_2_O]^+^, C_28_H_37_O_5_–H_2_O requires 435.2530.

### General Procedure for C5 Elimination and Acetal Deprotection

Acetic anhydride (6 equiv) and DMAP (0.2 equiv) were added to a solution of **3**, **4**, **5**, or **6** in pyridine, alternatively. The mixture was heated at 65 °C for 24 h. Solvent was evaporated, and the crude was extracted with DCM and HCl 1 N (3×). The crude products were purified by flash chromatography in a mixture of hexanes/ethyl acetate (6:4) to afford the final compounds then purified by HPLC.

#### 11β-Biphenyl-5α-hydroxyestra-9-en-17-one (**1a**)

Purification by HPLC on a Nucleodur Sphinx C18 5 μm (4.6 mm inner diameter x 250 mm) with ACN/H_2_O (80:20) as eluent (flow rate 3 mL/min) (*R*_*t*_ = 13 min, 72% yield). Appearance: white solid. ^1^H NMR (CDCl_3_, 400 MHz): δ 7.57 (d, *J* = 8.6 Hz, 2H), 7.52 (d, *J* = 8.6 Hz, 2H), 7.42 (t, *J* = 7.7 Hz, 2H), 7.33 (t, *J* = 7.4 Hz, 1H), 7.25 (d, *J* = 7.7 Hz, 2H), 5.85 (s, 1H), 4.47 (d, *J* = 7.4 Hz, 1H), 2.77 (dt, *J* = 14.4, 4.6 Hz, 1H), 2.66 (m, 3H), 2.60 (m, 1H), 2.52–2.31 (ovl, 4H), 2.21–2.02 (ovl, 3H), 1.96 (ovl, 1H), 1.61 (m, 3H), 0.60 (s, 3H). ^13^C NMR (CDCl_3_, 100 MHz): δ 218.9, 200.2, 157.0, 145.3, 143.1, 140.6, 139.1, 130.3, 128.9 (2C), 127.5 (4C), 127.4 (1C), 127.0 (2C), 123.4, 50.8, 47.9, 40.2, 38.2, 37.9, 36.8, 35.6, 31.1, 26.9, 26.0, 22.0, 14.7. HR ESIMS *m*/*z*: 422.2508 [M + H]^+^, C_30_H_30_O_2_ requires 422.2246.

#### 11β-(4-*N*,*N*-Dimethylaniline)-5α-hydroxyestra-9-en-17-one (**1b**)

The crude was purified by preparative HPLC employing an Agilent Pre-C18 column (30 × 100 mm, 5 μM) with solvents A (water 0.1% TFA) and B (ACN 0.1% TFA). The method was as follows: 30% B from 0 to 3 min, 30% B to 50% B in 10 min, 50% B to 90%B in 5 min (*R*_*t*_ = 11.6 min, 48% yield). ^1^H NMR (CDCl_3_, 400 MHz): δ 7.22 (d, *J* = 8.5 Hz, 2H), 7.16 (d, *J* = 8.5 Hz, 2H), 5.81 (s, 1H), 4.41 (d, *J* = 7.2 Hz, 1H), 3.06 (s, 6H), 2.73 (dt, *J* = 14.6, 5.0 Hz, 1H), 2.62 (m, 3H), 2.51–2.40 (ovl, 4H), 2.38–2.35 (m, 1H), 2.16–2.08 (ovl, 2H), 2.03 (m, 1H), 1.95 (m, 1H), 1.59 (m, 3H), 0.54 (s, 3H). ^13^C NMR (CDCl_3_, 100 MHz): δ 218.9, 199.3, 155.9, 144.7, 144.2, 130.6, 128.5 (2C), 123.8 (2C), 117.7, 50.7, 47.7, 43.9 (2C), 39.8 (2C), 38.0, 37.8, 36.9, 35.5, 31.0, 26.9, 26.1, 22.0, 14.7. HR ESIMS *m*/*z*: 390.2658. [M + H]^+^, C_26_H_31_NO_2_ requires 389.2355.

#### 11β-Phenylestra-4,9-dien-3,17-dione (**1c**)

Purification by HPLC on Luna C18 column (10 × 250 mm, 5 μM) with ACN/H_2_O (80:20) and 0.1% TFA as eluent (flow rate 3 mL/min) (*R*_*t*_ = 9.7 min, 45% yield). Appearance: white solid. ^1^H NMR (CDCl_3_, 400 MHz): δ 7.31–7.26 (m, 2H), 7.22–7.14 (m, 3H), 5.80 (s, 1H), 4.44 (d, *J* = 7.6 Hz, 1H), 2.75 (dt, *J* = 14.8, 4.5 Hz, 1H), 2.64 (m, 3H), 2.55 (m, 1H), 2.51–2.28 (ovl, 4H), 2.19–2.08 (m, 2H), 2.03–1.92 (m, 2H), 1.58 (ovl, 3H), 0.55 (s, 3H). ^13^C NMR (CDCl_3_, 100 MHz): δ 219.0, 199.4, 156.0, 144.7, 144.2, 130.4, 128.9 (2C), 127.1 (2C), 126.2, 123.6, 50.8, 47.8, 40.4, 38.2, 38.0, 37.0, 35.5, 31.0, 26.9, 26.1, 22.0, 14.5. HR ESIMS *m*/*z*: 347.2002 [M + H]^+^, C_24_H_26_O_2_ requires 346.1933.

#### 11β-(4-Methoxybenzyl)­estra-4,9-dien-3,17-dione (**1d**)

Purification by HPLC on Luna C18 column (10 mm × 250 mm, 5 μM) with ACN/H_2_O (75:25) and 0.1% TFA as eluent (flow rate 3 mL/min) (*R*_*t*_ = 11.4 min, 47% yield). Appearance: white solid. ^1^H NMR (CDCl_3_, 400 MHz): δ 7.00 (d, *J* = 8.6 Hz, 2H), 6.80 (d, *J* = 8.6 Hz, 2H), 5.68 (s, 1H), 3.77 (s, 3H), 3.29 (q, *J* = 7.6 Hz, 1H), 2.74 (d, *J* = 7.8 Hz, 2H), 2.66 (m, 1H), 2.60–2.36 (ovl, 4H), 2.33–2.19 (ovl, 2H), 2.15–2.05 (ovl, 2H), 2.02–1.94 (ovl, 2H), 1.82 (m, 1H), 1.60–1.39 (ovl, 4H), 1.18 (s, 3H). ^13^C NMR (CDCl_3_, 100 MHz) δ: 218.5, 199.8, 158.4, 156.8, 147.1, 132.2, 130.2 (2C), 128.0, 122.9, 114.0 (2C), 55.4, 51.7, 47.3, 42.5, 40.1, 37.2, 35.7, 35.4, 35.1, 30.7, 26.4, 26.0, 21.7, 16.2. HR ESIMS *m*/*z*: 391.3124 [M + H]^+^, C_26_H_30_O_3_ requires 390.2214.

#### Synthesis of 11β-(4-Hydroxybenzyl)­estra-4,9-dien-3,17-dione (**1e**)

In a solution of **23** in *dry* DCM at −20 °C was added BBr_3_ (3 equiv) dropwise. The reaction is left to stir at −20 °C overnight. The TLC showed complete conversion of the starting material to the product. The reaction was extracted with DCM (3×), and the organic layers were washed with brine, dried over Na_2_SO_4_, filtered, and concentrated in vacuo. The product was then purified by HPLC. Purification by HPLC on Luna C18 column (10 × 250 mm, 5 μM) with ACN/H_2_O (70:30) and 0.1% TFA as eluent (flow rate 3 mL/min) (*R*_*t*_ = 7.72 min, 44% yield). Appearance: white solid. ^1^H NMR (CDCl_3_, 400 MHz) δ 6.94 (d, *J* = 8.6 Hz, 2H), 6.72 (d, *J* = 9.0 Hz, 2H), 5.69 (s, 1H), 3.28 (q, *J* = 7.6 Hz, 1H), 2.74 (d, *J* = 7.5 Hz, 2H), 2.66 (m, 1H), 2.57–2.36 (ovl, 4H), 2.33–2.06 (ovl, 6H), 1.83–1.39 (ovl, 6H), 1.18 (s, 3H). ^13^C NMR (CDCl_3_, 400 MHz): δ 220.3, 198.6, 156.5, 156.2, 147.8, 136.8, 131.6 (2C), 130.3, 122.2, 116.3 (2C), 51.0, 48.4, 39.0, 38.0, 37.8, 36.8, 36.5, 34.6, 30.5, 29.5, 24.2, 22.5, 17.6. HR ESIMS *m*/*z*: 377.2234 [M + H]^+^, C_25_H_28_O_3_ requires 376.2038.

#### Grignard Reaction to Afford 11β-(4-Iodophenyl)-3,3-ethylenedioxyestra-4,9-dien-3,17-dione (**7**)

A solution of 1,4-diiodobenzene (2.2 equiv) in anhydrous THF (0.8 M) was cooled to −10 °C as a 2 M solution of isopropyl magnesium chloride (2.2 equiv) was added dropwise over a period of 15 min. After stirring for 20 min, cuprous chloride (0.9 equiv) was added as a solid, and the reaction mixture was stirred for 30 min. A solution of the epoxide 2 in THF (0.6 M) was added dropwise and stirred for 12 h, slowly warming to 10 °C. The reaction was quenched with saturated aqueous ammonium chloride solution and was extracted with ethyl acetate (3×). The combined organic layer was washed further with water and brine, dried over sodium sulfate, and evaporated in vacuo to afford the crude product. The crude product was purified by flash chromatography in a mixture of hexanes/ethyl acetate (6:4) to afford compound **7** (52%). Appearance: off-white solid. ^1^H NMR (CDCl_3_, 400 MHz): δ 7.57 (d, *J* = 8.5 Hz, 2H), 7.98 (d, *J* = 8.5 Hz, 2H), 4.26 (d, *J* = 7.4 Hz, 1H), 4.06–3.85 (m, 4H), 2.47–2.28 (ovl, 5H), 2.11–1.99 (ovl, 5H), 1.91–1.81 (m, 1H), 1.76 (d, *J* = 12.9 Hz, 1H), 1.67–1.61 (ovl, 2H), 1.53 (ovl, 3H), 1.20 (m, 1H), 0.49 (s, 3H). HR ESIMS *m*/*z*: 517.1246 [(M + H) – H_2_O]^+^, C_26_H_32_IO_4_–H_2_O requires 517.1234.

#### Synthesis of 11β-(4-Iodophenyl)­estra-4,9-dien-3,17-dione (**1f**)

Compound **1f** was synthesized according to a procedure similar to that for compounds **1a–1d**. Purification by HPLC on a Luna C18 column (10 × 250 mm, 5 μM) with solvent A (water 0.1% TFA) and B (ACN 0.1% TFA). The method was as follows: 65% B from 0 to 2 min, 75% B to 85% B in 20 min (*R*_*t*_ = 17.6 min, 70% yield). Appearance: white solid. ^1^H NMR (CDCl_3_, 400 MHz): δ 7.60 (d, *J* = 8.5 Hz, 2H), 6.95 (d, *J* = 8.5 Hz, 2H), 5.81 (s, 1H), 4.37 (d, *J* = 7.3 Hz, 1H), 2.72 (dt, *J* = 14.7, 5.3 Hz, 1H), 2.62 (m, 3H), 2.50 (ovl, 4H), 2.28 (m, 1H), 2.17–2.10 (ovl, 2H), 2.03 (m, 1H), 1.95 (m, 1H), 1.58 (ovl, 3H), 0.55 (s, 3H). ^13^C NMR (CDCl_3_, 100 MHz): δ 219.0, 199.5, 156.0, 144.0, 144.0, 137.9 (2C), 130.7, 129.2 (2C), 123.8, 91.4, 50.6, 47.8, 40.1, 38.1, 37.8, 36.8, 35.5, 31.0, 26.8, 26.0, 22.0, 14.7. HR ESIMS *m*/*z*: 473.0679 [M + H]^+^, C_24_H_25_IO_2_ requires 472.0899.

### General Procedure of Suzuki Coupling

Compound **1f**, boronic acid (2 equiv), Pd­(PPh_3_)_4,_ and potassium carbonate (2 equiv) were introduced in a flask fitted with a condenser, and the system was connected to a nitrogen-vacuum inlet; toluene and methanol (1:1) were added, and the flask was evacuated and backfilled with nitrogen 7–10 times. The flask was immersed in a preheated oil bath at 100 °C and refluxed overnight. The TLC showed complete conversion of the starting material to the product. The reaction was cooled in an ice bath, and water was added; the reaction was extracted with ethyl acetate, and the organic layer was washed with water and brine and dried over sodium sulfate. The crude material was purified by HPLC.

#### 11β-(4-Cyclopropylphenyl)-estra-4,9-dien-3,17-dione (**1g**)

Boronic acid: cyclopropylboronic acid. The crude was purified by preparative HPLC employing an Agilent Pre-C18 column (30 mm × 100 mm, 5 μM) with solvent A (water 0.1% FA) and B (ACN 0.1% FA). The method was as follows: 60% B from 0 to 2 min, 60% B to 80% B in 15 min (*R*_*t*_ = 10.05 min, 52% yield). Appearance: white solid. ^1^H NMR (CDCl_3_, 400 MHz): δ 7.05 (d, *J* = 8.6 Hz, 2H), 6.97 (d, *J* = 8.6 Hz, 2H), 5.80 (s, 1H), 4.39 (d, *J* = 7.1 Hz, 1H), 2.73 (dt, *J* = 14.6, 4.7 Hz, 1H), 2.66–2.61 (m, 3H), 2.52 (m, 1H) 2.45–2.26 (ovl, 4H), 2.18–2.00 (ovl, 3H), 1.91 (m, 1H), 1.84 (m, 1H), 1.58 (m, 3H), 1.00–0.88 (m, 2H), 0.71–0.59 (m, 2H), 0.56 (s, 3H). ^13^C NMR (CDCl_3_, 100 MHz): δ 219.3, 200.0, 156.7, 145.4, 141.9, 140.9, 130.1, 126.9 (2C), 126.0 (2C), 123.4, 50.7, 47.9, 40.1, 38.1, 37.8, 36.8, 35.6, 31.0, 26.8, 26.0, 22.0, 15.1, 14.6, 9.5, 9.4. HR ESIMS *m*/*z*: 387.4596 [M + H] ^+^, and C_27_H_30_O_2_ requires 386.2246.

#### 11β-(2,4,6-Trimethylphenyl)-estra-4,9-dien-3,17-dione (**1h**)

Boronic acid: 2,4,6-trimethylphenylboronic acid. The crude was purified by preparative HPLC employing an Agilent Pre-C18 column (30 × 100 mm, 5 μM) with solvent A (water 0.1% TFA) and B (ACN 0.1% TFA). The method was as follows: 60% B from 0 to 2 min, 60% B to 80% B in 15 min, 80% B to 98% in 1 min, 98% B from 18 to 22 min B (*R*_*t*_ = 20.0 min, 42% yield). Appearance: white solid. ^1^H NMR (CDCl_3_, 400 MHz): δ 7.22 (d, *J* = 8.3 Hz, 2H), 7.05 (d, *J* = 8.3 Hz, 2H), 6.93 (s, 2H), 5.87 (s, 1H), 4.50 (d, *J* = 6.8 Hz, 1H), 2.83 (dt, *J* = 14.0, 4.8 Hz, 1H), 2.60 (ovl, 2H), 2.54–2.38 (ovl, 4H), 2.32 (s, 3H), 2.18 (m, 2H), 2.05 (m, 1H), 1.98 (m, 1H), 1.95 (s, 6H), 1.62 (m, 3H), 1.26 (m, 1H), 0.60 (s, 3H). ^13^C NMR (CDCl_3_, 100 MHz): δ 219.5, 201.4, 158.3, 146.5, 142.1, 139.1, 138.6, 136.8, 136.1, 130.1, 129.8, 128.2 (2C), 127.1 (2C), 123.0, 50.8, 47.9, 40.4, 38.3, 38.0, 36.5, 35.6, 31.1, 29.9, 26.8, 25.9, 22.0, 21.2, 20.8 (3C), 14.3. HR ESIMS *m*/*z*: 465.3649 [M + H]^+^, C_33_H_36_O_2_ requires 464.2715.

#### 11β-(4-Methoxyphenyl)-estra-4,9-dien-3,17-dione (**2a**)

Boronic acid: 4-methoxyphenylboronic acid. The crude was purified by preparative HPLC employing an Agilent Pre-C18 column (30 × 100 mm, 5 μM) with solvent A (water 0.1% TFA) and B (ACN 0.1% TFA). The method was as follows: 60% B from 0 to 2 min, 60% B to 80% B in 15 min (*R*_*t*_ = 11.05 min, 90% yield). Appearance: white solid. ^1^H NMR (CDCl_3_, 400 MHz): δ 7.57–7.41 (m, 4H), 7.23 (d, *J* = 8.3 Hz, 2H), 6.93 (d, *J* = 8.7 Hz, 2H), 5.83 (s, 1H), 4.47 (d, *J* = 7.1 Hz, 1H), 3.85 (s, 3H), 2.77 (dt, *J* = 14.5, 5.1 Hz, 1H), 2.67 (m, 3H), 2.59 (m, 1H), 2.52–2.31 (ovl, 4H), 2.21–2.09 (ovl, 2H), 2.04 (m, 1H), 1.98 (m, 1H), 1.59 (m, 3H), 0.60 (s, 3H). ^13^C NMR (CDCl_3_, 100 MHz): δ 219.0, 199.5, 159.3, 156.1, 144.8, 142.5, 138.6, 133.1, 130.4, 128.0 (2C), 127.4 (2C), 127.0 (2C), 123.6, 114.4 (2C), 55.5, 50.8, 47.9, 40.2, 38.2, 37.8, 37.0, 35.6, 31.0, 26.9, 26.1, 22.0, 14.7. HR ESIMS *m*/*z*: 453.2659 [M + H]^+^, C_31_H_32_O_3_ requires 452.2351.

#### 11β-(3-Fluoro-4-methoxyphenyl)-estra-4,9-dien-3,17-dione (**2b**)

Boronic acid: 3-fluoro-4-methoxyphenylboronic acid. The crude was purified by HPLC employing a Luna C18 column (10 × 250 mm, 5 μM) with solvent A (water 0.1% TFA) and B (ACN 0.1% TFA). The method was as follows: 75% B from 0 to 2 min, 75% B to 80% B in 18 min (*R*_*t*_ = 13.9 min, 79% yield). Appearance: white solid. ^1^H NMR (CDCl_3_, 400 MHz): δ 7.45 (d, *J* = 8.1 Hz, 2H), 7.35–7.27 (m, 2H), 7.24 (d, *J* = 8.1 Hz, 2H), 7.01 (t, *J* = 8.8 Hz, 1H), 5.83 (s, 1H), 4.47 (d, *J* = 7.2 Hz, 1H), 3.93 (s, 3H), 2.77 (dt, *J* = 14.8, 5.2 Hz, 1H), 2.67 (m, 3H), 2.58 (m, 1H), 2.52–2.30 (ovl, 4H), 2.16 (m, 2H), 2.02 (ovl, 2H), 1.60 (m, 3H), 0.59 (s, 3H). ^13^C NMR (CDCl_3_, 100 MHz): δ 219.0, 199.4, 156.0, 150.9 (d, *J* = 686.7 Hz), 149.4 (d, *J* = 430.8 Hz), 144.6, 143.2, 137.6 (d, *J* = 1.7 Hz), 133.8, 130.5, 127.6 (2C), 127.0 (2C), 123.7, 122.5 (d, *J* = 3.1 Hz), 114.7 (d, *J* = 2.2 Hz), 113.8 (d, *J* = 2.2 Hz), 56.5, 50.8, 47.9, 40.2, 38.2, 37.9, 36.9, 35.6, 31.0, 27.0, 26.1, 22.0, 14.7. ^19^F NMR (CDCl_3_, 376 MHz): δ −115.44. HR ESIMS *m*/*z*: 471.3659 [M + H]^+^, C_31_H_31_FO_3_ requires 470.2257.

#### 11β-(2-Fluoro-4-methoxyphenyl)-estra-4,9-dien-3,17-dione (**2c**)

Boronic acid: 2-fluoro-4-methoxyphenylboronic acid. Purification by HPLC on a Luna C18 column (10 × 250 mm, 5 μM) with solvent A (water 0.1% TFA) and B (ACN 0.1% TFA). The method was as follows: 75% B from 0 to 2 min, 75% B to 85% B in 15 min (*R*_*t*_ = 14.37 min, 36% yield). ^1^H NMR (CDCl_3_, 400 MHz): δ 7.47–7.41 (m, 2H), 7.34 (t, *J* = 8.8 Hz, 1H), 7.23 (d, *J* = 8.2 Hz, 2H), 6.77 (dd, *J* = 12.6, 2.5 Hz, 1H), 6.70 (dd, *J* = 12.6, 2.5 Hz, 1H), 5.85 (s, 1H), 4.47 (d, *J* = 7.1 Hz, 1H), 3.83 (s, 3H), 2.78 (dt, *J* = 14.3, 4.9 Hz, 1H), 2.67 (ovl, 3H), 2.60 (m, 1H), 2.53–2.39 (ovl, 4H), 2.16 (m, 2H), 2.04 (m, 1H), 1.97 (m, 1H), 1.60 (m, 3H), 0.61 (s, 3H). ^13^C NMR (CDCl_3_, 100 MHz): δ 219.07, 199.52, 160.99 (d, *J* = 141.9 Hz) 159.82 (d, *J* = 117.2 Hz), 156.13, 144.71, 142.94, 133.66, 130.96 (d, *J* = 5.4 Hz), 130.41 (2C), 129.13 (d, *J* = 2.7 Hz), 127.12 (2C), 123.64, 120.83 (d, *J* = 13.7 Hz), 110.48 (d, *J* = 3.0 Hz), 102.22 (d, *J* = 26.6 Hz), 55.77, 50.81, 47.90, 40.22, 38.11, 37.80, 36.95, 35.56, 31.00, 26.90, 26.11, 22.00, 14.70. ^19^F NMR (CDCl_3_, 376 MHz): δ −115.44. HR ESIMS *m*/*z*: 471.2654 [M + H]^+^, C_31_H_31_FO_3_ requires 470.2257.

#### 11β-(4-Bromopyridin-3-yl)­phenyl-estra-4,9-dien-3,17-dione (**2d**)

Boronic acid: (4-bromopyridin-3-yl)­boronic acid. The crude was purified by HPLC employing a Luna C18 column (10 × 250 mm, 5 μM) with solvent A (water 0.1% TFA) and B (ACN 0.1% TFA). The method was as follows: 75% B from 0 to 2 min, 75% B to 85% B in 18 min (*R*_*t*_ = 12.6 min, 20% yield). Appearance: white solid. ^1^H NMR (CDCl_3_, 400 MHz): δ 8.62 (s, 1H), 7.75 (dd, *J* = 8.3, 2.4 Hz, 1H), 7.57 (d, *J* = 8.3 Hz, 1H), 7.49 (d, *J* = 8.0 Hz, 2H), 7.32 (d, *J* = 8.2 Hz, 2H), 5.85 (s, 1H), 4.48 (d, *J* = 7.2 Hz, 1H), 2.77 (dt, *J* = 14.7, 5.5 Hz, 1H), 2.67 (m, 3H), 2.57 (m, 1H), 2.53–2.36 (ovl, 3H), 2.22–2.15 (ovl, 3H), 2.12–1.98 (ovl, 2H), 1.62 (m, 3H), 0.59 (s, 3H). ^13^C NMR (CDCl_3_, 100 MHz): δ 218.9, 199.4, 155.9, 148.4, 144.9, 144.1, 141.0, 136.9, 135.5, 134.4, 130.6, 128.2, 128.0 (2C), 127.4 (2C), 123.8, 50.7, 47.9, 40.2, 38.2, 37.9, 36.9, 35.5, 31.0, 26.9, 26.1, 22.0, 14.7. HR ESIMS *m*/*z*: 502.4567 [M + H]^+^, C_29_H_28_BrNO_2_ requires 501.1303.

#### 11β-(3,5-Dimethoxyphenyl)-estra-4,9-dien-3,17-dione (**2e**)

Boronic acid: 3,5-dimethoxyphenylboronic acid. The crude was purified by HPLC employing a Luna C18 column (10 × 250 mm, 5 μM) with solvent A (water 0.1% TFA) and B (ACN 0.1% TFA). The method was as follows: 75% B from 0 to 2 min, 75% B to 80% B in 18 min (*R*_*t*_ = 14.3 min, 51% yield). Appearance: white solid. ^1^H NMR (CDCl_3_, 400 MHz): δ 7.50 (d, *J* = 8.7 Hz, 2H), 7.25 (d, *J* = 9.2 Hz, 2H), 6.72 (s, 1H), 6.71 (s, 1H), 6.45 (t, *J* = 2.3 Hz, 1H), 5.83 (s, 1H), 4.47 (d, *J* = 7.1 Hz, 1H), 3.84 (s, 6H), 2.77 (dt, *J* = 14.2, 5.4 Hz, 1H), 2.67 (m, 3H), 2.59 (m, 1H), 2.52–2.31 (ovl, 4H), 2.21–2.09 (ovl, 2H), 2.04 (m, 1H), 1.99 (m, 1H), 1.61 (ovl, 3H), 0.60 (s, 3H). ^13^C NMR (CDCl_3_, 100 MHz): δ 218.7, 199.4, 161.2 (2C), 156.0, 144.6, 143.6, 142.8, 139.0, 130.4, 127.5, 127.4, 123.7, 105.4 (2C), 99.4, 55.6, 55.6, 50.8, 47.9, 40.2, 38.2, 37.9, 37.0, 35.6, 31.0, 29.8, 29.8, 26.9, 26.1, 22.0, 14.7. HR ESIMS *m*/*z*: 483.4596 [M + H]^+^, C_32_H_34_O_4_ requires 482.2457.

#### 11β-(3-Cyanophenyl)-estra-4,9-dien-3,17-dione (**2f**)

Boronic acid: 3-cyanophenylboronic acid. The crude was purified by preparative HPLC employing an Agilent Pre-C18 column (30 × 100 mm, 5 μM) with solvent A (water 0.1% FA) and B (MeOH 0.1% FA). The method was as follows: 65% B from 0 to 1 min, 65% B to 85% B in 15 min (*R*_*t*_ = 8.3 min, 46% yield). Appearance: white solid. ^1^H NMR (CDCl_3_, 400 MHz): δ 7.85 (t, *J* = 1.7 Hz, 1H), 7.79 (dt, *J* = 7.7, 1.4 Hz, 1H), 7.62 (dt, *J* = 7.7, 1.4 Hz, 1H), 7.54 (t, *J* = 7.8 Hz, 1H), 7.50 (d, *J* = 8.4 Hz, 2H), 7.32 (d, *J* = 8.4 Hz, 2H), 5.83 (s, 1H), 4.49 (d, *J* = 7.2 Hz, 1H), 2.77 (dt, *J* = 14.7, 5.7 Hz, 1H), 2.67 (m, 3H), 2.58 (m, 1H), 2.53–2.29 (ovl, 4H), 2.22–1.98 (ovl, 4H), 1.60 (ovl, 3H), 0.59 (s, 3H). ^13^C NMR (CDCl_3_, 100 MHz): δ 218.8, 199.3, 155.8, 144.7, 144.2, 141.9, 136.8, 131.4, 130.8, 130.6, 130.7, 129.8, 127.9 (2C), 127.5 (2C), 123.8, 119.0, 113.2, 50.7, 47.9, 40.2, 38.2, 37.9, 36.9, 35.5, 31.0, 26.9, 26.1, 22.0, 14.7. HR ESIMS *m*/*z*: 448.2654 [M + H]^+^, C_31_H_29_NO_2_ requires 447.2198.

#### 11β-(3-Acetylphenyl)-estra-4,9-dien-3,17-dione (**2g**)

Boronic acid: 3-acetylphenylboronic acid. The crude was purified by preparative HPLC employing an Agilent Pre-C18 column (30 × 100 mm, 5 μM) with solvent A (water 0.1% FA) and B (ACN 0.1% FA). The method was as follows: 60% B from 0 to 2 min, 60% B to 80% B in 15 min (*R*_*t*_ = 8.2 min, 52% yield). Appearance: white solid. ^1^H NMR (CDCl_3_, 400 MHz): δ 8.16 (t, *J* = 1.8 Hz, 1H), 7.92 (dt, *J* = 7.9, 1.3 Hz, 1H), 7.77 (dt, *J* = 7.9, 1.3 Hz, 1H), 7.60–7.48 (ovl, 3H), 7.29 (d, *J* = 8.1 Hz, 2H), 5.83 (s, 1H), 4.49 (d, *J* = 7.2 Hz, 1H), 2.78 (dt, *J* = 14.1, 5.05 Hz, 1H), 2.67 (ovl, 3H), 2.65 (s, 3H), 2.60 (m, 1H), 2.53–2.31 (ovl, 4H), 2.22–2.09 (ovl, 2H), 2.07–1.97 (ovl, 2H), 1.62 (m, 3H), 0.60 (s, 3H). ^13^C NMR (CDCl_3_, 100 MHz): δ 218.9, 199.4, 198.2, 156.0, 144.5, 144.0, 141.1, 138.1, 137.8, 131.6, 130.5, 129.2, 127.7 (2C), 127.6 (2C), 127.4, 126.8, 123.7, 50.8, 47.9, 40.2, 38.2, 37.9, 37.0, 35.6, 31.0, 26.9, 26.9, 26.1, 22.0, 14.7. HR ESIMS *m*/*z*: 465.3568 [M + H]^+^, C_32_H_32_O_3_ requires 464.2351.

#### 11β-(4-Cyanophenyl)-estra-4,9-dien-3,17-dione (**2h**)

Boronic acid: 4-cyanophenylboronic acid. The crude was purified by preparative HPLC employing an Agilent Pre-C18 column (30 × 100 mm, 5 μM) with solvent A (water 0.1% FA) and B (ACN 0.1% FA). The method was as follows: 60% B from 0 to 2 min, 60% B to 80% B in 15 min (*R*_*t*_ = 8.7 min, 45% yield). Appearance: white solid. ^1^H NMR (CDCl_3_, 400 MHz): δ 7.71 (d, *J* = 8.0 Hz, 2H), 7.66 (d, *J* = 8.1 Hz, 2H), 7.53 (d, *J* = 7.8 Hz, 2H), 7.30 (d, *J* = 7.7 Hz, 2H), 5.82 (s, 1H), 4.49 (d, J = 7.2, 1H), 2.76 (dt, *J* = 15.1, 5.10 Hz, 1H), 2.66 (m, 3H), 2.58 (m, 1H), 2.51–2.41 (ovl, 2H), 2.38–2.29 (ovl, 2H), 2.20–2.11 (ovl, 2H), 2.05 (m, 1H), 2.00 (m, 1H), 1.62–1.54 (ovl, 3H), 0.58 (s, 3H). ^13^C NMR (CDCl_3_, 100 MHz): δ 218.9, 199.3, 155.8, 145.1, 145.0, 144.2, 137.0, 132.8 (2C), 130.6, 127.9 (2C), 127.6 (2C), 127.6 (2C), 123.8, 119.1, 111.0, 50.7, 47.8, 40.2, 38.2, 37.9, 36.9, 35.5, 31.0, 26.9, 26.1, 22.0, 14.7. HR ESIMS *m*/*z*: 448.2264. [M + H]^+^, C_31_H_29_NO_2_ requires 447.2198.

#### 11β-(2-Cyanophenyl)-estra-4,9-dien-3,17-dione (**2i**)

Boronic acid: 2-cyanophenylboronic acid. The crude was purified by preparative HPLC employing an Agilent Pre-C18 column (30 × 100 mm, 5 μM) with solvent A (water 0.1% FA) and B (ACN 0.1% FA). The method was as follows: 60% B from 0 to 2 min, 60% B to 80% B in 15 min (*R*_*t*_ = 8.0 min, 53% yield). Appearance: white solid. ^1^H NMR (CDCl_3_, 400 MHz): δ 7.75 (dd, *J* = 7.8, 1.4 Hz, 1H), 7.64 (td, *J* = 7.7, 1.4 Hz, 1H), 7.54–7.49 (ovl, 3H), 7.43 (td, *J* = 7.6, 1.2 Hz, 1H), 7.32 (d, *J* = 7.7 Hz, 2H), 5.82 (s, 1H), 4.50 (d, *J* = 7.2 Hz, 1H), 2.78 (dt, *J* = 15.2, 5.6 Hz, 1H), 2.69–2.64 (ovl, 3H), 2.60 (m, 1H), 2.51–2.33 (ovl, 4H), 2.20–2.10 (m, 2H), 2.05–1.97 (ovl, 2H), 1.62–1.54 (ovl, 3H), 0.59 (s, 3H). ^13^C NMR (CDCl_3_, 100 MHz): δ 218.7, 199.4, 156.0, 145.0, 144.9, 144.2, 136.1, 134.0, 133.0, 130.6, 130.1, 129.2 (2C), 127.7, 127.4 (2C), 123.8, 118.9, 111.3, 50.8, 47.9, 40.2, 38.0, 37.9, 36.9, 35.5, 31.0, 26.9, 26.1, 22.0, 14.7. HR ESIMS *m*/*z*: 448.5686. [M + H]^+^, C_31_H_29_NO_2_ requires 447.2198.

#### 11β-(3-Cyano-4-methoxyphenyl)-estra-4,9-dien-3,17-dione (**2j**)

Boronic acid: 3-cyano-4-methoxyphenylboronic acid. Purification by HPLC on a Luna C18 column (10 × 250 mm, 5 μM) with solvent A (water 0.1% TFA) and B (ACN 0.1% TFA). The method was as follows: 75% B from 0 to 2 min, 75% B to 80% B in 15 min (*R*_*t*_ = 12.6 min, 58% yield). Appearance: white solid. ^1^H NMR (CDCl_3_, 400 MHz): δ 7.72–7.64 (ovl, 2H), 7.37 (d, *J* = 7.4 Hz, 2H), 7.20 (d, *J* = 7.2 Hz, 2H), 6.97 (d, *J* = 8.7 Hz, 1H), 5.75 (s, 1H), 4.46–4.34 (d, *J* = 7.0 Hz, 1H), 3.90 (s, 3H), 2.70 (dt, *J* = 14.3, 5.11 Hz, 1H), 2.60 (ovl, 3H), 2.51 (d, *J* = 13.7 Hz, 1H), 2.44–2.25 (ovl, 4H), 2.13–2.04 (ovl, 2H), 1.92 (m, 1H), 1.56–1.48 (ovl, 4H), 0.52 (s, 3H). ^13^C NMR (CDCl_3_, 100 MHz): δ 218.9, 199.4, 160.7, 155.9, 144.4, 143.8, 136.5, 133.7, 132.9, 132.1, 130.5, 127.8 (2C), 127.0 (2C), 123.8, 111.9, 102.4, 56.4, 50.7, 47.9, 40.2, 38.2, 37.9, 36.9, 35.5, 31.0, 26.9, 26.1, 26.0, 22.0, 14.7. HR ESIMS *m*/*z*: 478.5686. [M + H]^+^, C_32_H_31_NO_3_ requires 477.2304.

#### 11β-(3-Cyano-5-methoxyphenyl)-estra-4,9-dien-3,17-dione (**2k**)

Boronic acid: (3-cyano-5-methoxyphenyl)­boronic acid. Purification by HPLC on a Luna C18 column (10 × 250 mm, 5 μM) with solvent A (water 0.1% TFA) and B (ACN 0.1% TFA). The method was as follows: 75% B from 0 to 2 min, 75% B to 80% B in 15 min (*R*_*t*_ = 12.5 min, 47% yield). Appearance: white solid. ^1^H NMR (CDCl_3_, 400 MHz): δ 7.48 (d, *J* = 8.1 Hz, 2H), 7.44 (t, *J* = 1.4 Hz, 1H), 7.32–7.27 (ovl, 3H), 7.10 (dd, *J* = 2.5, 1.3 Hz, 1H), 5.82 (s, 1H), 4.48 (d, *J* = 7.2 Hz, 1H), 3.88 (s, 3H), 2.76 (m, 1H), 2.67 (ovl, 3H), 2.58 (d, *J* = 13.6 Hz, 1H), 2.51–2.30 (ovl, 4H), 2.20–2.11 (ovl, 2H), 2.04 (m, 1H), 2.00 (m, 1H), 1.61 (overlapped with H_2_O, 3H), 0.58 (s, 3H). ^13^C NMR (CDCl_3_, 100 MHz): δ 218.9, 199.32, 160.21, 155.86, 144.81, 144.20, 143.37, 136.76, 130.61, 127.83 (2C), 127.50 (2C), 123.8, 123.3, 118.9, 118.0, 115.4, 113.7, 55.8, 50.7, 47.8, 40.2, 38.2, 37.9, 36.9, 35.5, 31.0, 26.9, 26.1, 22.0, 14.7. HR ESIMS *m*/*z*: 478.6854. [M + H]^+^, C_32_H_31_NO_3_ requires 477.2304.

#### 11β-(5-Cyano-2-methoxyphenyl)-estra-4,9-dien-3,17-dione (**2l**)

Boronic acid: (5-cyano-2-methoxyphenyl)­boronic acid. The crude was purified by preparative HPLC employing an Agilent Pre-C18 column (30 × 100 mm, 5 μM) with solvent A (water 0.1% FA) and B (ACN 0.1% FA). The method was as follows: 60% B from 0 to 2 min, 60% B to 80% B in 18 min (*R*_*t*_ = 7.9 min, 39% yield). Appearance: white solid. ^1^H NMR (CDCl_3_, 400 MHz): δ 7.64–7.55 (ovl, 2H), 7.43–7.40 (d, *J* = 8.2 Hz, 2H), 7.26 (d, *J* = 8.2 Hz, 2H), 7.01 (d, *J* = 8.5 Hz, 1H), 5.82 (s, 1H), 4.48 (d, *J* = 7.4 Hz, 1H), 3.88 (s, 3H), 2.77 (dt, *J* = 15.1, 6.0 Hz, 1H), 2.68–2.64 (ovl, 3H), 2.60 (d, *J* = 13.8 Hz, 1H), 2.52–2.33 (ovl, 4H), 2.21–2.11 (ovl, 2H), 2.04 (m, 1H), 1.98 (m, 1H), 1.60 (overlapped with H_2_O, 3H), 0.60 (s, 3H). ^13^C NMR (CDCl_3_, 100 MHz): δ 218.0, 199.5, 159.9, 156.0, 144.5, 143.8, 134.5, 134.1, 133.3, 131.3, 130.5, 129.8 (2C), 127.0 (2C), 123.7, 119.3, 111.7, 104.4, 56.0, 50.8, 47.9, 40.2, 38.1, 37.8, 37.0, 35.6, 31.0, 26.9, 26.2, 22.0, 14.8. HR ESIMS *m*/*z*: 478.4596. [M + H]^+^, C_32_H_31_NO_3_ requires 477.2304.

#### 11β-(3-Cyano-4-methylphenyl)-estra-4,9-dien-3,17-dione (**2m**)

Boronic acid: (3-cyano-4-methylphenyl)­boronic acid. The crude was purified by preparative HPLC employing an Agilent Pre-C18 column (30 × 100 mm, 5 μM) with solvent A (water 0.1% FA) and B (ACN 0.1% FA). The method was as follows: 60% B from 0 to 2 min, 60% B to 80% B in 15 min (*R*_*t*_ = 10.9 min, 63% yield). Appearance: white solid. ^1^H NMR (CDCl_3_, 400 MHz): δ 7.79 (s, 1H), 7.67 (d, *J* = 8.0 Hz, 1H), 7.47 (d, *J* = 8.2 Hz, 2H), 7.37 (d, *J* = 8.1 Hz, 1H), 7.28 (d, *J* = 8.0 Hz, 2H), 5.82 (s, 1H), 4.52–4.43 (d, *J* = 6.8 Hz, 1H), 2.76 (dt, *J* = 14.8, 5.0 Hz, 1H), 2.69–2.65 (ovl, 3H), 2.58 (ovl, 1H), 2.57 (s, 3H), 2.51–2.30 (ovl, 4H), 2.22–2.11 (ovl, 2H), 2.05 (m, 1H), 1.99 (m, 1H), 1.60 (overlapped with H_2_O, 3H), 0.59 (s, 3H). ^13^C NMR (CDCl_3_, 100 MHz): δ 218.9, 199.4, 155.9, 144.3, 140.8, 139.0, 136.7, 131.2, 130.9, 130.8, 130.6, 127.8 (2C), 127.3 (2C), 123.8, 118.3, 113.5, 50.7, 47.9, 40.2, 38.2, 37.9, 36.9, 35.6, 31.0, 26.9, 26.1, 22.0, 20.3, 14.7. HR ESIMS *m*/*z*: 462.4567. [M + H]^+^, C_32_H_31_NO_2_ requires 461.2355.

#### 11β-(3-Cyano-5-methylphenyl)-estra-4,9-dien-3,17-dione (**2n**)

Boronic acid: 3-cyano-5-methylphenylboronic acid. Purification by HPLC on a Luna C18 column (10 × 250 mm, 5 μM) with solvent A (water 0.1% TFA) and B (ACN 0.1% TFA). The method was as follows: 70% B from 0 to 2 min, 70% B to 85% B in 15 min (*R*_*t*_ = 15.3 min, 32% yield). Appearance: white solid. ^1^H NMR (CDCl_3_, 400 MHz): δ 7.64 (s, 1H), 7.59 (s, 1H), 7.48 (d, *J* = 8.4 Hz, 2H), 7.42 (s, 1H), 7.29 (d, *J* = 8.1 Hz, 2H), 5.83 (s, 1H), 4.48 (d, *J* = 7.2 Hz, 1H), 2.77 (dt, *J* = 15.2, 5.0 Hz, 1H), 2.67 (m, 3H), 2.58 (d, *J* = 13.7 Hz, 1H), 2.53–2.46 (ovl, 2H), 2.44 (s, 3H), 2.42–2.29 (ovl, 2H), 2.22–2.15 (ovl, 2H), 2.05–1.97 (ovl, 2H), 1.61 (m, 3H), 0.58 (s, 3H). ^13^C NMR (CDCl_3_, 100 MHz): δ 218.9, 199.3, 155.9, 144.6, 144.3, 141.8, 139.9, 137.0, 132.4, 131.3, 130.6, 127.9, 127.8 (2C), 127.5 (2C), 123.8, 119.1, 112.9, 50.7, 47.9, 40.2, 38.2, 37.9, 36.9, 35.6, 31.0, 26.9, 26.1, 22.0, 21.4, 14.7. HR ESIMS *m*/*z*: 462.4685. [M + H]^+^, C_32_H_31_NO_2_ requires 461.2355.

#### 11β-(5-Cyano-2-methylphenyl)-estra-4,9-dien-3,17-dione (**2o**)

Boronic acid: (5-cyano-2-methylphenyl)­boronic acid. The crude was purified by preparative HPLC employing an Agilent Pre-C18 column (30 × 100 mm, 5 μM) with solvent A (water 0.1% FA) and B (ACN 0.1% FA). The method was as follows: 60% B from 0 to 2 min, 60% B to 80% B in 18 min (*R*_*t*_ = 10.6 min, 45% yield). Appearance: white solid. ^1^H NMR (CDCl_3_, 400 MHz): δ 7.55–7.49 (ovl, 2H), 7.35 (d, *J* = 7.9 Hz, 1H), 7.26 (ovl, 2H), 7.20 (d, *J* = 8.2 Hz, 2H), 5.82 (s, 1H), 4.50 (d, *J* = 7.5 Hz, 1H), 2.79 (dt, *J* = 15.4, 4.5 Hz, 1H), 2.66 (m, 3H), 2.59 (d, *J* = 13.9 Hz, 1H), 2.52–2.43 (ovl, 2H), 2.41–2.34 (ovl, 2H), 2.29 (s, 3H), 2.20–2.11 (m, 2H), 2.05 (m, 1H), 1.99 (m, 1H), 1.60 (overlapped with H_2_O, 3H), 0.59 (s, 3H). ^13^C NMR (CDCl_3_, 100 MHz): δ 219.0, 199.4, 156.0, 144.4, 143.8, 142.6, 141.5, 137.5, 133.4, 131.3, 130.8, 130.6, 129.4 (2C), 127.2 (2C), 123.8, 119.1, 109.9, 50.8, 47.9, 40.2, 38.1, 37.9, 36.9, 35.6, 31.0, 26.9, 26.2, 22.0, 21.0, 14.6. HR ESIMS *m*/*z*: 462.2425. [M + H]^+^, C_32_H_31_NO_2_ requires 461.2355.

#### 11β-(3-Ethynylphenyl)-estra-4,9-dien-3,17-dione (**2p**)

Boronic acid: (3-ethynylphenyl)­boronic acid. The crude was purified by preparative HPLC employing a Luna C18 column (10 × 250 mm, 5 μM) with solvent A (water 0.1% TFA) and B (ACN 0.1% TFA). The method was as follows: 80% B from 0 to 3 min, 80% B to 98% B in 10 min, 50% B to 90%B in 5 min (*R*_*t*_ = 11.6 min, 48% yield). Appearance: white solid. ^1^H NMR (CDCl_3_, 400 MHz): δ 7.71 (s, 1H), 7.55 (dt, *J* = 7.7, 1.5 Hz, 1H), 7.53–7.48 (d, *J* = 8.2 Hz, 2H), 7.46 (dt, *J* = 7.7, 1.4 Hz, 1H), 7.38 (t, *J* = 7.4 Hz, 1H), 7.26 (ovl, *J* = 8.2 Hz, 2H), 5.84 (s, 1H), 4.48 (d, *J* = 7.2 Hz, 1H), 3.10 (s, 1H), 2.77 (dt, *J* = 14.7, 5.0 Hz, 1H), 2.67 (m, 2H), 2.59 (d, *J* = 13.8 Hz, 1H), 2.53–2.33 (ovl, 4H), 2.21–1.96 (ovl, 5H), 1.60 (m, 3H), 0.59 (s, 3H). ^13^C NMR (CDCl_3_, 100 MHz): δ 219.0, 199.4, 156.0, 144.5, 143.8, 140.8, 131.0, 130.8, 130.5, 129.0, 127.6 (2C), 127.5, 127.5 (2C), 123.7, 122.8, 93.1, 83.7, 51.1, 50.8, 47.9, 40.2, 38.2, 37.9, 37.0, 35.6, 31.0, 26.9, 26.1, 22.0, 14.7. HR ESIMS *m*/*z*: 447.4686. [M + H]^+^, C_32_H_30_O_2_ requires 446.2246.

#### 11β-(4-Ethynylphenyl)-estra-4,9-dien-3,17-dione (**2q**)

Boronic acid: (4-ethynylphenyl)­boronic acid. The crude was purified by preparative HPLC employing a Luna C18 column (10 × 250 mm, 5 μM) with solvent A (water 0.1% TFA) and B (ACN 0.1% TFA). The method was as follows: 70% B from 0 to 1 min, 70% B to 95% B in 15 min (*R*_*t*_ = 12.1 min, 43% yield). Appearance: white solid. ^1^H NMR (CDCl_3_, 400 MHz): δ 7.54 (s, 4H), 7.51 (d, *J* = 8.0 Hz, 2H), 7.27 (ovl, 2H), 5.82 (s, 1H), 4.47 (d, *J* = 7.0 Hz, 1H), 3.12 (s, 1H), 2.77 (dt, *J* = 14.6, 4.6 Hz, 1H), 2.66 (m, 3H), 2.59 (d, *J* = 13.4 Hz, 1H), 2.52–2.30 (ovl, 4H), 2.21–2.09 (m, 2H), 2.06–1.96 (m, 2H), 1.60 (overlapped with H_2_O, 3H), 0.59 (s, 3H). ^13^C NMR (CDCl_3_, 100 MHz): δ 219.2, 198.1, 153.0, 149.9, 143.3, 142.0, 141.4, 140.7, 129.9 (2C), 129.3 (2C), 128.9 (2C), 127.3 (2C), 123.0, 122.8, 84.1, 78.9, 50.8, 48.3, 39.7, 36.9, 36.7, 36.0, 33.8, 30.5, 28.5, 24.8, 22.4, 17.6. HR ESIMS *m*/*z*: 447.4686. [M + H]^+^, C_32_H_30_O_2_ requires 446.2246.

#### 11β-(2-Ethynylphenyl)-estra-4,9-dien-3,17-dione (**2r**)

Boronic acid: (2-ethynylphenyl)­boronic acid. The crude was purified by preparative HPLC employing a Luna C18 column (10 × 250 mm, 5 μM) with solvent A (water 0.1% TFA) and B (ACN 0.1% TFA). The method was as follows: 65% B from 0 to 2 min, 65% B to 90% B in 23 min (*R*_*t*_ = 19.3 min, 40% yield). Appearance: white solid. ^1^H NMR (CDCl_3_, 400 MHz): δ 7.60 (d, *J* = 7.5 Hz, 1H), 7.51 (d, *J* = 8.1 Hz, 2H), 7.39 (t, *J* = 7.4 Hz, 1H), 7.36 (d, *J* = 7.4 Hz, 1H), 7.29 (t, *J* = 7.5 Hz, 1H), 7.24 (d, *J* = 8.1 Hz, 2H), 5.82 (s, 1H), 4.49 (d, *J* = 7.1 Hz, 1H), 3.01 (s, 1H), 2.79 (m, 1H), 2.69–2.65 (ovl, 3H), 2.61 (d, *J* = 13.7 Hz, 1H), 2.51–2.36 (ovl, 4H), 2.16 (m, 2H), 2.04 (m, 1H), 1.97 (m, 1H), 1.62 (m, 3H), 0.59 (s, 3H). ^13^C NMR (CDCl_3_, 100 MHz): δ 219.1, 199.7, 156.3, 144.8, 143.9, 143.4, 138.1, 134.1, 130.4, 129.7 (2C), 129.6, 129.1, 127.1, 126.7 (2C), 123.6, 120.5, 83.2, 80.3, 50.8, 47.9, 40.3, 38.1, 37.9, 36.9, 35.6, 31.0, 26.9, 26.1, 22.0, 14.6. HR ESIMS *m*/*z*: 447.4652. [M + H]^+^, C_32_H_30_O_2_ requires 446.2246.

#### 11β-(3-Methylbenzoate)-estra-4,9-dien-3,17-dione (**2s**)

Boronic acid: 3-methoxycarbonylphenylboronic acid. The crude was purified by preparative HPLC employing an Agilent Pre-C18 column (30 × 100 mm, 5 μM) with solvent A (water 0.1% FA) and B (ACN 0.1% FA). The method was as follows: 60% B from 0 to 2 min, 60% B to 80% B in 18 min (*R*_*t*_ = 10.9 min, 63% yield). Appearance: white solid. ^1^H NMR (CDCl_3_, 400 MHz): δ 8.16 (t, *J* = 1.8 Hz, 1H), 7.92 (d, *J* = 7.8 Hz, 1H), 7.77 (d, *J* = 7.7 Hz, 1H), 7.59–7.48 (ovl, 3H), 7.27 (d, *J* = 7.5 Hz, 2H), 5.82 (s, 1H), 4.49 (d, *J* = 7.2 Hz, 1H), 2.78 (dt, *J* = 14.0, 4.8 Hz, 1H), 2.67 (ovl, 3H), 2.65 (s, 3H), 2.59 (d, *J* = 14.1 Hz, 1H), 2.52–2.30 (ovl, 4H), 2.23–2.10 (m, 2H), 2.06–1.97 (m, 2H), 1.60 (overlapped with H_2_O, 3H), 0.60 (s, 3H). ^13^C NMR (CDCl_3_, 100 MHz): δ 218.9, 199.4, 198.2, 155.9, 144.5, 144.0, 141.1, 138.1, 137.8, 131.6, 130.5, 129.2, 127.7 (2C), 127.6 (2C), 127.4, 126.9, 123.7, 50.8, 47.9, 40.2, 38.2, 37.9, 37.0, 35.6, 31.0, 26.9, 26.9, 26.1, 22.0, 14.7. HR ESIMS *m*/*z*: 481.2369. [M + H]^+^, C_32_H_32_O_4_ requires 480.2301.

#### Synthesis of 11β-(3-Benzoic)-estra-4,9-dien-3,17-dione (**2t**)

The methyl ester intermediate (**2s**) was dissolved in MeOH/H_2_O 1:1 v/v, and then NaOH (2 equiv) was added. The reaction mixture was stirred at reflux for 4 h. After TLC monitoring, the reaction was acidified with HCl 6 N solution and extracted with ethyl acetate (3 × 10 mL), dried over anhydrous Na_2_SO_4_, filtered, and concentrated to give the corresponding compound (**2t**). The crude was purified by preparative HPLC employing an Agilent Pre-C18 column (30 mm × 100 mm, 5 μM) with solvent A (water 0.1% FA) and B (MeOH 0.1% FA). The method was as follows: 65% B from 0 to 1 min, 65% B to 85% B in 15 min (*R*_*t*_ = 13.4 min, 20% yield). Appearance: white solid.

#### 11β-(3-Benzoic)-estra-4,9-dien-3,17-dione (**2t**)

^1^H NMR (CDCl_3_, 400 MHz): δ 8.31 (t, *J* = 1.8 Hz, 1H), 8.05 (dt, *J* = 7.8, 1.3 Hz, 1H), 7.82 (ddd, *J* = 7.7, 2.0, 1.1 Hz, 1H), 7.63–7.47 (m, 3H), 7.30 (d, *J* = 8.1 Hz, 2H), 5.83 (s, 1H), 4.49 (d, *J* = 7.3 Hz, 1H), 2.78 (dt, *J* = 14.4, 5.1 Hz, 1H), 2.67 (ovl, 3H), 2.60 (d, *J* = 13.6 Hz, 1H), 2.53–2.32 (ovl, 4H), 2.22–2.10 (m, 2H), 2.07–1.97 (m, 2H), 1.60 (overlapped with H_2_O, 3H), 0.60 (s, 3H). ^13^C NMR (CDCl_3_, 100 MHz): δ 219.2, 198.0, 167.6, 152.1, 149.9, 141.4, 140.8, 140.4, 138.6, 131.2, 130.2, 129.9, 129.0, 128.5, 127.5, 126.5, 122.8, 50.5, 48.3, 39.7, 36.9, 36.7, 36.1, 33.8, 30.5, 28.5, 24.5, 22.4, 18.8. HR ESIMS *m*/*z*: 466.4561 [M – H]^−^, C_31_H_30_O_4_ requires 466.2144.

### AlphaScreen on LIF-LIFR

Recombinant human LIFR (His-Tag) and LIF (biotinylated) were purchased from Sino Biologicals (Sino Biological Europe GmbH, Dusseldorf, Germany) and R&D Systems (Abingdon, UK), respectively, and both were reconstituted as required by the manufacturer. Inhibition of LIFR/LIF binding was measured by AlphaScreen, in white, low-volume, 384-well AlphaPlates (PerkinElmer, Waltham, MA, USA) using a final volume of 15 μL and an assay buffer containing 25 mM Hepes (pH 7.4), 100 mM NaCl, and 0.005% Kathon. The concentration of DMSO in each well was maintained at 5% v/v. LIFR (His-Tag, final concentration 4.5 nM) was incubated with the compounds or DMSO for 45 min under continuous shaking. Then, LIF was added (biotinylated, final concentration 9 nM), and the samples were incubated for 15 min prior to adding nickel chelate acceptor beads (final concentration 20 ng/μL) for 30 min. Then, streptavidin donor beads were added (final concentration 20 ng/μL), and the plate was incubated in the dark for 2 h and then read in an EnSpire Alpha multimode plate reader (PerkinElmer, Waltham, MA, USA). Each molecule’s IC_50_ was calculated by fitting curves obtained from testing at least 8 concentrations. Each concentration was tested in triplicate (*N* = 3). When present, outliers were removed, resulting in *N* = 2. Data, reported as averaged intensity values ± standard deviation and *N*, were exploited to fit the curves through GraphPad Prism 8.

### In Vitro Metabolic Stability

Human liver S9 fraction (Sigma-Aldrich, St. Louis, MO, USA) was used. All incubations were performed in duplicate in a thermoblock (Dlab dry bath HB 120 S) at 37 °C. Each incubation mixture contained the compound at a final concentration of 1 μM with 0.5% DMSO, human liver S9 fraction (0.3 mg of protein per mL), 5 mM taurine, 5 mM glycine, 5 mM glucose 6-phosphate, 5 mM GSH, 1 mM NADPH, 1 mM ATP, 0.4 mM CoA, 0.4 mM UDPGA, 0.1 mM PAPS, and 0.4 U·mL^–1^ glucose 6-phosphate dehydrogenase, in a total volume of 0.25 mL. The incubation buffer was 50 mM potassium phosphate buffer (pH 7.4), containing 5 mM MgCl_2_. At defined times of 0, 30, 60, 120, 180, and 300 min after S9 addition, 25 μL aliquots were withdrawn and the enzymatic reaction was quenched with 100 μL of ice-cold ACN. The samples were centrifuged for 10 min at 10,000 rpm, and the supernatants were transferred into vials for LC–MS analysis, employing the conditions described above. The slope of the linear regression of the curve obtained by reporting the natural logarithm of compound area versus incubation time (−*k*) was used in the conversion to in vitro *t*_1/2_ values, calculated as *t*_1/2_ = −ln(2)/*k*. In vitro intrinsic clearance (Cl_int_ expressed as μL/min/mg) was obtained by first calculating the *V* using the following formula: *V* = volume of reaction (μL)/protein of liver microsomes (mg); intrinsic clearance was then calculated as Cl_int_ = (*V* × ln 2)/*t*_1/2_. Testosterone was used as a positive control for phase I enzymes, and 7-hydroxycoumarin was used as a positive control for phase II enzymes.

### Molecular Modeling

#### Receptor and Ligand Preparation

##### GPBAR1

The human cryo-EM structure of GPBAR1 in the active conformation (PDB ID: 7CFN)^39^ was downloaded from the Protein Data Bank website (http://www.rcsb.org). The ligand, along with cholesterol and palmitic acid molecules, was removed. The GPBAR1-Gα_s_β_1_γ_2_ homology model system was generated by adding the missing residues using the Modeler 10.5 software package (manuscript in preparation).^48^ The receptor was prepared using the Protein Preparation Wizard tool^49^ implemented in Maestro GUI ver. 22.4 ^50^ to assign bond orders, add hydrogen atoms, adjust disulfide bonds, and assign residues’ protonation state at pH 7.4. The GPBAR1 ligand-binding site was used to define the inner grid box coordinates (10.0 Å in size).

##### *h*LIFR

The X-ray structure of *h*LIFR (PDB ID: 3E0G) was downloaded from the Protein Data Bank website and prepared with the Protein Preparation Wizard tool to apply structural corrections such as adding missing hydrogen atoms, determining the most probable protonation states of amino acids, and correcting residues with missing atoms. The prepared structure obtained was then used for docking studies by positioning the grid box with a default coordinate size of 10 Å on the centroid of the *h*LIFR binding site defined by loops L2–L3.

The three-dimensional structure of compound **2o** was generated using the graphical user interface (GUI) of Maestro ver. 22.4.^50^ The protonation state at pH 7.4 in water was calculated using the Epik module.^51^ Finally, the ligand was minimized using the OPLS2005 force field^52^ through 2500 iteration steps of the Polak–Ribiere Conjugate Gradient (PRCG) algorithm.^53^

##### Docking Protocol

For both receptors, we used the same two-step protocol, which was successfully adopted in our previous work.^21^ In particular, the initial step was carried out using the QPLD^54^ algorithm to enhance docking accuracy by incorporating ligand charges derived from ab initio calculations. The most energetically favorable protein–ligand poses were then subjected to a second docking step using the IFD protocol^55^ to account for the simultaneous structural adaptation of both the ligand and the protein. During the QPLD step, 10 docking poses were generated for each ligand, and the most energetically favorable poses were forwarded to the second IFD step. The extended sampling protocol was applied in this step, generating up to 80 poses within an energy window of 2.5 kcal/mol for the ligand conformational sampling.

### MDs Simulations

#### GPBAR1

The best-scored IFD docking pose of **2o** in GPBAR1-Gα_s_β_1_γ_2_ complex was subjected to three replicas of 200 ns each of MD simulation (MDs). An *N*-acetylglucosamine glycosylation was introduced at ASN76, and the system was placed in a 16 × 16 × 17 nm box, embedded in a lipid bilayer composed of cholesterol (CHL) and 1-palmitoyl-2-oleoyl-*sn*-glycero-3-phosphocholine (POPC) at a 30:70 ratio, using the CHARMM-GUI webserver (http://charmm-gui.org/).^56^ For solvation, TIP3P water molecules were employed, and NaCl was added to a final concentration of 150 mM to maintain electrostatic neutrality. The simulation was carried out using the CHARMM36 force field^57^ within the GROMACS 2021.5 software package.^58^ The system was first energy-minimized using the steepest descent algorithm for 5000 steps, followed by equilibration for 100 ps under isothermal–isovolumetric (*NVT*) conditions and 50 ns under isothermal–isobaric (*NPT*) conditions. *NVT* equilibration was performed to gradually heat the system to 300 K, using a 1 fs time step with positional restraints applied to the heavy atoms of the protein, membrane, and ligand to maintain their initial conformations. *NPT* equilibration was carried out in five consecutive 10 ns steps, allowing the system to relax by progressively reducing the positional restraints with a 2 fs time step. A final 2 ns *NPT* equilibration was then conducted without any restraints. During equilibration, the system’s temperature and pressure were maintained at 300 K and 1 bar, respectively, using velocity rescaling for temperature coupling^59,60^ and a Parrinello–Rahman barostat with semi-isotropic pressure coupling.^61,62^ The LINCS algorithm was applied to constrain all bonds involving hydrogen atoms.^63^ A 12 Å cutoff was set for short-range nonbonded interactions, while long-range electrostatic interactions were calculated using the particle Mesh Ewald (PME) summation method^64^ with a Fourier grid spacing of 1.2 Å. Three independent production runs of 200 ns each were conducted under the same conditions, with the temperature regulated using a Nosé–Hoover thermostat.^65,66^ The cluster analysis trajectory was carried out on the merged trajectories of the three MD replicas using the GROMACS gmx cluster tools with the GROMOS method^67^ and a 0.15 nm cutoff. PCA and covariance analyses were conducted using the gmx covar and gmx anaeig tools.

#### *h*LIFR

MDs were performed with the ibverbs version of NAMD ver. 2.14. Specifically, *h*LIFR was parametrized with AMBER ff14SB force field,^68^ while for compound **2o** the General Amber Force Field 2 (GAFF2) force field was used.^69^ Ligand charges were computed via the restrained electrostatic potential (RESP) fitting procedure with the Gaussian16 package, generating the ESP at the Hartree–Fock level (6-31G* basis set). The Antechamber and Leap modules of AmberTools23 ^70^ were used to generate ligand topologies with RESP charges and GAFF2 parameters.

The *h*LIFR/**2o** complex was then solvated in a pre-equilibrated 10 Å cubic TIP3P water box and neutralized with Na^+^ and Cl^–^ ions. Four minimization steps were performed: (i) minimization of only hydrogen atoms (5000 steps), (ii) minimization of water and hydrogen atoms keeping the solute restrained (20.000 steps), (iii) minimization of the protein side-chains, water, and hydrogen atoms (50.000 steps), while the protein backbone atoms and ligand atoms were restrained, and (iv) full system minimization without any restraint (100.000 steps). The minimized system was then subjected to three stages of equilibration: (i) 5 ns in *NVT* ensemble with the Langevin thermostat gradually increasing the temperature from 0 K to 300 K every 50 K, and gradually relaxing the solute constraints from 10 to 1 kcal/mol Å^2^, (ii) 5 ns *NPT* ensemble at 1 atm using the Langevin piston to control the system pressure, and (iii) 5 ns *NPT* without restraints. A 200 ns long MD production run was performed in the *NPT* ensemble (2 fs time step), using the SHAKE algorithm for hydrogen-containing bonds and PME for long-range electrostatics (10 Å cutoff). MD trajectories were visualized in VMD 1.9.3,^71^ while clustering analysis, SASA, and PCA were performed via CPPTRAJ.^72^ All figures were rendered with PyMOL 2.5.0 (http://www.pymol.org/pymol).

#### Transactivation Assay

Hek293T cells, obtained from ATCC (Manassas, VA, USA), were cultured in a D-MEM medium (Sigma-Merk Life Science S.r.l., Milan, Italy) enriched with 10% fetal bovine serum, 1% l-glutamine, and 1% penicillin/streptomycin. HEPG2 cells, also from ATCC, were grown in an MEM medium (Euroclone, reference ECB2071L) supplemented with 10% fetal bovine serum (Euroclone, reference ECS5000L), 1% l-glutamine (Euroclone, reference ECB3000D), and 1% penicillin/streptomycin (Euroclone, reference ECB3001D). All cultures were maintained in a humidified environment with 5% CO_2_ at 37 °C. Additionally, it was confirmed that the cells were free from Mycoplasma contamination, as verified using the Mycoplasma PCR Detection Kit (Aurogene, reference G238).

The luciferase reporter gene assay protocol takes place over four days. On the first day, HEPG2 or HEK293T cells are seeded in a 24-well plate at a density of 7.5 × 10^4^ cells/well for HEPG2 or 2.5 × 10^4^ cells/well for HEK293T. On the second day, cells are transiently transfected, preparing a separate mix for each receptor. To assess the phosphorylation of STAT3 signaling, HEPG2 cells were transfected with 200 ng of the reporter vector containing the Pgl4.47 response element, along with 100 ng each of LIFR, IL6ST (gp130), and pGL4.70 (Promega, Madison, WI), a vector encoding the human Renilla gene. The following day, the cells were stimulated with 10 ng/mL LIF, either alone or in combination with **2o** at concentrations of 0.1, 1, 10, 25, and 50 μM, to generate a dose–response curve. For GPBAR1 agonism, HEK293T cells were transfected with 200 ng of the reporter vector pG29-CRE-LUC, 150 ng of pcmvsport-hTGR5, and 150 ng of pGL4.70, the human Renilla gene. The following day, cells were stimulated with 10 μM TLCA alone as a positive control and compound **2o** at concentrations of 1 and 10 nM, as well as 0.1, 1, 2.5, 5, 10, 20, and 30 μM, to generate a dose–response curve. On the final day, cellular lysates were analyzed for luciferase and Renilla activities using the Dual-Luciferase Reporter Assay System (Promega, Madison, WI). pLuminescence was measured with a Glomax 20/20 luminometer (Promega, Madison, WI), and luciferase activities were normalized to Renilla activities.

### Human HSC (LX-2)

LX-2 cells (provided by Cytion, reference 305039) were grown in a high-glucose D-MEM medium (Euroclone, reference ECB7501L), supplemented with 2% fetal bovine serum, 1% l-glutamine, and 1% penicillin/streptomycin. LX2 cells were seeded at a density of 2.5 × 10^5^ cells per well in a 6-well plate (2 mL/well) using their specific culture medium. The following day, cells were starved in FBS-free DMEM for 24 h. They were then stimulated with LIF (10 ng/mL) alone or in combination with **2o** at concentrations of 1, 5, 10, and 20 μM. After 24 h of treatment, RNA extraction was performed using Tri-Reagent (Zymo Research) and the Direct-zol RNA MicroPrep kit with Zymo-Spin IIC Columns (Zymo Research, Irvine, CA). Relative gene expression analysis was then conducted.

### Reverse and Real-Time PCR

Following genomic DNA removal using DNase I (Thermo Fisher Scientific, Waltham, MA), 1–2 μg of total RNA from each sample was reverse-transcribed into cDNA using the FastGene Scriptase Basic Kit (Nippon Genetics, Mariaweilerstraβe, Düren, Germany) in a total reaction volume of 20 μL. Subsequently, 50 ng of cDNA was amplified in a 20 μL reaction mixture containing 200 nM of each primer and 10 μL of either PowerUp SYBR Green Master Mix or TaqMan Gene Expression Master Mix (Thermo Fisher Scientific, Waltham, MA). All qPCR reactions were performed in triplicate, following the thermal cycling conditions specified in the respective datasheets.

Primer sequences were designed using PRIMER3 software (http://frodo.wi.mit.edu/primer3/) based on published sequences available in the NCBI database. The primers used for the analysis of mouse genes were as follows [forward (for) and reverse (rev)]:

LIFR (*H. Sapiens*; for GCTCGTAAAATTAGTGACCCACA; rev GCACATTCCAAGGGCATATC), GPBAR1 (*H. Sapiens*; for ACTGCAGCTCCCAGGCTAT; rev GACAGAGAGGAAGGCAGCA) COL1A1 (*H. sapiens*; for CCCAAGGCTTCCAAGGTC; rev GACCAGGTTTTCCAGCTTCC), ASMA (*H. sapiens*; for GTGTTCCCGTCCATCGTG; rev CTCTTGCTCTGAGCCTCGTC), TGFB (*H. sapiens*; for GTGGAAACCCACAACGAAAT; rev CACGTGCTGCTCCACTTTTA), TIMP1 (*H. sapiens*; for ACATCCGGTTCGTCTACACC; rev GGGGGCCGTGTAGATAAACT), MMP-9 (*H. sapiens*; for CCGGACCAAGGATACAGTTT; rev TCAGTGAAGCGGTACATAGGG), Col1a1 (*M. musculus*; for TGACTGGAAGAGCGGAGAGT; rev AGACGGCTGAGTAGGGAACA), Asma (*M. musculus*; for AGAGCTACGAACTGCCTGAC; rev TAGGTGGTTTCGTGGATGCC), Tgfb (*M. musculus*; for TTGCTTCAGCTCCACAGAGA; rev TGGTTGTAGAGGGCAAGGAC), Lif (*M. musculus*, TaqMan probe), Lifr (*M. musculus*; for CTGGTGATCACGAAGTCACA; rev GATCTCGGGAGTCTCTGGA), Gpbar1 (*M. musculus*; TaqMan probe), Fxr (*M. musculus*; for AGCTTCCAGGGTTTCAGACA; rev CTTCCAACAGGTCTGCATGA), Shp (*M. musculus*; for ACGATCCTCTTCAACCCAGA; rev AGGGCTCCAAGACTTCACAC), Pxr (*M. musculus*; for ACGGCAGCATCTGGAACTAC; rev TGGTCCTCAATAGGCAGGTC), Lxr (*M. musculus*; for GGCTCACCAGCTTCATTAGC; rev GCAGGACCAGCTCCAAGTAG), Vdr (*M. musculus*; TCACAGATGAGGAGGTGCAG; rev GAGCAGGATGGCGATAATGT), Pparα (*M. musculus*; CAGAGGTCCGATTCTTCCAC; rev GATCAGCATCCCGTGTTTGT), Pparγ (*M. musculus*; GCCAGTTTCGATCCGTAGAA; rev AATCCTTGGCCCTCTGAGAT), Fgf21 (*M. musculus*; CCTGGGTGTCAAAGCCTCTA; rev CTCCAGCAGCAGTTCTCTGA).

### Animals and Fibrosis Protocols

Male C57BL/6J mice, purchased from Charles River, were housed in the animal facility of the University of Perugia under controlled environmental conditions, including a constant temperature of 22 °C and a 12:12 h light/dark cycle. Mice had ad libitum access to standard chow and tap water and were acclimated to these conditions for at least 7 days before inclusion in the experiments. All procedures were conducted in accordance with EU and Italian regulations and were approved by the Ethical Committee of the University of Perugia and the National Committee of the Italian Ministry of Health (permit no. 214/2017 PR). Animal health and body conditions were monitored daily by the facility veterinarian. Only male mice aged 10–12 weeks were included in the experiments, with 5–8 animals per group. Liver fibrosis was induced by intraperitoneal (i.p.) administration of carbon tetrachloride (CCl_4_) at a dose of 500 μL/kg, dissolved in an equal volume of olive oil, and administered twice per week for 1 week, as previously described. CCl_4_-treated mice were randomly assigned to receive compound **2o** at a dose of 10 mg/kg/day via oral gavage starting from the first day of the experiment. Mice were sacrificed 1 week later. At the end of the study, animals were euthanized with an overdose of anesthetic, and blood and liver samples were collected for biochemical, histological, and gene expression analyses.

### Histological Analysis

H&E staining: For histological analysis, liver lobe samples were fixed in 10% formalin, embedded in paraffin, sectioned, and stained with H&E to assess tissue morphology.

SR staining: For fibrosis evaluation, liver lobe samples were fixed in 10% formalin, embedded in paraffin, sectioned, and stained with SR.

Fibrotic area quantification was performed using ImageJ software by measuring the percentage of SR-positive stained regions (% area) (https://imagej.net/ij/docs/examples/stained-sections/index.html).

### Pharmacokinetics Analysis in Rats

The pharmacokinetic profile of compound **2o** was evaluated in three male C57BL/6J mice (10–12 weeks old; Charles River). All experimental procedures were approved by and conducted in accordance with EU and Italian regulations and were approved by the Ethical Committee of the University of Perugia and the National Committee of the Italian Ministry of Health (permit 214/2017 PR). At five time points (0, 1, 3, 6, and 24 h) after oral cassette dosing of 10 mg/kg (vehicle: methylcellulose), blood was collected from the tail vein into Li-heparin tubes, cooled on dry ice within 1–2 min of sampling, and stored at −20 °C until further processing. Compound **2o** was quantified by LCMS, and pharmacokinetic analysis was carried out using the standard trapezoidal rule.

## Supplementary Material







## Data Availability

The atomic coordinates of the molecular complexes discussed are available on the public repository Zenodo.^73^ Authors will release the atomic coordinates upon article publication.

## Notes

^1^H and ^13^C NMR of compounds **1a–1h** and **2a–2t**, HPLC traces of compounds **1c**, **2l**, **2o**, and **2s**, in vitro antagonism profile assessed by Alphascreen assay, and docking and molecular dynamics results (PDF)
Molecular formula strings (CSV)
MD simulation trajectory movies (ZIP)

## Abbreviations

ALT: alanine transaminase
AST: aspartate transaminase
CCL-2: chemokine (C–C motif) ligand 2
FXR: farnesoid-X-receptor
GPBAR1: G protein-coupled bile acid receptor 1
IL-6: interleukin-6
IL-17: interleukin 17
JAK: Janus kinase
LIF: leukemia inhibitory factor
LRIs: leukemia receptor inhibitors
MAPK: mitogen-activated protein kinases
MDs: molecular dynamics simulations
PDAC: pancreatic ductal adenocarcinoma cells
PI3K/AKT: phosphoinositide 3-kinase/protein kinase B
STAT: signal transducer and activator of transcription
THF: tetrahydrofuran
TNF-α: tumor necrosis factor
TGF-β: transforming growth factor beta
