# Supplementary material for: Harnessing the Estradienone Scaffold to Develop Dual GPBAR1 and LIFR Modulators for Liver Fibrosis
Source: J Med Chem. 2025 Sep 24;68(19):20037–59. doi: 10.1021/acs.jmedchem.5c00705 (PMC12516728; doi:10.1021/acs.jmedchem.5c00705)
Supplement: Supplementary file 1 [file jm5c00705_si_001.pdf]

Supporting Information

Harnessing the Estradienone Scaffold to Develop Dual GPBAR1 and LIFR Modulators for Liver Fibrosis

Rosa De Gregorio,[1,°] Federica Moraca,[1,°] Pasquale Rapacciuolo,[1] Bianca Fiorillo,[1] Elva Morretta,[1] Cristina Di Giorgio,[2] Silvia Marchianò,[2] Ginevra Lachi,[2] Carmen Massa,[2] Benedetta Sensini,[2] Michele Biagioli,[2] Lucio Spinelli,[1] Maria Chiara Monti,[1] Bruno Catalanotti,[1] Valentina Sepe,[1,*] Stefano Fiorucci,[2] Angela Zampella[1]

[1]Department of Pharmacy, University of Naples "Federico II", Via D. Montesano, 49, I-80131 Naples, Italy

[2]Department of Medicine and Surgery, University of Perugia, Piazza L. Severi 1-06132 Perugia, Italy

°Contributed equally to this work.

*To whom correspondence should be addressed: valentina.sepe@unina.it

Table of contents	S1-3
^{1}H-NMR spectrum of compound **1a**	S4
^{13}C-NMR spectrum of compound **1a**	S4
^{1}H-NMR spectrum of compound **1b**	S5
^{13}C-NMR spectrum of compound **1b**	S5
^{1}H-NMR spectrum of compound **1c**	S6
^{13}C-NMR spectrum of compound **1c**	S6
^{1}H-NMR spectrum of compound **1d**	S7
^{13}C-NMR spectrum of compound **1d**	S7
^{1}H-NMR spectrum of compound **1e**	S8
^{13}C-NMR spectrum of compound **1e**	S8
^{1}H-NMR spectrum of compound **1f**	S9
^{13}C-NMR spectrum of compound **1f**	S9
^{1}H-NMR spectrum of compound **1g**	S10
^{13}C-NMR spectrum of compound **1g**	S10
^{1}H-NMR spectrum of compound **1h**	S11
^{13}C-NMR spectrum of compound **1h**	S11
^{1}H-NMR spectrum of compound **2a**	S12
^{13}C-NMR spectrum of compound **2a**	S12
^{1}H-NMR spectrum of compound **2b**	S13
^{13}C-NMR spectrum of compound **2b**	S13

^{19}F-NMR spectrum of compound **2b** S14
^{1}H-NMR spectrum of compound **2c** S15
^{13}C-NMR spectrum of compound **2c** S15
^{19}F-NMR spectrum of compound **2c** S16
^{1}H-NMR spectrum of compound **2d** S17
^{13}C-NMR spectrum of compound **2d** S17
^{1}H-NMR spectrum of compound **2e** S18
^{13}-NMR spectrum of compound **2e** S18
^{1}H-NMR spectrum of compound **2f** S19
^{13}C-NMR spectrum of compound **2f** S19
^{1}HNMR spectrum of compound **2g** S20
^{13}C NMR spectrum of compound **2g** S20
^{1}H-NMR spectrum of compound **2h** S21
^{13}C-NMR spectrum of compound **2h** S21
^{1}H-NMR spectrum of compound **2i** S22
^{13}C-NMR spectrum of compound **2i** S22
^{1}H-NMR spectrum of compound **2j** S23
^{13}C-NMR spectrum of compound **2j** S23
^{1}H-NMR spectrum of compound **2k** S24
^{13}C-NMR spectrum of compound **2k** S24
^{1}H-NMR spectrum of compound **2l** S25
^{13}C-NMR spectrum of compound **2l** S25
^{1}H-NMR spectrum of compound **2m** S26
^{13}C-NMR spectrum of compound **2m** S26
^{1}H-NMR spectrum of compound **2n** S27
^{13}C-NMR spectrum of compound **2n** S27
^{1}H-NMR spectrum of compound **2o** S28
^{13}C-NMR spectrum of compound **2o** S28
^{1}H-NMR spectrum of compound **2p** S29
^{13}C-NMR spectrum of compound **2p** S29
^{1}H-NMR spectrum of compound **2q** S30
^{13}C-NMR spectrum of compound **2q** S30
^{1}H-NMR spectrum of compound **2r** S31
^{13}C-NMR spectrum of compound **2r** S31
^{1}H-NMR spectrum of compound **2s** S32
^{13}C-NMR spectrum of compound **2s** S32
^{1}H-NMR spectrum of compound **2t** S33
^{13}C-NMR spectrum of compound **2t** S33
HPLC trace of compound **1a** S34
HPLC trace of compound **1b** S34
HPLC trace of compound **1c** S35
HPLC trace of compound **1d** S35
HPLC trace of compound **1e** S36
HPLC trace of compound **1f** S36
HPLC trace of compound **1g** S37
HPLC trace of compound **1h** S37
HPLC trace of compound **2a** S38
HPLC trace of compound **2b** S38
HPLC trace of compound **2c** S39
HPLC trace of compound **2d** S39
HPLC trace of compound **2e** S40
HPLC trace of compound **2f** S40

HPLC trace of compound **2g** S41
HPLC trace of compound **2h** S41
HPLC trace of compound **2i** S42
HPLC trace of compound **2j** S42
HPLC trace of compound **2k** S43
HPLC trace of compound **2l** S43
HPLC trace of compound **2m** S44
HPLC trace of compound **2n** S44
HPLC trace of compound **2o** S45
HPLC trace of compound **2p** S45
HPLC trace of compound **2q** S46
HPLC trace of compound **2r** S46
HPLC trace of compound **2s** S47
HPLC trace of compound **2t** S47
HRMS spectrum of compound **1c** S48
HRMS spectrum of compound **2h** S48
HRMS spectrum of compound **2o** S49
HRMS spectrum of compound **2s** S49
Figure S1 S50
Figure S2 S51
Figure S3 S51
Figure S4 S52
Figure S5 S52
Figure S6 S53
Figure S7 S53
Figure S8 S53
Figure S9 S54
Figure S10 S54
Figure S11 S55
Figure S12 S55
Figure S13 S56
Figure S14 S56

^{1}H NMR (400 MHz, $CDCl_3$) Compound **1a**

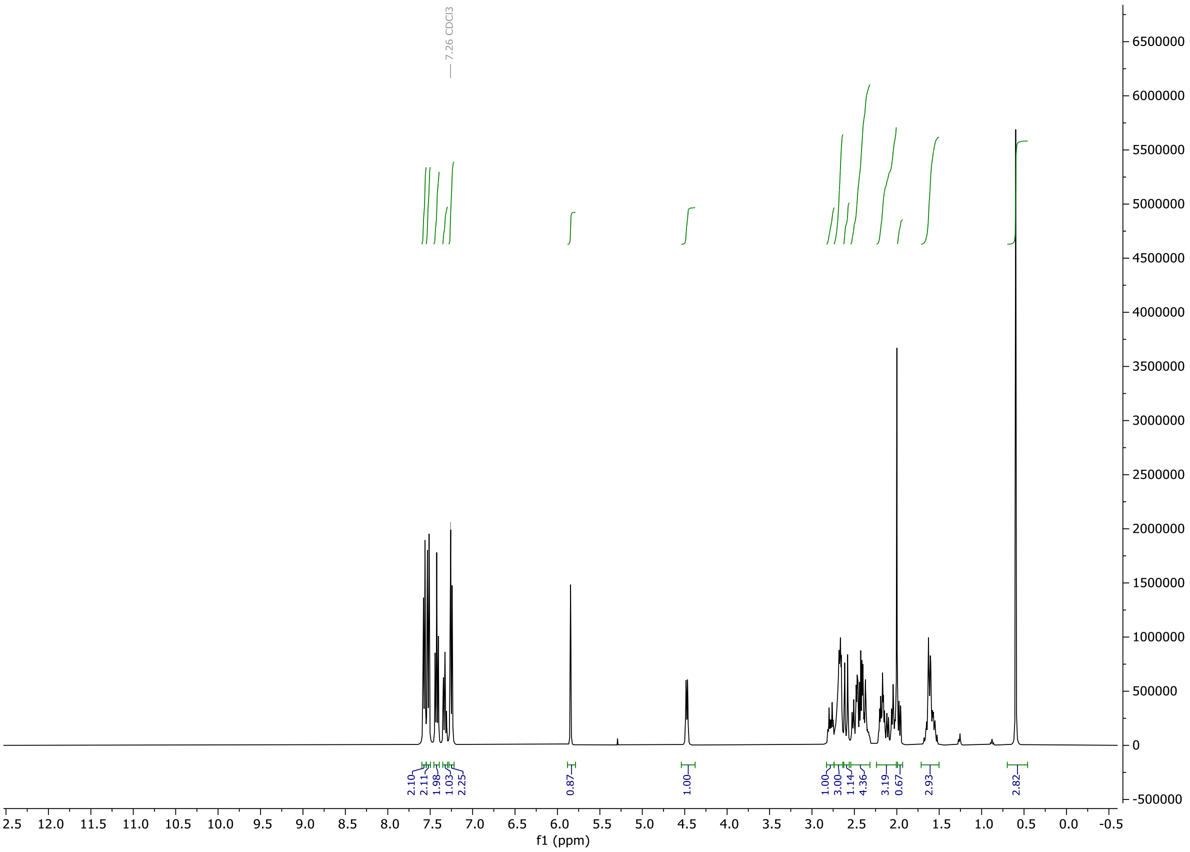

^{13}C NMR (100 MHz, $CDCl_3$) Compound **1a**

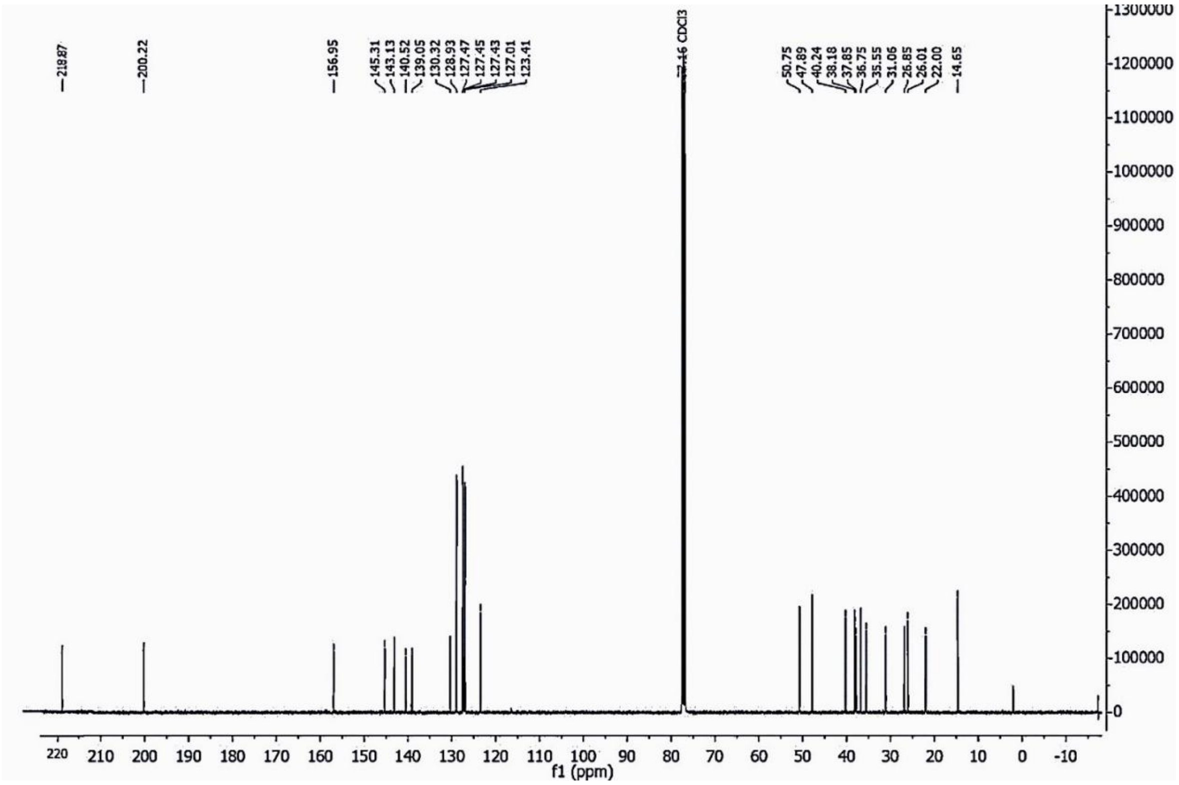

^{1}H NMR (400 MHz, CDCl₃) Compound **1b**

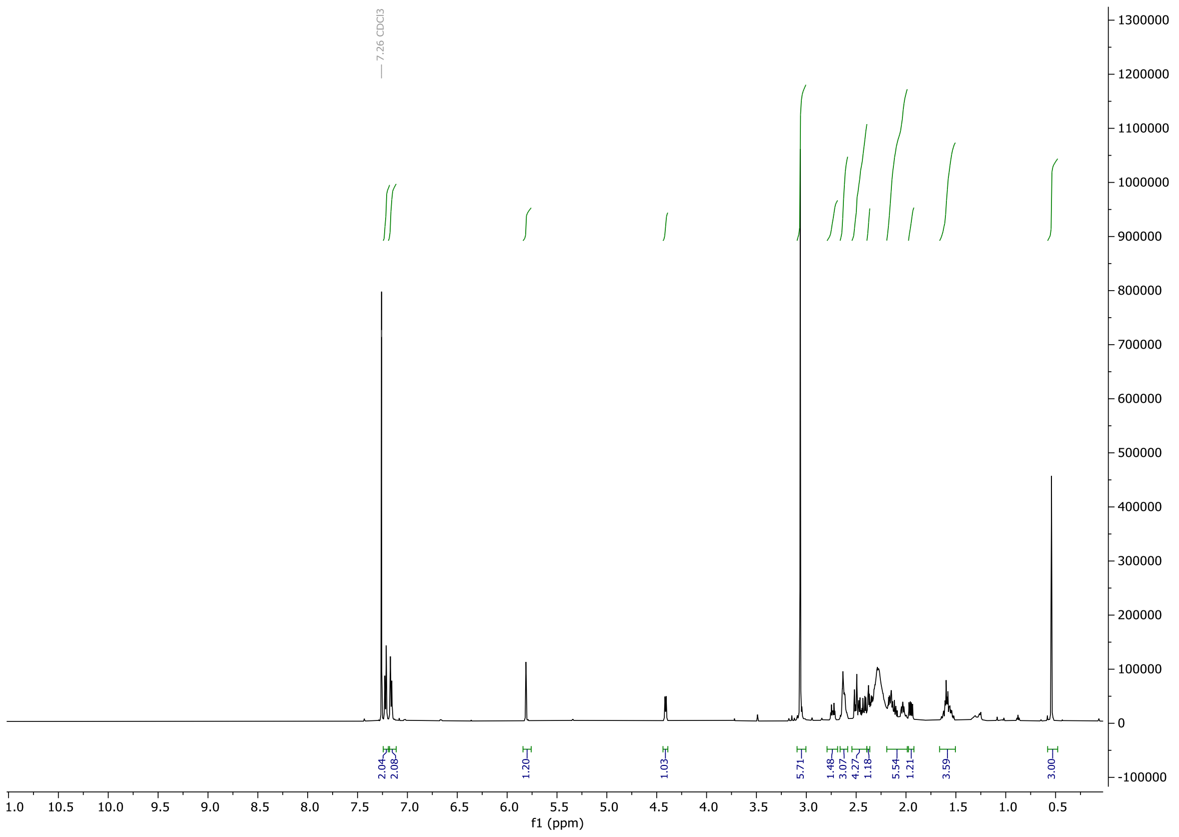

^{13}C NMR (100 MHz, CDCl₃) Compound **1b**

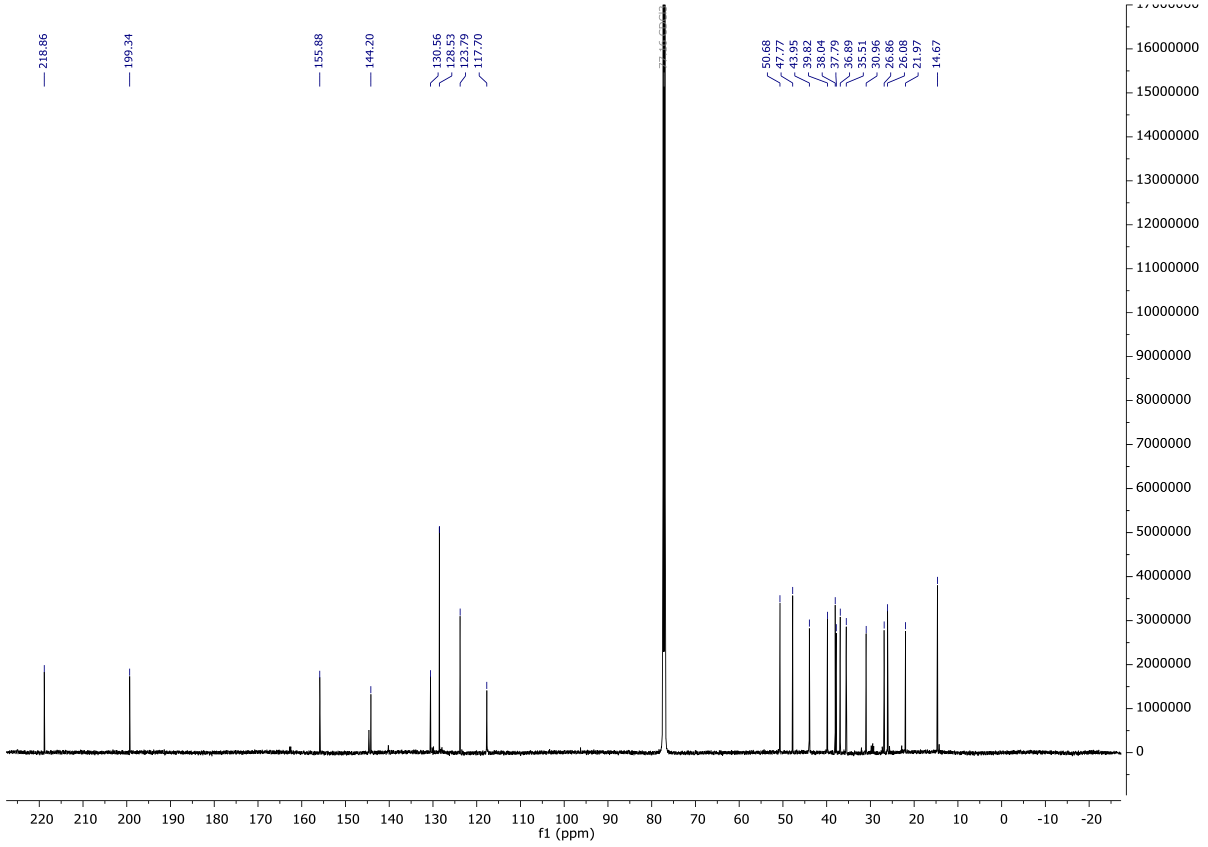

^{1}H NMR (400 MHz, $CDCl_3$) Compound **1c**

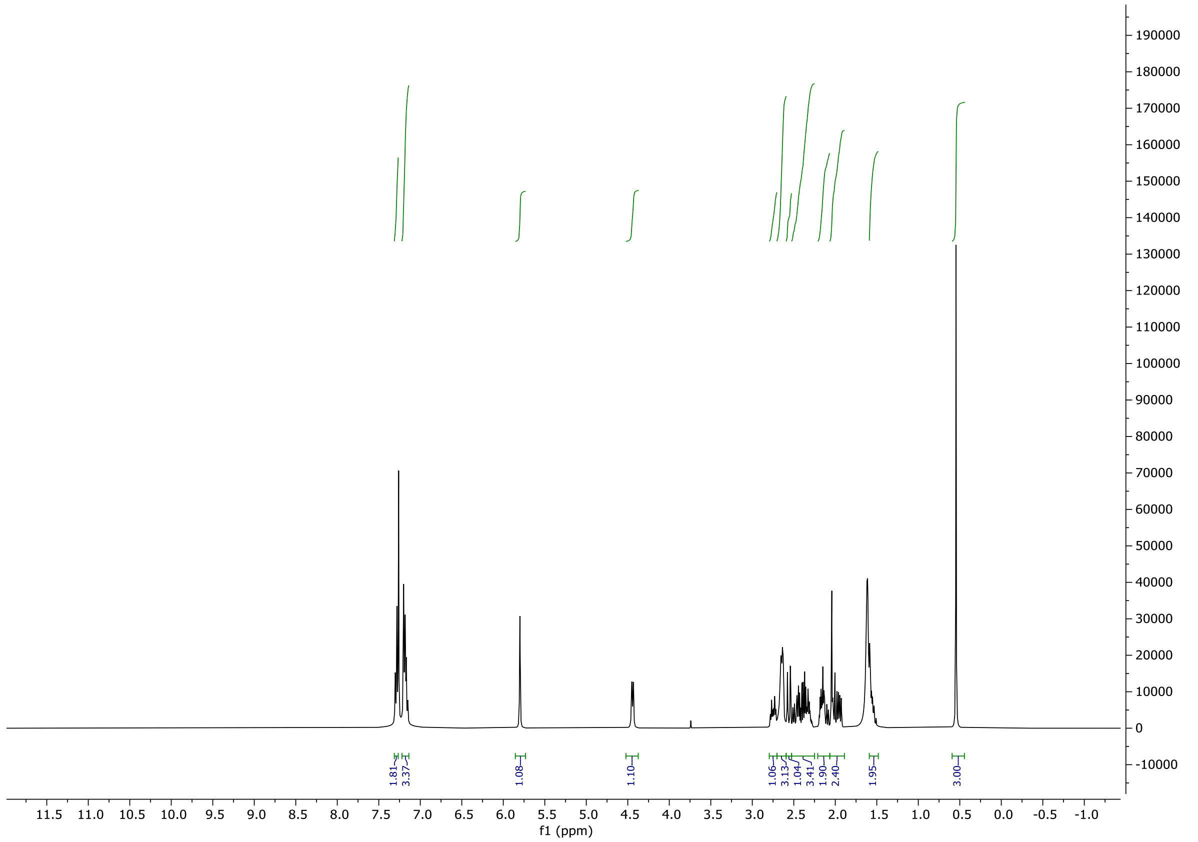

^{13}C NMR (100 MHz, $CDCl_3$) Compound **1c**

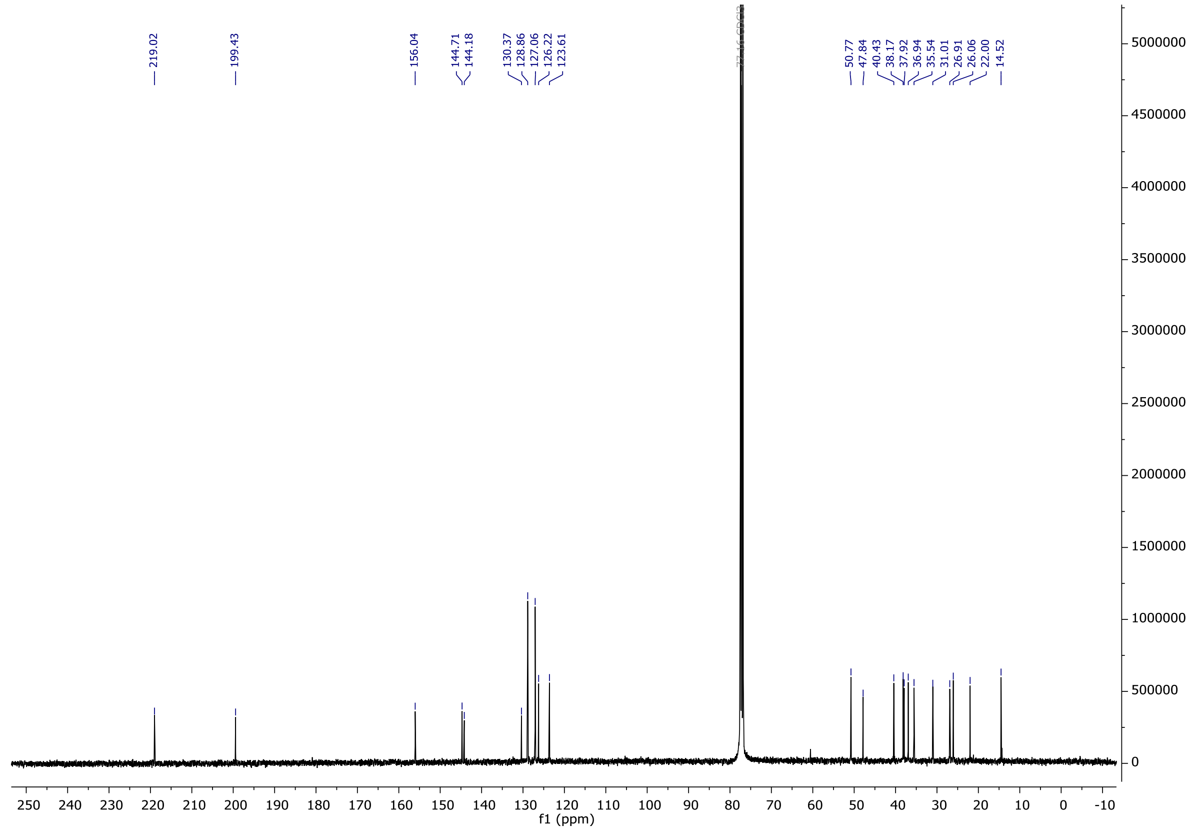

^{1}H NMR (400 MHz, $CDCl_3$) Compound **1d**

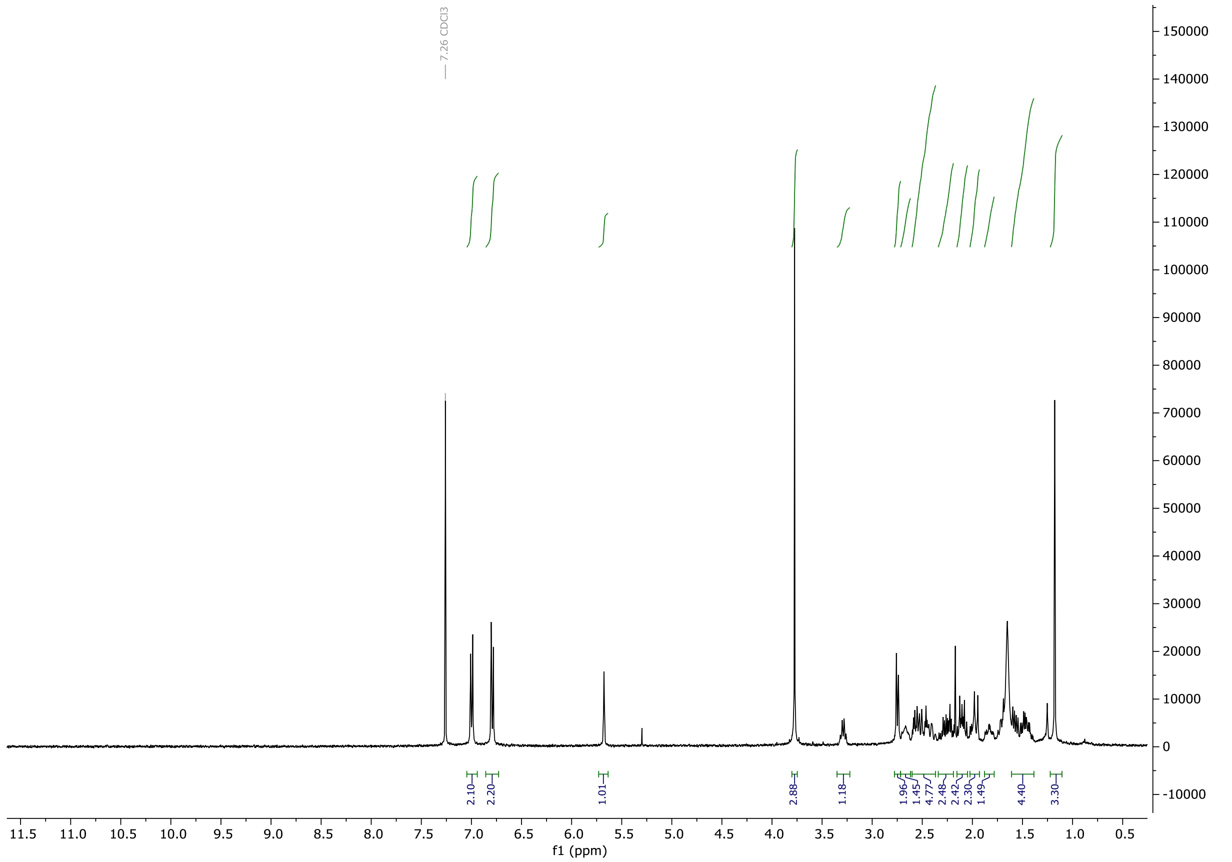

^{13}C NMR (100 MHz, $CDCl_3$) Compound **1d**

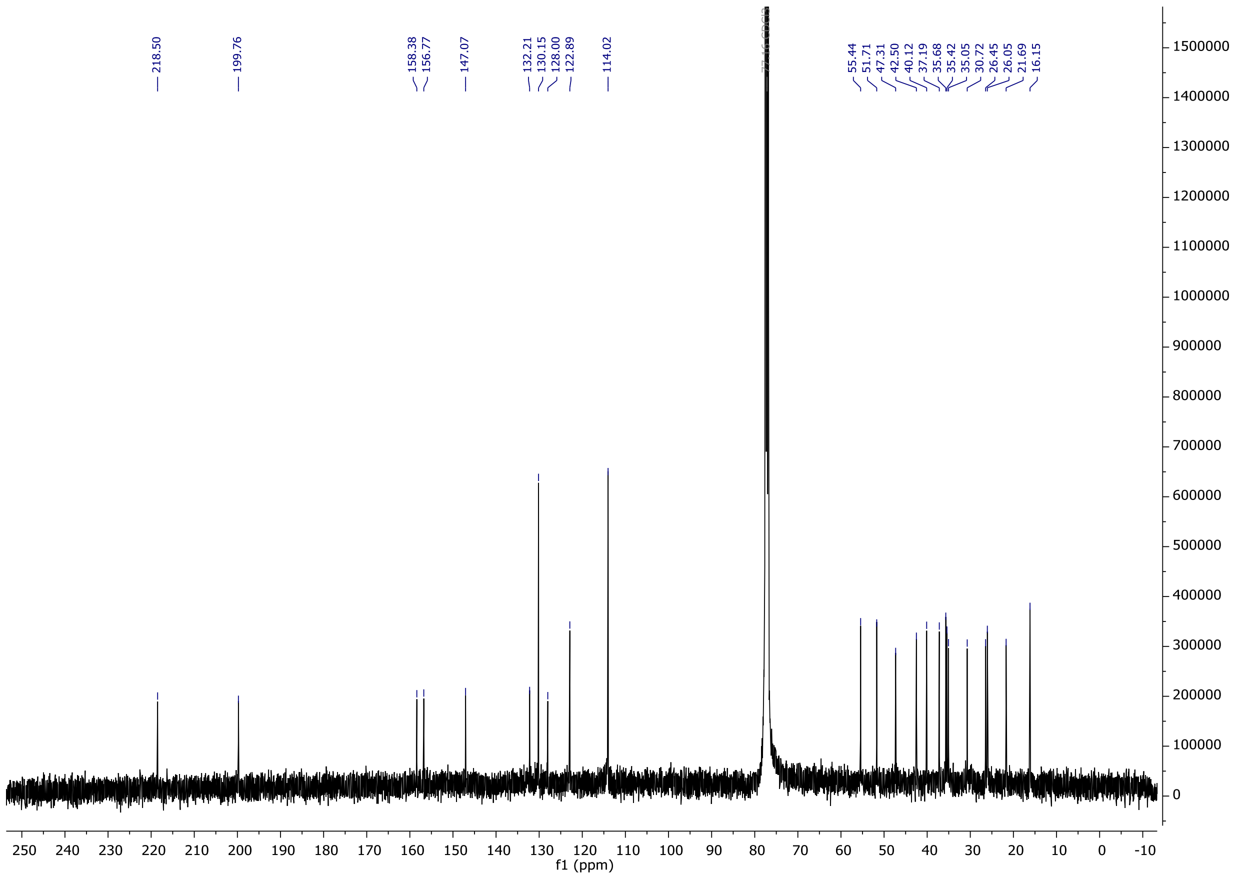

^{1}H NMR (400 MHz, CDCl₃) Compound **1e**

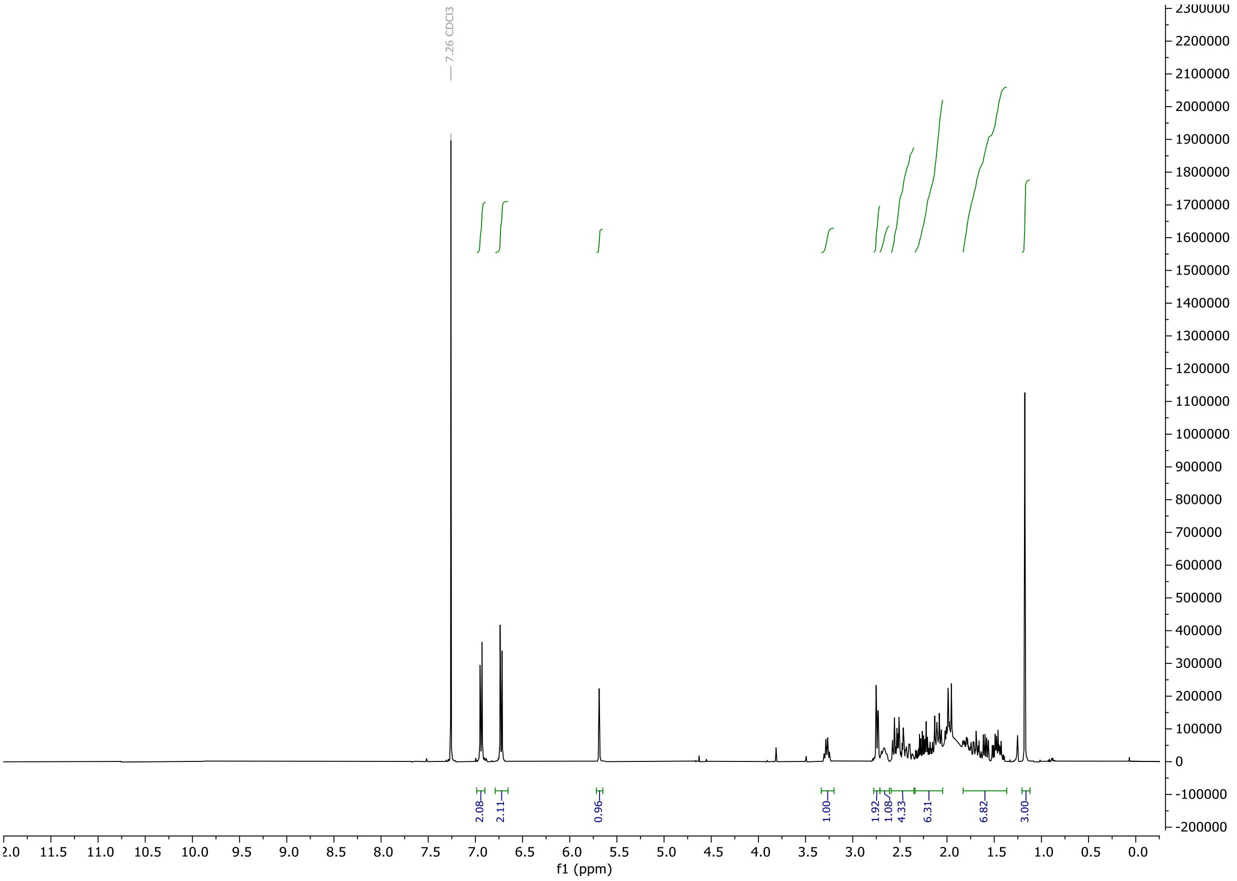

^{13}C NMR (CDCl₃, 100 MHz) Compound **1e**

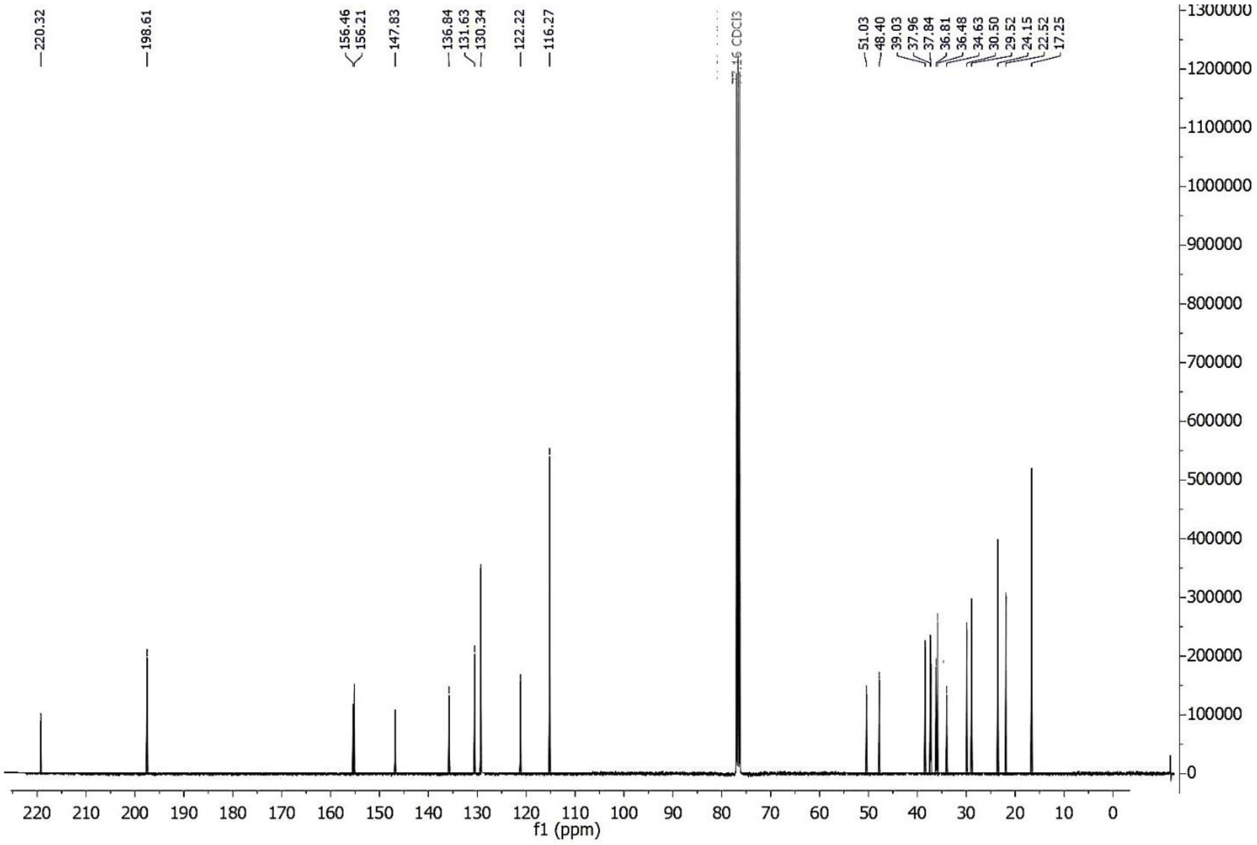

^{1}H NMR (400 MHz, $CDCl_3$) Compound **1f**

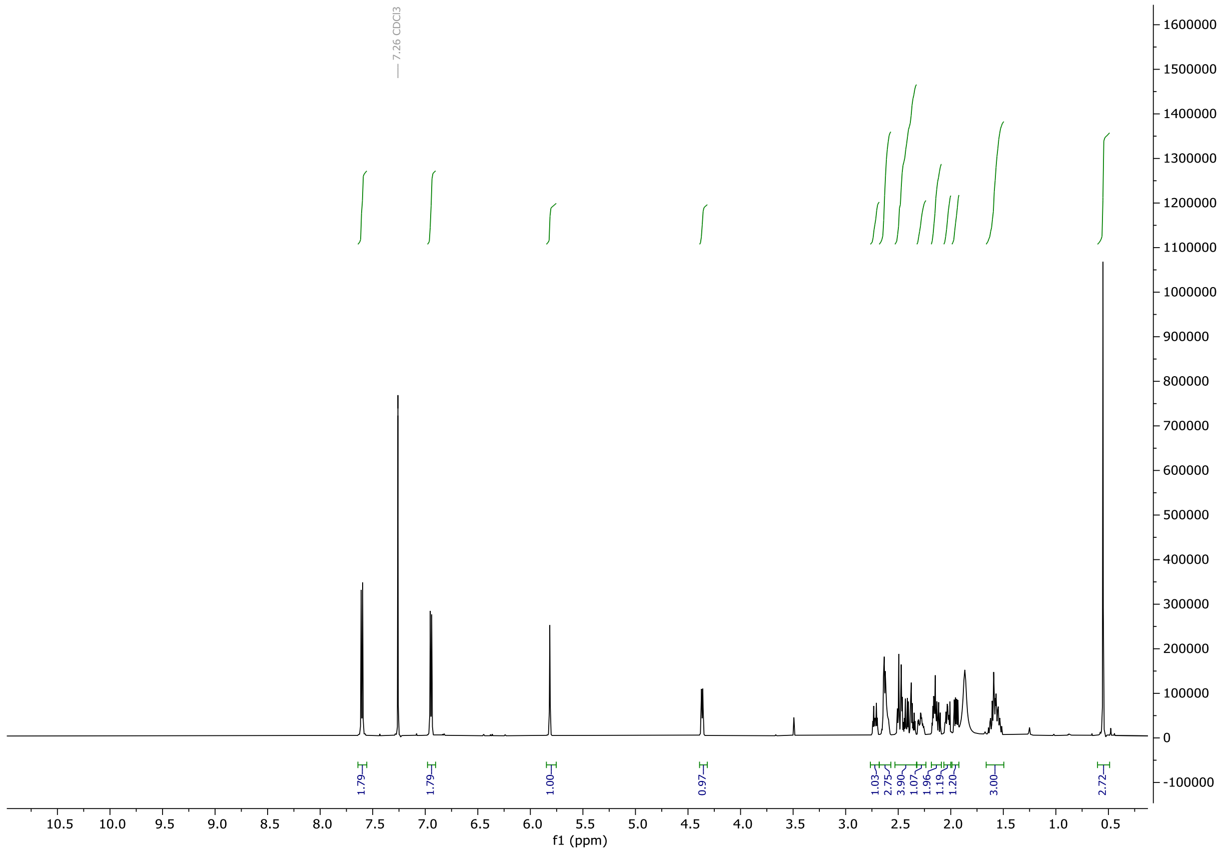

^{13}C NMR (100 MHz, $CDCl_3$) Compound **1f**

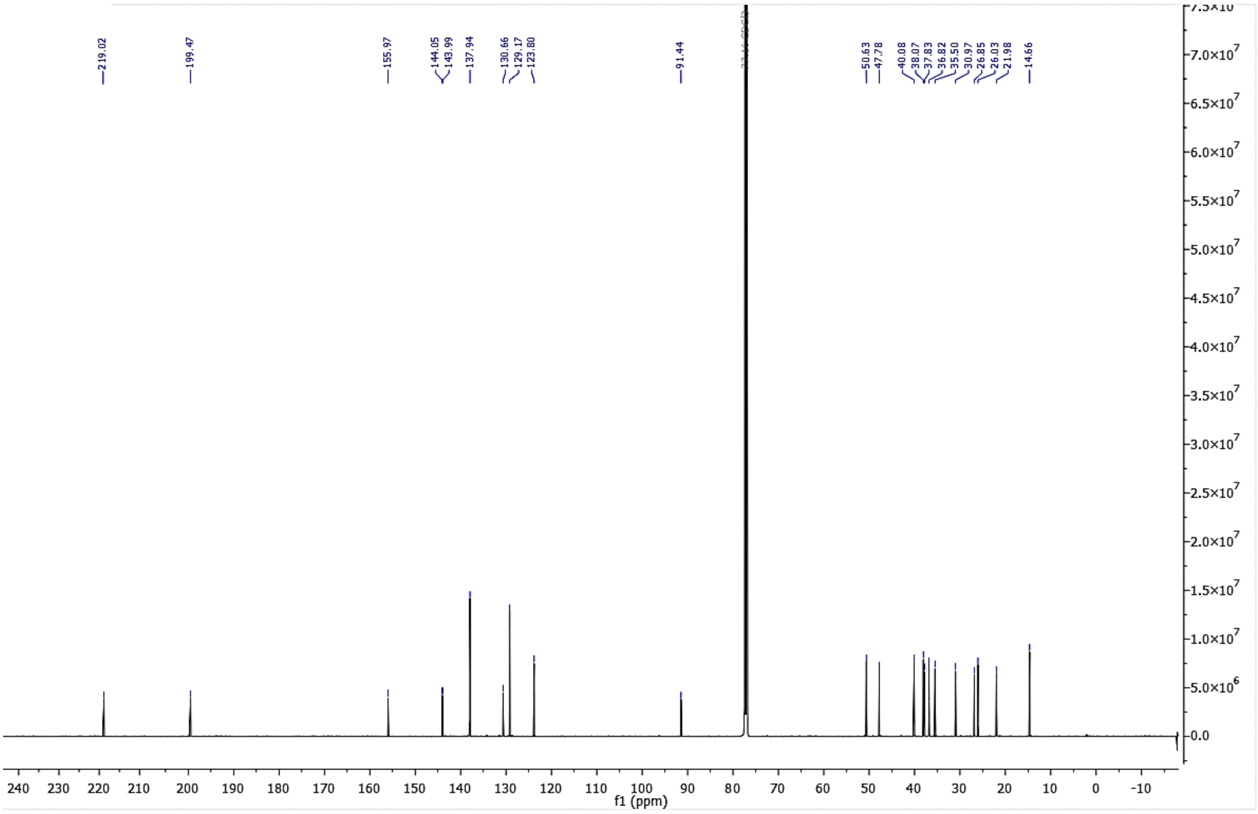

^{1}H NMR (400 MHz, CDCl$_3$) Compound **1g**

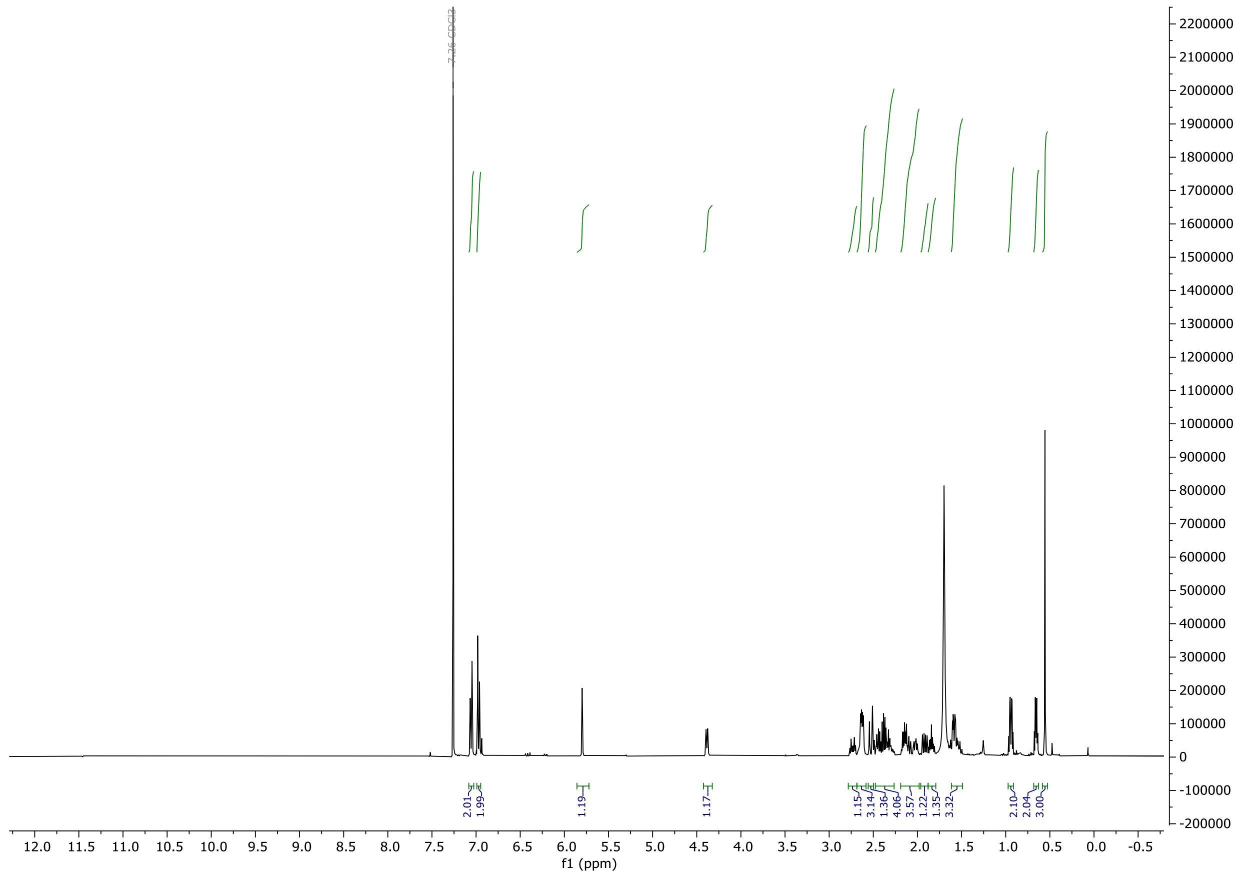

^{13}C NMR (100 MHz, CDCl$_3$) Compound **1g**

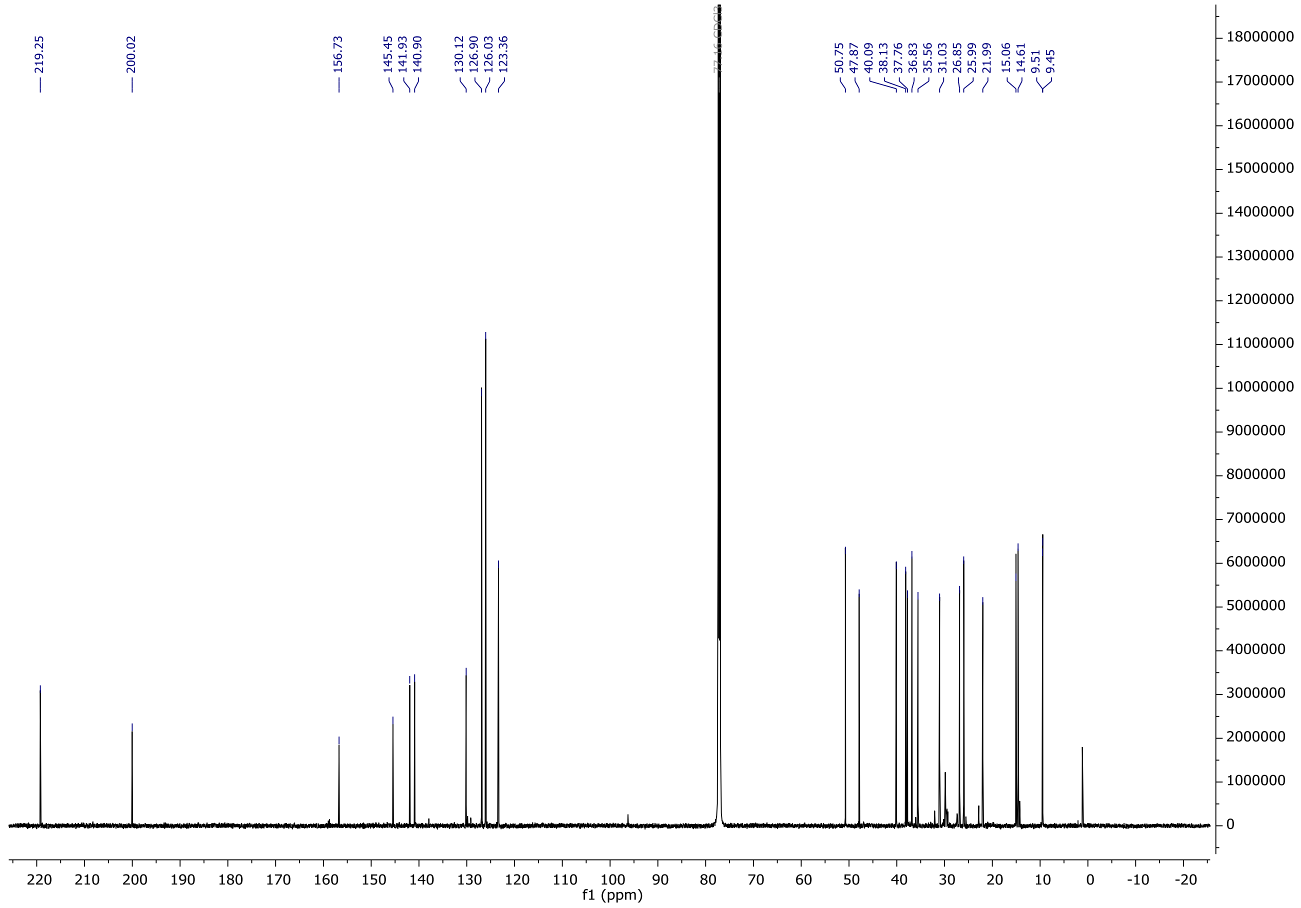

^{1}H NMR (400 MHz, $CDCl_3$) Compound **1h**

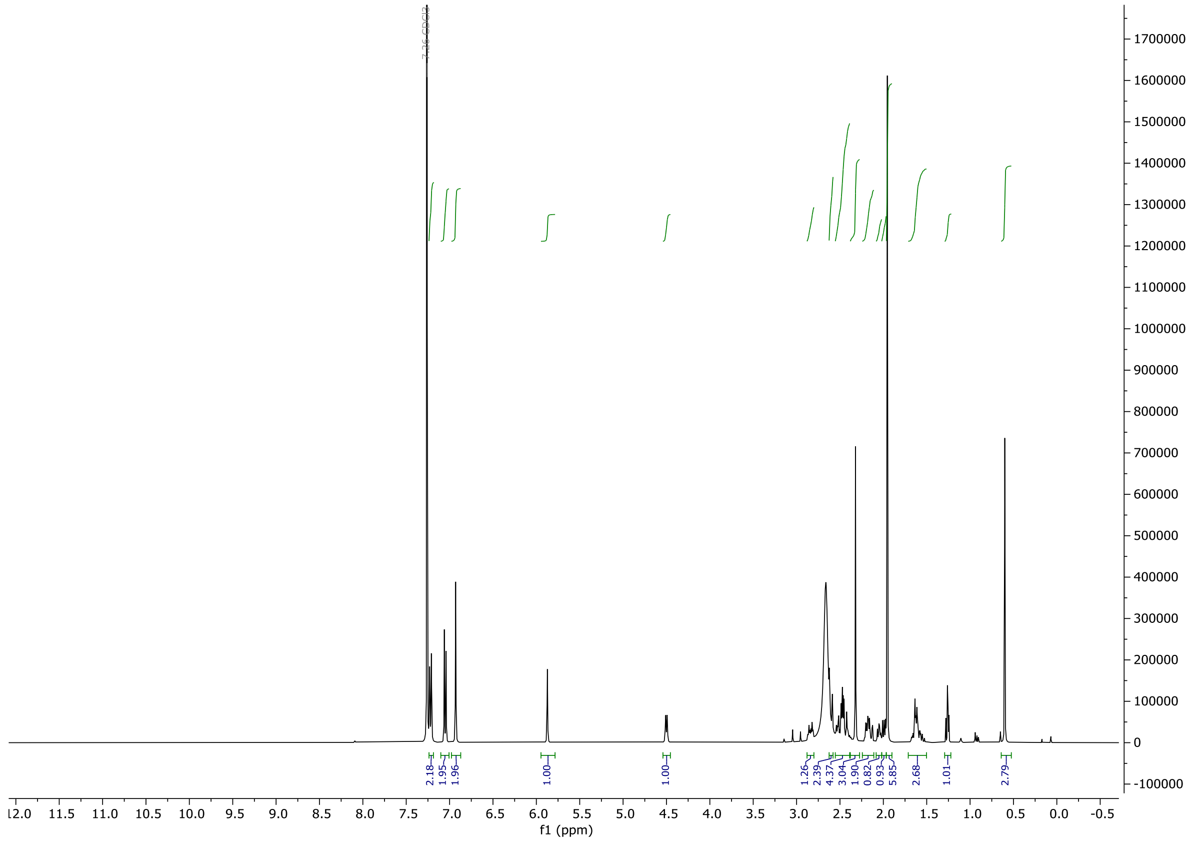

^{13}C NMR (100 MHz, $CDCl_3$) Compound **1h**

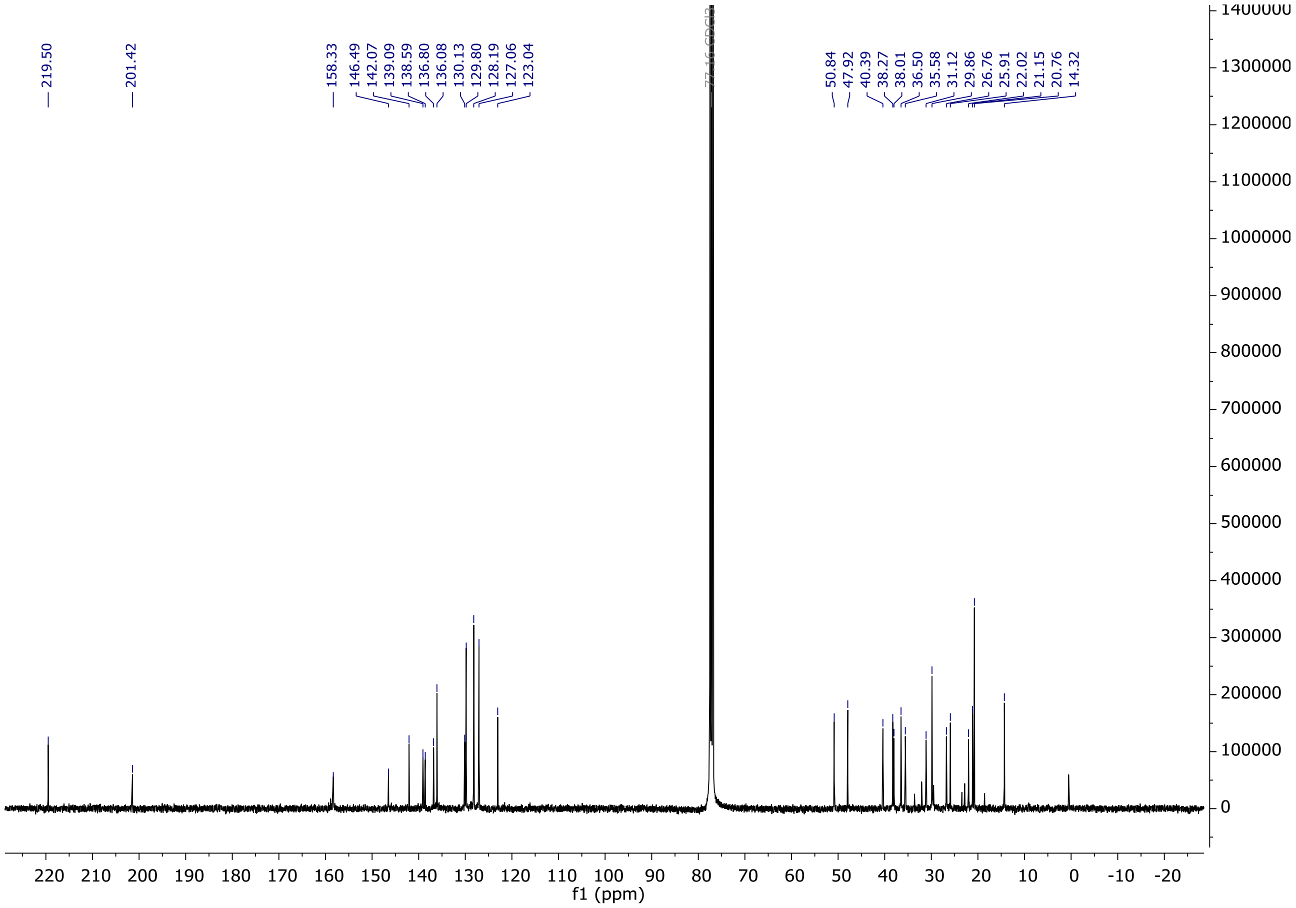

^{1}H NMR (400 MHz, CDCl$_3$) Compound **2a**

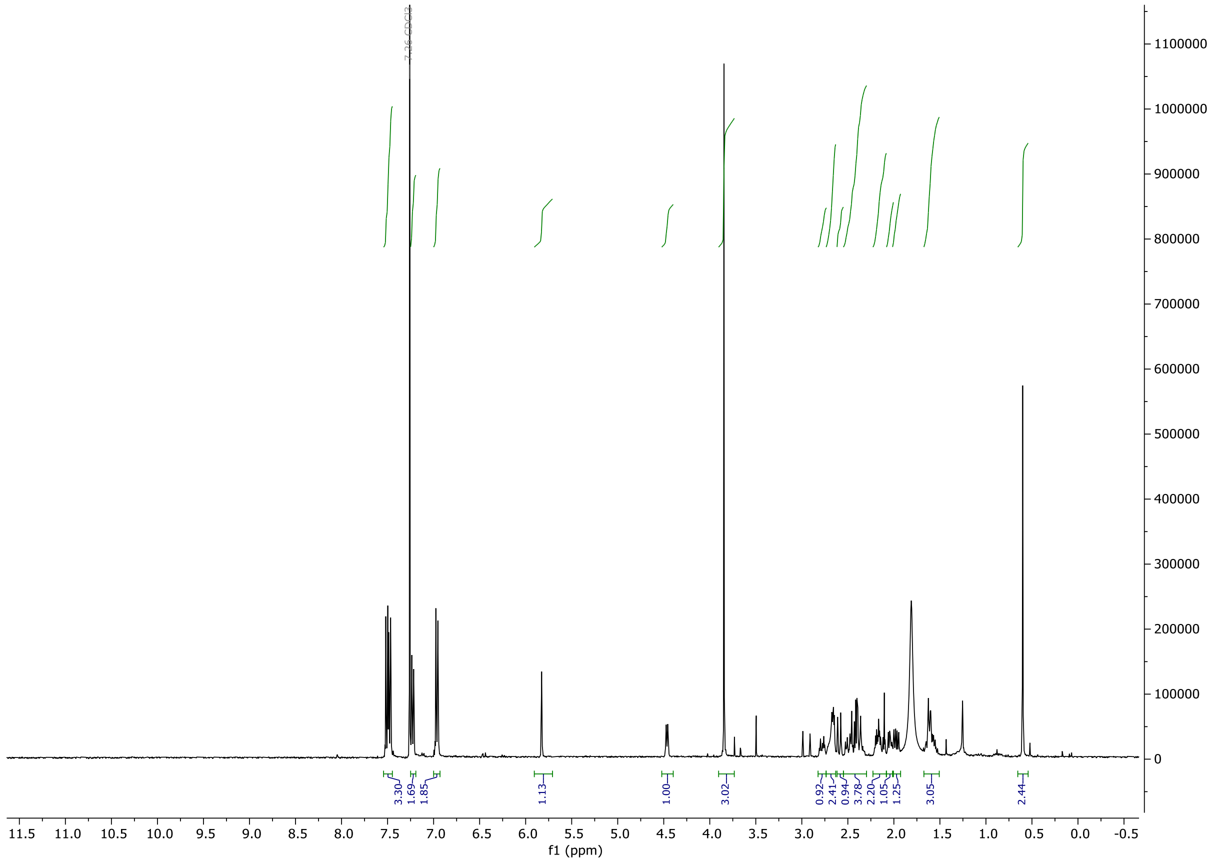

^{13}C NMR (100 MHz, CDCl$_3$) Compound **2a**

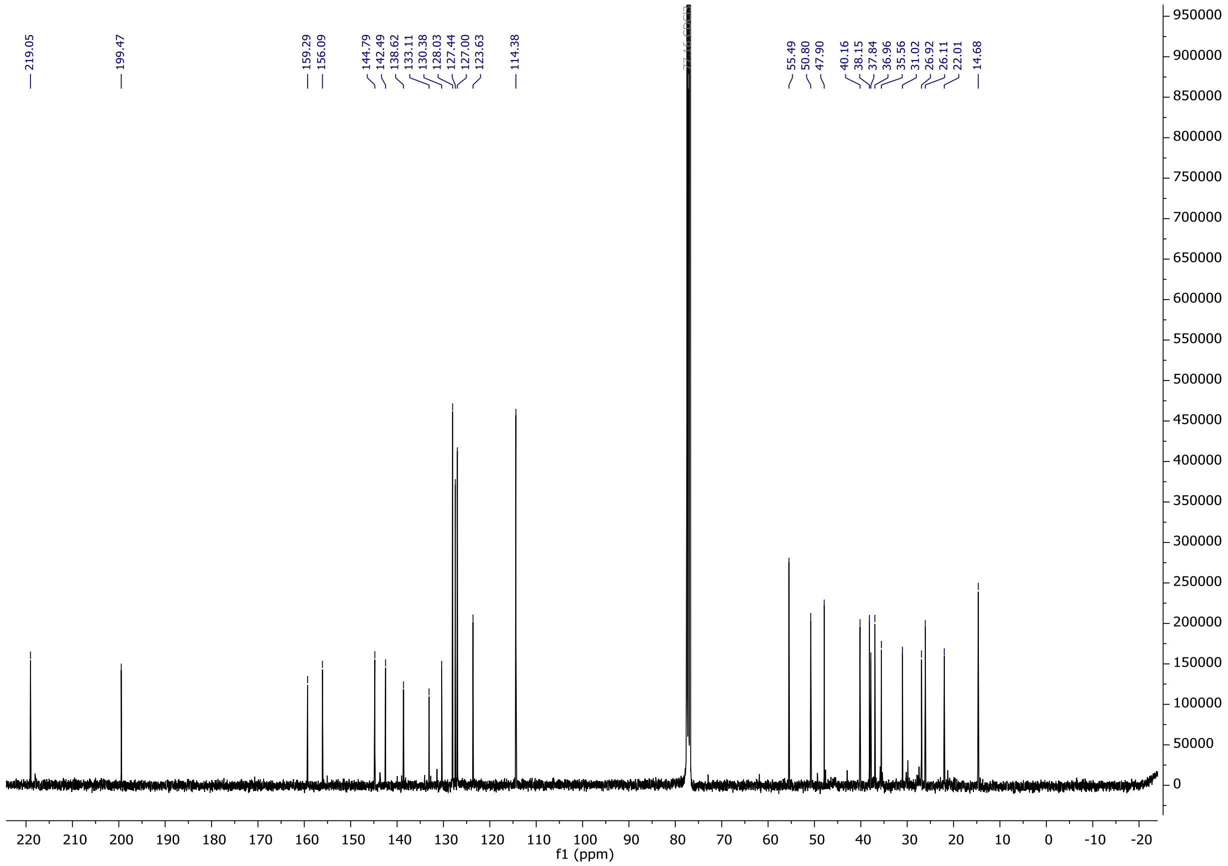

^{1}H NMR (400 MHz, CDCl$_3$) Compound **2b**

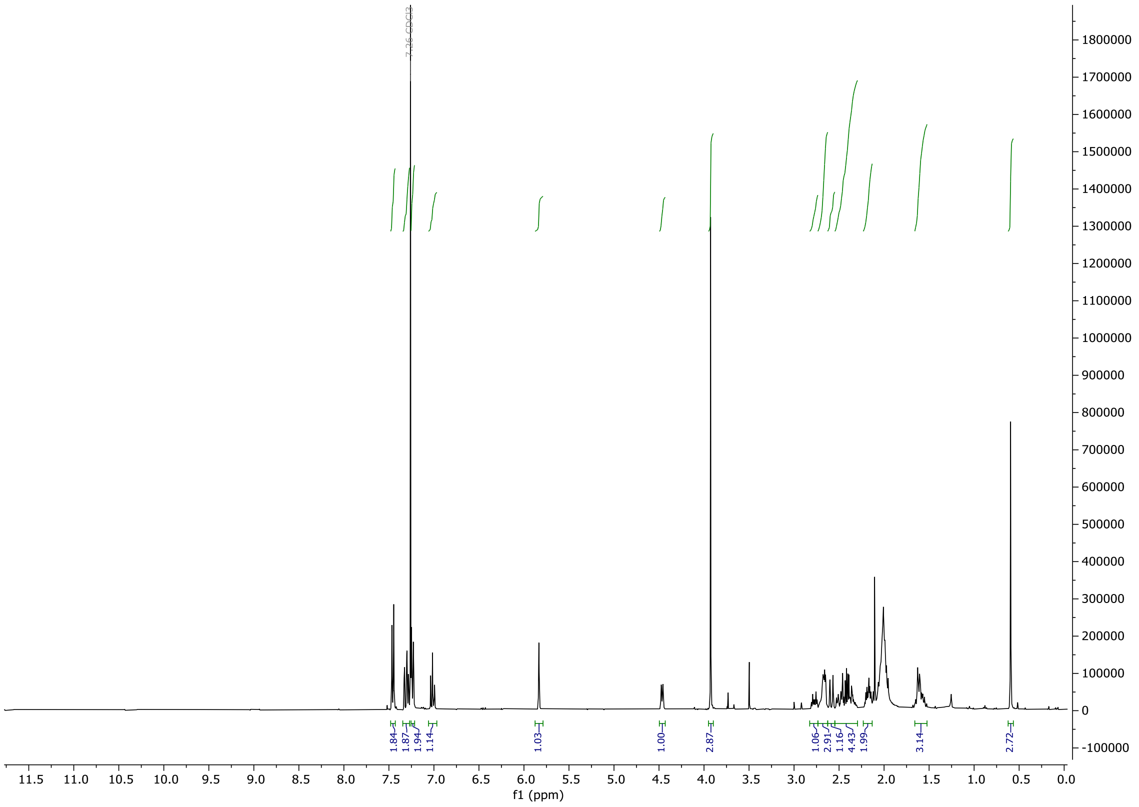

^{13}C NMR (100 MHz, CDCl$_3$) Compound **2b**

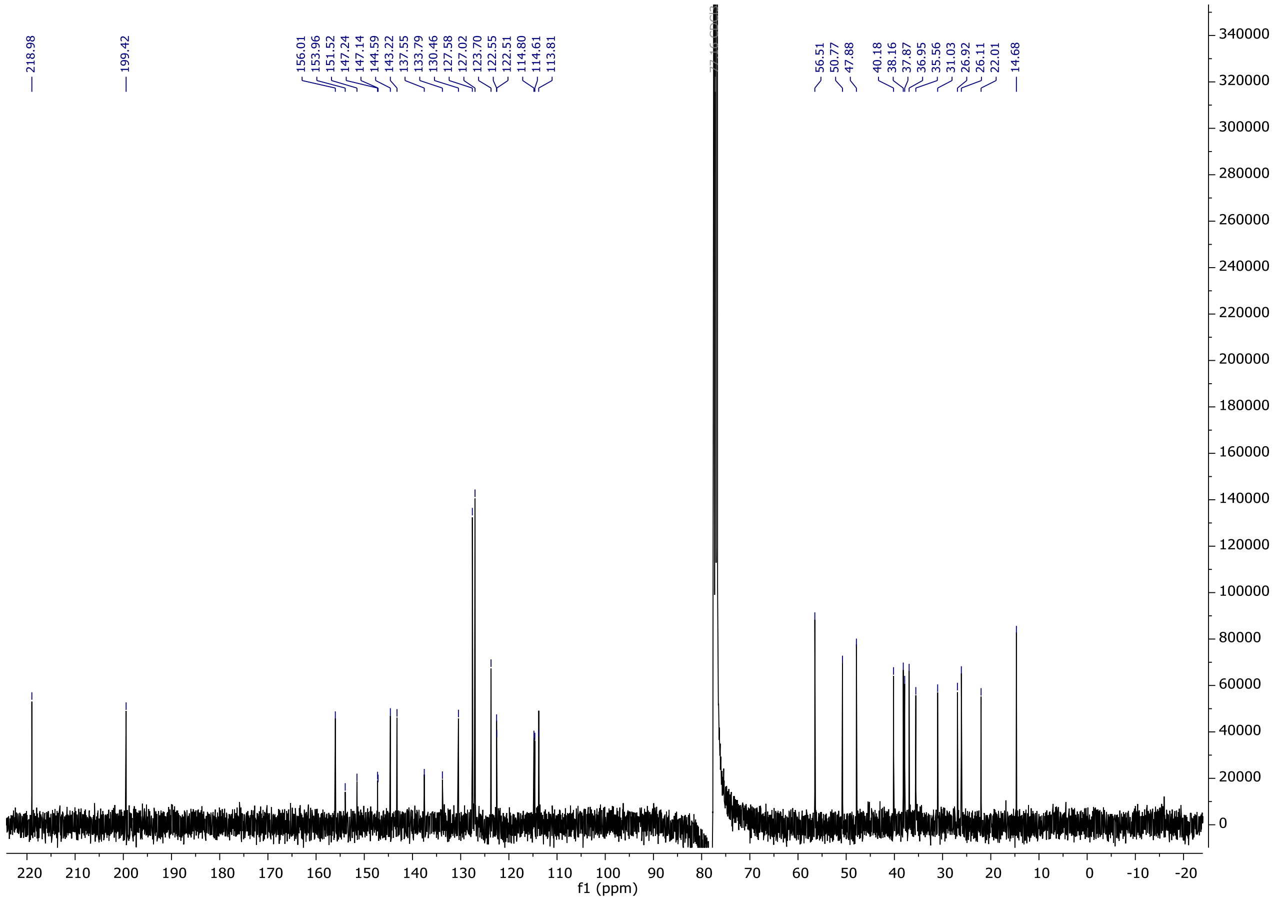

^{19}F NMR (376 MHz, $CDCl_3$) Compound **2b**

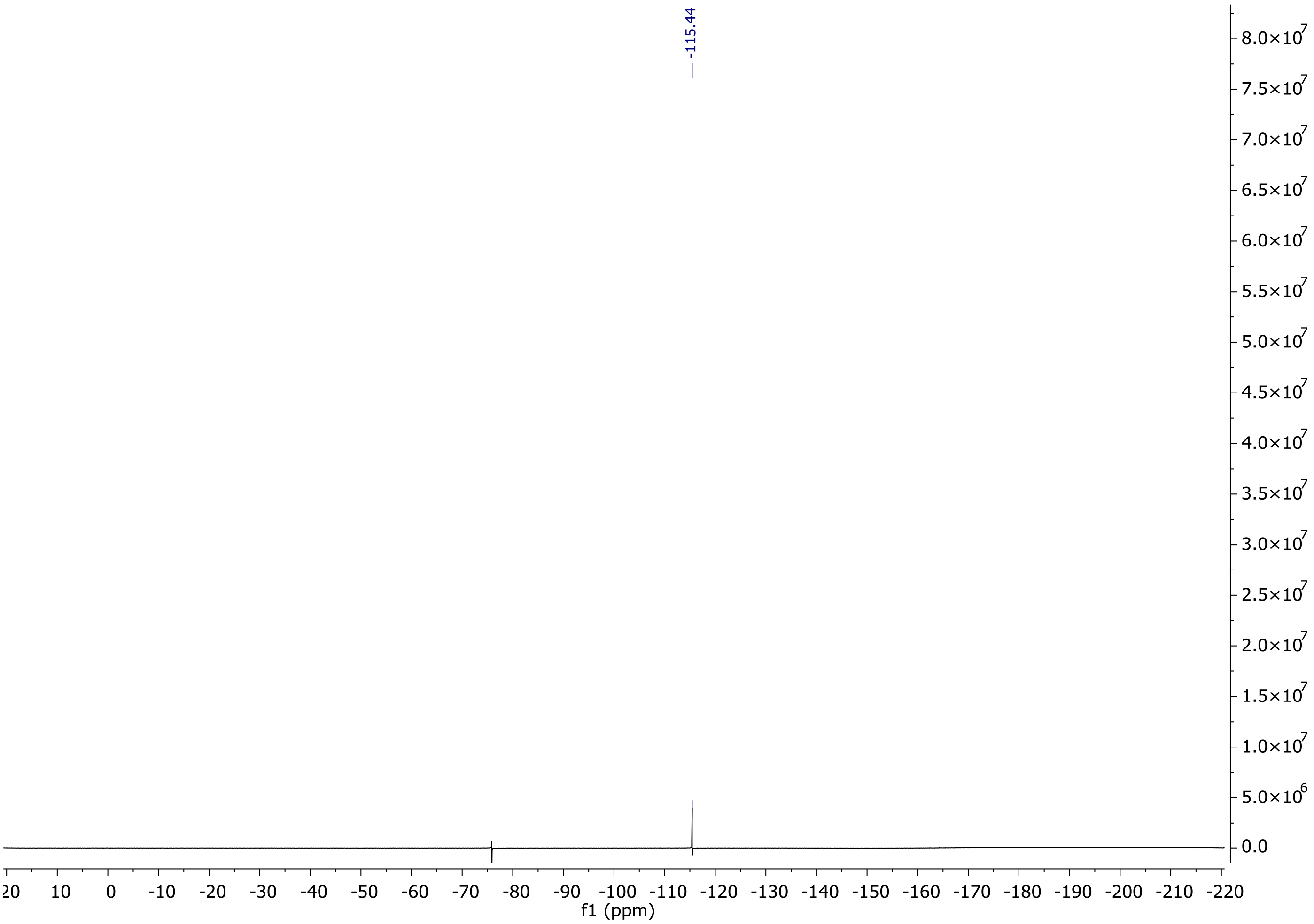

^{1}H NMR (400 MHz, $CDCl_3$) Compound **2c**

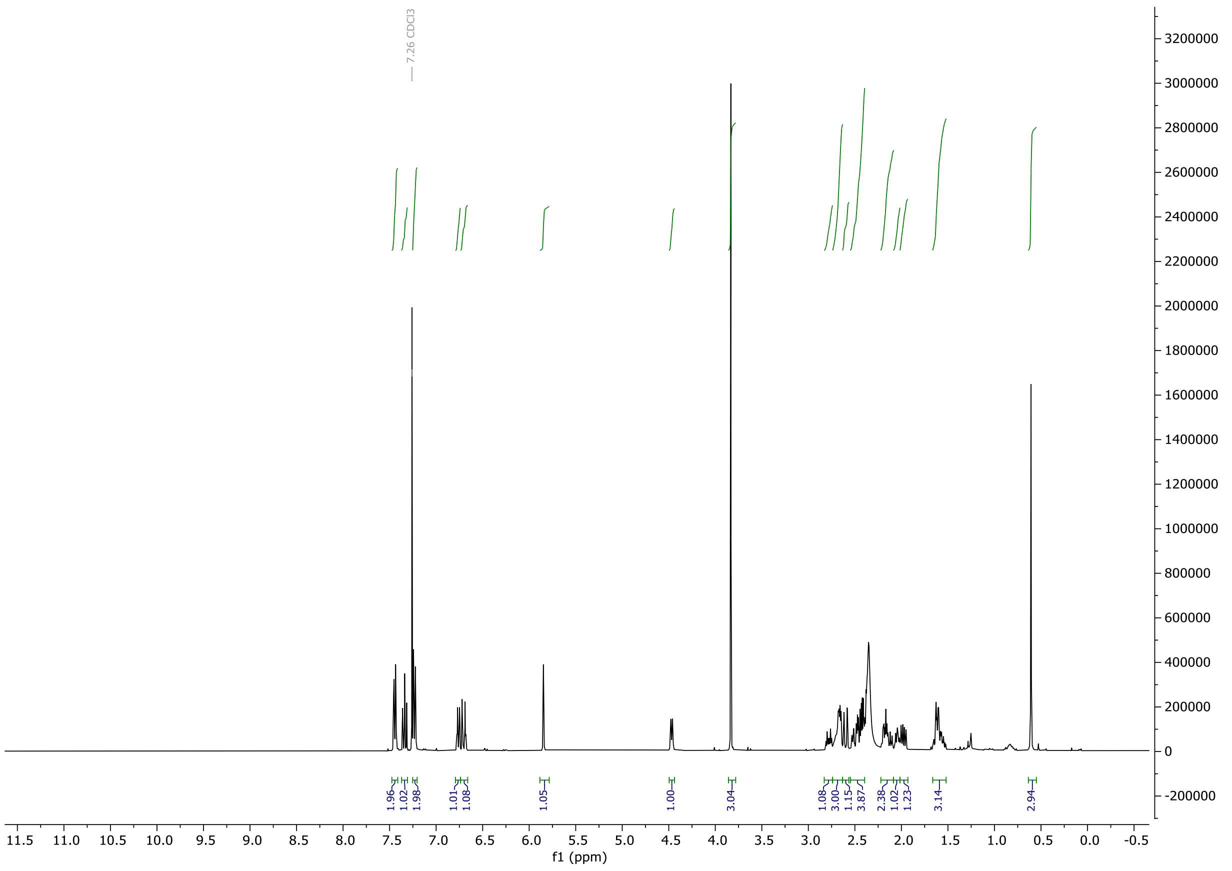

^{13}C NMR (100 MHz, $CDCl_3$) Compound **2c**

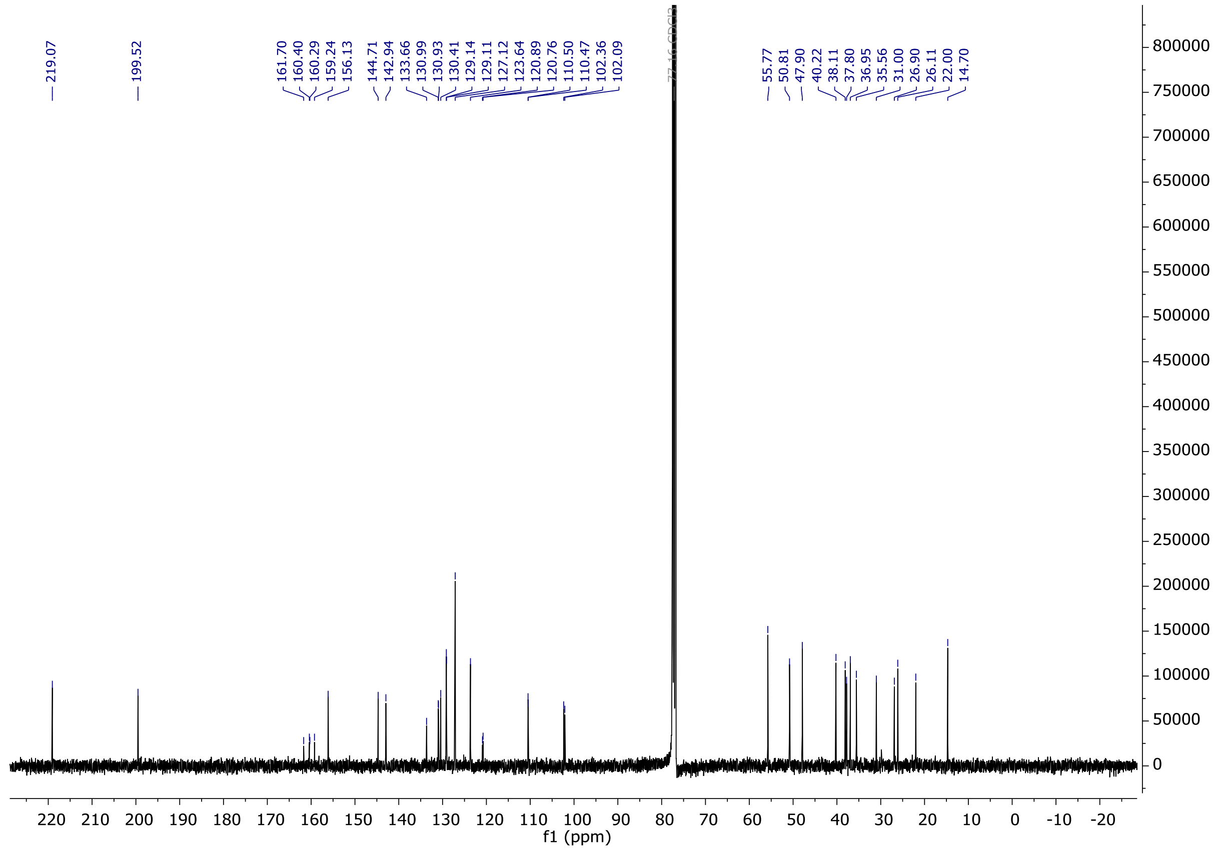

^{19}F NMR (376 MHz, CDCl$_3$) Compound **2c**

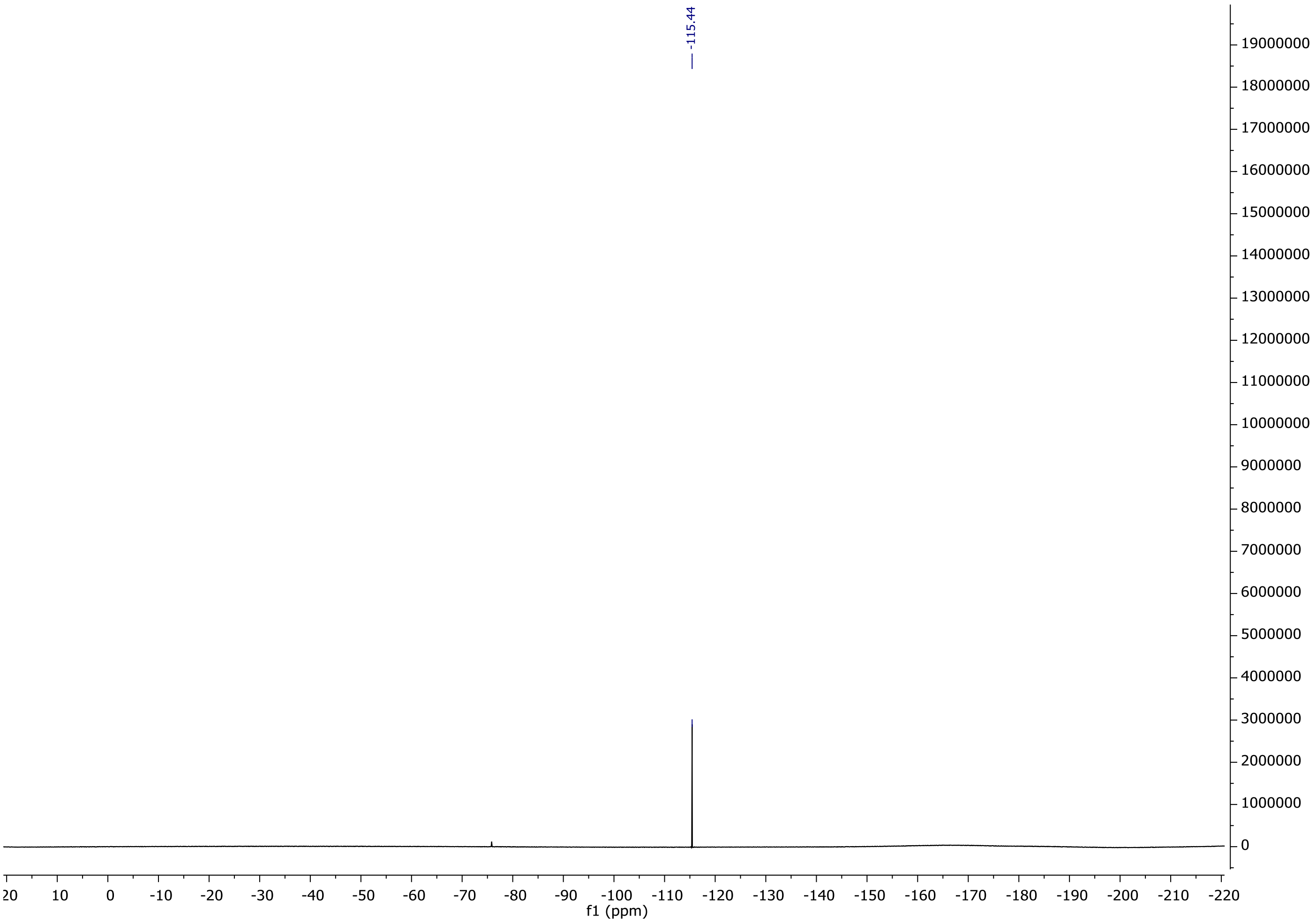

^{1}H NMR (400 MHz, $CDCl_3$) Compound **2d**

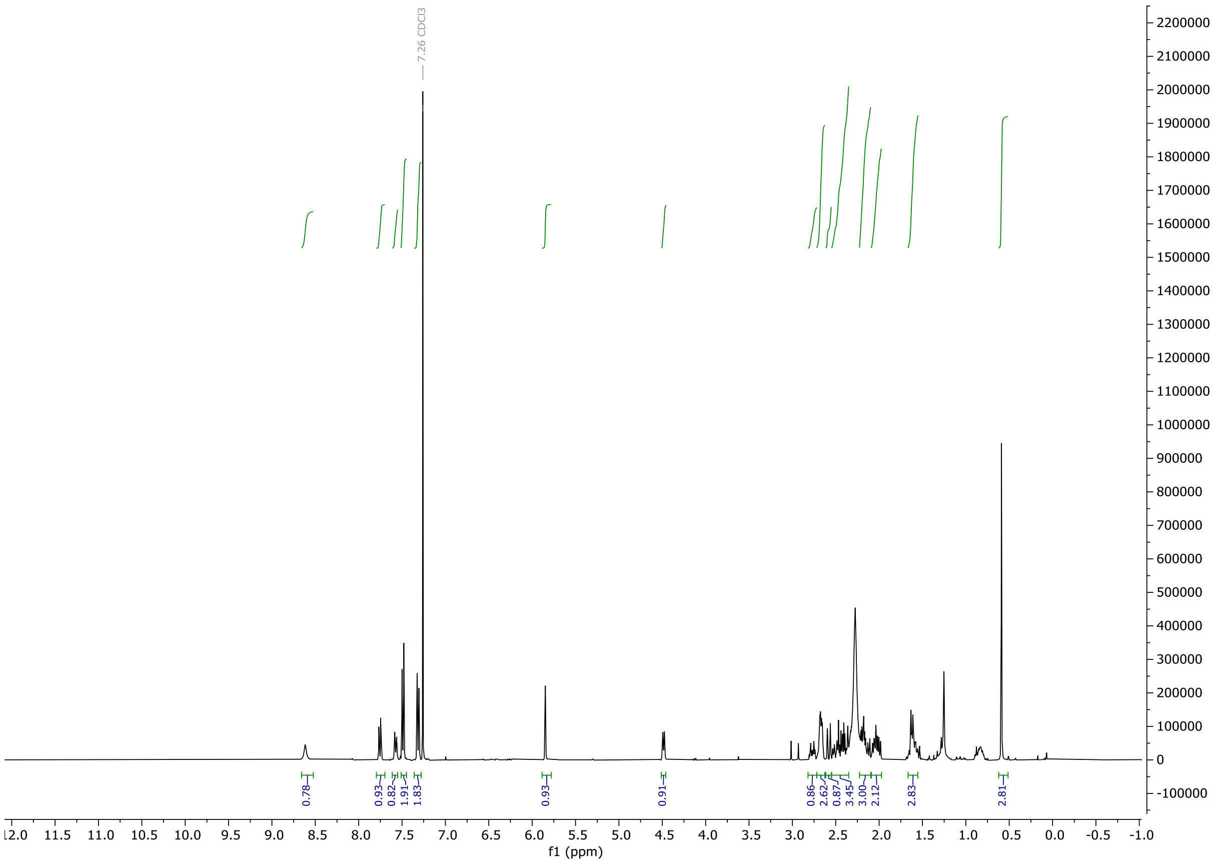

^{13}C NMR (100 MHz, $CDCl_3$) Compound **2d**

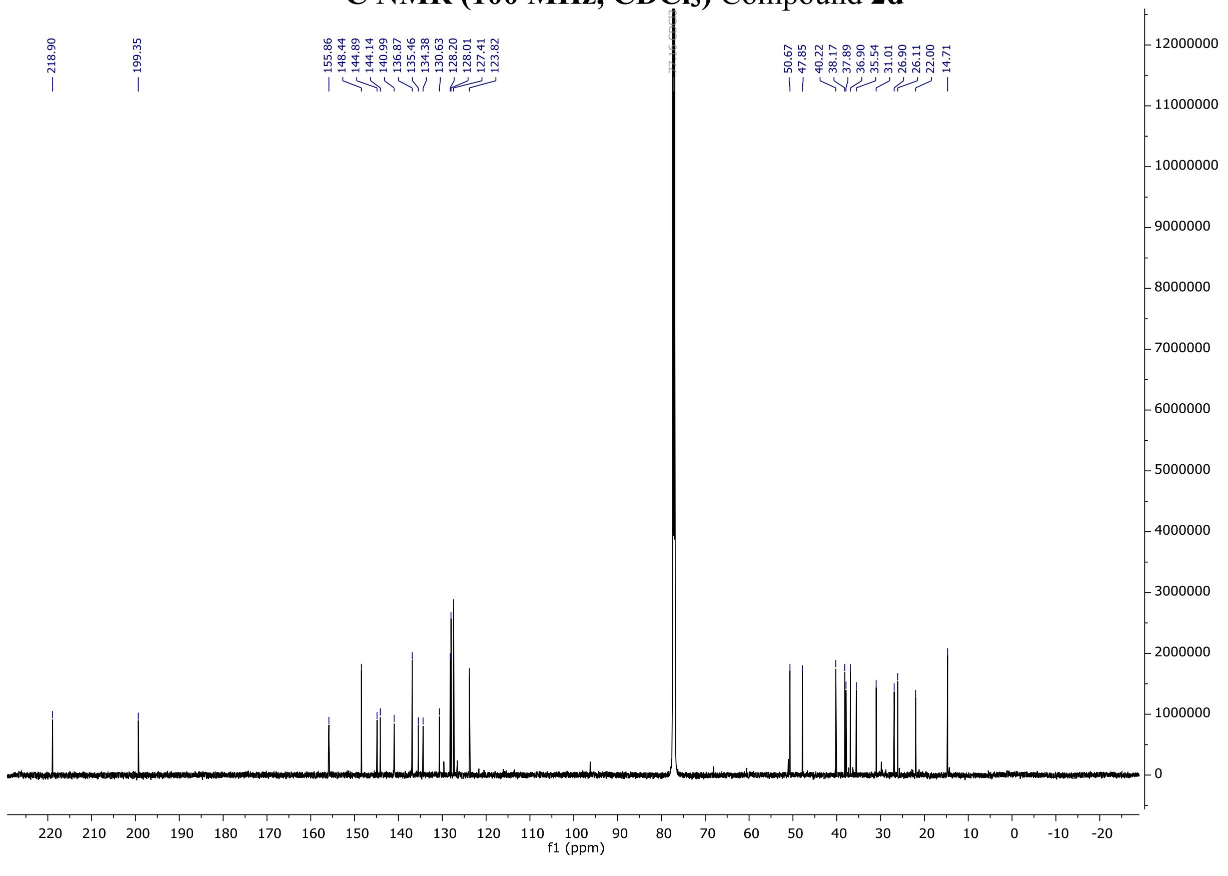

^{1}H NMR (400 MHz, $CDCl_3$) Compound 2e

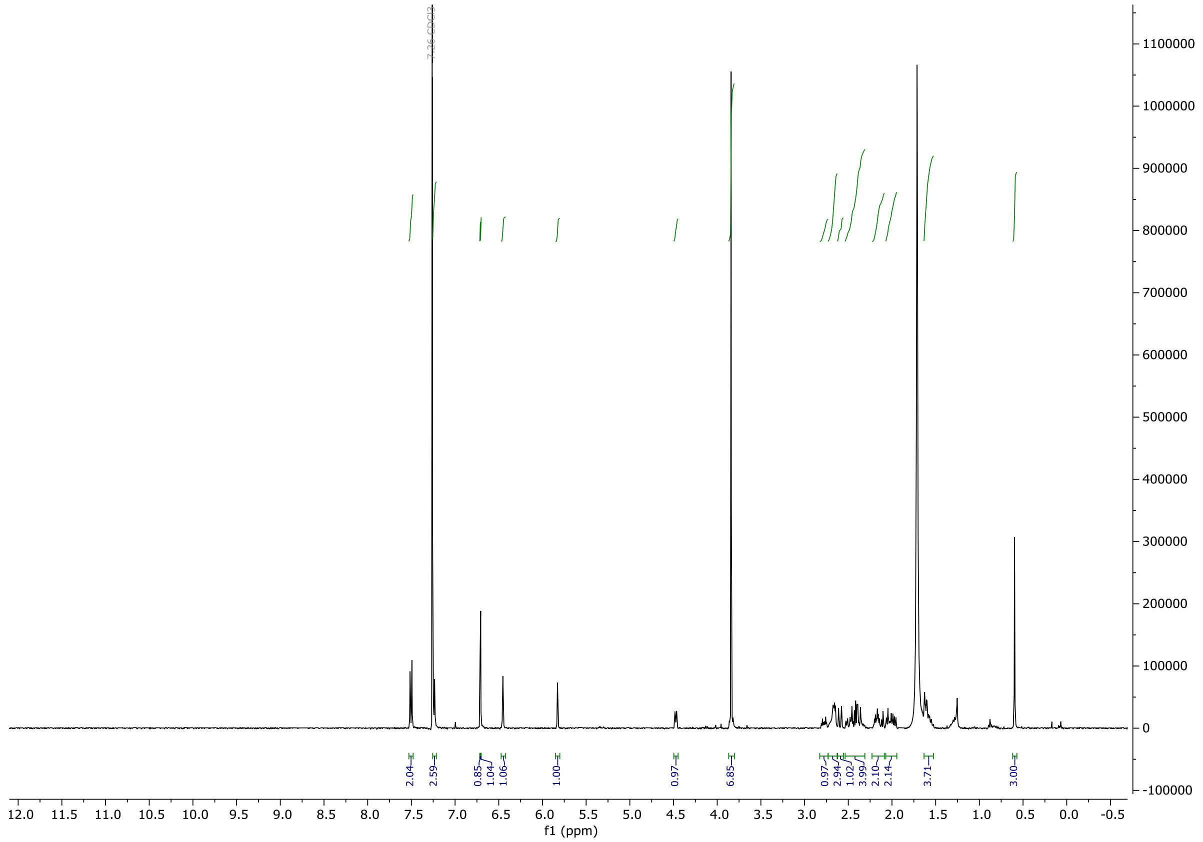

^{13}C NMR (100 MHz, $CDCl_3$) Compound 2e

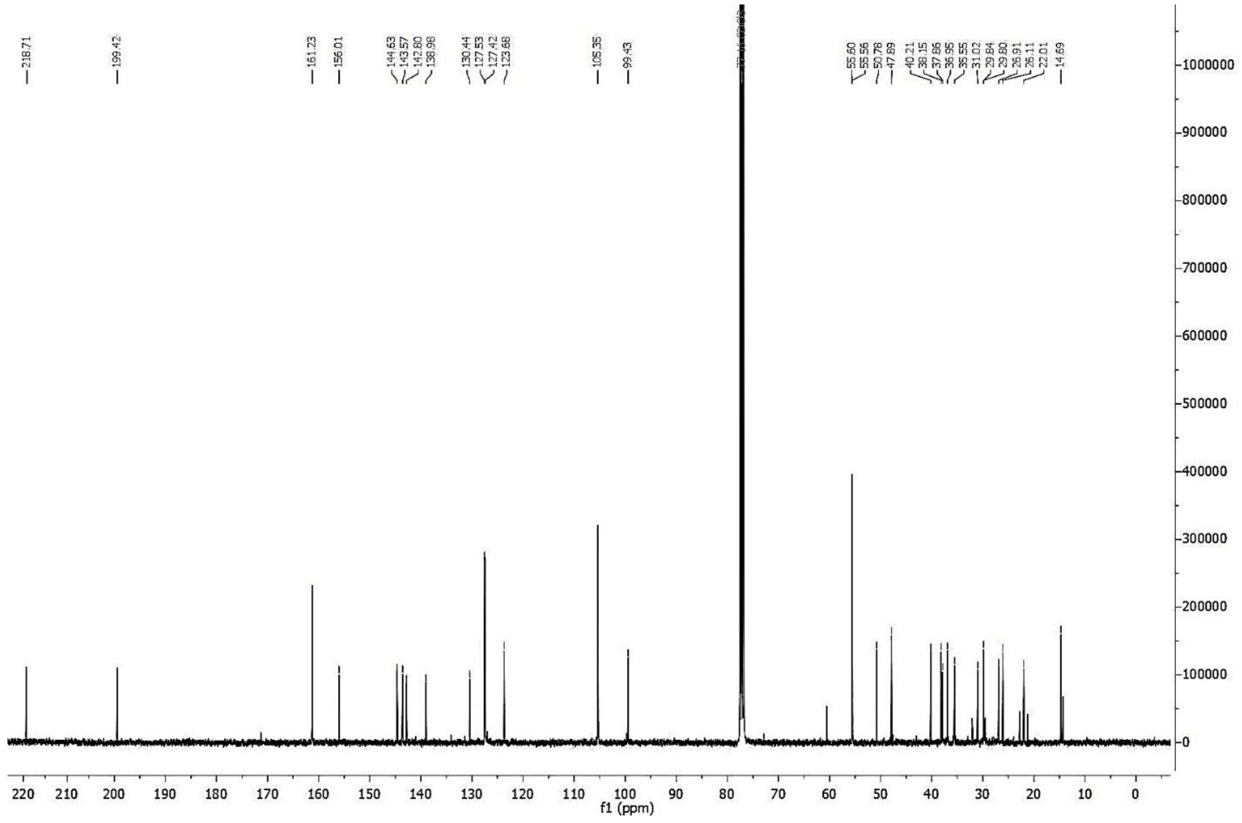

^{1}H NMR (400 MHz, $CDCl_3$) Compound **2f**

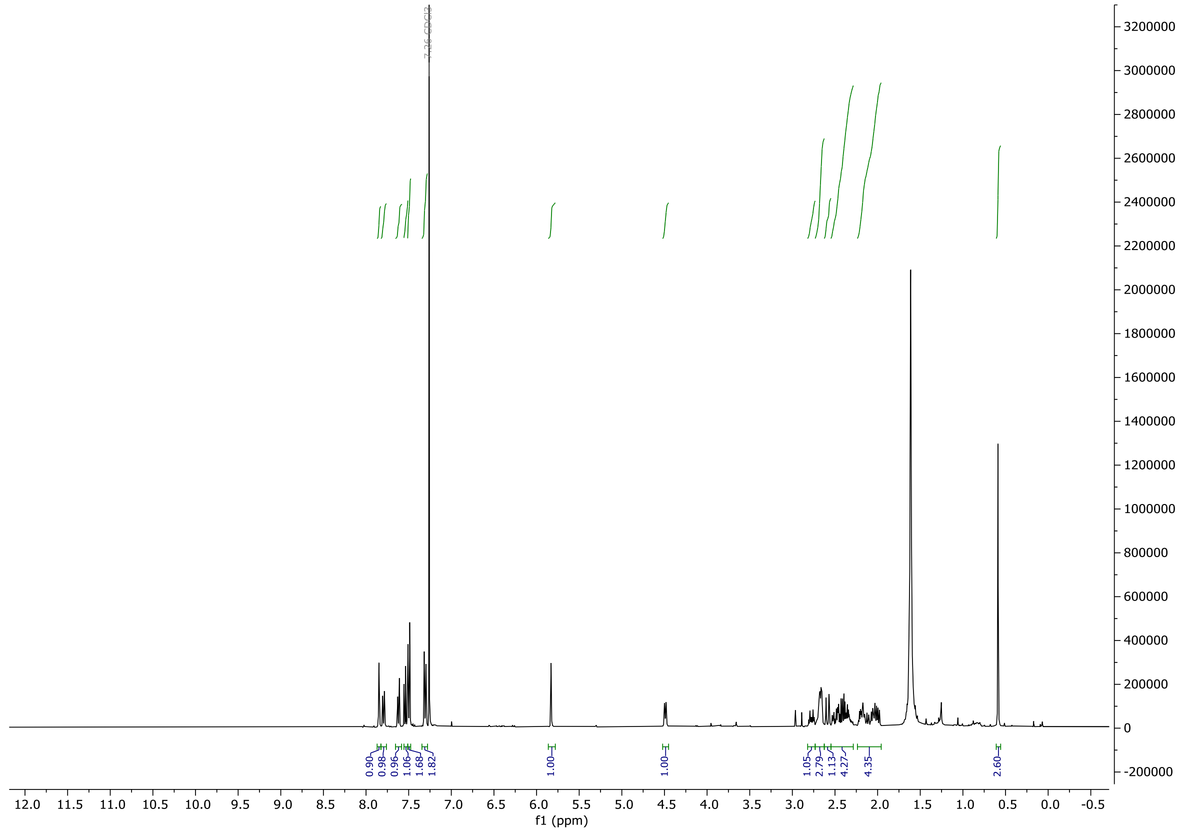

^{13}C NMR (100 MHz, $CDCl_3$) Compound **2f**

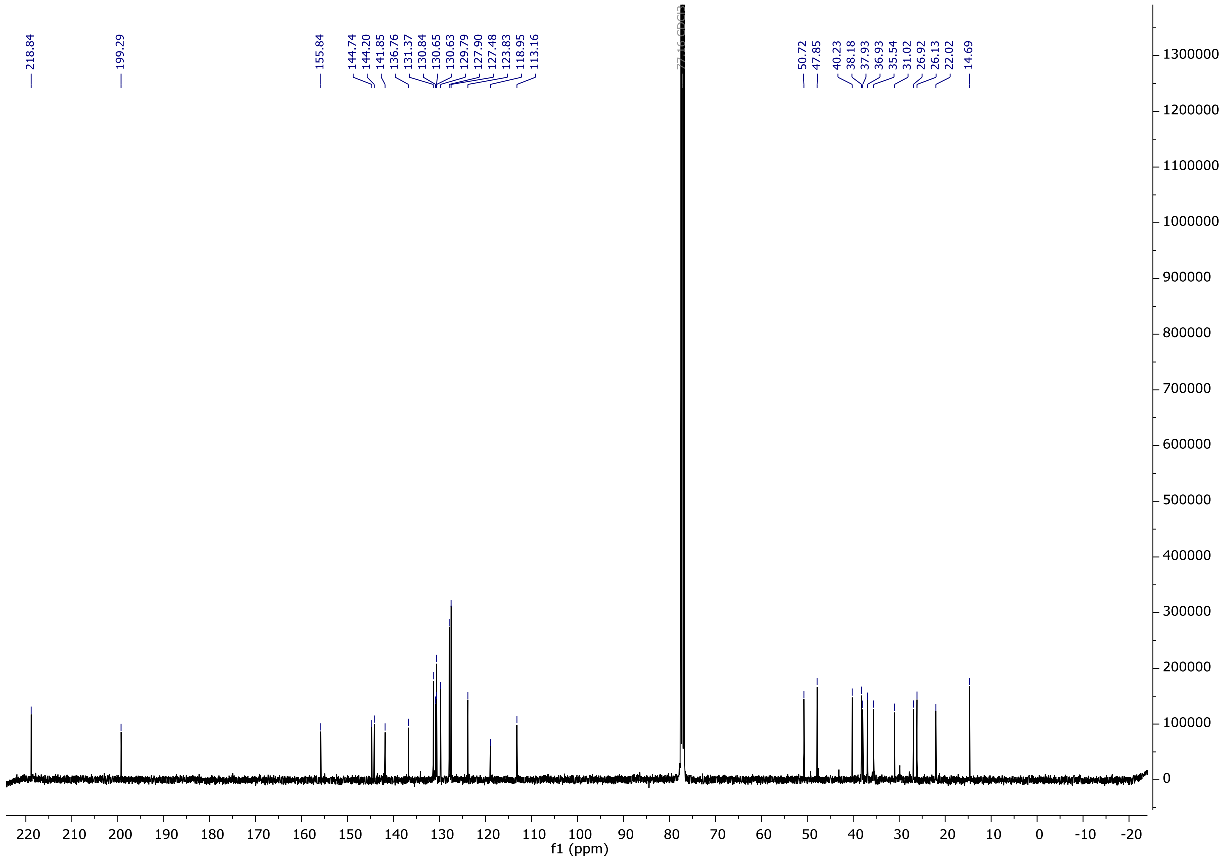

^{1}H NMR (400 MHz, $CDCl_3$) Compound 2g

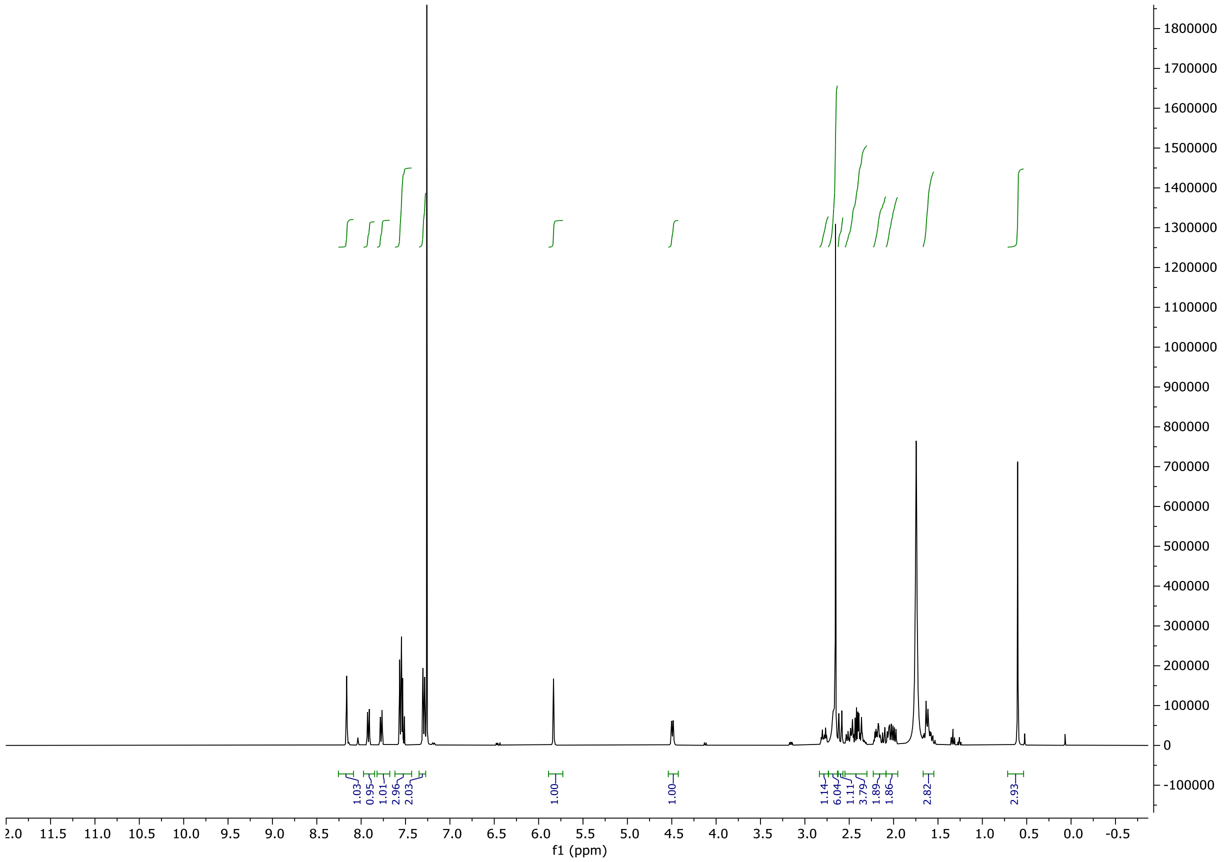

^{13}C NMR (100 MHz, $CDCl_3$) Compound 2g

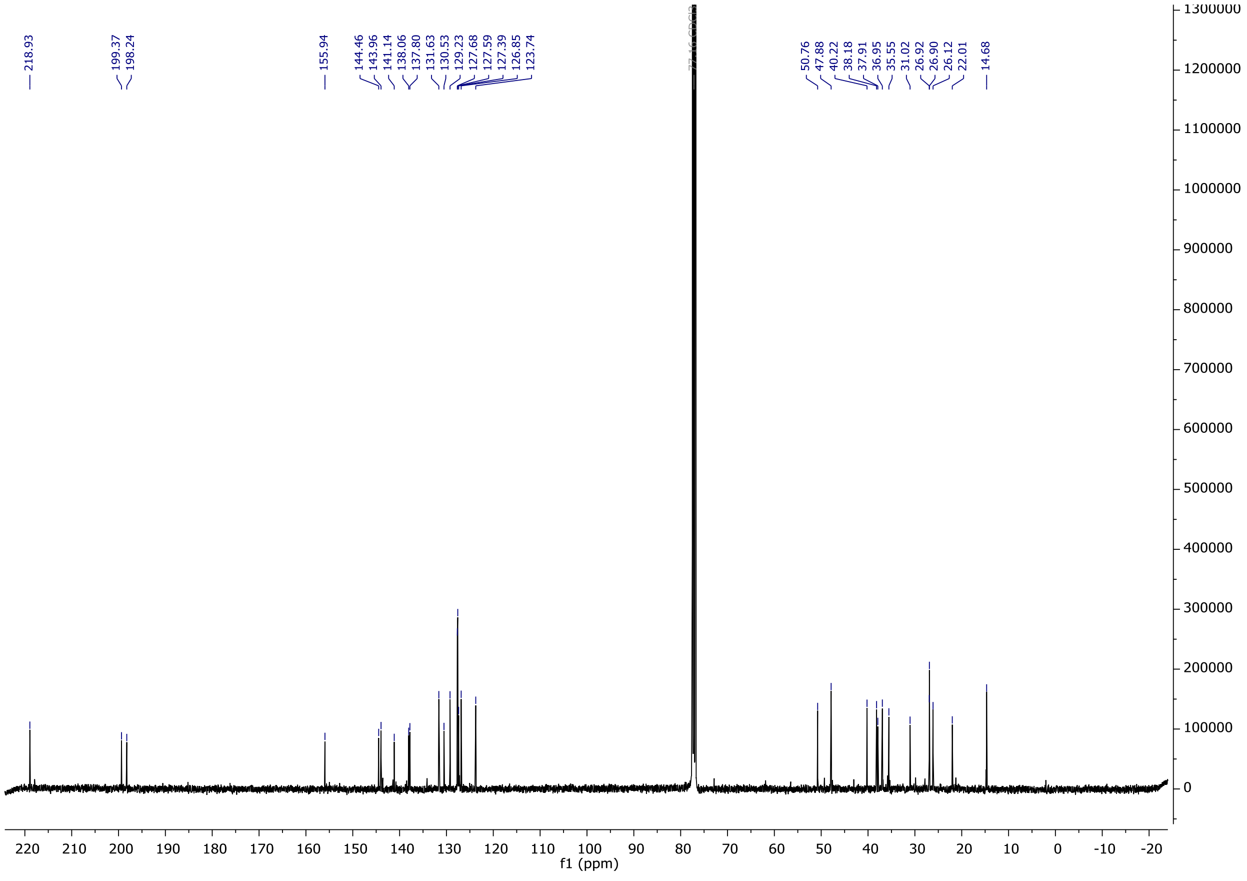

^{1}H NMR (400 MHz, $CDCl_3$) Compound **2h**

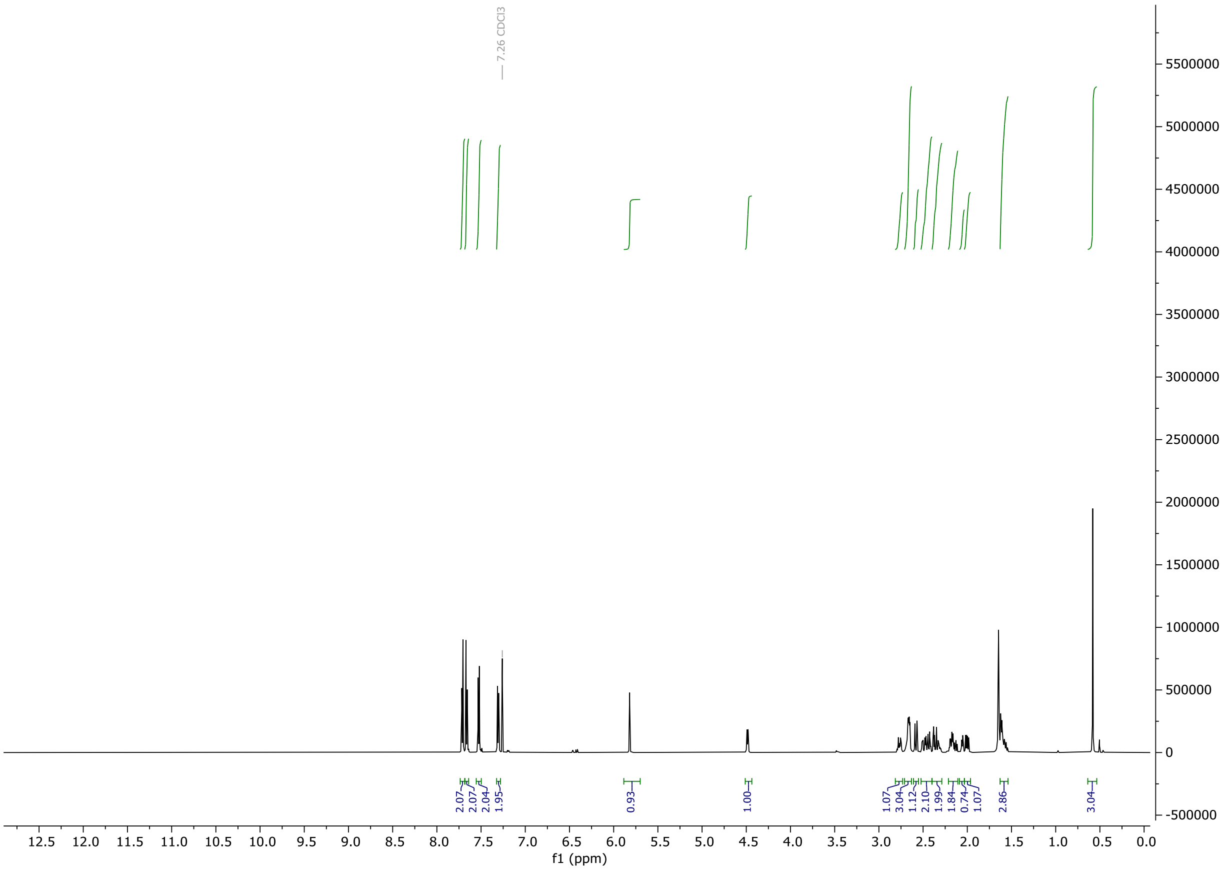

^{13}C NMR (100 MHz, $CDCl_3$) Compound **2h**

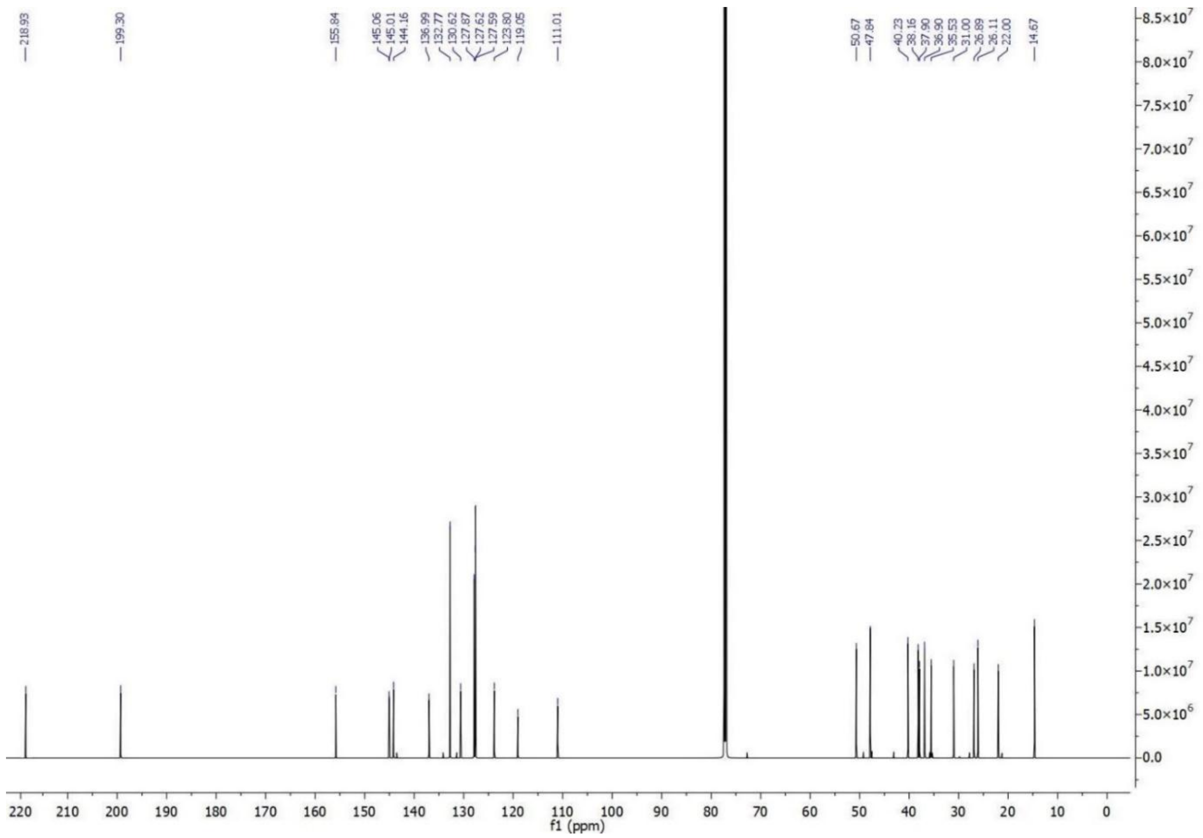

^{1}H NMR (400 MHz, CDCl$_3$) Compound **2i**

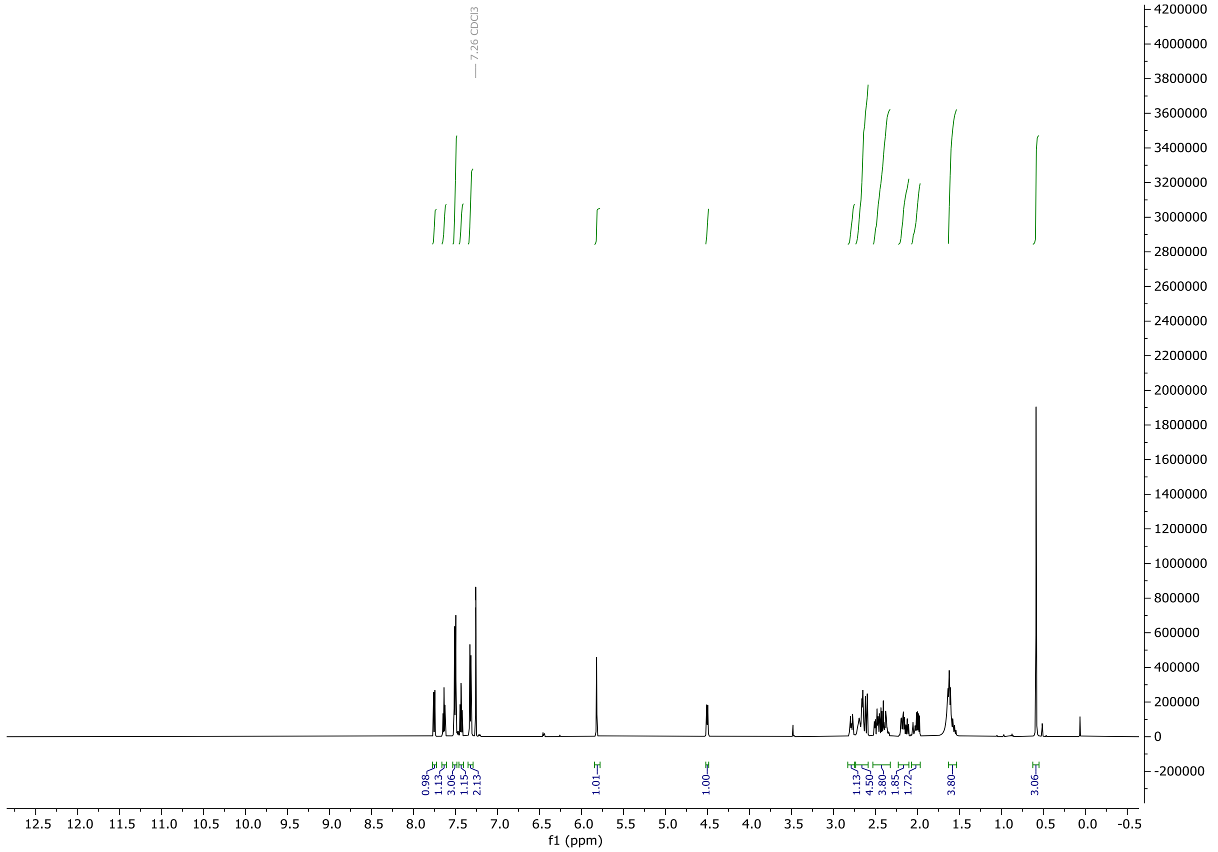

^{13}C NMR (100 MHz, CDCl$_3$) Compound **2i**

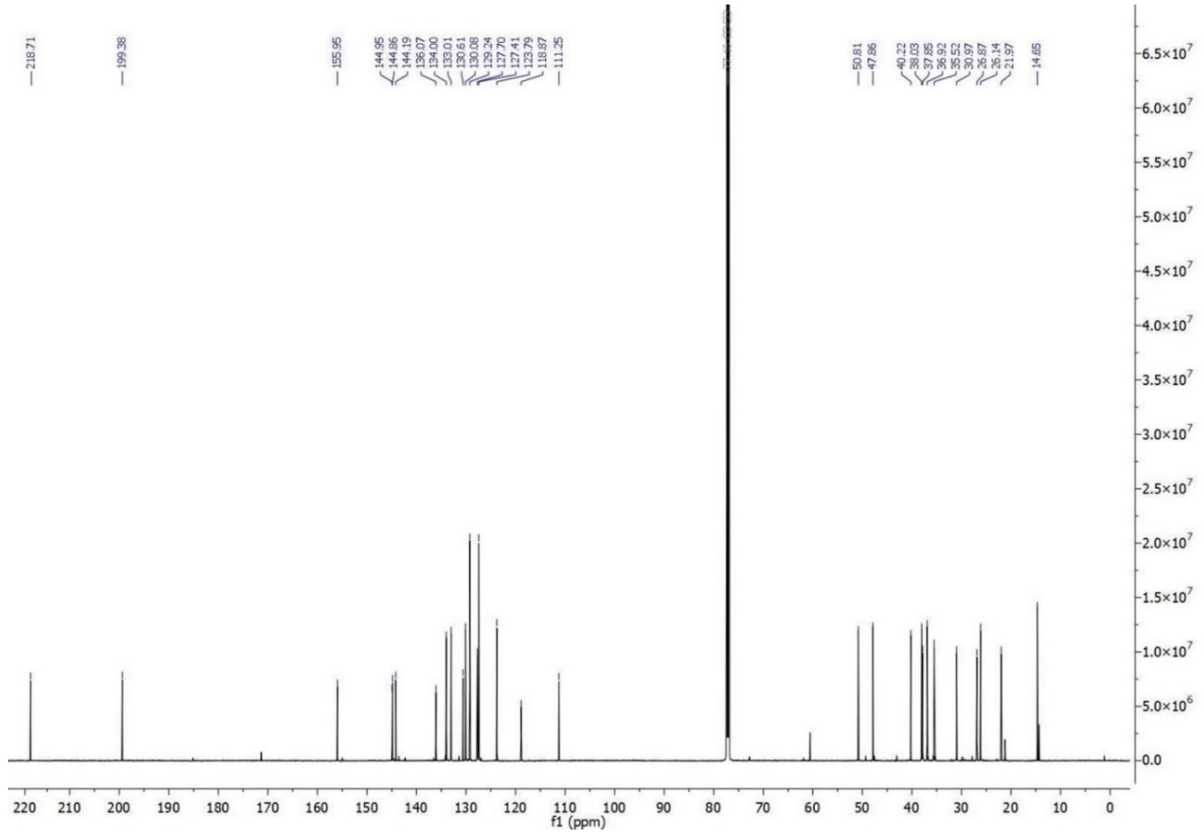

^{1}H NMR (400 MHz, $CDCl_3$) Compound **2j**

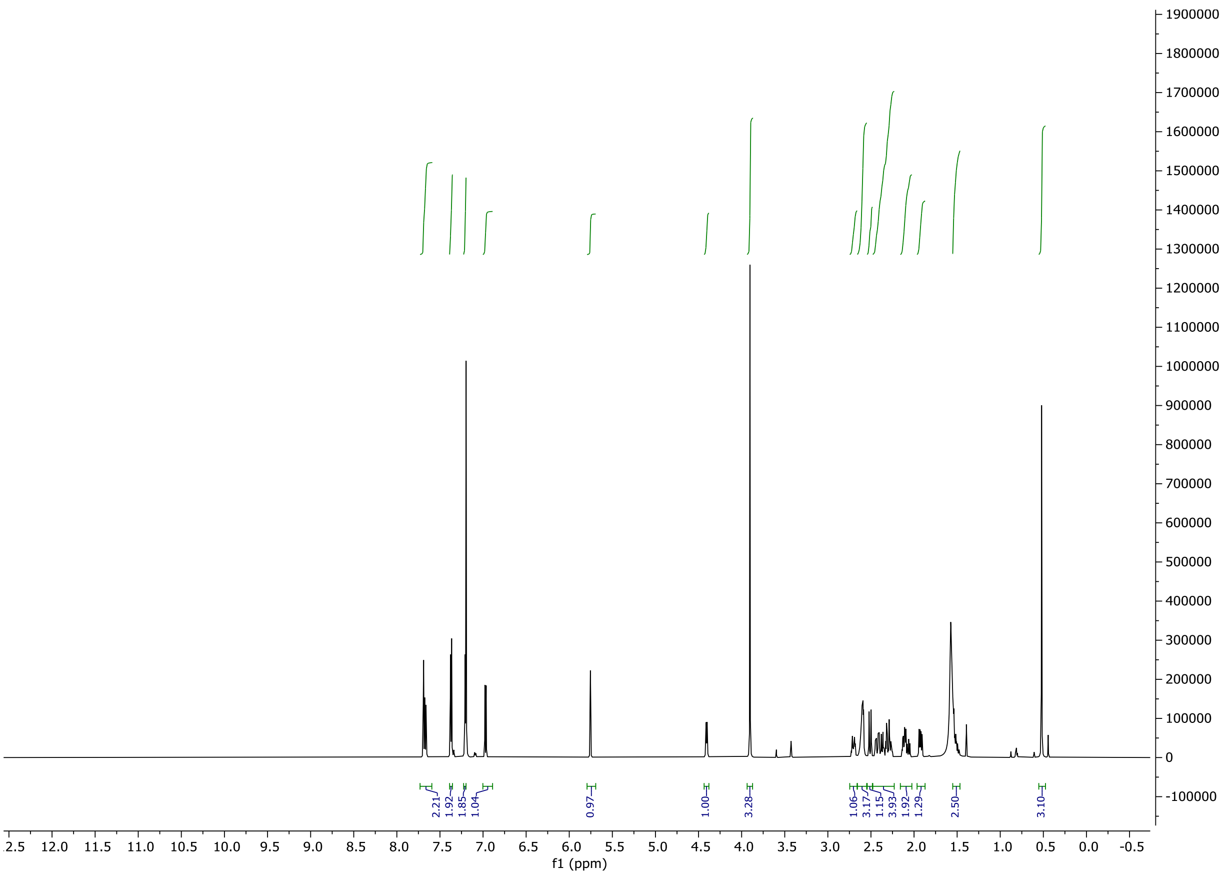

^{13}C NMR (100 MHz, $CDCl_3$) Compound **2j**

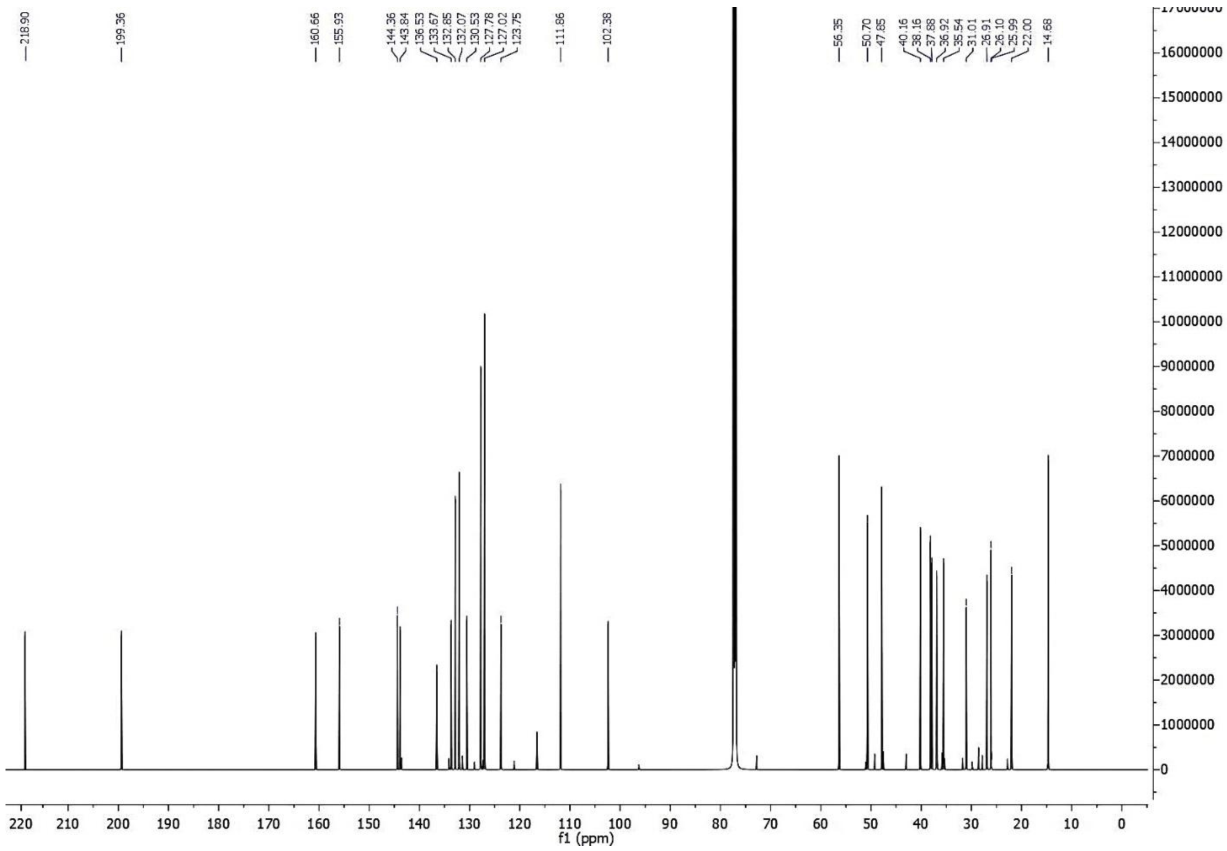

^{1}H NMR (400 MHz, $CDCl_3$) Compound **2k**

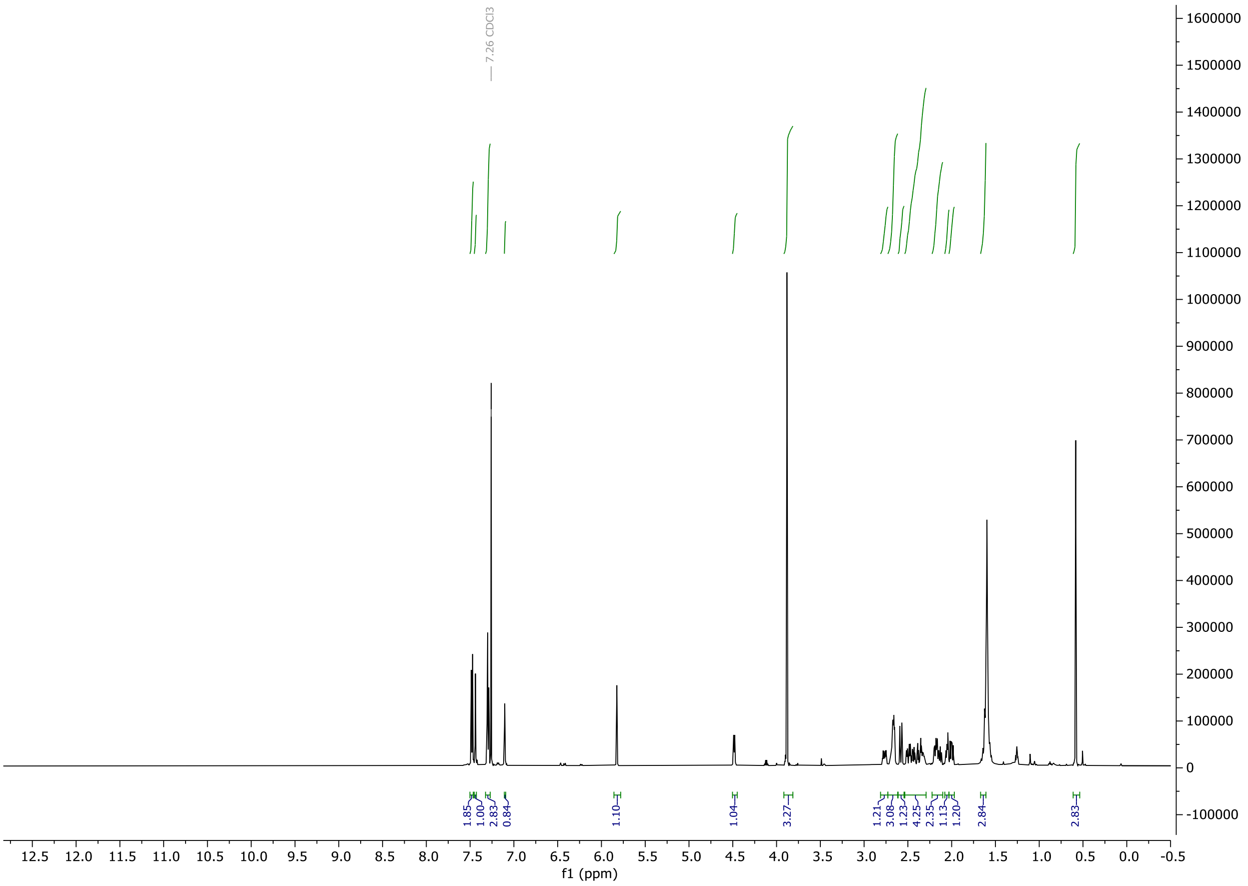

^{13}C NMR (100 MHz, $CDCl_3$) Compound **2k**

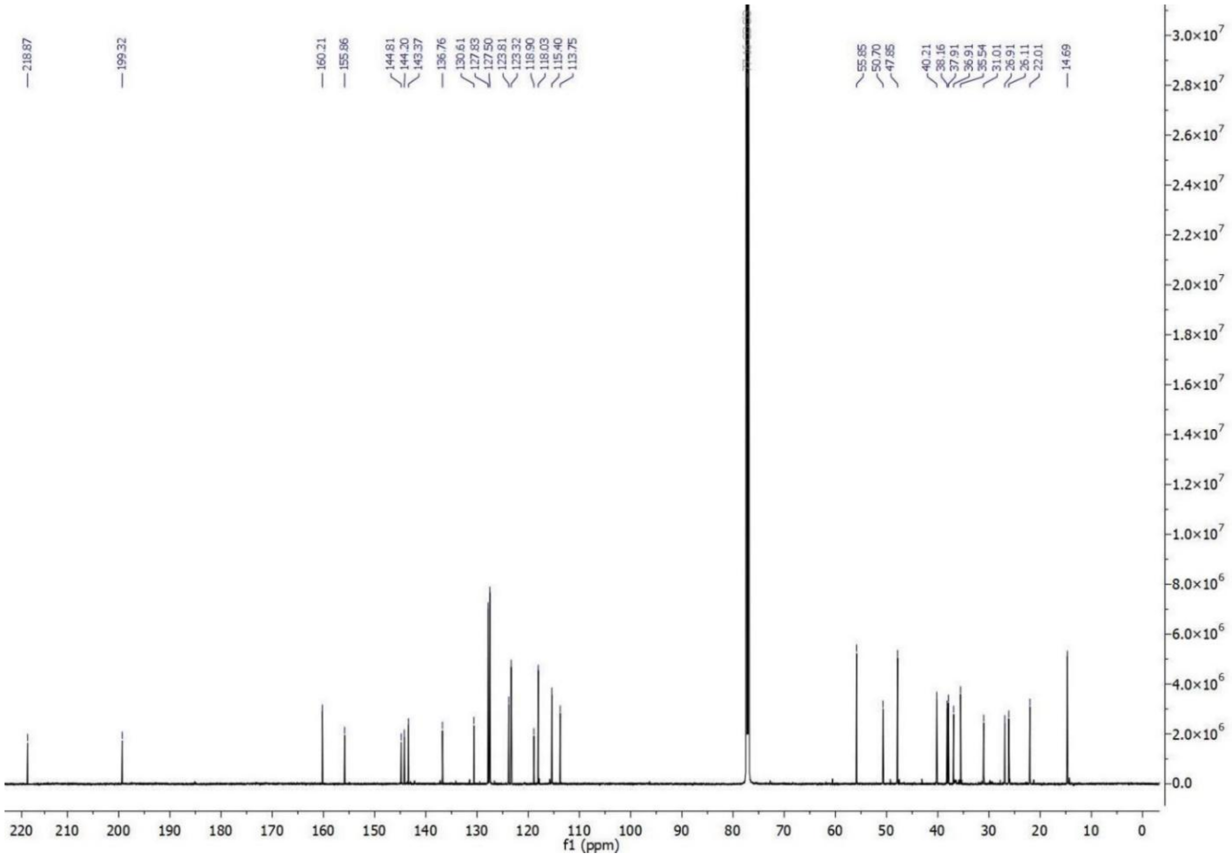

^{1}H NMR (400 MHz, $CDCl_3$) Compound **2l**

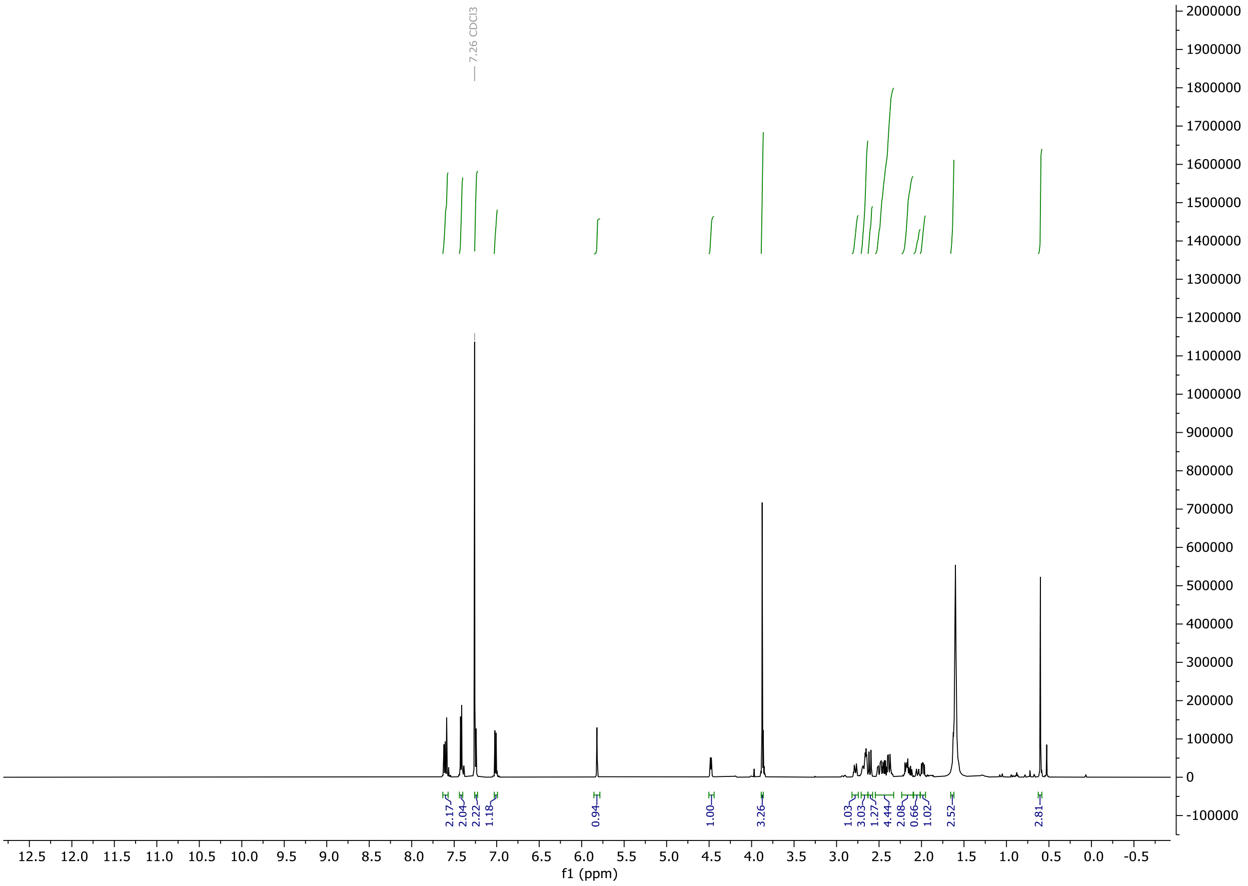

^{13}C NMR (100 MHz, $CDCl_3$) Compound **2l**

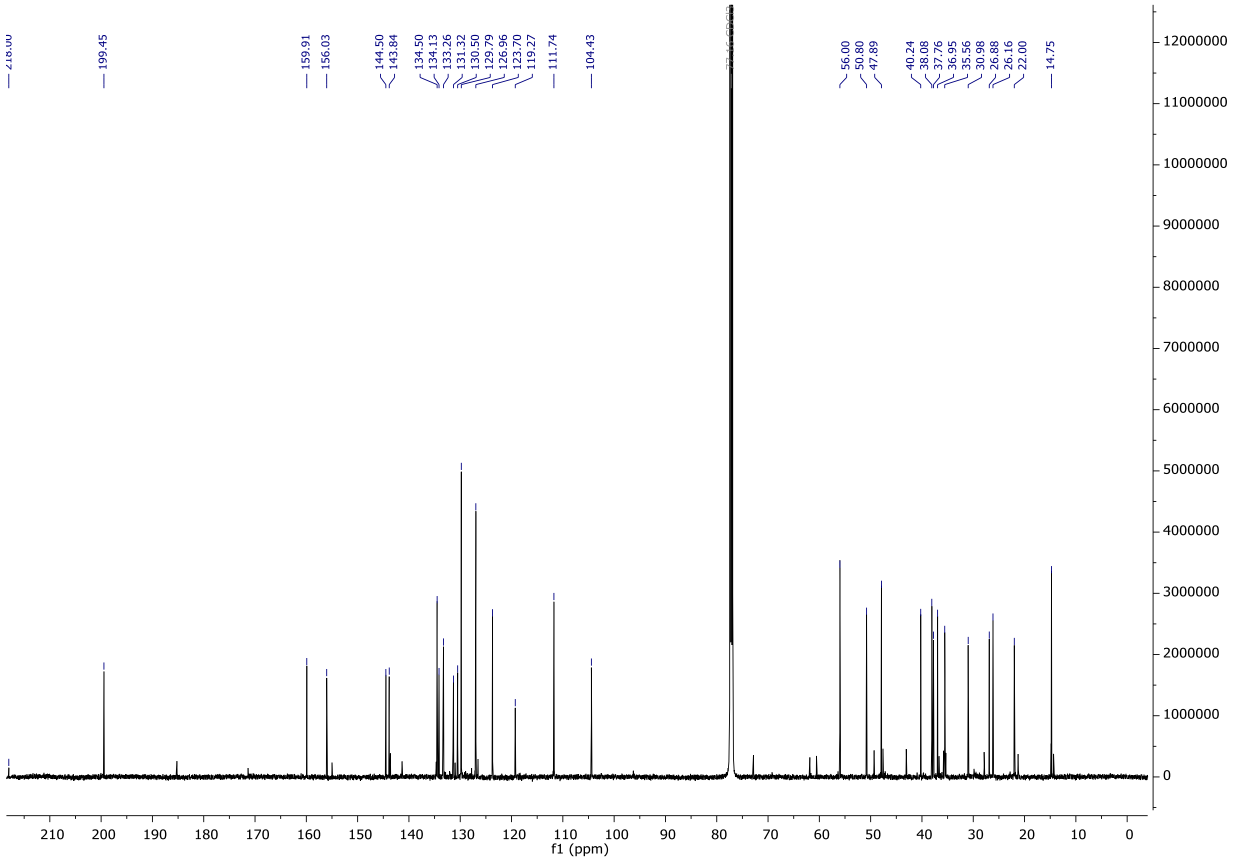

^{1}H NMR (400 MHz, $CDCl_3$) Compound **2m**

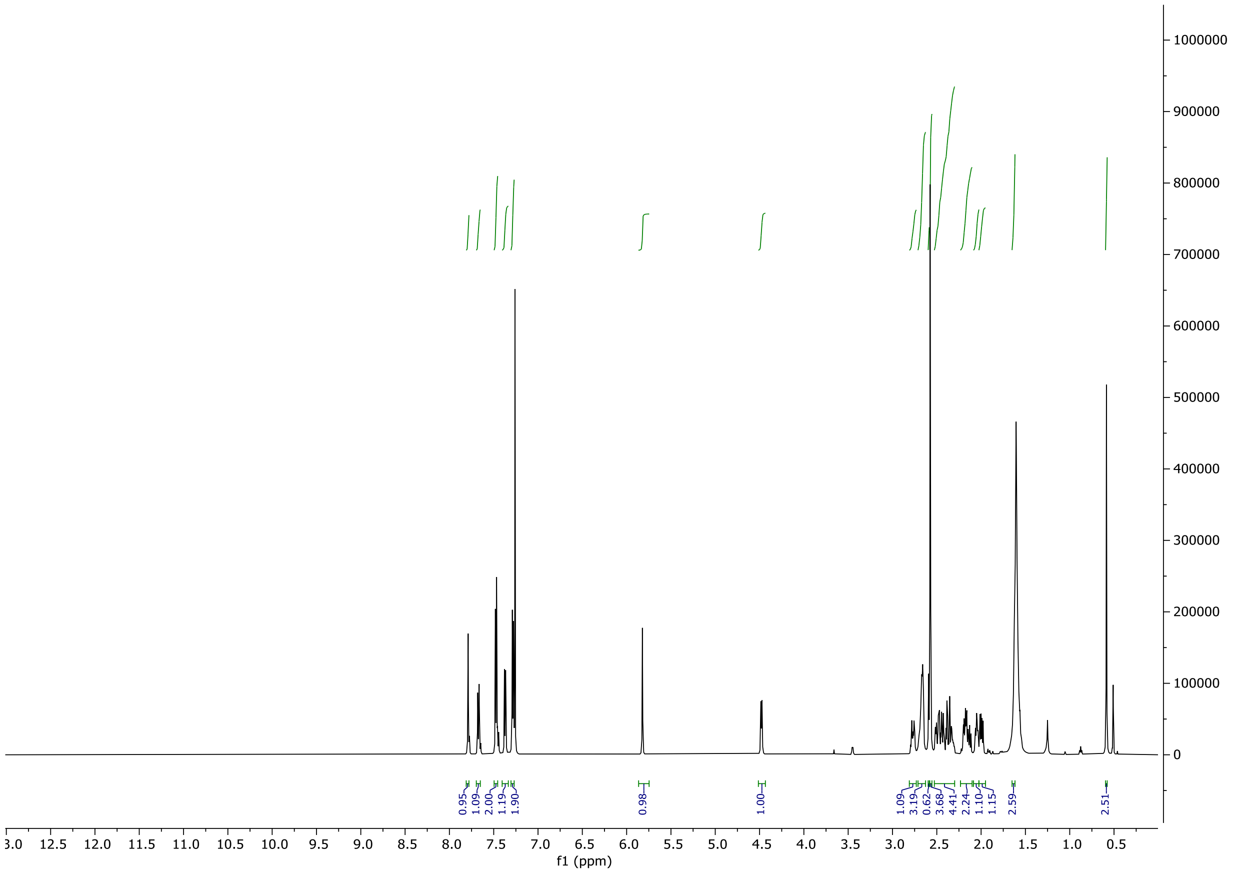

^{13}C NMR (100 MHz, $CDCl_3$) Compound **2m**

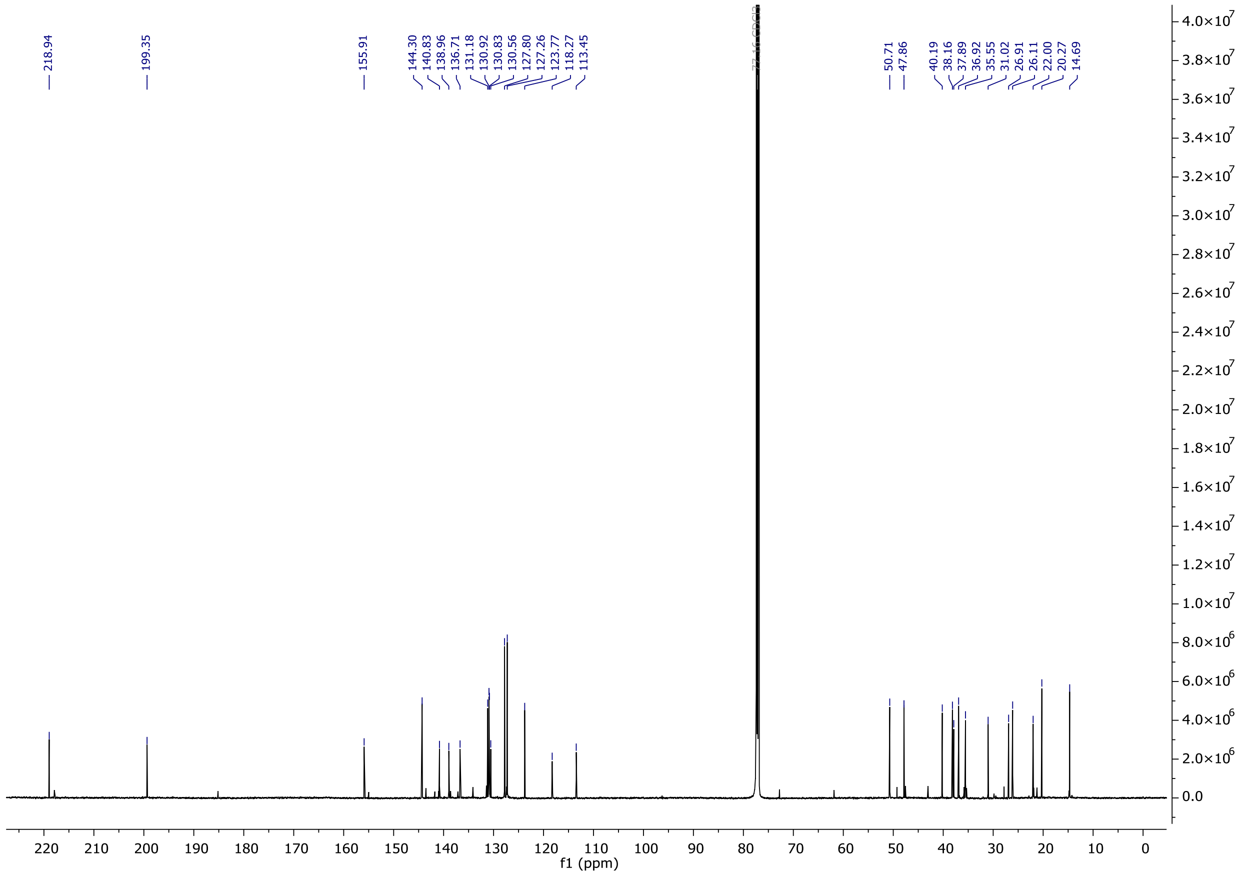

^{1}H NMR (400 MHz, $CDCl_3$) Compound **2n**

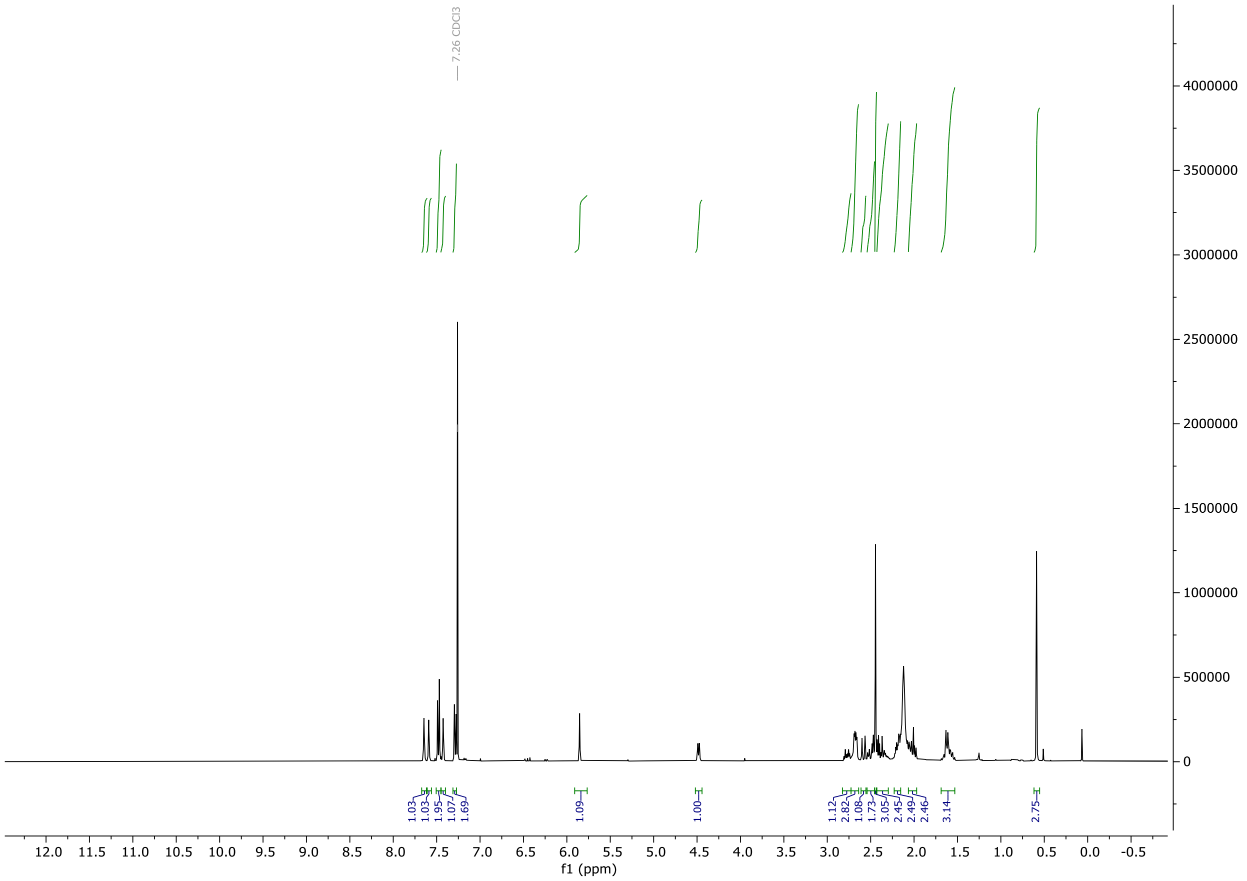

^{13}C NMR (100 MHz, $CDCl_3$) Compound **2n**

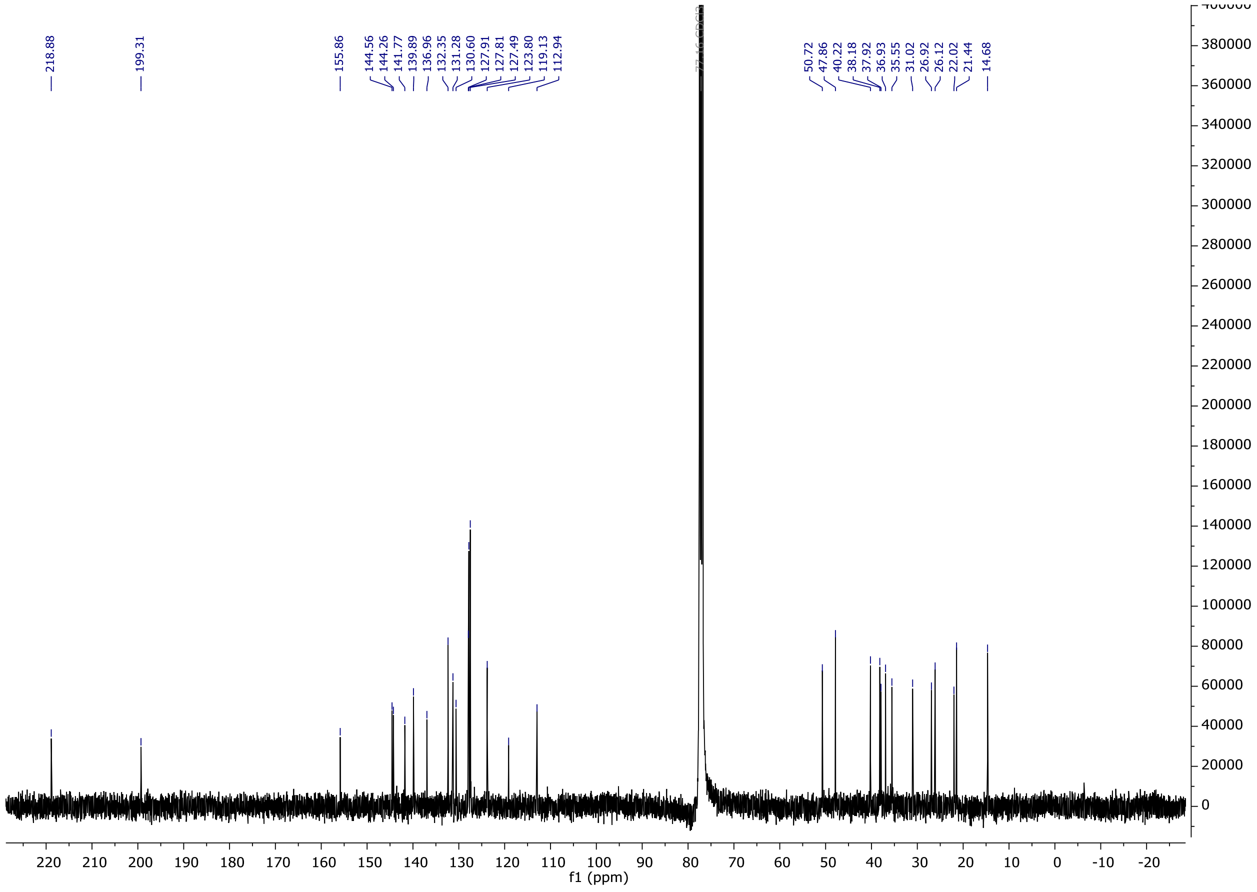

^{1}H NMR (400 MHz, CDCl$_3$) Compound **2o**

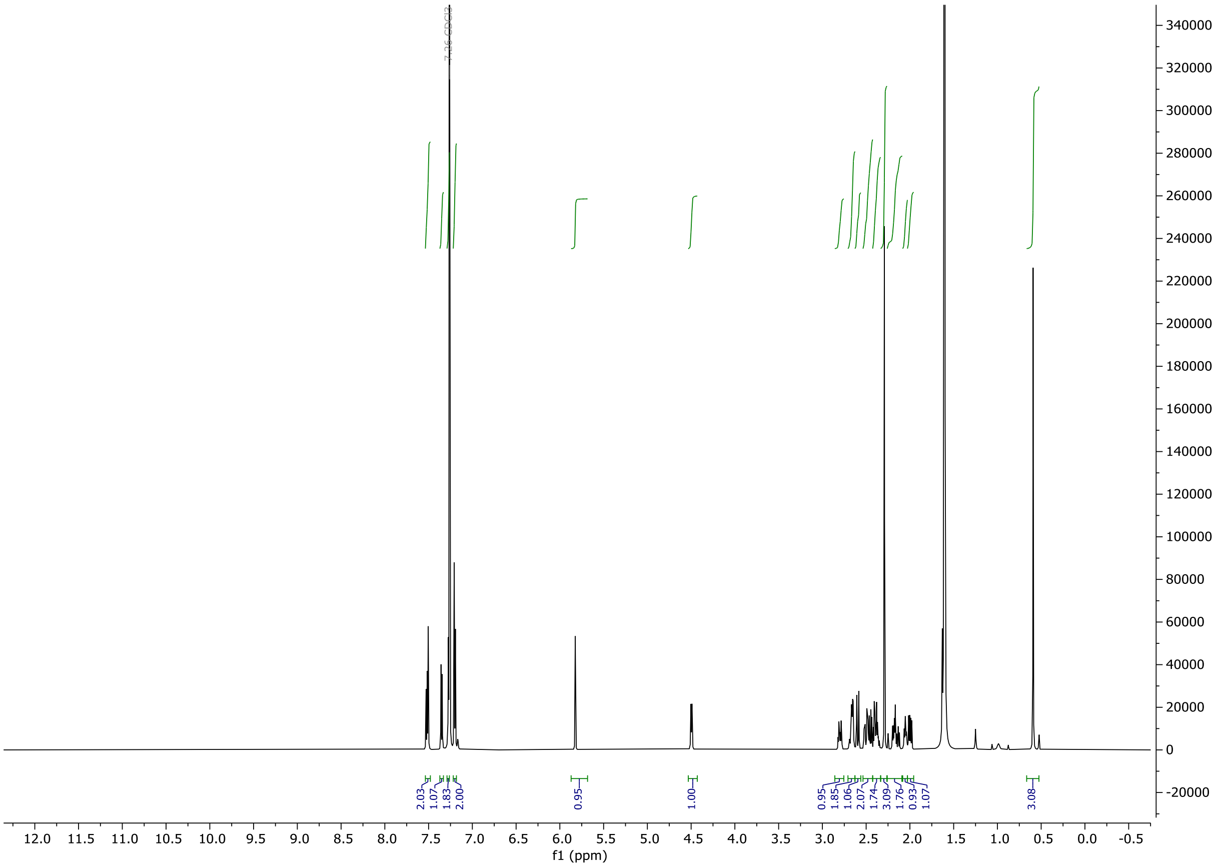

^{13}C NMR (100 MHz, CDCl$_3$) Compound **2o**

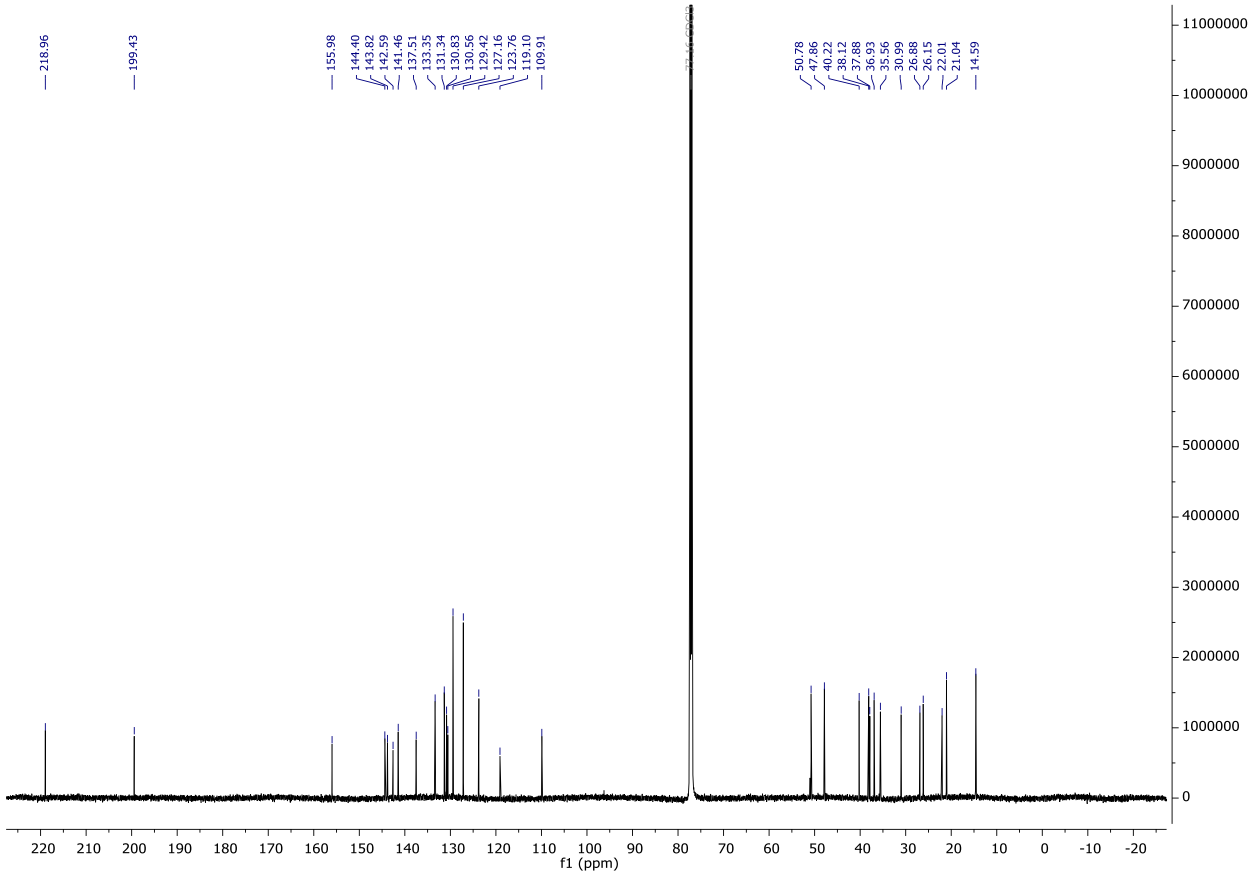

^{1}H NMR (400 MHz, $CDCl_3$) Compound **2p**

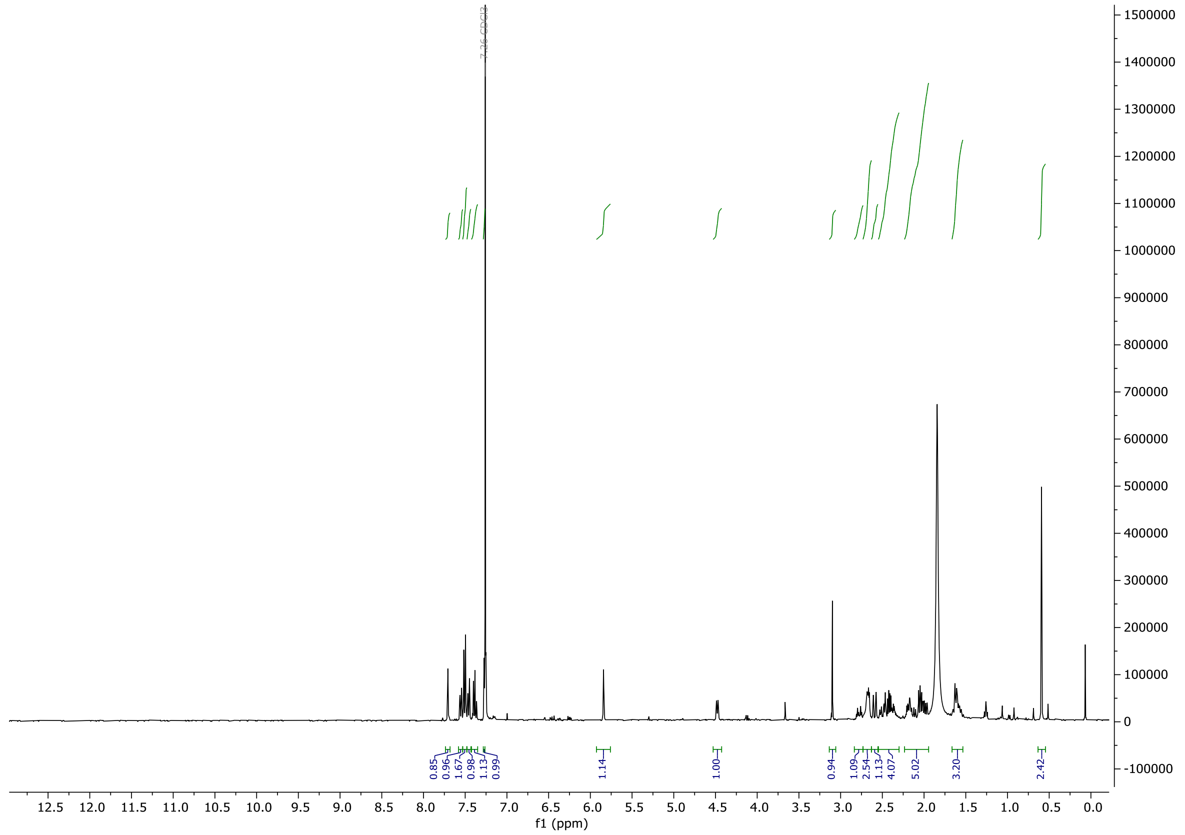

^{13}C NMR (100 MHz, $CDCl_3$) Compound **2p**

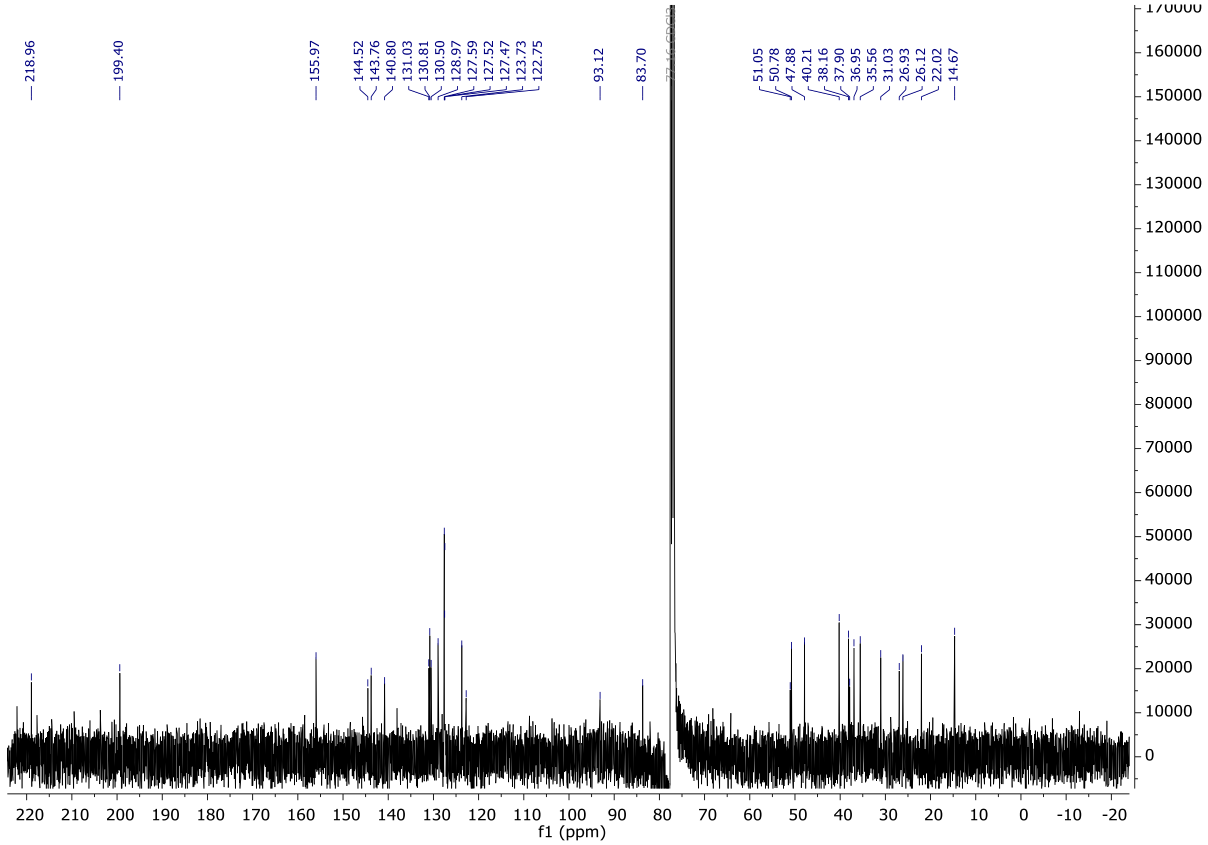

^{1}H NMR (400 MHz, $CDCl_3$) Compound **2q**

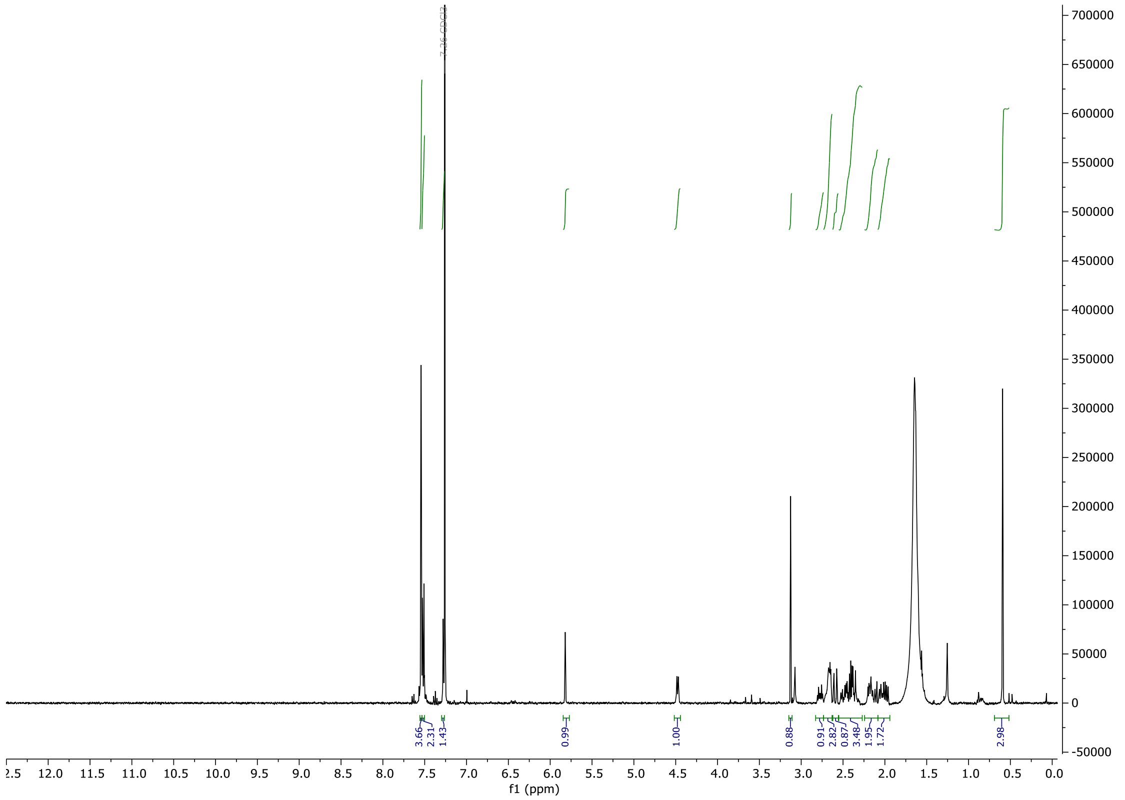

^{13}C NMR (100 MHz, $CDCl_3$) Compound **2q**

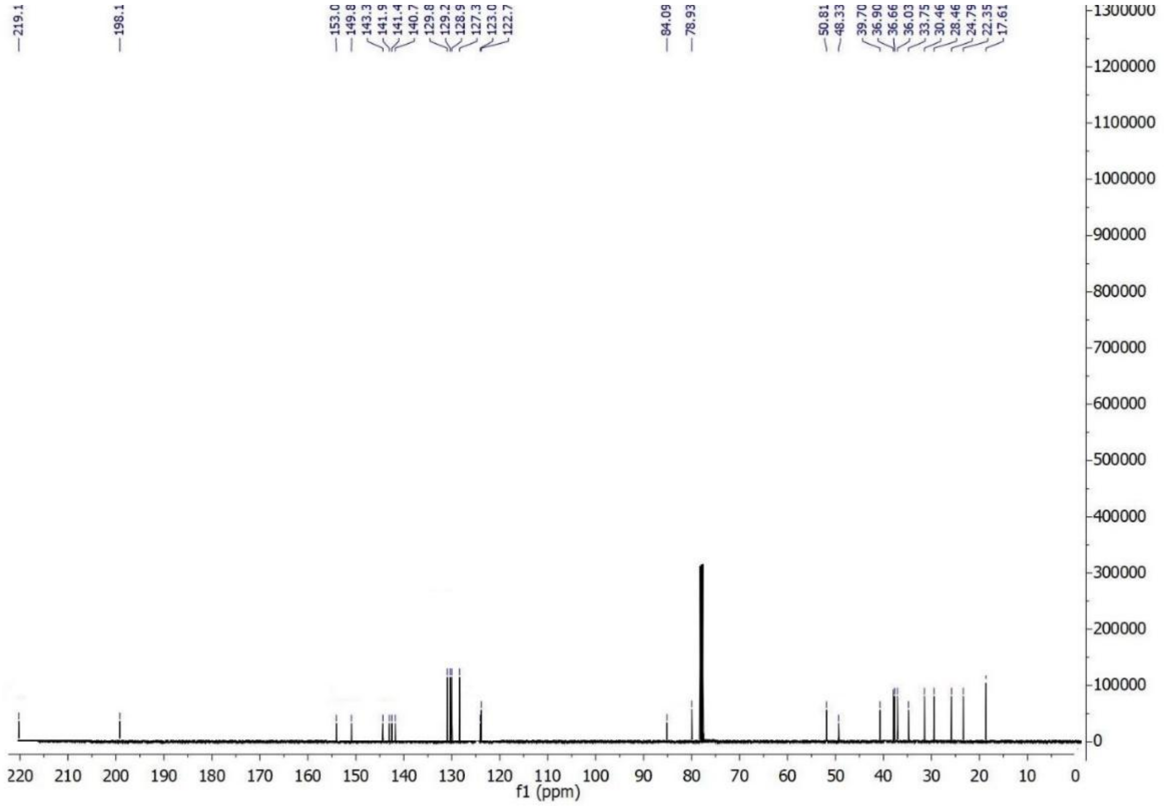

^{1}H NMR (400 MHz, CDCl$_3$) Compound **2r**

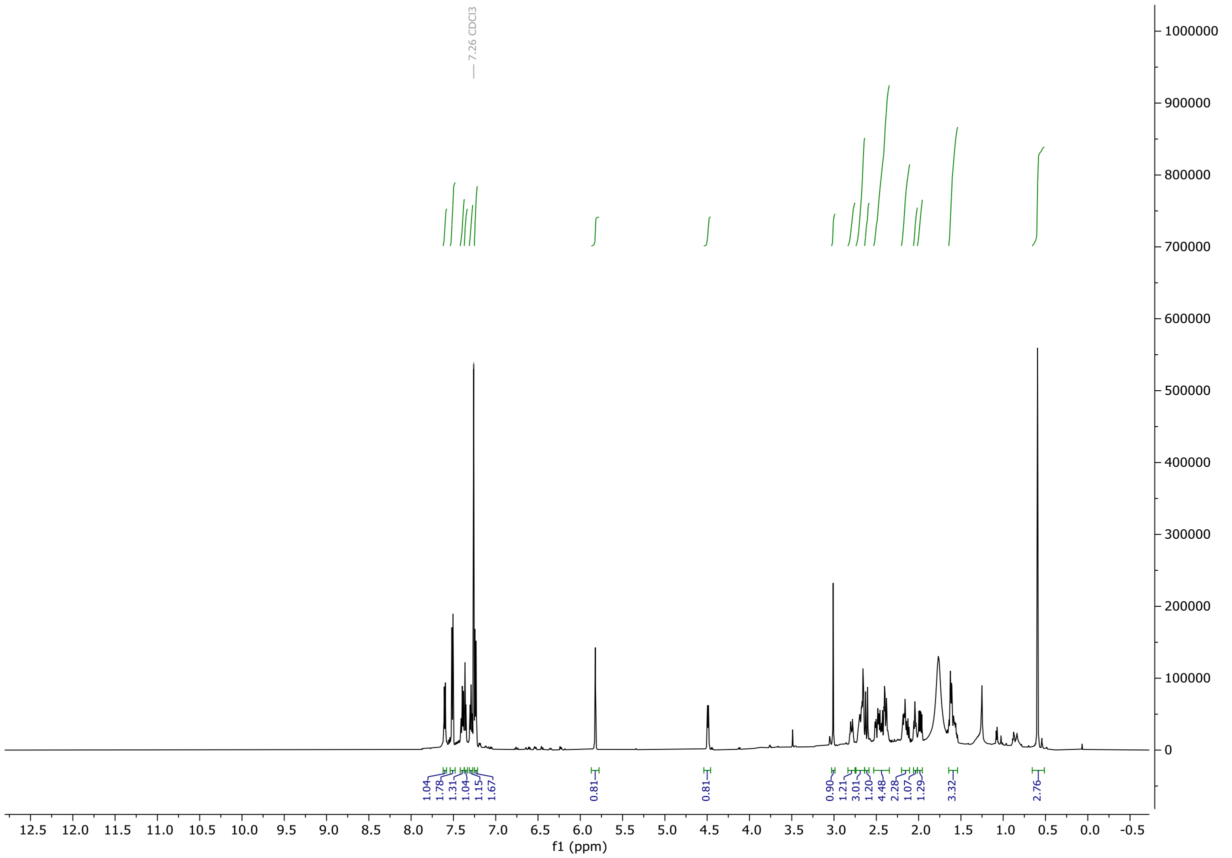

^{13}C NMR (100 MHz, CDCl$_3$) Compound **2r**

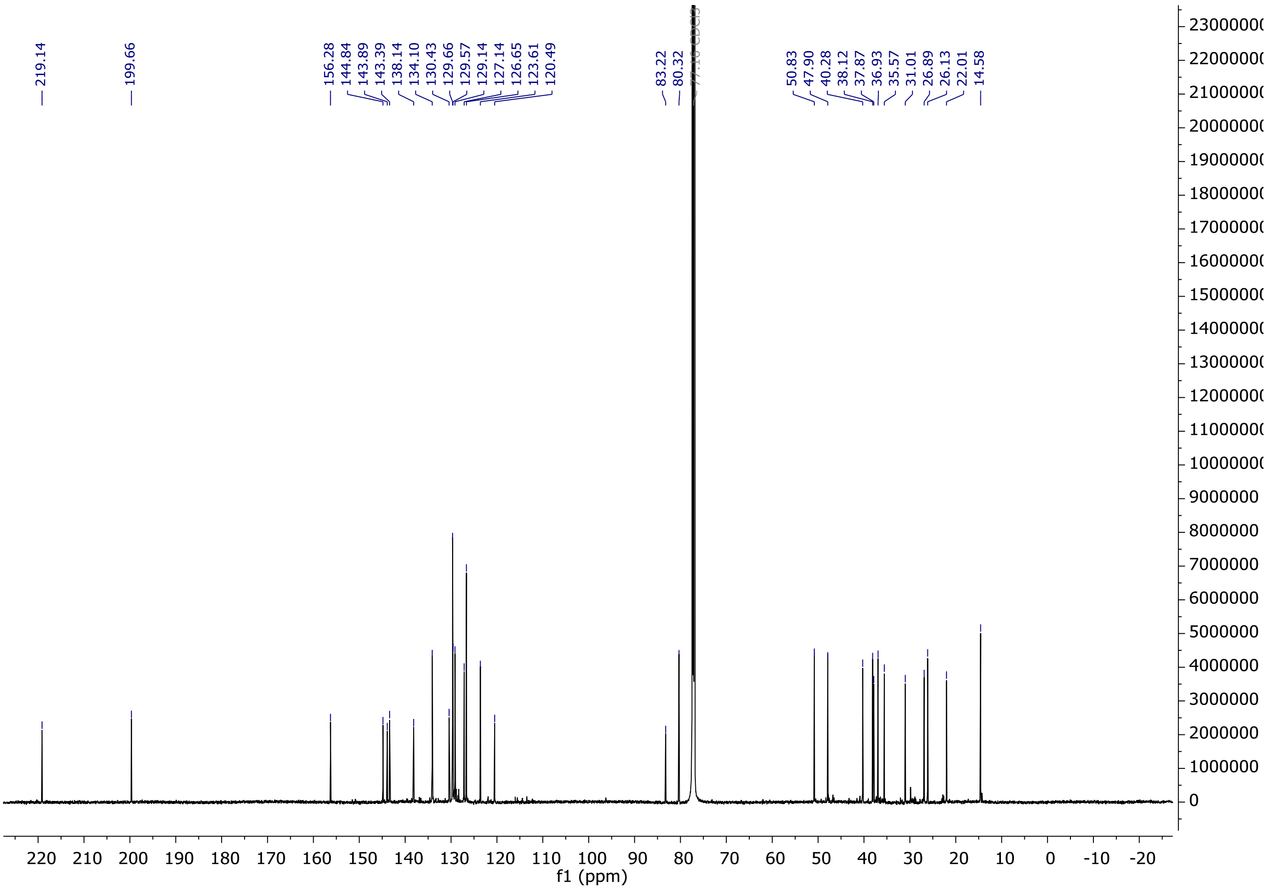

^{1}H NMR (400 MHz, $CDCl_3$) Compound **2s**

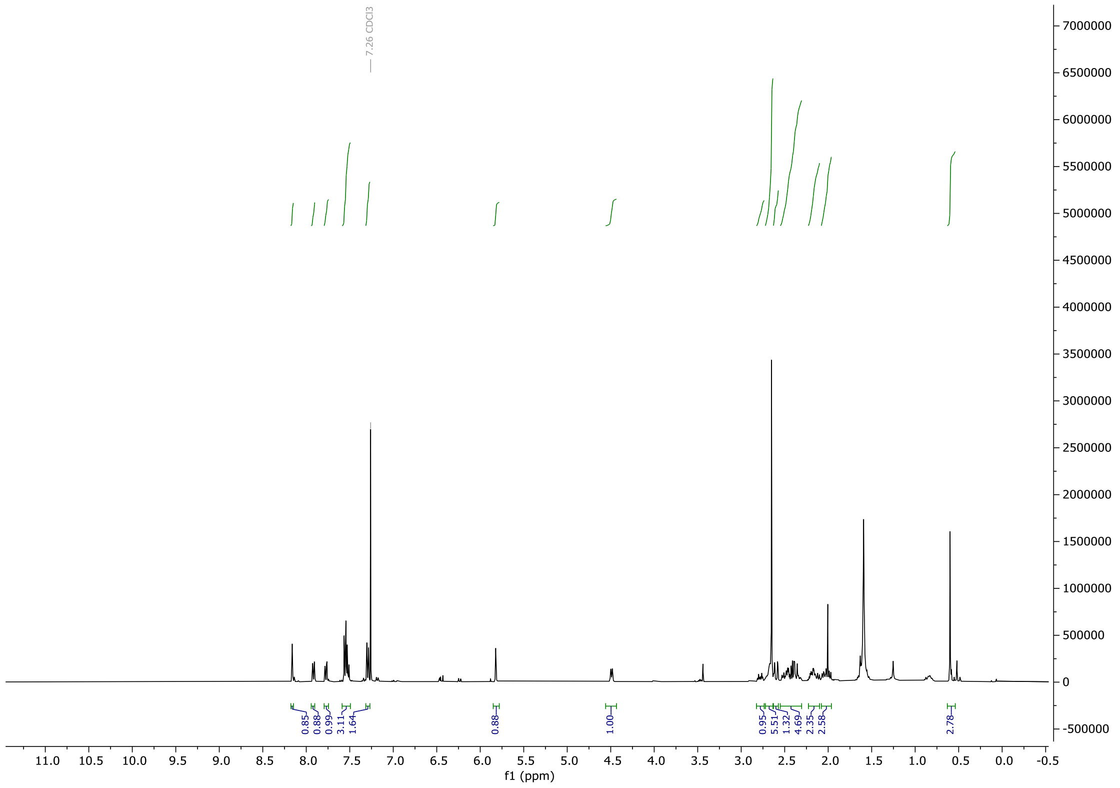

^{13}C NMR (100 MHz, $CDCl_3$) Compound **2s**

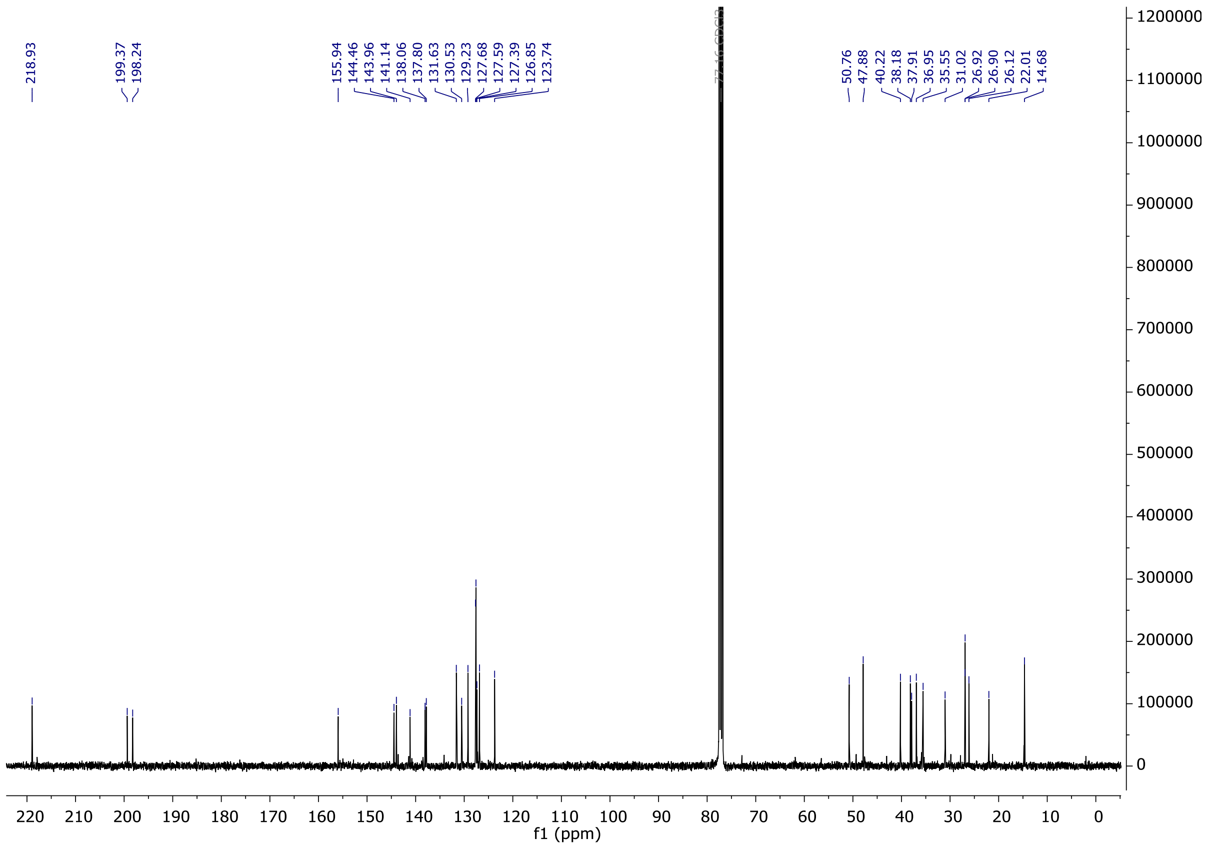

^{1}H NMR (CDCl$_3$, 400 MHz) Compound **2t**

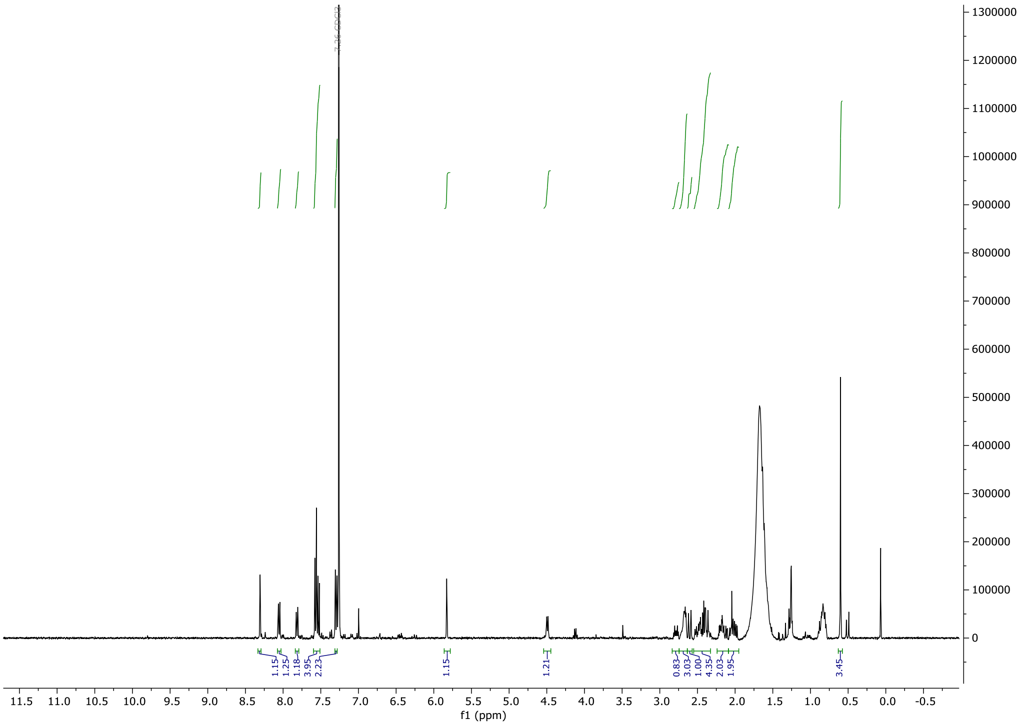

^{13}C NMR (100 MHz, CDCl$_3$) Compound **2t**

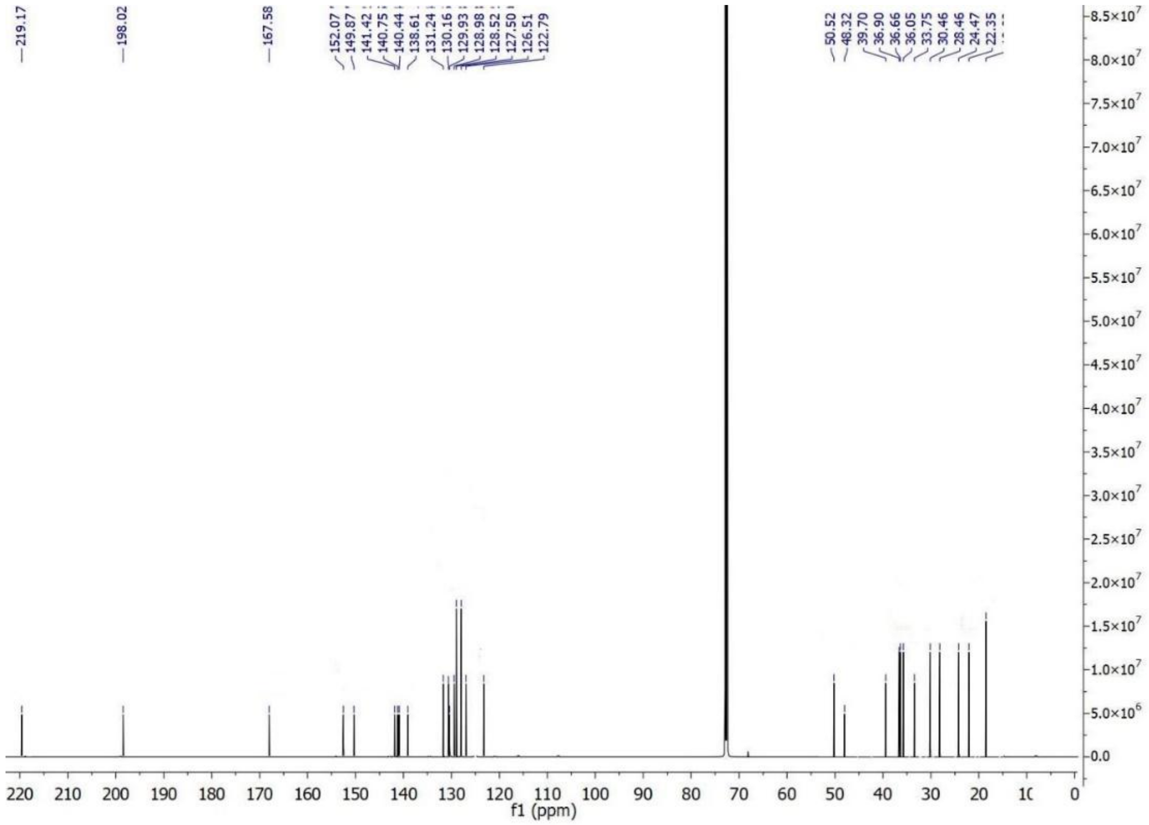

HPLC traces of selected compounds

The purity of compounds was determined by analytical HPLC analysis *via* Knauer system equipped with AZURA P 6.1L Analytical HPLC Pumps and AZURA® RID 2.1L Refractive Index Detector. The analysis was performed on a Luna 5µm C18 100 Å (250 x 4.6 mm) with ACN/H_2O (80:20) and 0.1% FA as eluent (flow rate 1 mL/min) for compounds **1a**, **1c**, **1d**, **1f-1h**, **2a-2s**. The analysis of compounds **1e** and **2t** was performed on a Luna 5µm C18 100 Å (250 x 4.6 mm) with ACN/H_2O (65:35) and 0.1% FA as eluent (flow rate 1 mL/min) while the analysis of compound **1b** was evaluated on a Luna 5µm C18 100 Å (250 x 4.6 mm) with ACN/H_2O (65:35) as eluent (flow rate 1 mL/min).

HPLC trace of compound **1a**

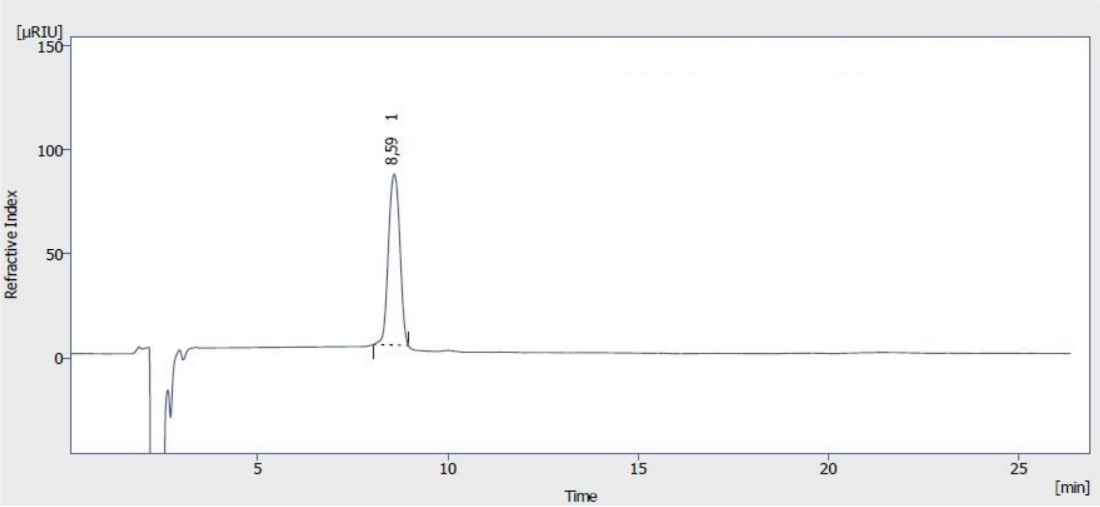

	Reten. Time [min]	Area [µRIU.s]	Height [µRIU]	Area [%]	Height [%]	W05 [min]	Compound Name
1	8,590	1712,841	82,149	100,0	100,0	0,34	
	Total	1712,841	82,149	100,0	100,0		

HPLC trace of compound **1b**

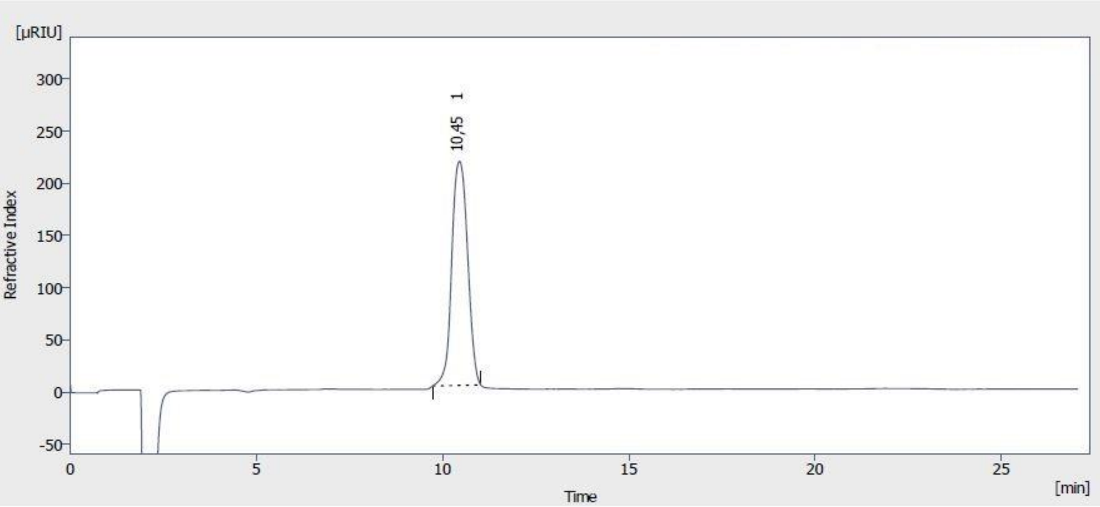

	Reten. Time [min]	Area [µRIU.s]	Height [µRIU]	Area [%]	Height [%]	W05 [min]	Compound Name
1	10,453	6328,814	214,536	100,0	100,0	0,47	
	Total	6328,814	214,536	100,0	100,0		

HPLC trace of compound **1c**

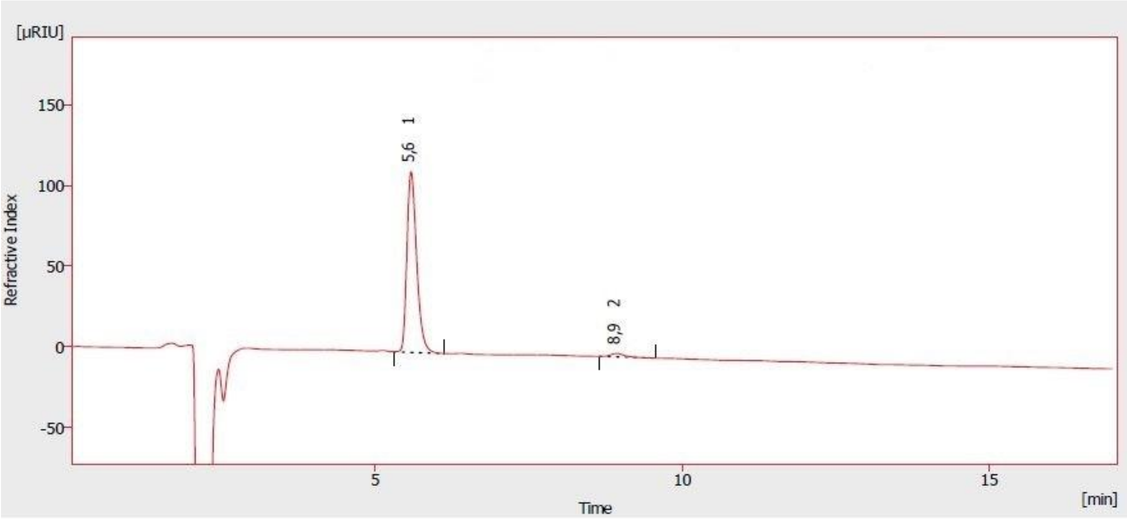

	Reten. Time [min]	Area [µRIU.s]	Height [µRIU]	Area [%]	Height [%]	W05 [min]	Compound Name
1	5,580	1236,158	111,813	97,3	98,2	0,17	
2	8,927	34,177	2,078	2,7	1,8	0,23	
	Total	1270,335	113,890	100,0	100,0		

HPLC trace of compound **1d**

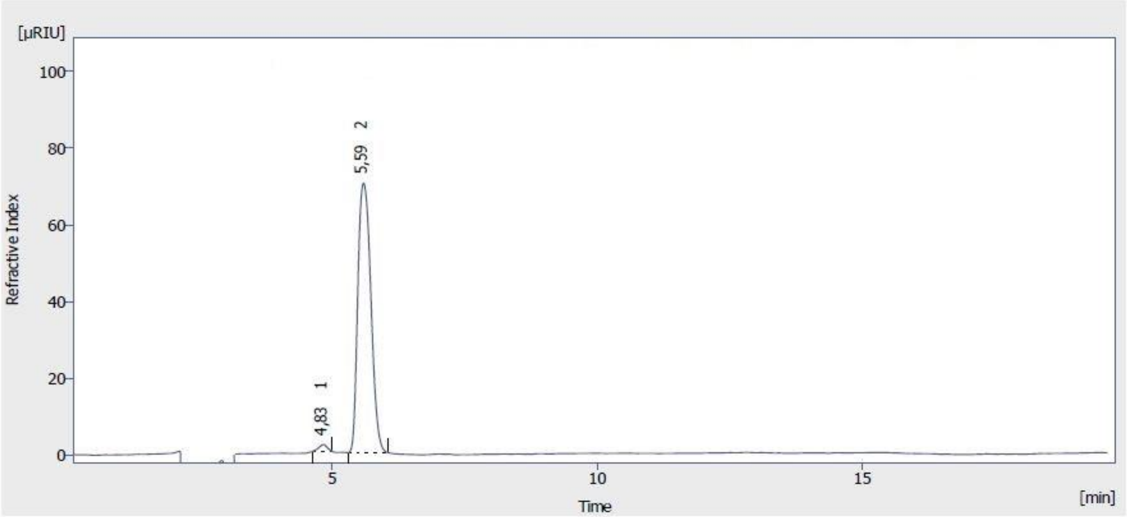

	Reten. Time [min]	Area [µRIU.s]	Height [µRIU]	Area [%]	Height [%]	W05 [min]	Compound Name
1	4,833	18,269	1,722	1,5	2,4	0,17	
2	5,593	1199,183	70,222	98,5	97,6	0,28	
	Total	1217,451	71,943	100,0	100,0		

HPLC trace of compound **1e**

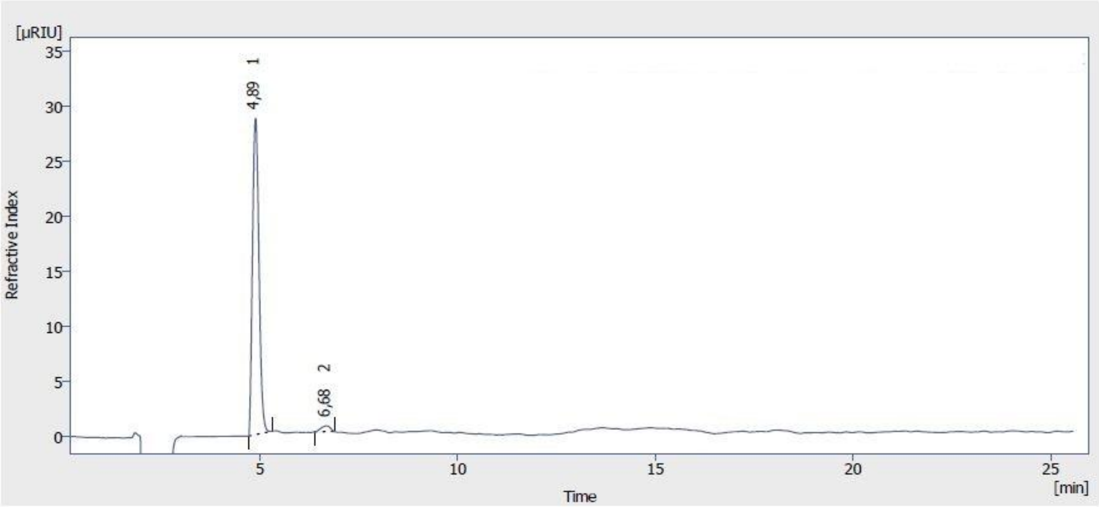

	Reten. Time [min]	Area [µRIU.s]	Height [µRIU]	Area [%]	Height [%]	W05 [min]	Compound Name
1	4,888	325,666	28,721	97,5	98,2	0,18	
2	6,683	8,469	0,526	2,5	1,8	0,27	
	Total	334,135	29,247	100,0	100,0		

HPLC trace of compound **1f**

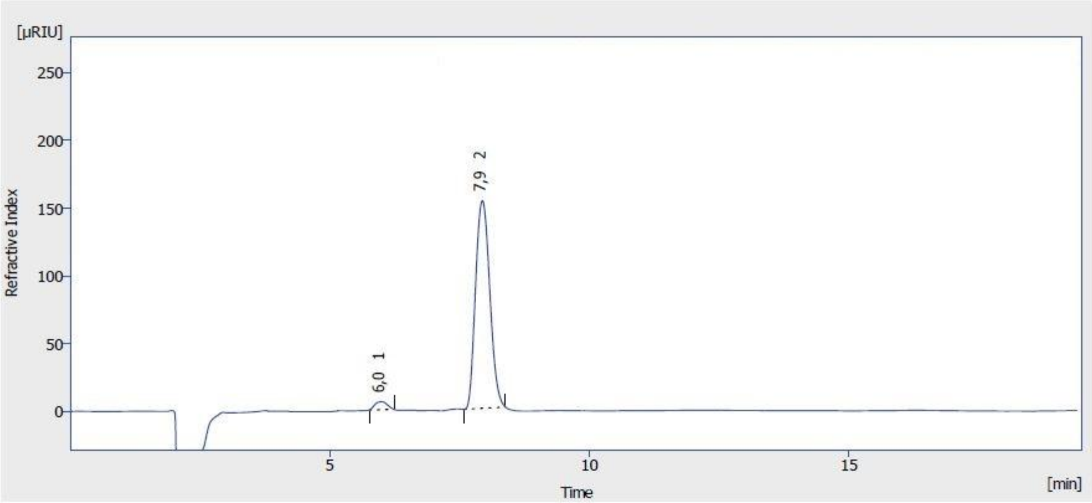

	Reten. Time [min]	Area [µRIU.s]	Height [µRIU]	Area [%]	Height [%]	W05 [min]	Compound Name
1	5,985	97,911	5,929	3,2	3,7	0,28	
2	7,938	2928,788	153,340	96,8	96,3	0,31	
	Total	3026,700	159,268	100,0	100,0		

HPLC trace of compound **1g**

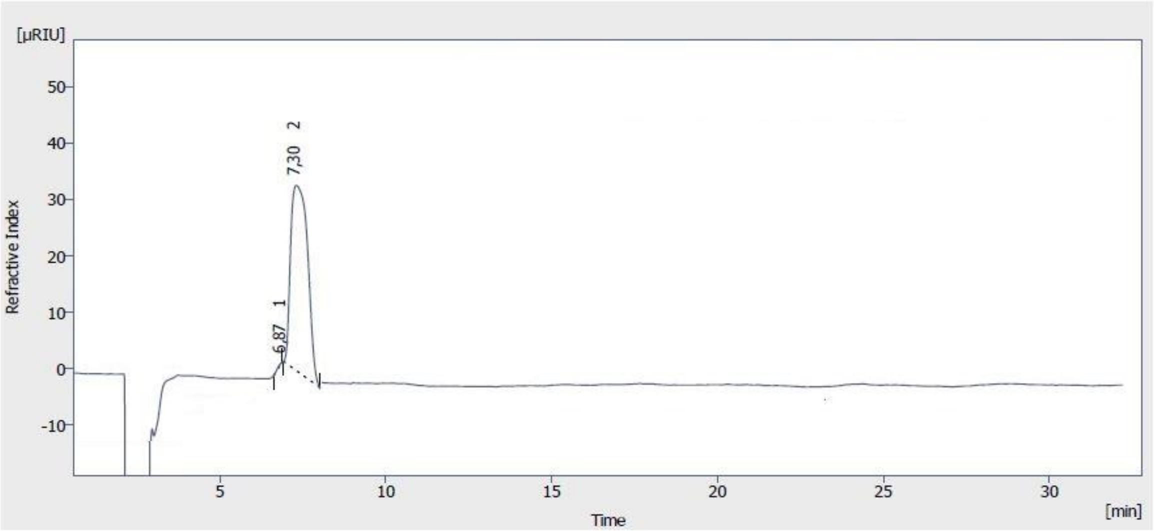

	Reten. Time [min]	Area [μRIU.s]	Height [μRIU]	Area [%]	Height [%]	W05 [min]	Compound Name
1	6,865	2,241	0,019	0,2	0,1	0,01	
2	7,305	1176,276	32,804	99,8	99,9	0,59	
	Total	1178,517	32,823	100,0	100,0		

HPLC trace of compound **1h**

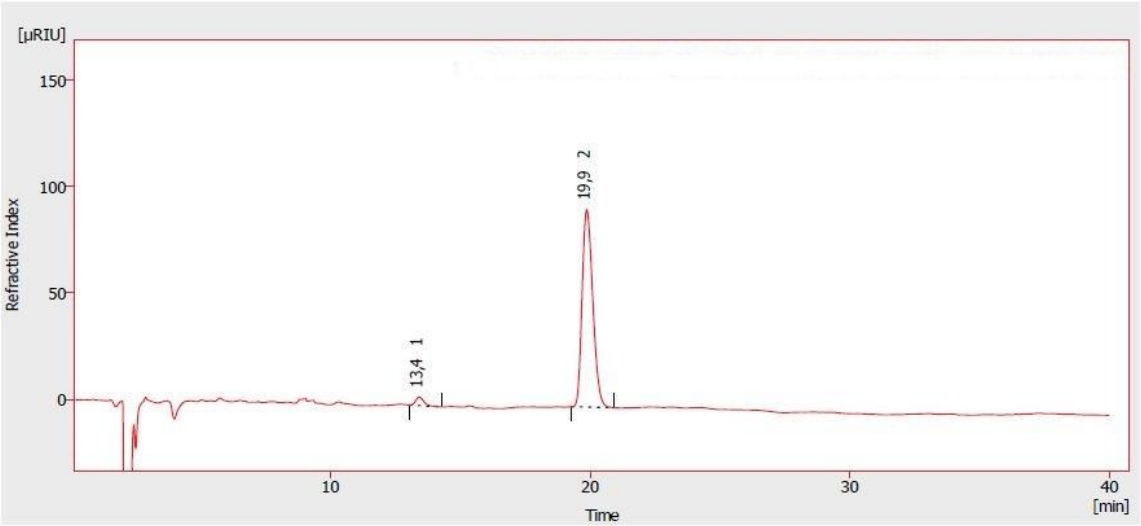

	Reten. Time [min]	Area [μRIU.s]	Height [μRIU]	Area [%]	Height [%]	W05 [min]	Compound Name
1	13,425	87,384	3,985	3,2	4,1	0,33	
2	19,870	2643,780	92,673	96,8	95,9	0,45	
	Total	2731,164	96,658	100,0	100,0		

HPLC trace of compound **2a**

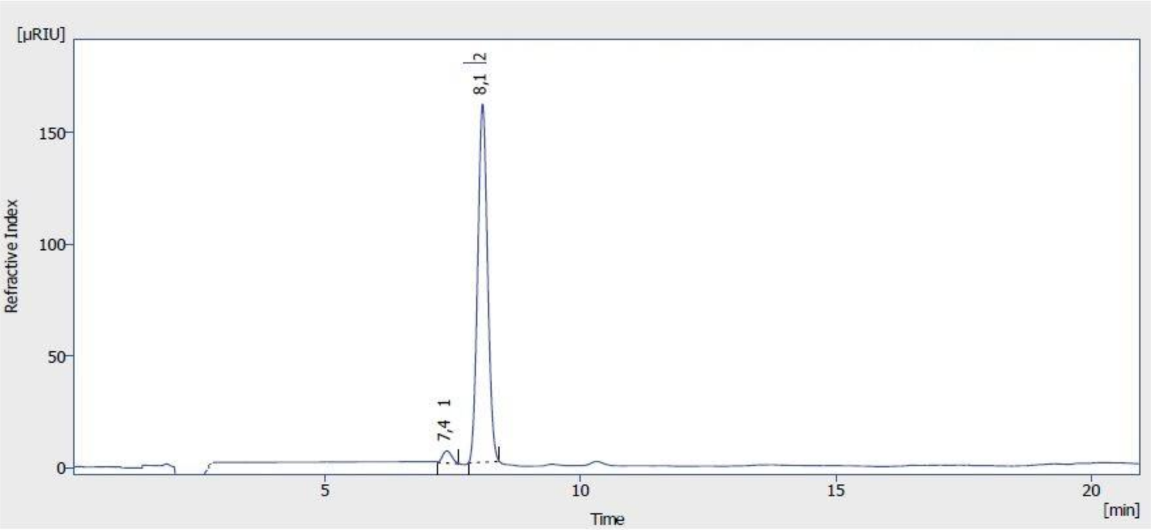

	Reten. Time [min]	Area [μRIU.s]	Height [μRIU]	Area [%]	Height [%]	W05 [min]	Compound Name
1	7,385	68,140	5,495	3,1	3,3	0,20	
2	8,085	2148,438	160,284	96,9	96,7	0,21	
	Total	2216,578	165,779	100,0	100,0		

HPLC trace of compound **2b**

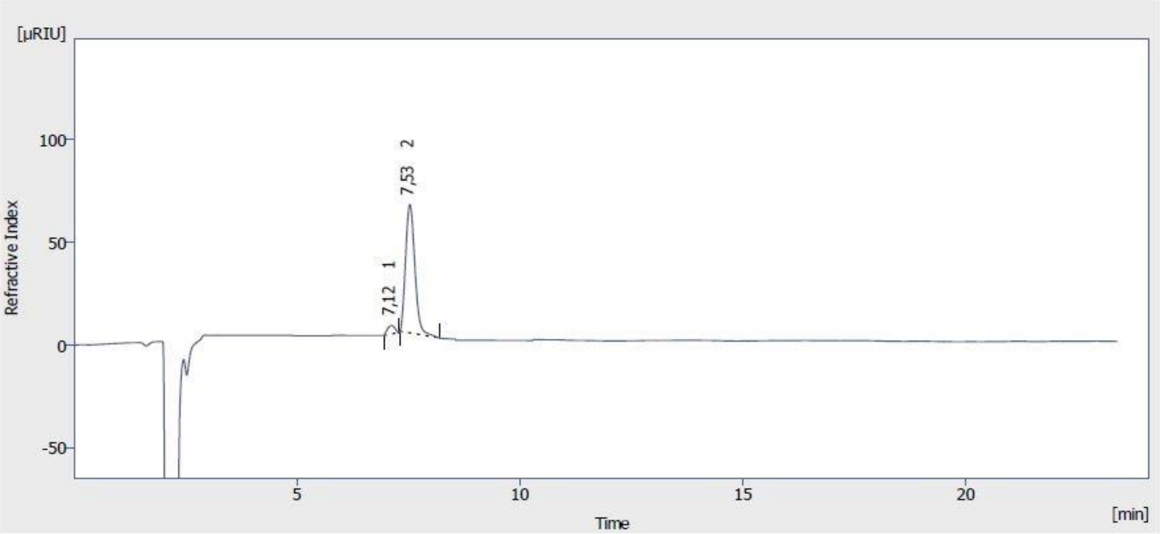

	Reten. Time [min]	Area [μRIU.s]	Height [μRIU]	Area [%]	Height [%]	W05 [min]	Compound Name
1	7,123	47,727	4,096	5,0	6,2	0,20	
2	7,533	905,253	62,481	95,0	93,8	0,23	
	Total	952,981	66,577	100,0	100,0		

HPLC trace of compound **2c**

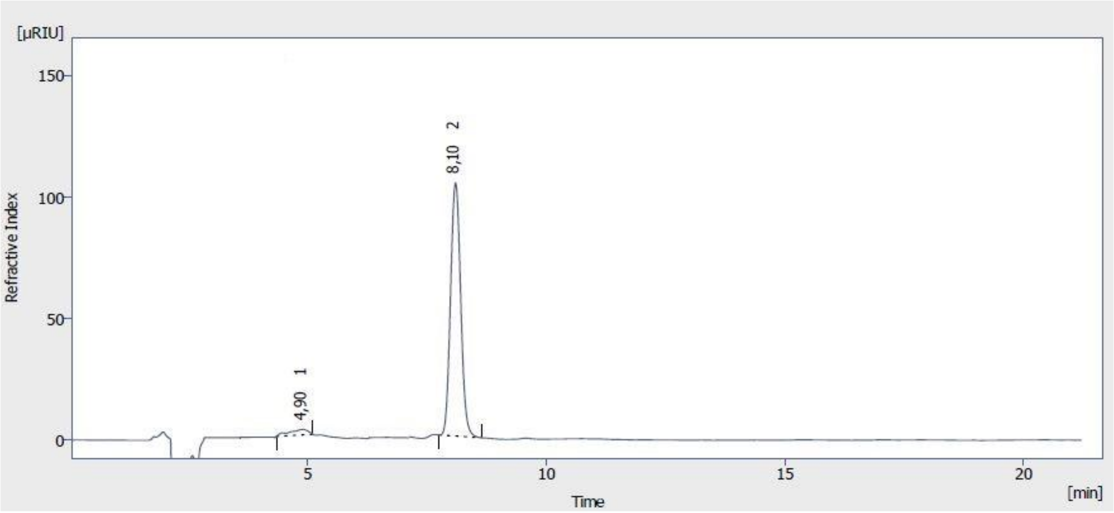

	Reten. Time [min]	Area [µRIU.s]	Height [µRIU]	Area [%]	Height [%]	W05 [min]	Compound Name
1	4,902	64,971	2,229	4,1	2,1	0,42	
2	8,098	1537,479	104,100	95,9	97,9	0,23	
	Total	1602,450	106,330	100,0	100,0		

HPLC trace of compound **2d**

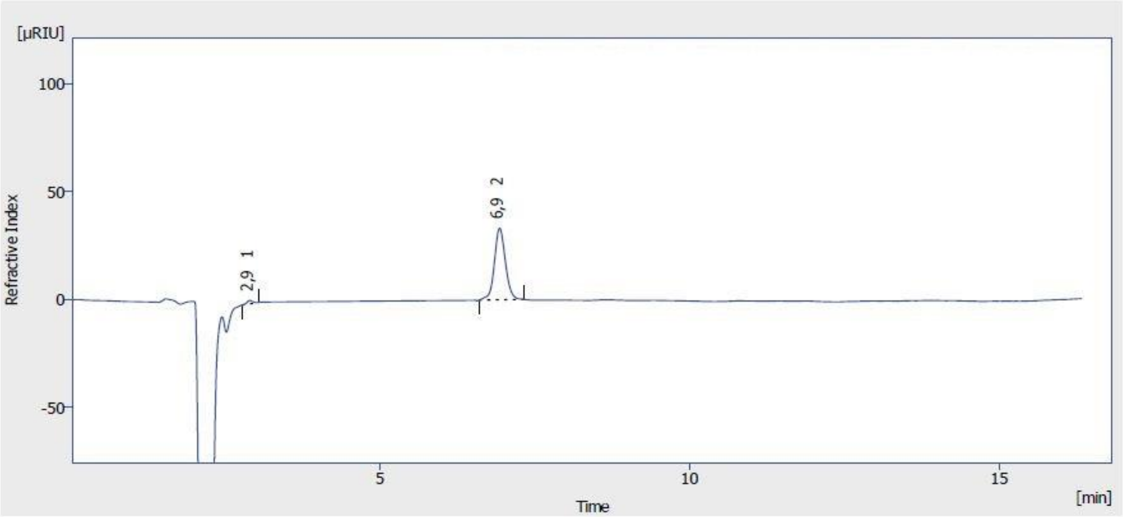

	Reten. Time [min]	Area [µRIU.s]	Height [µRIU]	Area [%]	Height [%]	W05 [min]	Compound Name
1	2,898	10,345	1,608	2,5	4,6	0,11	
2	6,932	408,735	33,172	97,5	95,4	0,19	
	Total	419,080	34,780	100,0	100,0		

HPLC trace of compound **2e**

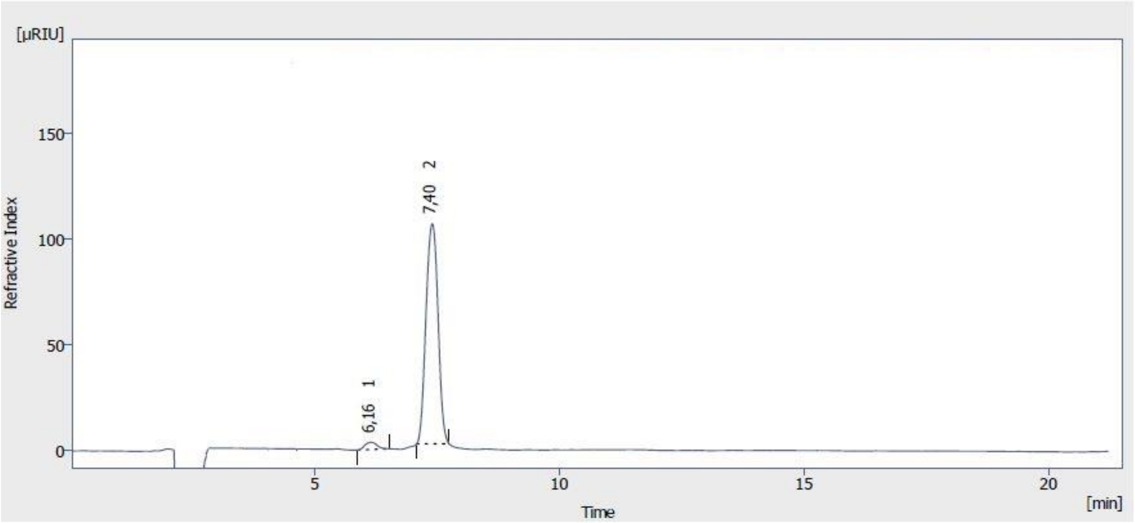

	Reten. Time [min]	Area [µRIU.s]	Height [µRIU]	Area [%]	Height [%]	W05 [min]	Compound Name
1	6,157	59,400	3,386	3,2	3,2	0,28	
2	7,403	1777,179	104,067	96,8	96,8	0,28	
	Total	1836,579	107,453	100,0	100,0		

HPLC trace of compound **2f**

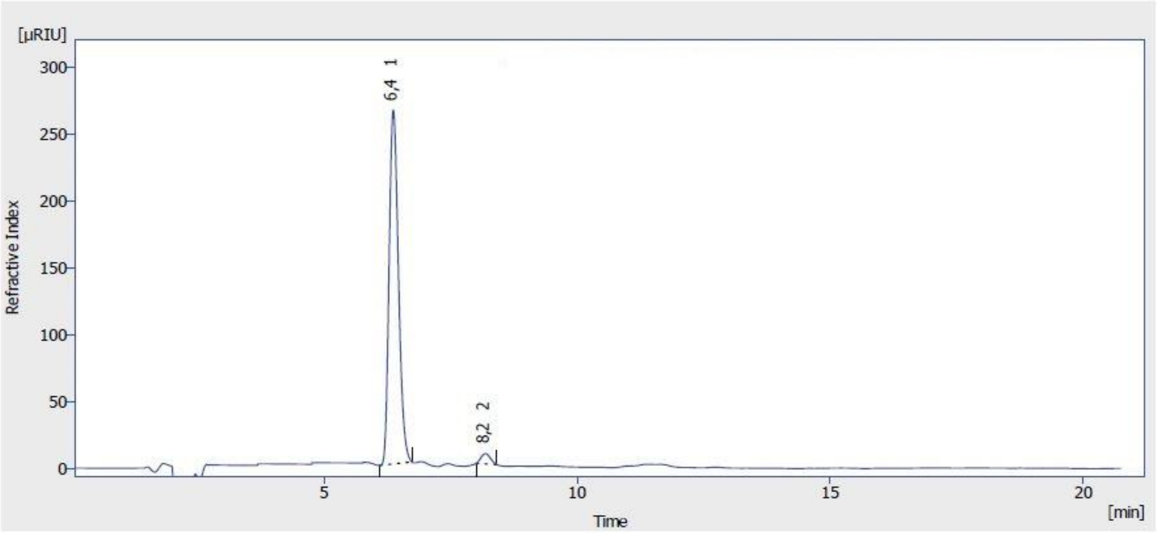

	Reten. Time [min]	Area [µRIU.s]	Height [µRIU]	Area [%]	Height [%]	W05 [min]	Compound Name
1	6,368	3385,840	264,403	97,2	97,2	0,20	
2	8,188	97,723	7,699	2,8	2,8	0,21	
	Total	3483,562	272,102	100,0	100,0		

HPLC trace of compound **2g**

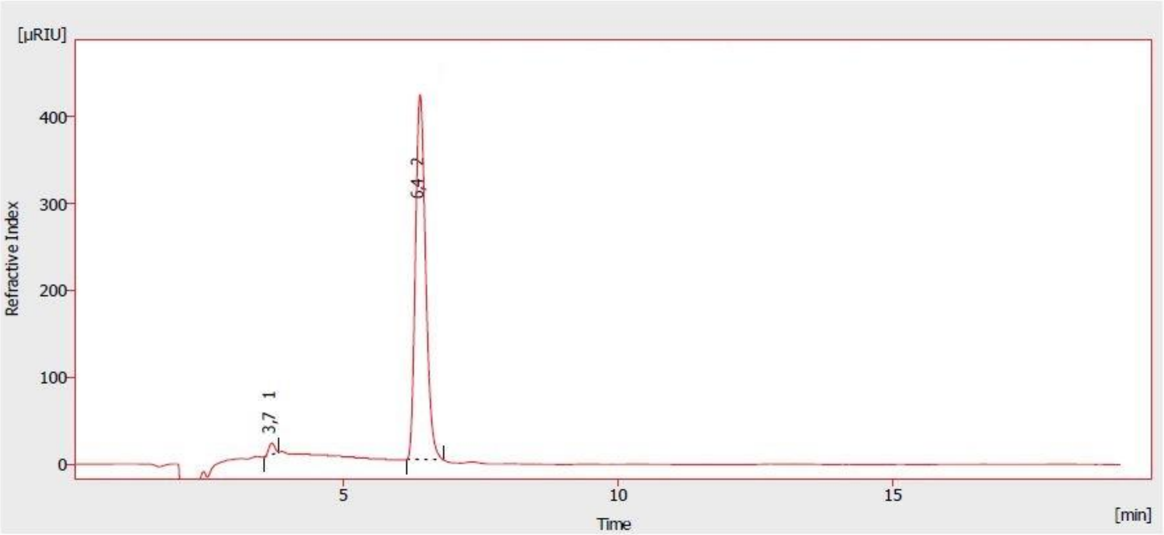

	Reten. Time [min]	Area [µRIU.s]	Height [µRIU]	Area [%]	Height [%]	W05 [min]	Compound Name
1	3,697	97,870	13,160	1,8	3,0	0,13	
2	6,395	5420,570	419,620	98,2	97,0	0,20	
	Total	5518,441	432,781	100,0	100,0		

HPLC trace of compound **2h**

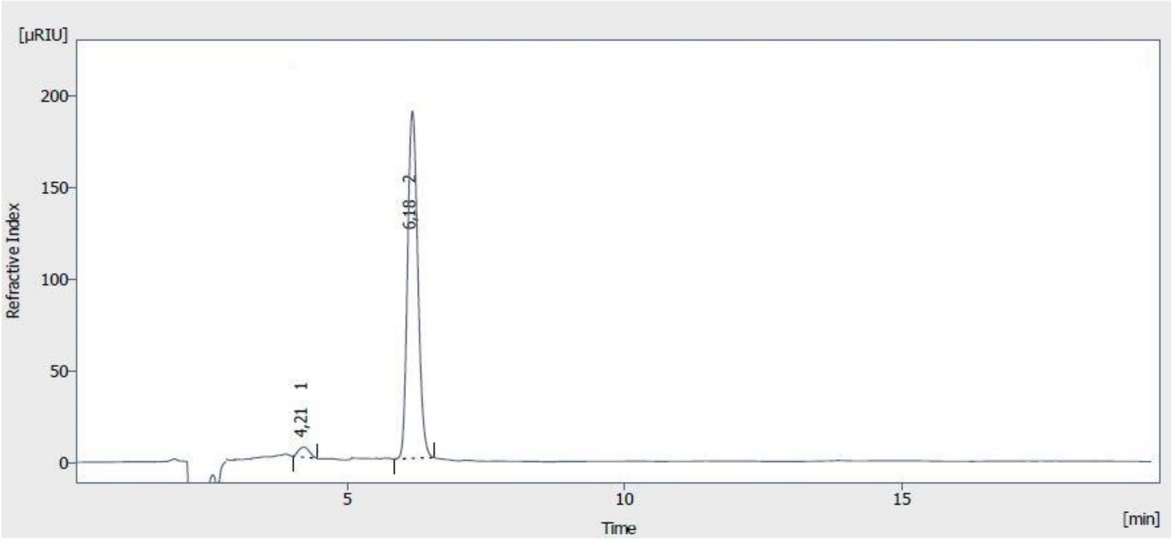

	Reten. Time [min]	Area [µRIU.s]	Height [µRIU]	Area [%]	Height [%]	W05 [min]	Compound Name
1	4,213	76,208	5,581	3,0	2,9	0,23	
2	6,177	2434,713	189,300	97,0	97,1	0,20	
	Total	2510,921	194,881	100,0	100,0		

HPLC trace of compound **2i**

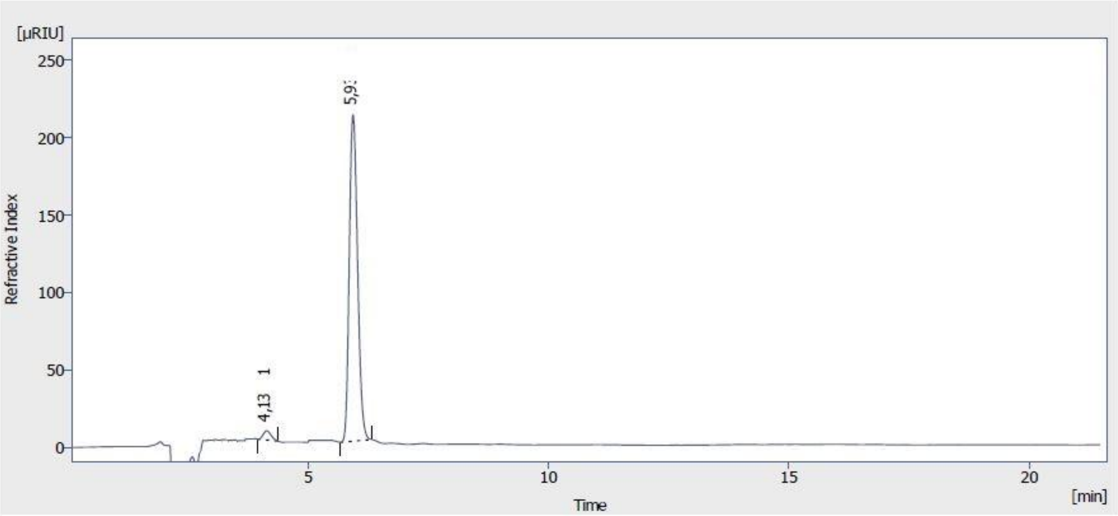

	Reten. Time [min]	Area [µRIU.s]	Height [µRIU]	Area [%]	Height [%]	W05 [min]	Compound Name
1	4,135	67,917	5,985	2,6	2,8	0,18	
2	5,930	2515,514	210,385	97,4	97,2	0,19	
	Total	2583,430	216,370	100,0	100,0		

HPLC trace of compound **2j**

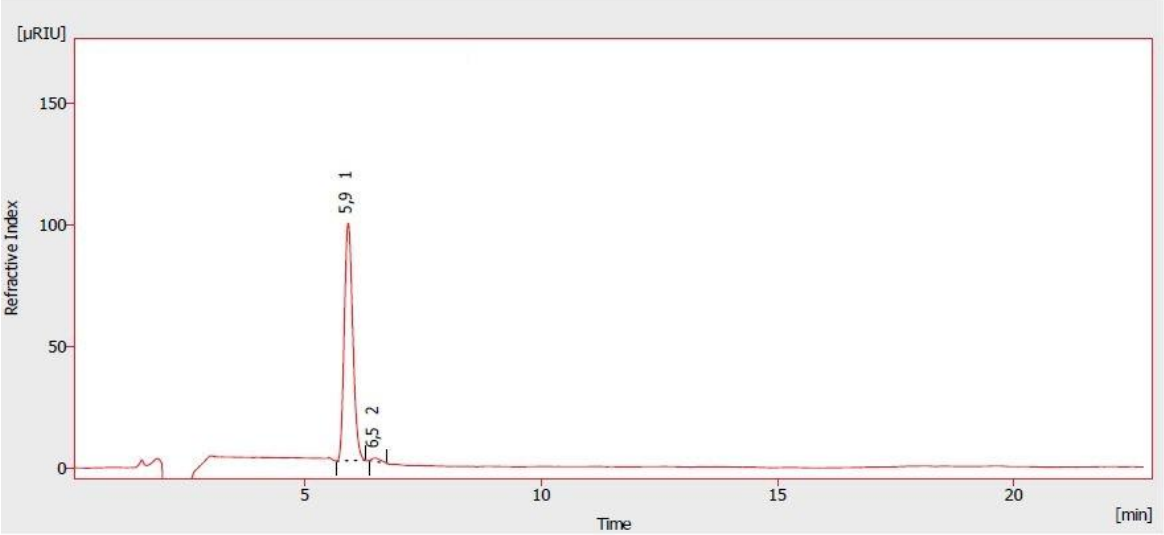

	Reten. Time [min]	Area [µRIU.s]	Height [µRIU]	Area [%]	Height [%]	W05 [min]	Compound Name
1	5,918	1217,398	97,695	98,4	98,5	0,20	
2	6,483	19,268	1,458	1,6	1,5	0,19	
	Total	1236,666	99,154	100,0	100,0		

HPLC trace of compound **2k**

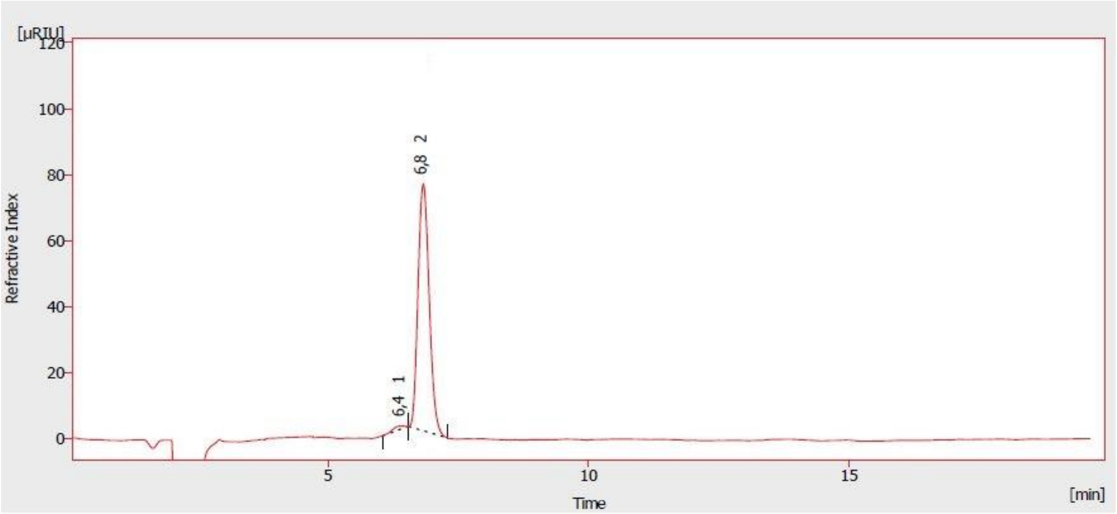

	Reten. Time [min]	Area [μRIU.s]	Height [μRIU]	Area [%]	Height [%]	W05 [min]	Compound Name
1	6,405	16,013	0,936	1,4	1,2	0,23	
2	6,828	1112,234	74,819	98,6	98,8	0,23	
	Total	1128,246	75,756	100,0	100,0		

HPLC trace of compound **2l**

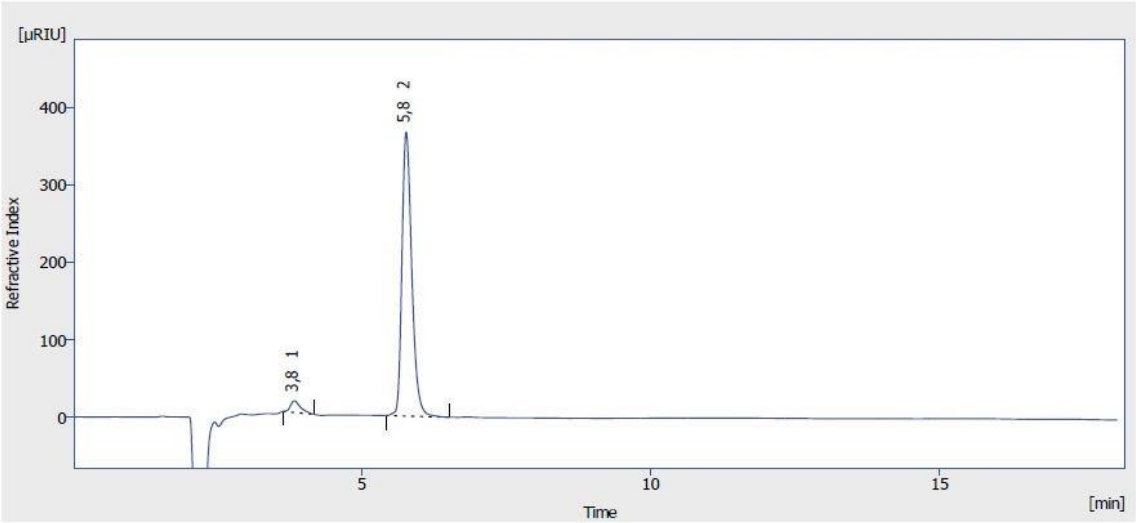

	Reten. Time [min]	Area [μRIU.s]	Height [μRIU]	Area [%]	Height [%]	W05 [min]	Compound Name
1	3,832	188,218	15,187	4,1	4,0	0,17	
2	5,765	4348,996	366,999	95,9	96,0	0,18	
	Total	4537,214	382,186	100,0	100,0		

HPLC trace of compound **2m**

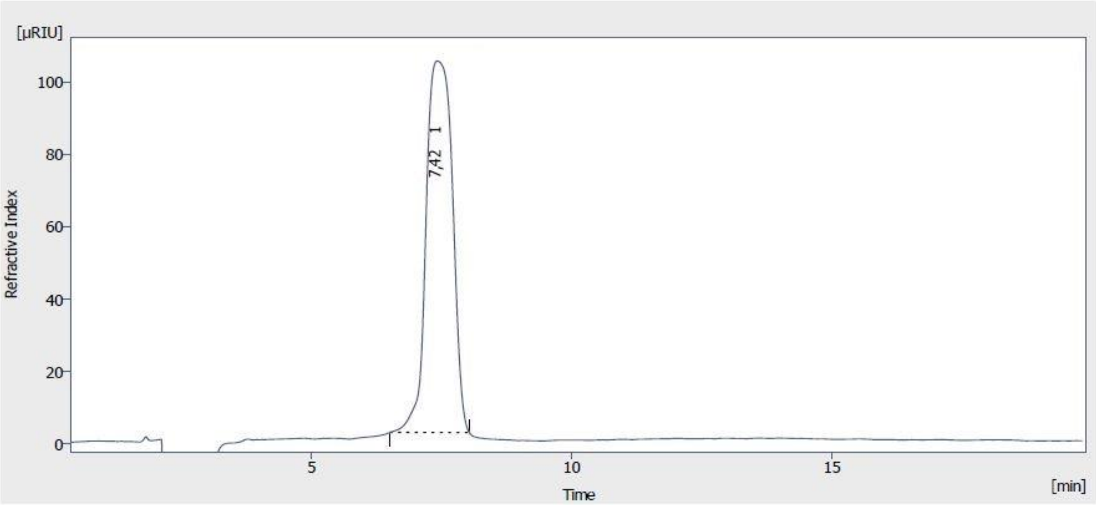

	Reten. Time [min]	Area [µRIU.s]	Height [µRIU]	Area [%]	Height [%]	W05 [min]	Compound Name
1	7,418	3607,354	102,540	100,0	100,0	0,57	
	Total	3607,354	102,540	100,0	100,0		

HPLC trace of compound **2n**

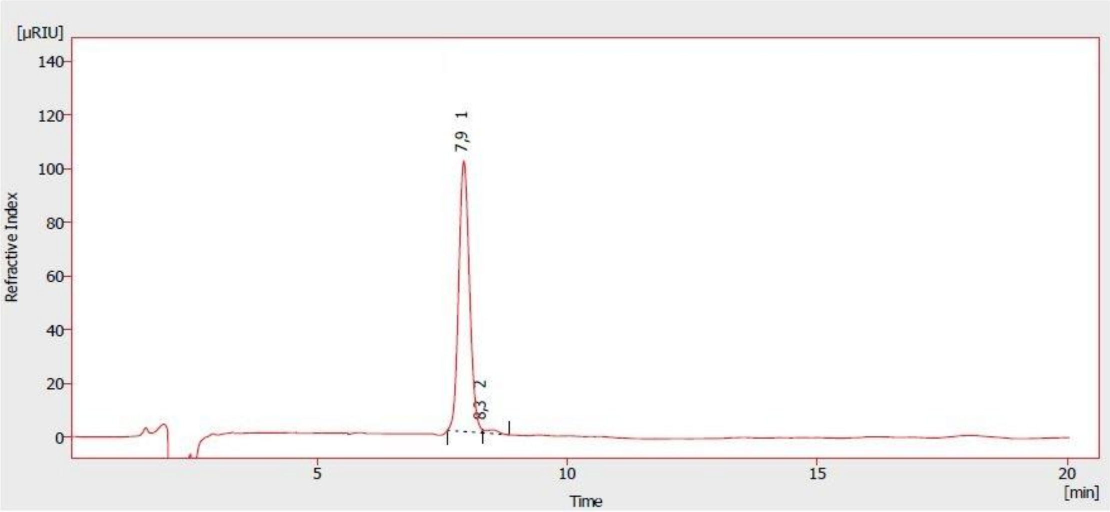

	Reten. Time [min]	Area [µRIU.s]	Height [µRIU]	Area [%]	Height [%]	W05 [min]	Compound Name
1	7,933	1509,474	100,834	98,3	98,8	0,23	
2	8,303	25,766	1,177	1,7	1,2	0,31	
	Total	1535,239	102,011	100,0	100,0		

HPLC trace of compound **2o**

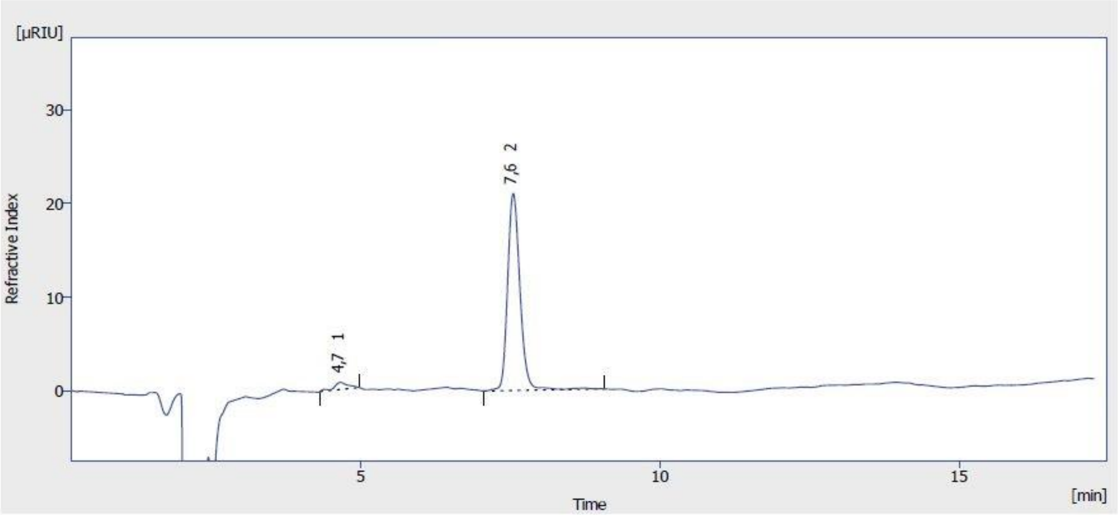

	Reten. Time [min]	Area [µRIU.s]	Height [µRIU]	Area [%]	Height [%]	W05 [min]	Compound Name
1	4,660	12,463	0,764	3,9	3,5	0,29	
2	7,550	303,245	21,009	96,1	96,5	0,22	
	Total	315,708	21,773	100,0	100,0		

HPLC trace of compound **2p**

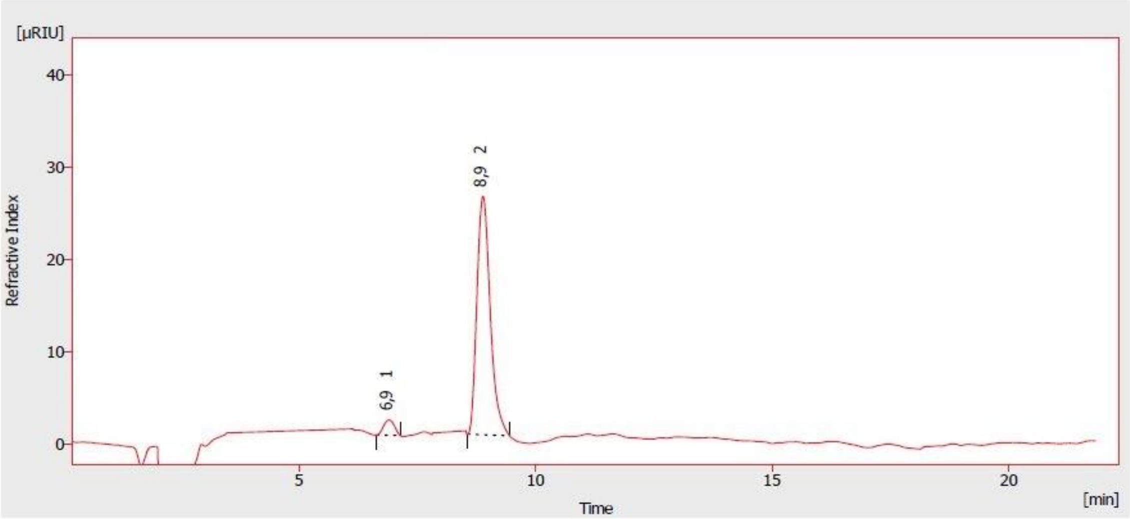

	Reten. Time [min]	Area [µRIU.s]	Height [µRIU]	Area [%]	Height [%]	W05 [min]	Compound Name
1	6,897	26,035	1,648	4,9	6,0	0,27	
2	8,892	506,514	25,809	95,1	94,0	0,30	
	Total	532,549	27,457	100,0	100,0		

HPLC trace of compound **2q**

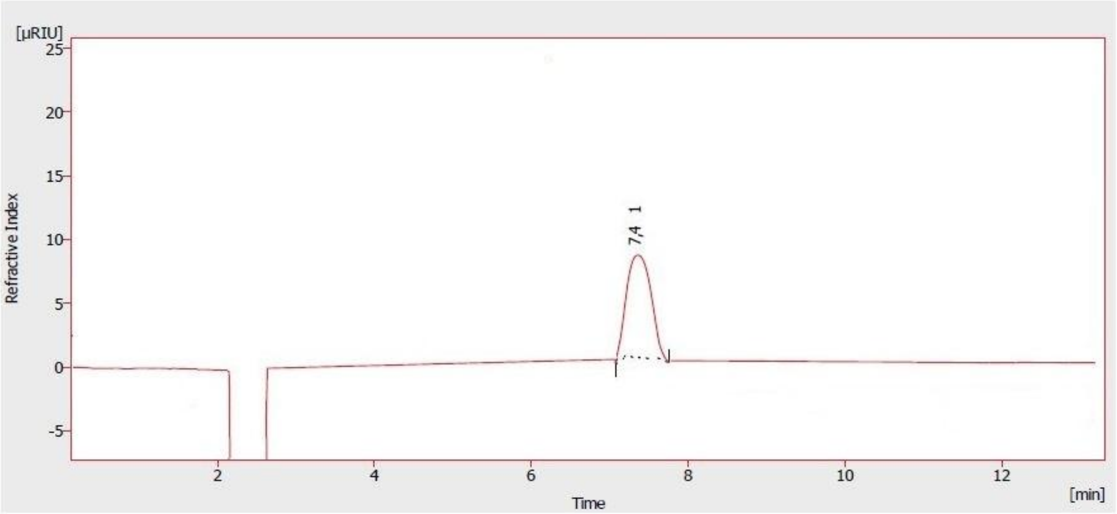

	Reten. Time [min]	Area [µRIU.s]	Height [µRIU]	Area [%]	Height [%]	W05 [min]	Compound Name
1	7,390	196,747	8,571	100,0	100,0	0,38	
	Total	196,747	8,571	100,0	100,0		

HPLC trace of compound **2r**

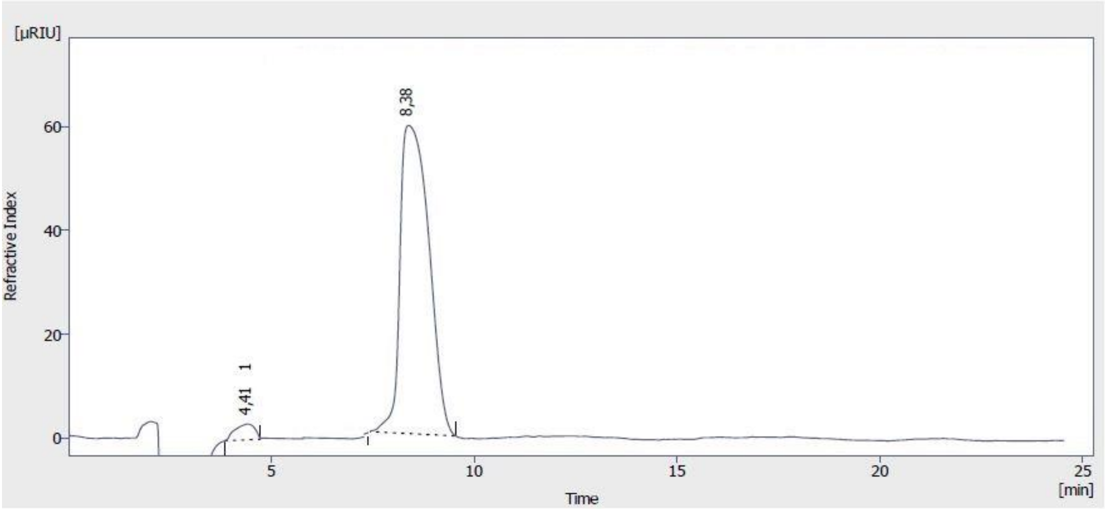

	Reten. Time [min]	Area [µRIU.s]	Height [µRIU]	Area [%]	Height [%]	W05 [min]	Compound Name
1	4,413	103,149	3,034	3,4	4,9	0,62	
2	8,385	2938,265	59,312	96,6	95,1	0,80	
	Total	3041,414	62,346	100,0	100,0		

HPLC trace of compound **2s**

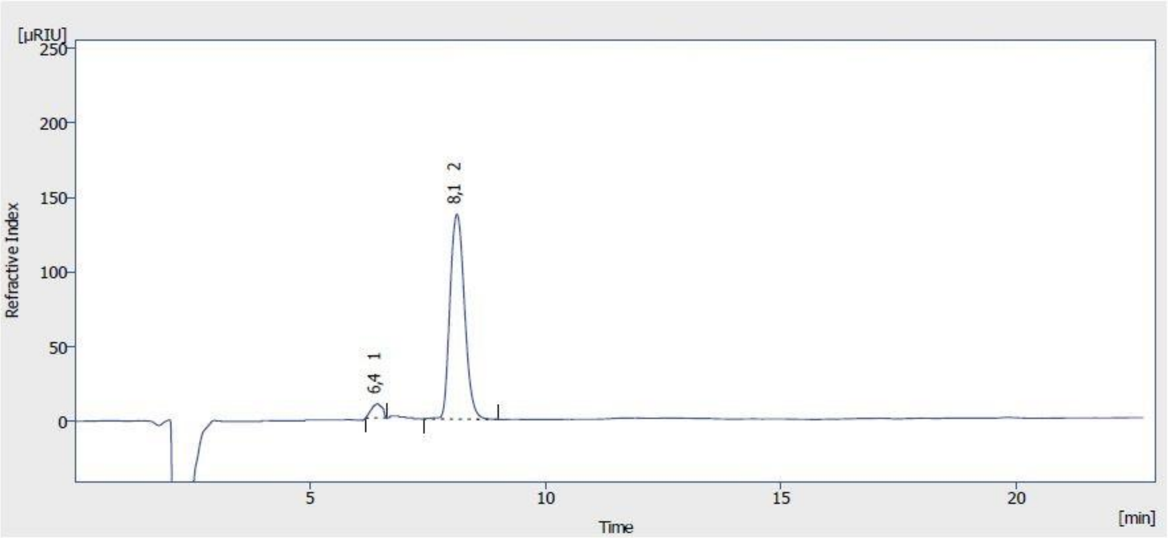

	Reten. Time [min]	Area [µRIU.s]	Height [µRIU]	Area [%]	Height [%]	W05 [min]	Compound Name
1	6,420	150,527	9,368	4,9	6,4	0,29	
2	8,117	2949,487	137,274	95,1	93,6	0,34	
	Total	3100,014	146,642	100,0	100,0		

HPLC trace of compound **2t**

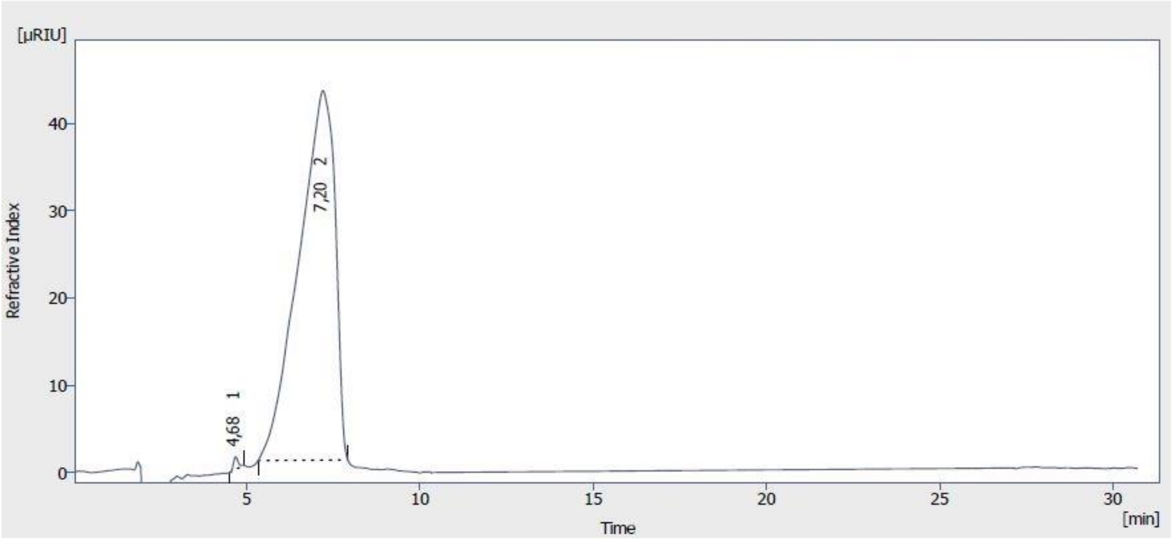

	Reten. Time [min]	Area [µRIU.s]	Height [µRIU]	Area [%]	Height [%]	W05 [min]	Compound Name
1	4,683	13,511	1,455	0,4	3,3	0,15	
2	7,202	3071,398	42,321	99,6	96,7	1,18	
	Total	3084,908	43,777	100,0	100,0		

HRMS spectra of compound **1c**

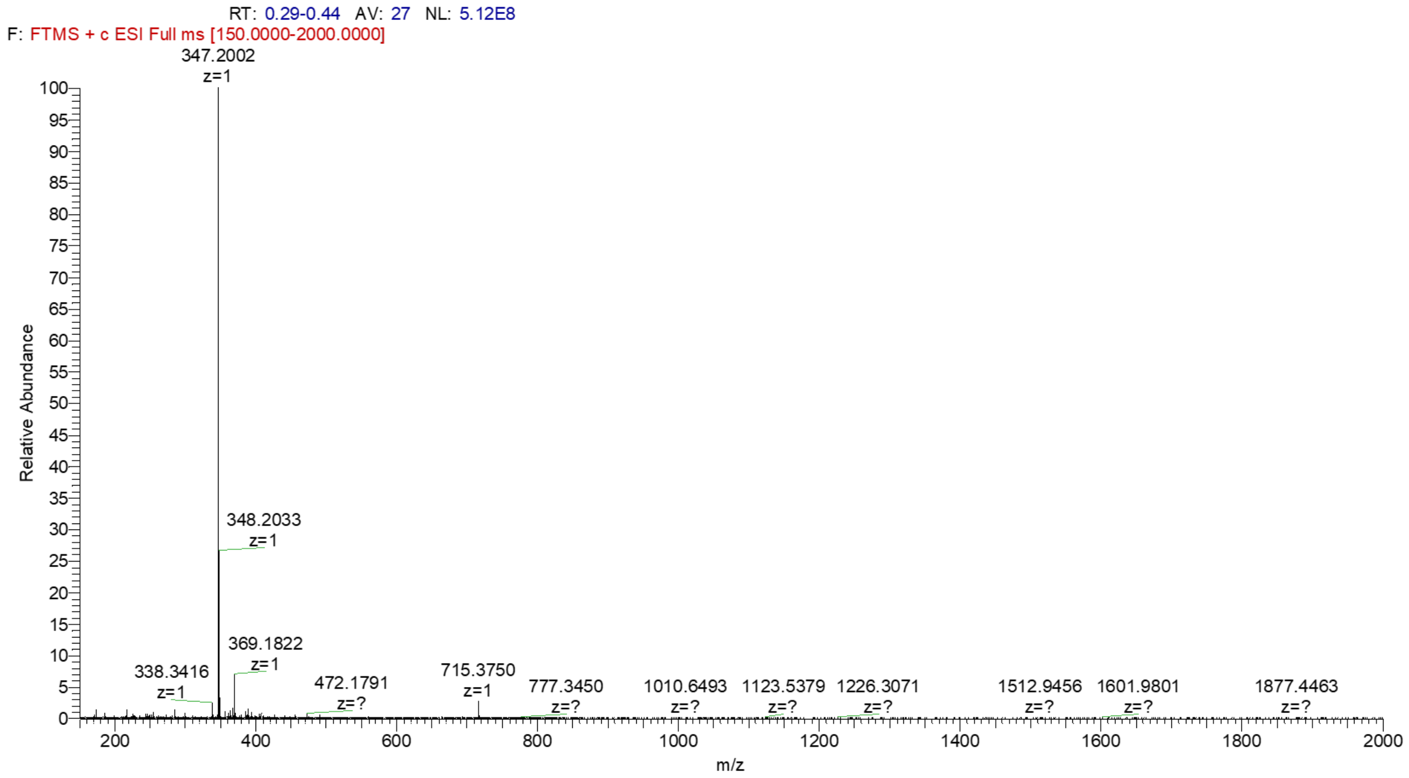

HRMS spectra of compound **2h**

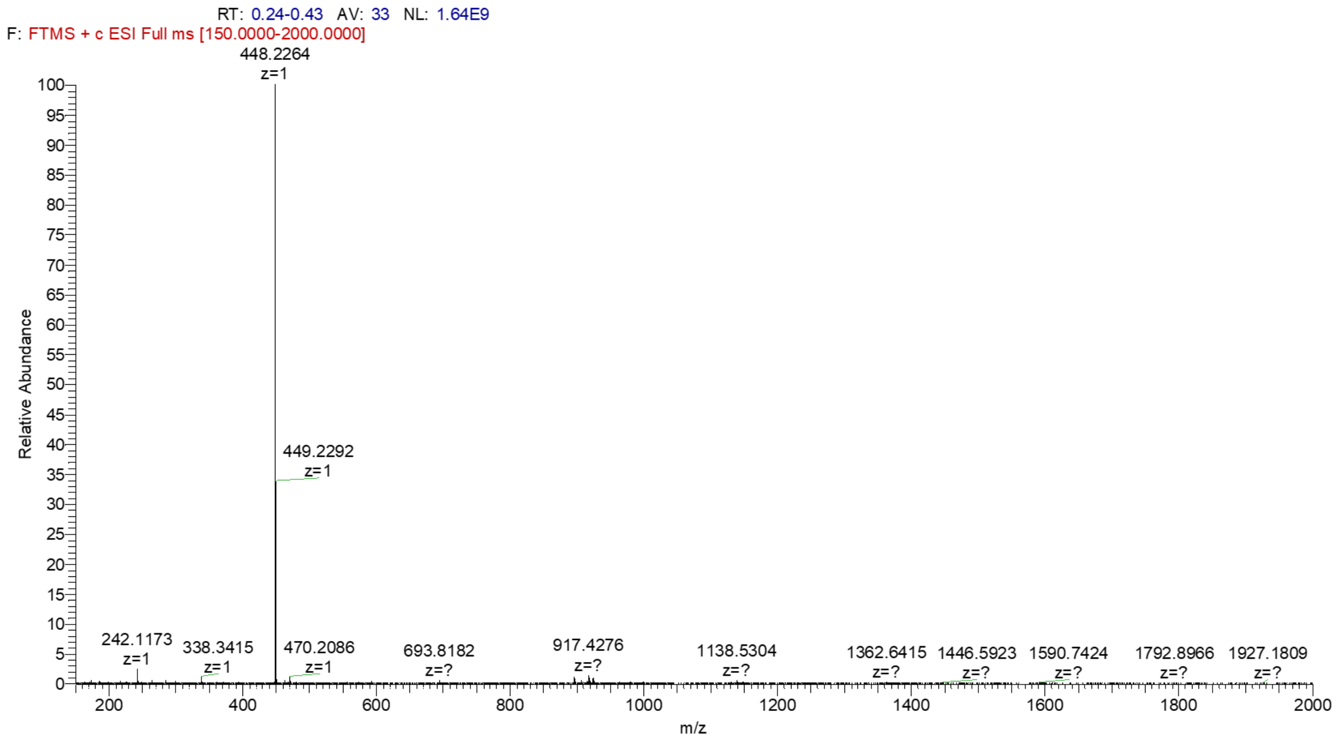

HRMS spectra of compound **2o**

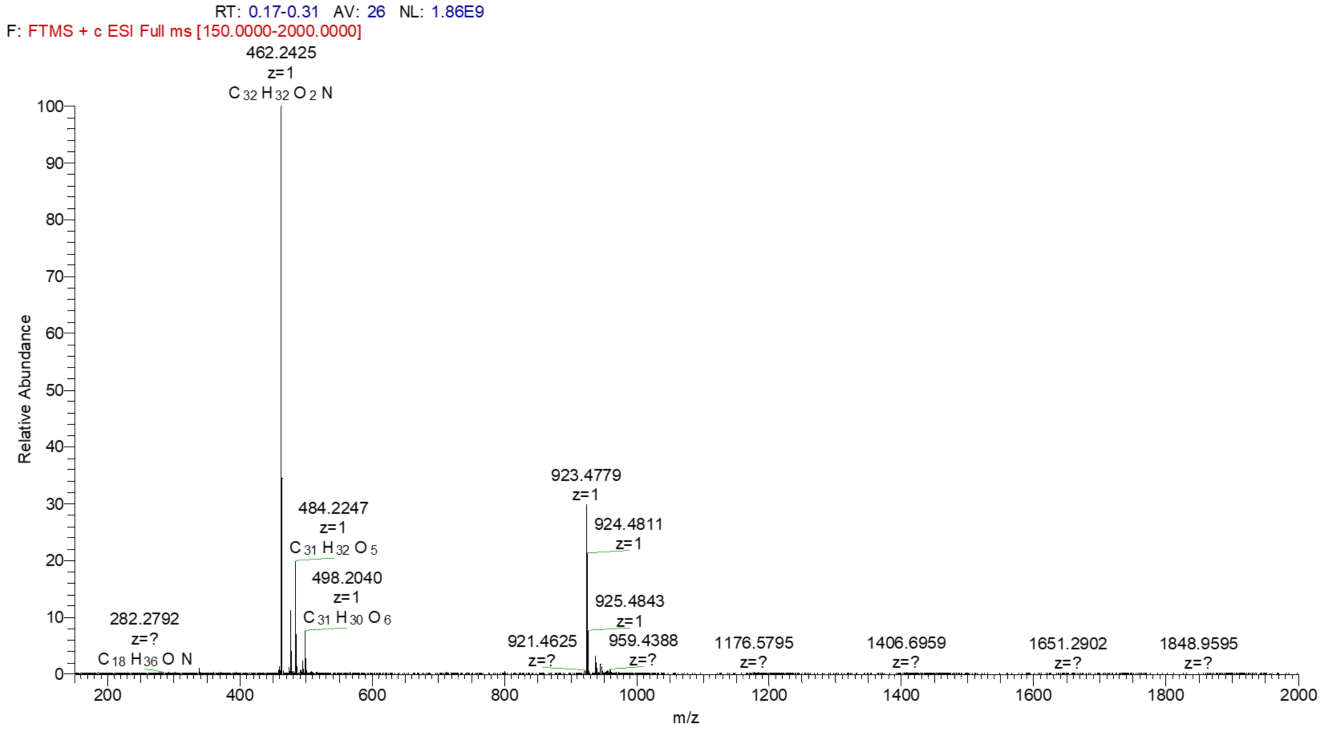

HRMS spectra of compound **2s**

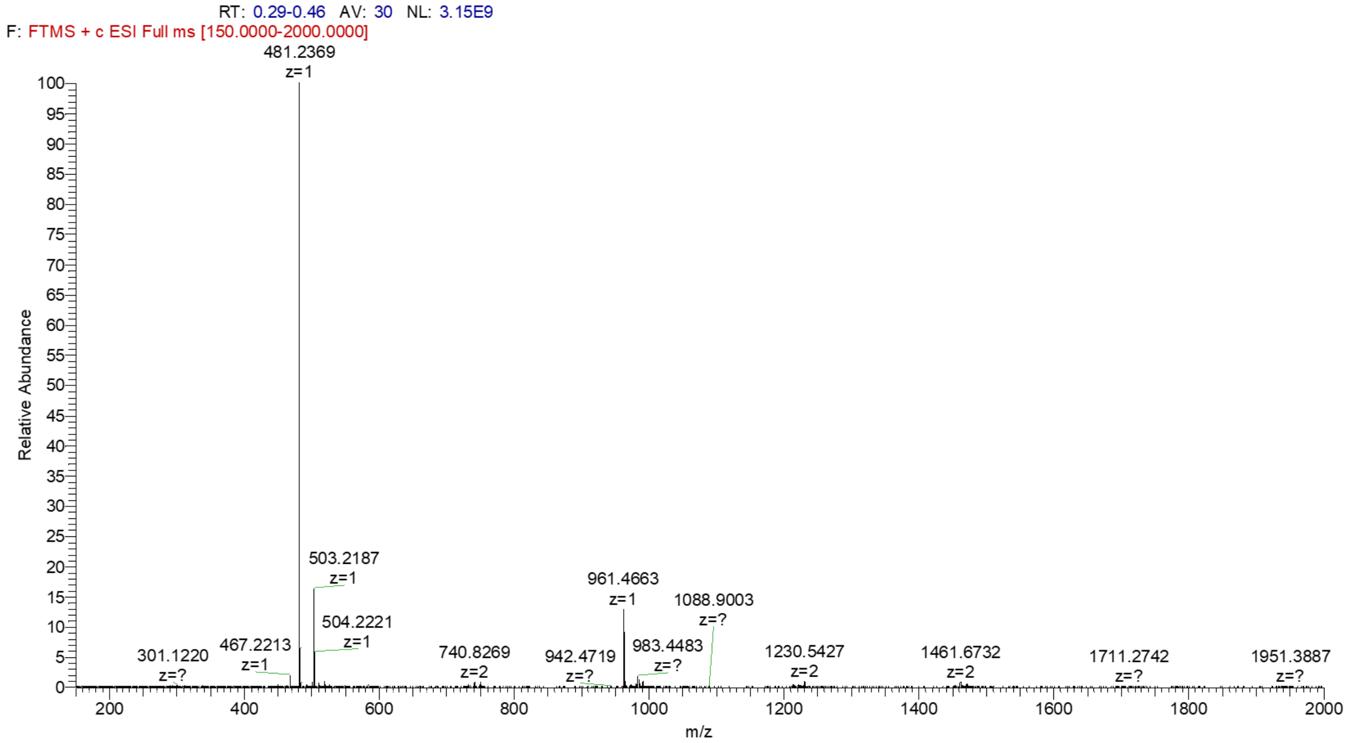

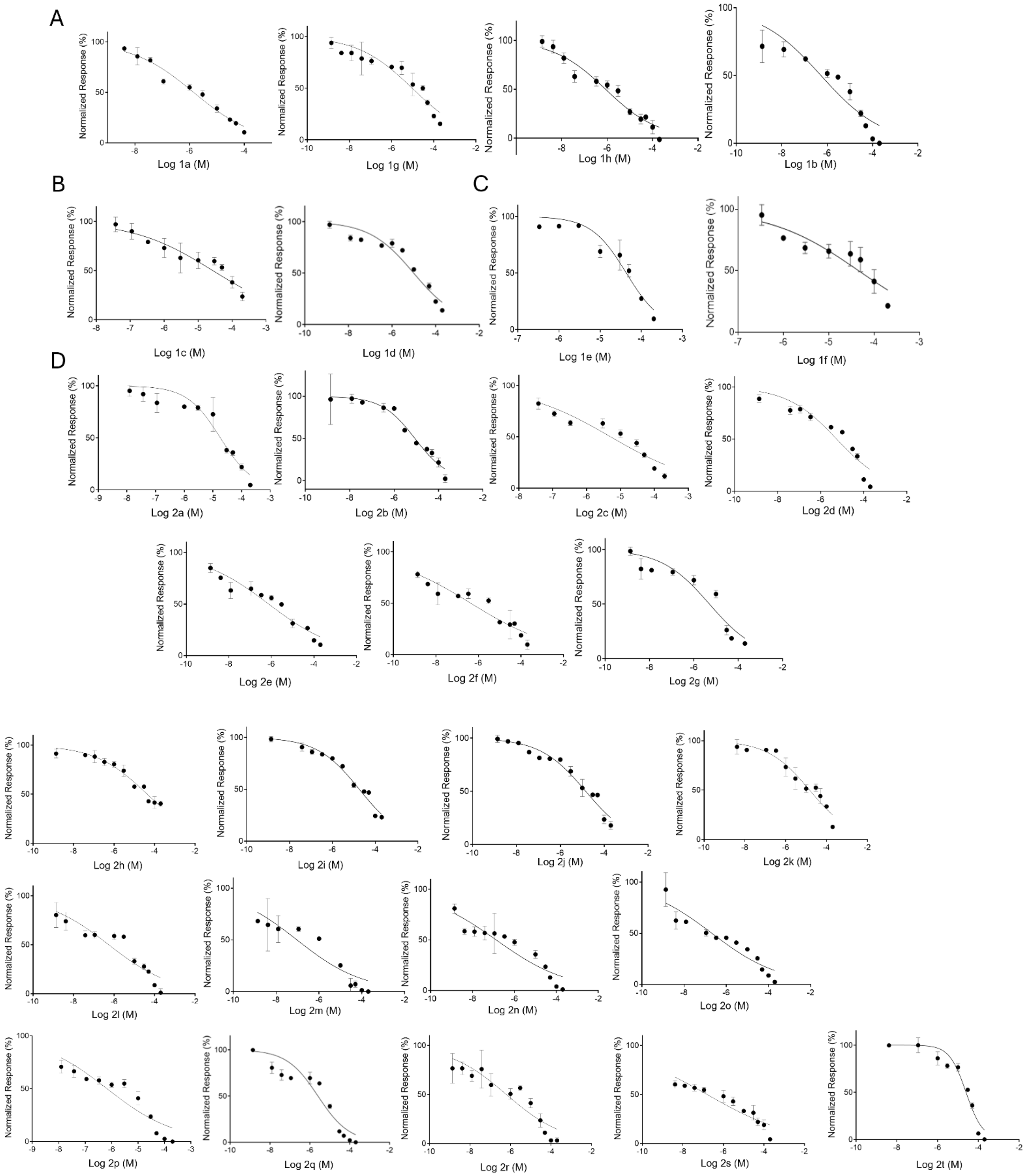

Figure S1. Alpha screen results for the inhibition of LIF/LIFR binding. Panel A shows the IC_{50} curves for the compounds of Table 1; Panels B, C and D show the IC_{50} curves for the compounds of Table 2 and Panel E shows the IC_{50} curves for the compounds of Table 3.

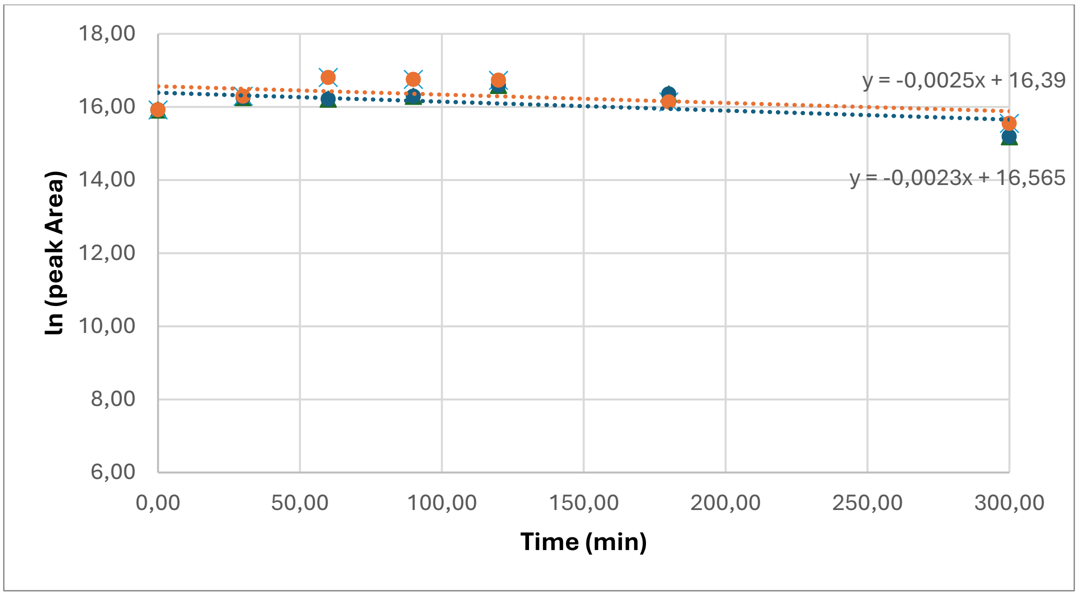

Figure S2. Compound **2o** stability over time incubated with S9 fraction: the ln peak area of compound **2o** as measured by LC-MS is plotted against time up to 300 min.

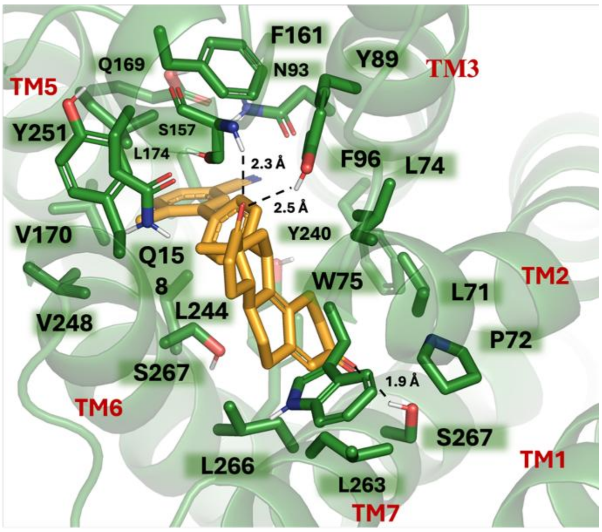

Figure S3. IFD docking results of compound **2o** in GPBAR1-$G\alpha_s\beta_1\gamma_2$ homology model. The ligand is represented as orange sticks, and the interacting residues of the receptor are shown in green and labelled, with oxygen atoms in red and nitrogen in blue. The receptor is represented as ribbons with its helix labelled and H-bonds are displayed as black dashed lines.

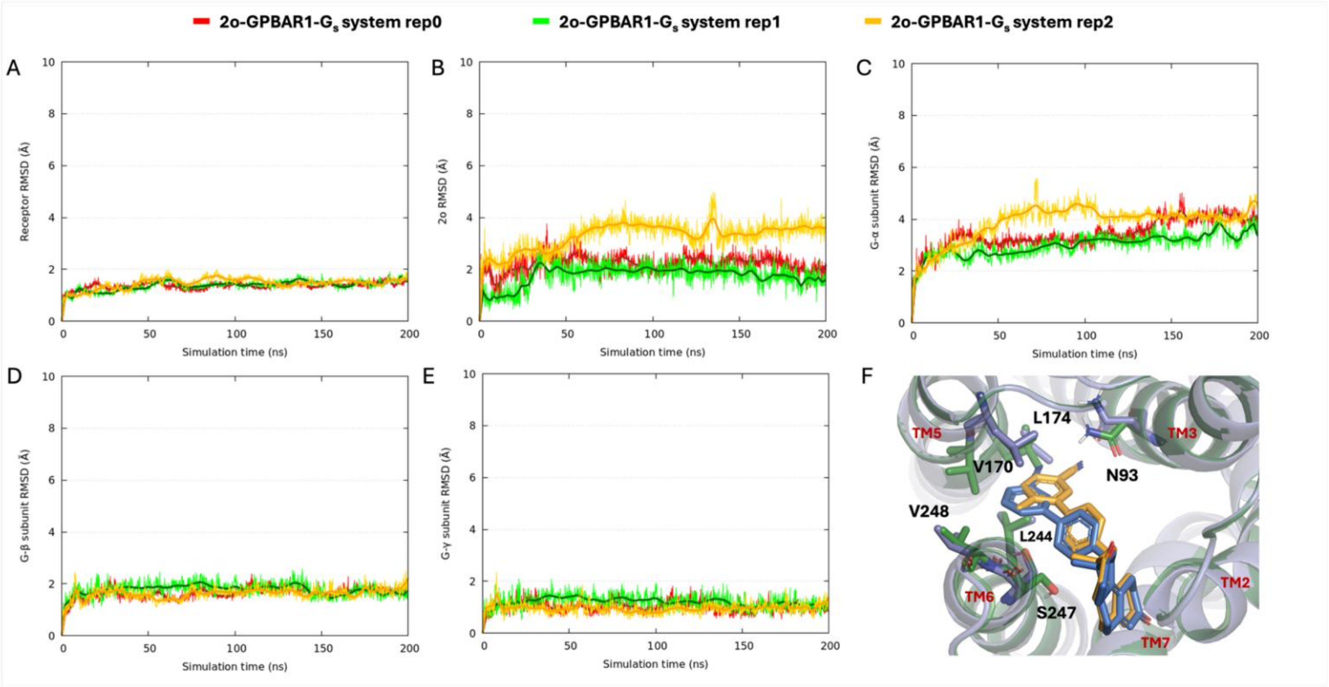

Figure S4. Average RMSD plots of the three MD replicas calculated for: A) the receptor backbone; B) the ligand heavy atoms, and C-E) the backbone of Gα, Gβ, and Gγ subunits, respectively. F) Superimposition of IFD docking results of compound **2f** (in blue sticks) and the one of compound **2o** (in orange sticks) in GPBAR1-$G\alpha_s\beta_1\gamma_2$ homology model (represented in purple and in green cartoon, respectively, with residues in sticks and labelled).

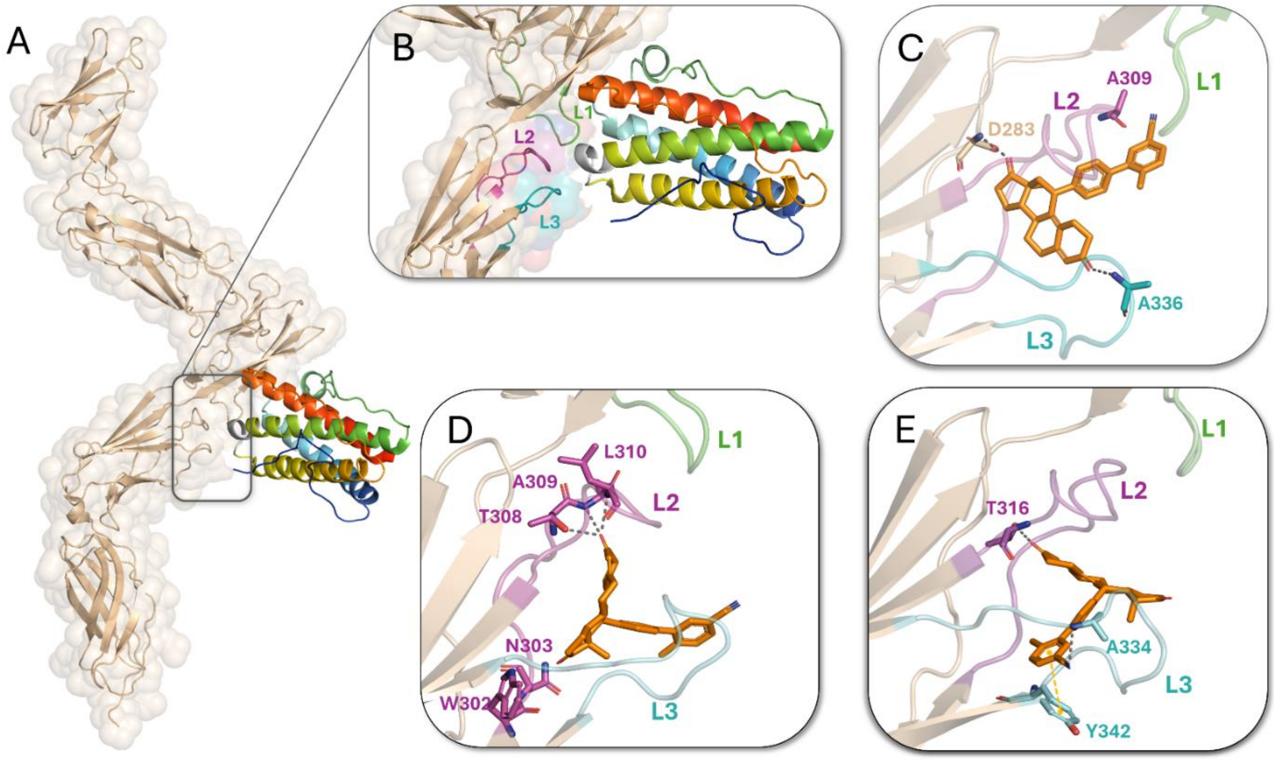

Figure S5. A) X-ray structure of *h*LIFR bound to LIF (PDB ID: 2Q7N); B) focus on the LIF-LIFR binding interface, highlighting the L1-L3 loops; C-D) IFD pose A and pose B, respectively, of compound **2o** in the pocket of LIFR receptor defined by L2-L3 loops; E) The sixth ranked IFD binding mode (pose C) showing a direct interaction (hydrogen bond) of the -C≡N group with A334 of LIFR. Compound **2o** is colored as orange sticks, while L1, L2 and L3 residues of LIFR are colored as green, magenta and cyan sticks, respectively. Hydrogen bond interactions are shown as dashed black lines, while π-π stacking interactions are shown as yellow dashed lines.

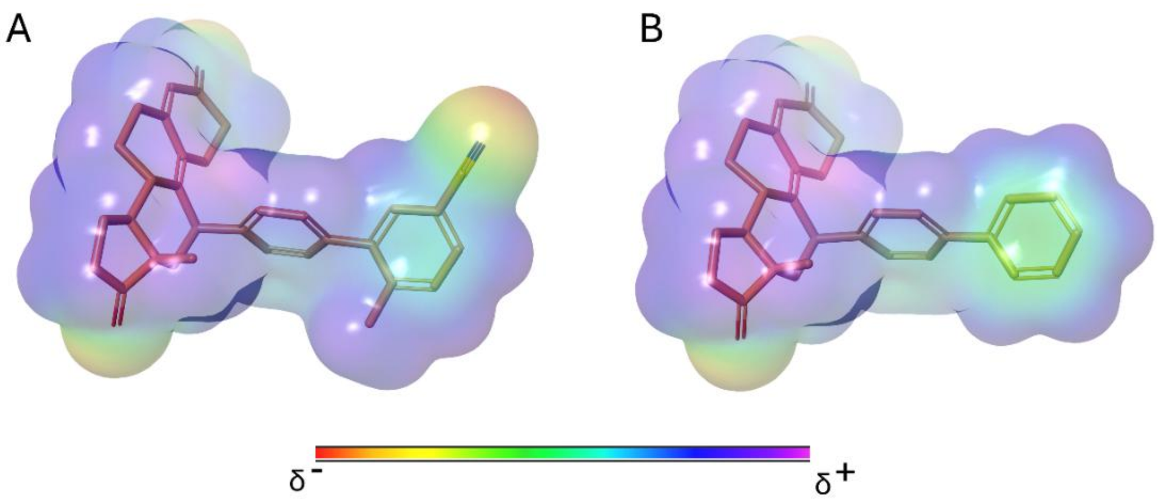

Figure S6. Electrostatic Potential Surface (ESPs) mapped onto electron density isosurfaces of: (A) Compound **2o** compared with that of (B) Compound **1a**. It can be observed how the -C≡N group slightly increases the electrophilicity of the aromatic ring, which instead is more nucleophilic.

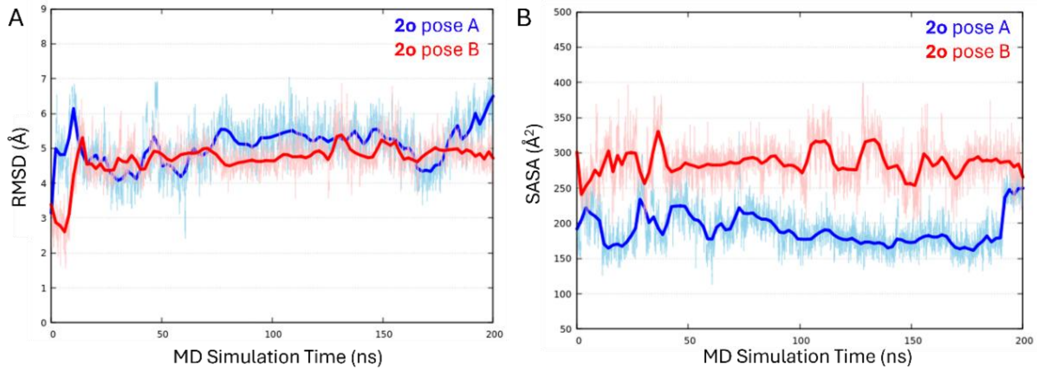

Figure S7. A) Average RMSD plots calculated for the ligand heavy atom in both pose A and B from MDs simulations. B) Average SASA plots calculated in both pose A (blue liens) and B (red lines) from MDs simulations.

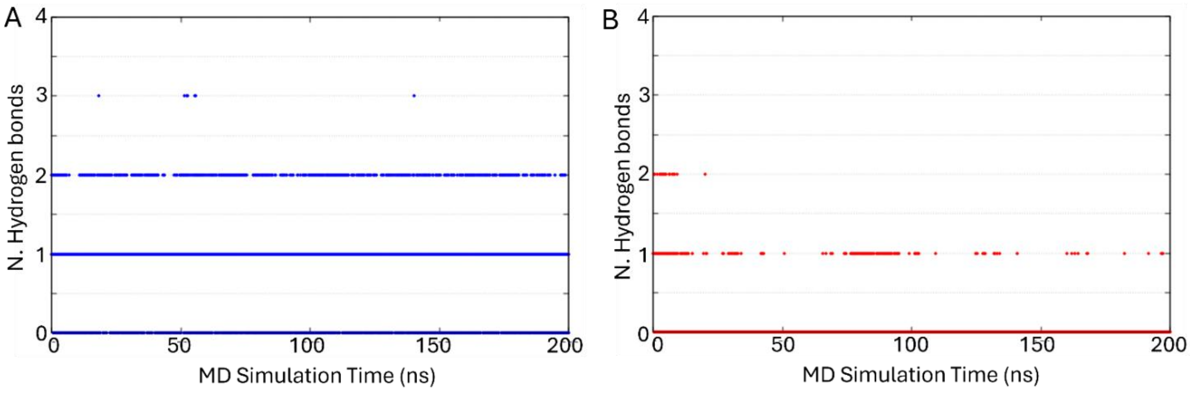

Figure S8. Time-dependent variations within the hydrogen donor-acceptor (HD-A) distances for compound **2o** in pose A (blue) and B (red) during MDs simulation.

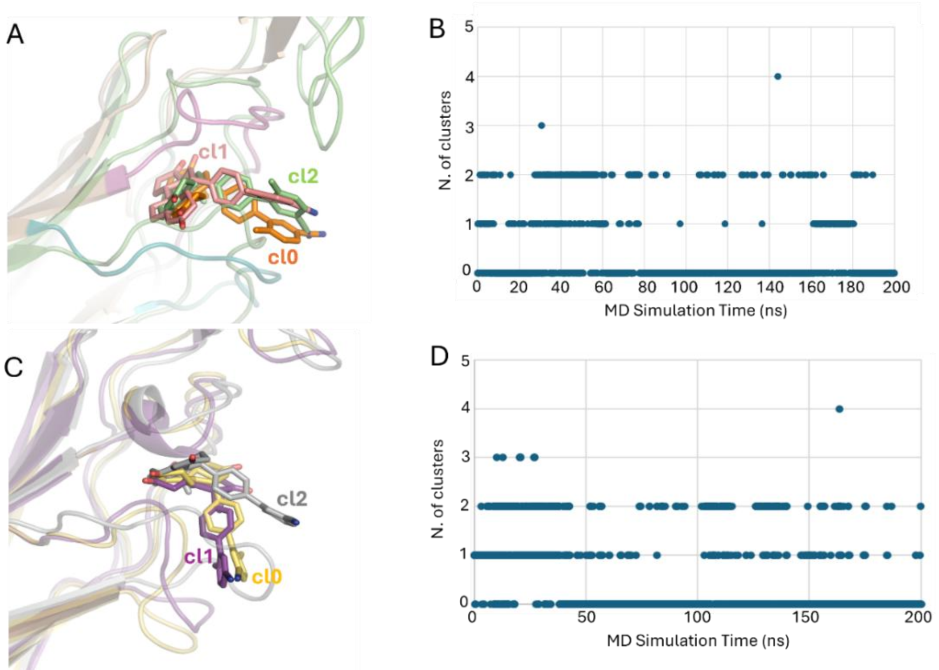

Figure S9. Cluster analysis of 200 ns MDs of compound **2o** in the LIFR receptor. A) Superimposition of the three most populated clusters obtained from Pose A (c0 orange, c1 pink and c2 green) B) Time course analysis of the five clusters, showing the population of cl0, cl1 and cl2 of pose A. C) Superimposition of the most populated clusters obtained from Pose B (c0 yellow, c1 violet and c2 grey), showing the slightly higher variability of the pose B during MDs; D) Time course analysis of the five clusters, showing the population of cl0, cl1 and cl2 of pose B.

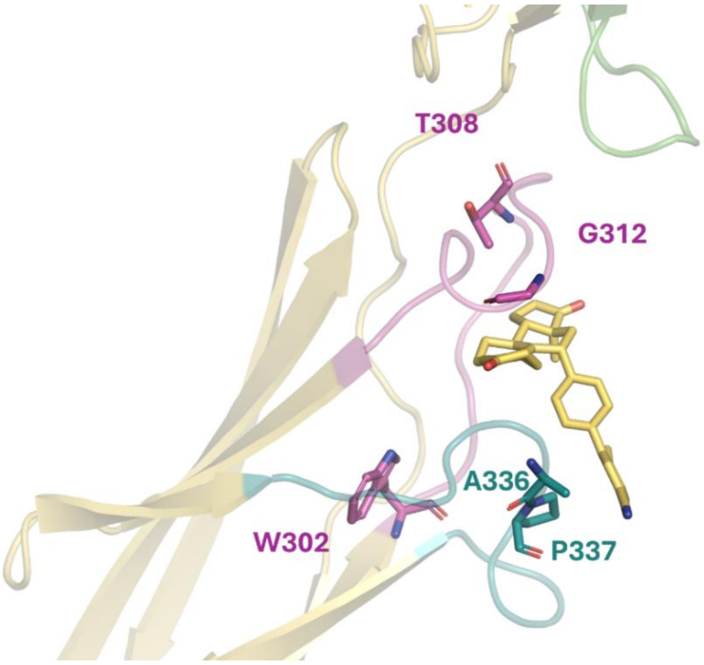

Figure S10. Main populated cluster (c0) of **2o** in pose B, characterized by a less hydrogen bond network and a wider solvent-exposed area.

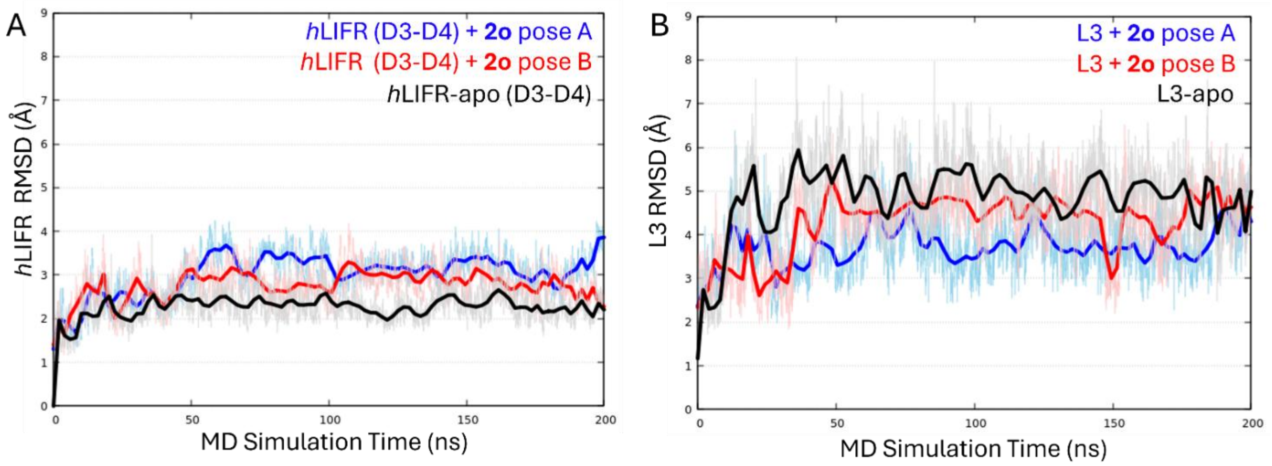

Figure S11. Average RMSD plot calculated on: A) the backbone atoms of the hLIFR D3-D4 domains; B) the backbone atoms of L3 loop.

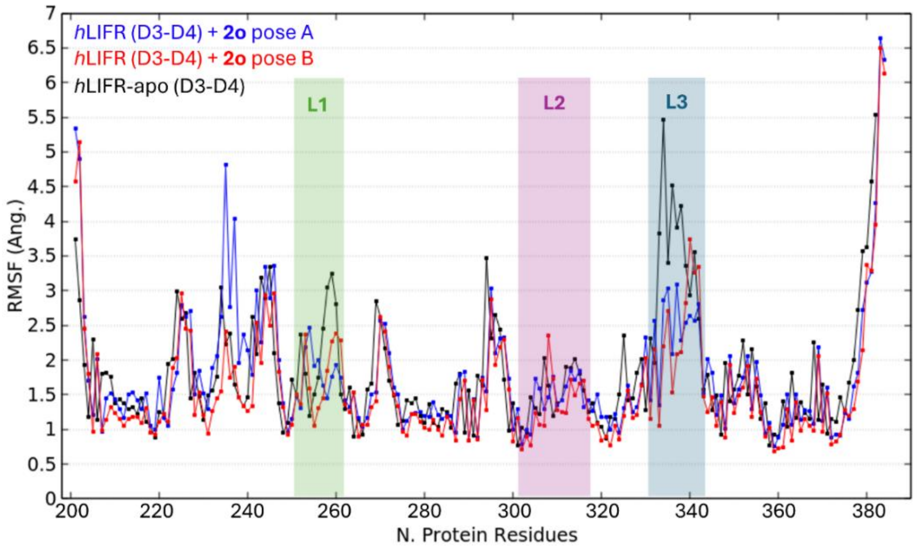

Figure S12. RMSF plot calculated on the hLIFR D3-D4 domains in the apo form and upon binding of **2o** (pose A and B) the backbone atoms of L3 loop.

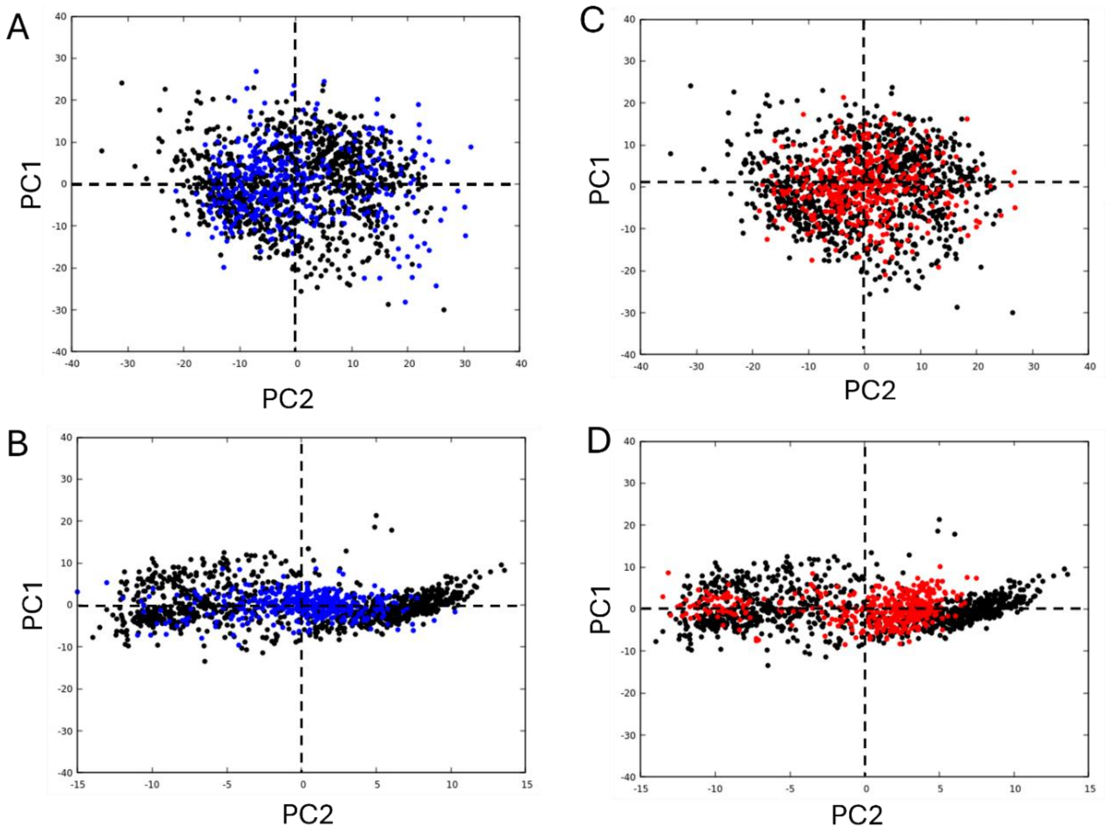

Figure S13. 2D projection of eigenvector 1 and 2 (PC1 and PC2) computed on the receptor's Cα atoms of A) and C) the hLIFR's D3-D4 domains respectively bound to **2o** in pose A (blue points) and pose B (red points); B) and D) on the L3 loop respectively bound to **2o** in pose A (blue points) and pose B (red points). PC1 and PC2 eigenvectors are compared with those of the hLIFR's D3-D4 domains in apo form (black points).

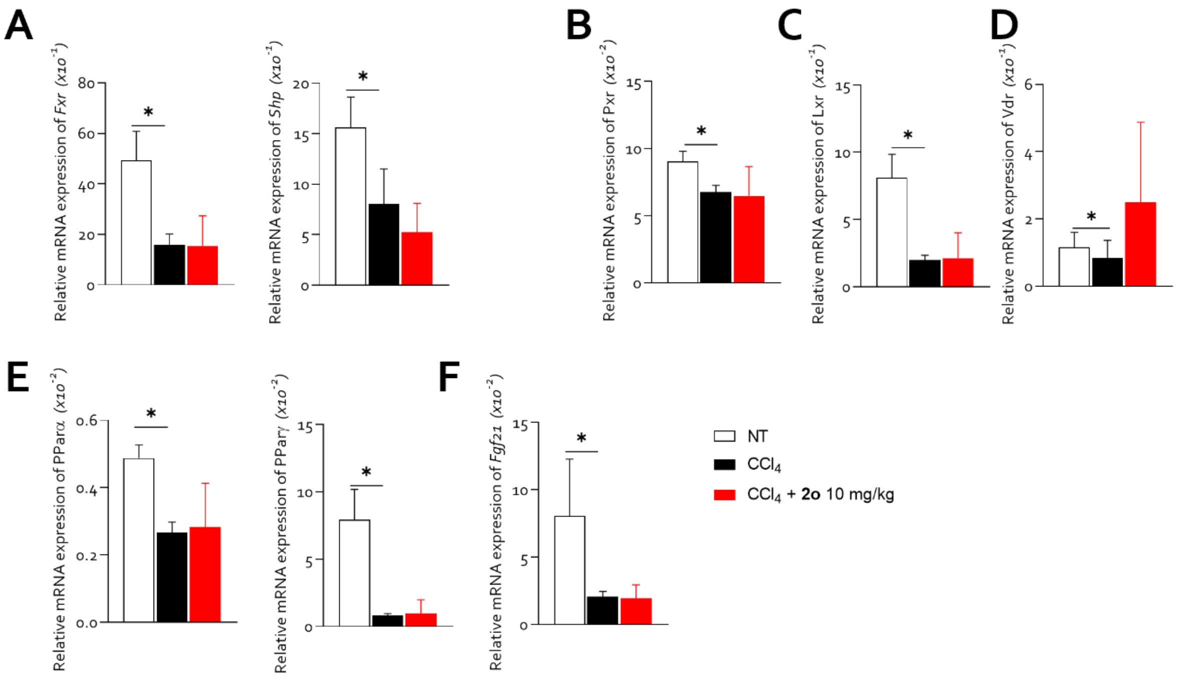

Figure S14. Liver injury was induced in 12-week-old male C57BL/6J mice by intraperitoneal injection of CCl_4 (0.5 mL/kg, twice weekly) for one week, administered either alone or in combination with compound **2o** (10 mg/Kg). To evaluate the selective hepatic activity of the compound, real-time PCR was performed on liver tissue to quantify mRNA expression of: **(A)** *Fxr* and *Shp*; **(B)** *Pxr*; **(C)** *Lxr*; **(D)** *Vdr;* **(E)** *Pparα* and *Pparγ*; **(F)** *Fgf21*. Expression values were normalized to *Gapdh*. Data are presented as mean ± SEM from 5–8 mice per group. $p < 0.05$.
